# LIM-domain-only 4 (LMO4) enhances CD8^+^ T-cell stemness and tumor rejection by boosting IL-21-STAT3 signaling

**DOI:** 10.1038/s41392-024-01915-z

**Published:** 2024-08-09

**Authors:** Roland C. Schelker, Jessica Fioravanti, Fabio Mastrogiovanni, Jeremy G. Baldwin, Nisha Rana, Peng Li, Ping Chen, Timea Vadász, Rosanne Spolski, Christoph Heuser-Loy, Dragana Slavkovic-Lukic, Pedro Noronha, Giuseppe Damiano, Laura Raccosta, Daniela Maggioni, Sree Pullugula, Jian-Xin Lin, Jangsuk Oh, Patrick Grandinetti, Mario Lecce, Leo Hesse, Emilia Kocks, Azucena Martín-Santos, Claudia Gebhard, William G. Telford, Yun Ji, Nicholas P. Restifo, Vincenzo Russo, Michael Rehli, Wolfgang Herr, Warren J. Leonard, Luca Gattinoni

**Affiliations:** 1 Division of Functional Immune Cell Modulation, Leibniz Institute for Immunotherapy, Regensburg, Germany; 2 Center for Cancer Research, National Cancer Institute, National Institutes of Health, Bethesda, MD USA; 3 Laboratory of Molecular Immunology and the Immunology Center, National Heart, Lung and Blood Institute, National Institutes of Health, Bethesda, MD USA; 4 Department of Internal Medicine III, University Hospital Regensburg, Regensburg, Germany; 5 Next Generation Sequencing Core, Leibniz Institute for Immunotherapy, Regensburg, Germany; 6 Immuno-Biotherapy of Melanoma and Solid Tumors Unit, Division of Experimental Oncology, IRCCS Scientific Institute San Raffaele, Milan, Italy; 7 Vita-Salute San Raffaele University, Milan, Italy; 8 University of Regensburg, Regensburg, Germany; 9 National Center for Tumor Diseases, WERA Site, Würzburg-Erlangen-Regensburg-Augsburg, Germany; 10 Center for Immunomedicine in Transplantation and Oncology, University Hospital Regensburg, Regensburg, Germany

**Keywords:** Immunotherapy, Tumour immunology

## Abstract

High frequencies of stem-like memory T cells in infusion products correlate with superior patient outcomes across multiple T cell therapy trials. Herein, we analyzed a published CRISPR activation screening to identify transcriptional regulators that could be harnessed to augment stem-like behavior in CD8^+^ T cells. Using IFN-γ production as a proxy for CD8^+^ T cell terminal differentiation, *LMO4* emerged among the top hits inhibiting the development of effectors cells. Consistently, we found that *Lmo4* was downregulated upon CD8^+^ T cell activation but maintained under culture conditions facilitating the formation of stem-like T cells. By employing a synthetic biology approach to ectopically express LMO4 in antitumor CD8^+^ T cells, we enabled selective expansion and enhanced persistence of transduced cells, while limiting their terminal differentiation and senescence. LMO4 overexpression promoted transcriptional programs regulating stemness, increasing the numbers of stem-like CD8^+^ memory T cells and enhancing their polyfunctionality and recall capacity. When tested in syngeneic and xenograft tumor models, LMO4 overexpression boosted CD8^+^ T cell antitumor immunity, resulting in enhanced tumor regression. Rather than directly modulating gene transcription, LMO4 bound to JAK1 and potentiated STAT3 signaling in response to IL-21, inducing the expression of target genes (*Tcf7*, *Socs3*, *Junb*, and *Zfp36*) crucial for memory responses. CRISPR/Cas9-deletion of *Stat3* nullified the enhanced memory signature conferred by LMO4, thereby abrogating the therapeutic benefit of LMO4 overexpression. These results establish *LMO4* overexpression as an effective strategy to boost CD8^+^ T cell stemness, providing a new synthetic biology tool to bolster the efficacy of T cell-based immunotherapies.

## Introduction

T cell-based therapies are transforming medical oncology, treating previously incurable hematologic cancers, but their efficacy against solid tumors remains disappointing.^1^ Preclinical models^2–5^ and retrospective analyses^6,7^ of T cell therapies have emphasized the crucial role of stem-like memory T cells in mediating successful antitumor responses. Therefore, significant research endeavors have been directed toward unraveling the pathways regulating the formation of these adult stem-like cells.^8^ Knockout studies have been instrumental in identifying key transcriptional regulators that physiologically support long-term immune responsiveness and memory function such as c-MYB,^9^ T cell factor 1 (TCF1),^10^ forkhead box protein O1 (FOXO1),^11^ BTB Domain And CNC Homolog 2 (BACH2),^12^ DNA-binding protein inhibitor ID3,^13^ and Signal Transducer and Activator of Transcription 3 (STAT3).^14^

Synthetic biology offers the opportunity to impart new functions to a cell or to enhance desired features through genetic manipulation of genes that do not normally operate in a specific cell under physiological conditions.^15,16^ To this end, CRISPR activation (CRISPRa) gain-of-function screens are powerful tools for uncovering genes that might not be active under normal conditions but have the potential to promote phenotypes of interest.^17^ By conducting a CRISPRa screen analysis, we identified LIM-domain-only 4 (LMO4) among the top transcriptional regulators that could be harnessed to augment stem-like behavior in CD8^+^ T cells.

LMO4 is a member of the LMO family proteins, which are transcriptional co-regulators essential for determining cell fate and differentiation, as well as regulating organ development.^18^ Much of our understanding of LMO proteins in T cells comes from research on their oncogenic properties.^19^ For instance, LMO1 and LMO2 were initially identified due to their involvement in translocations associated with acute T cell lymphocytic leukemia (T-ALL).^19^ LMO4 is the only LMO family member expressed in mature post-thymic T cells, but the expression is very low when compared to innate immune cell populations (https://www.immgen.org). Furthermore, in contrast to well-established transcription factors regulating memory (e.g., *TCF1*) and effector development (e.g., *PRDM1*),^20^ *LMO4* expression is not dynamically modulated across the CD8^+^ T cell differentiation spectrum (Supplementary Fig. 1), thereby providing an opportunity to exploit its therapeutic potential through synthetic biology.

Here, we demonstrate that LMO4 can be repurposed in CD8^+^ T cells to augment their stemness. Instead of directly influencing gene transcription, LMO4 primarily modulated cytokine signaling, specifically related to interleukin-21 (IL-21). IL-21 is a pleiotropic cytokine with broad functions, including promoting T follicular helper cell and plasma cell differentiation,^21^ but it is also known to cooperatively expand CD8^+^ T cells^22^ and to promote adoptive transfer-mediated antitumor activity.^23,24^ By boosting the IL-21-STAT3 pathway, LMO4 promoted the expression of memory-related factors crucial for the generation of memory T cells while curbing their terminal differentiation. Lastly, our findings reveal the therapeutic potential of LMO4 manipulation: enforcing LMO4 expression in adoptively transferred CD8^+^ T cells significantly boosted CD8^+^ T-cell antitumor immunity, resulting in enhanced tumor clearance and prolonged survival of tumor-bearing animals.

## Results

### CRISPRa screen identifies LMO4 as a candidate gene to enhance CD8^+^ T-cell stemness

**Fig. 1:**
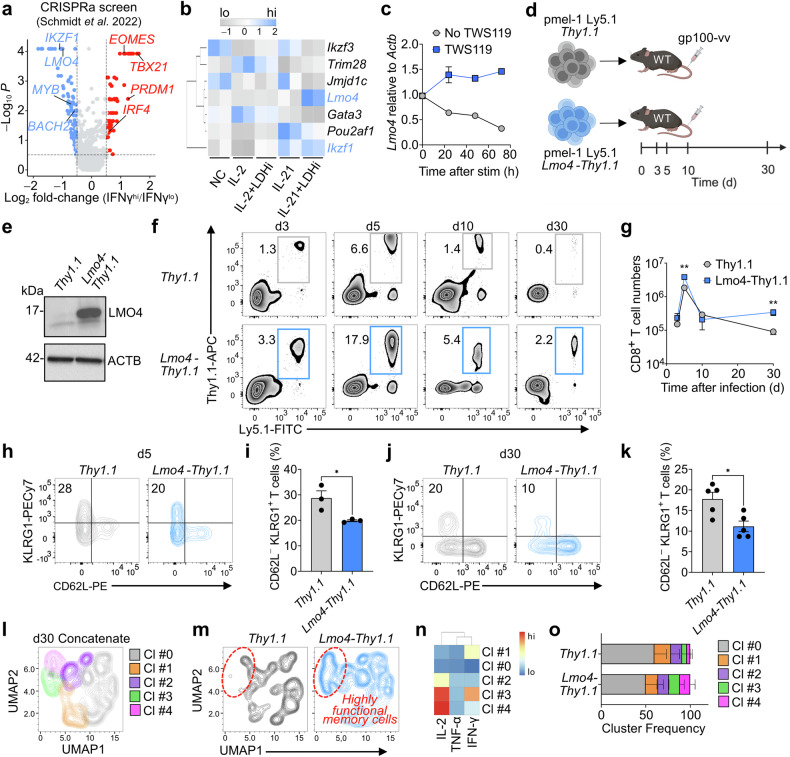
*Lmo4* overexpression enhances CD8^+^ T cell stemness and polyfunctionality. **a** Volcano plot displaying median sgRNA log_2_-fold change (IFN-γ^hi^/^lo^ sorting bin counts) for each gene tested in the genome-wide CRISPRa screen.^17^ Genes included in the analysis were exclusively transcription factors or transcription regulators. **b** Heatmap depicting the expression levels (assessed by RNA-seq) of the top negative hits identified in the CRISPRa screen in CD8^+^ T cells cultured for 4 d under different conditions: No Cytokine (NC), IL-2, IL-2 + LDHi, IL-21, IL-21 + LDHi^23^. **c** q-PCR of *Lmo4* mRNA in CD8^+^ T cells cultured for 72 h with or without TWS119. **d** Experimental design to assess the impact of *Lmo4* overexpression on stem-like CD8^+^ T cell formation. **e** Immunoblot of LMO4 in *Thy1.1* and *Lmo4-Thy1.1* overexpressing T cells. ACTB served as control. **f**, **g** Flow cytometry analysis (**f**) and quantification (**g**) of splenic pmel-1 CD8^+^ T cells following transfer of either 10^5^ pmel-1 Ly5.1^+^ *Thy1.1*^+^ or *Lmo4-Thy1.1*^+^ CD8^+^ T cells into wild-type mice infected with gp100-vv. Assessment was conducted at various time points from 3 to 30 days post-transfer, with three mice per group for each time point. Data are from one representative of 3 experiments. **h**, **i** Flow cytometry analysis (**h**) and percentages (**i**) of CD62L^−^KLRG1^+^ splenic pmel-1 T cells 5 d after transfer as in (**f**, **g**). **j**, **k** Flow cytometry analysis (**j**) and percentages (**k**) of CD62L^−^KLRG1^+^ splenic pmel-1 T cells 30 d after transfer as in (**f**, **g**). **l** UMAP plot of concatenated *Thy1.1*^+^ and *Lmo4-Thy1.1*^+^ pmel-1 CD8^+^ T cells isolated from spleens 5 d after treatment as in (**f**, **g**) showing the distribution of clusters (Cl) identified by FlowSOM. **m** UMAP plot of concatenated *Thy1.1*^+^ and *Lmo4-Thy1.1*^+^ pmel-1 CD8^+^ T cells showing differences in cluster distributions. **n** Heatmap showing the relative expression levels of indicated cytokines in the FlowSOM clusters. **o** Bar plot of *Thy1.1*^+^ and *Lmo4-Thy1.1*^+^ pmel-1 CD8^+^ T cells isolated from spleens 5 d after treatment as in (**f**, **g**) quantifying the distribution of clusters assessed by FlowSOM. *P < 0.05, **P < 0.01 (unpaired two-tailed Student’s *t*-test). (**d**) was created with BioRender.com

To identify transcriptional regulators that could be harnessed to augment stem-like behavior in CD8^+^ T cells beyond physiological function, we analyzed a recently published genome-wide CRISPRa screen dataset.^17^ We used interferon-γ (IFN-γ) production as a proxy for terminal differentiation to select the most potent negative regulators of effector programs. Top positive hits included well-established transcription factors that orchestrate effector differentiation such as *EOMES*, *TBX21*, *PRDM1*, and *IRF4* (Fig. 1a and Supplementary Table 1).^20,25^ *MYB* and *BACH2*, which are known regulators of CD8^+^ T-cell stemness^9,12^ were among the negative hits, validating the robustness of the assay (Fig. 1a and Supplementary Table 1). Notably, except *IKZF1*, which has recently been demonstrated to restrain effector differentiation,^26^ the most significant negative hits have unknown functions in CD8^+^ T-cell memory formation (Supplementary Table 1). To shorten the list of candidate genes, we examined the expression of the top 14 transcriptional regulators in culture conditions favoring the formation of stem-like T cells. We previously showed that stem-like T cells can be efficiently generated by activating CD8^+^ T cells together with IL-21 and a lactate dehydrogenase inhibitor (LDHi).^23^ Several of the candidates, such as *Foxf1*, *Foxf2*, *Foxl2*, *Cebpb*, and *Gata6* were not expressed in CD8^+^ T cells (Supplementary Table 2). Strikingly, only *Lmo4* and *Ikzf1* were upregulated in cells cultured with IL-21 + LDHi compared to cells grown with no cytokine (NC), IL-2, IL-2 + LDHi, or IL-21 (Fig. 1b and Supplementary Table 2). Given the known function of LMO family proteins in cell differentiation, and the as-yet-undetermined role of LMO4 in mature CD8^+^ T cells, we selected this molecule for further investigation. To determine whether the *Lmo4* induction represents a common trait of cells undergoing stem-like T-cell differentiation, we assessed the abundance of *Lmo4* transcripts in CD8^+^ T cells following activation in the presence of TWS119. TWS119 is a glycogen synthase kinase-3β (GSK-3β) inhibitor, which we have shown to drive the formation of both human and mouse stem-like T cells^4,5^ and is being used in a clinical trial evaluating CD19 CAR-modified stem-like T cells (NCT01087294). Priming naive CD8^+^ T cells in the absence of TWS119 downregulated *Lmo4*, whereas *Lmo4* mRNA levels were mildly increased when the GSK-3β inhibitor was added to the culture medium (Fig. 1c). Together, these results reveal that the maintenance of *Lmo4* is a common feature of stem-like T cells, suggesting that modulating its expression might regulate CD8^+^ T-cell stemness.

### Ectopic LMO4 expression boosts the generation of stem-like T cells while limiting their terminal differentiation

To ascertain if CD8^+^ T-cell stemness could be enhanced by increasing LMO4 expression at supraphysiological levels, we employed a synthetic biology approach to overexpress *Lmo4* in CD8^+^ T cells and measured their capacity to generate long-lived memory cells. We transduced pmel-1 Ly5.1^+^ T cells (which recognize the shared melanoma–melanocyte differentiation antigen gp100) with *Lmo4*-*Thy1.1* or *Thy1.1* and transferred them into wild-type mice infected with gp100-vv (Fig. 1d). Overexpression of LMO4 protein was confirmed by western blotting before adoptive T-cell transfer (Fig. 1e). We found that enforced expression of *Lmo4* enhanced the expansion of pmel-1 T cells, which accumulated in the spleen at almost three-fold the frequency of *Thy1.1* cells during the expansion (day 3) and at the peak (day 5) of the immune response (Fig. 1f, g). Similar findings were observed in lymph nodes and lungs (Supplementary Fig. 2), indicating that LMO4 overexpression enhances the accumulation of antigen-specific T cells in both lymphoid and peripheral tissues. These differences were amplified in the memory phase of the immune response as manifested by a five-fold increase in pmel-1 *Lmo4-Thy1.1* T-cell frequencies and numbers 30 days after infection (Fig. 1f, g). Similarly, we observed a notable increase in pmel-1 T cells overexpressing *Lmo4* on day 30 in the lungs and an even more pronounced accumulation of these cells in the lymph nodes, which are known to serve as a niche for stem-like T cells^27^ (Supplementary Fig. 2a–d).

We next investigated whether LMO4 overexpression could also qualitatively affect the immune response by altering the frequencies of CD8^+^ T-cell effector and memory subsets. We found that enforced expression of *Lmo4* restrained terminal effector differentiation, resulting in lower frequencies of CD62L^−^KLRG1^+^ short-lived effectors compared to control *Thy1.1* cells both at the peak of expansion (day 5) and in the late memory phase (day 30) (Fig. 1h–k). Conversely, knocking out *Lmo4* (Supplementary Fig. 3a, b) did not significantly impact pmel-1 T cell accumulation (Supplementary Fig. 3c–e), but resulted in higher frequencies of CD62L^−^KLRG1^+^ short-lived effectors and a decline in CD62L^+^KLRG1^−^ memory precursor compared to pmel-1 *Lmo4*^fl/fl^ T cells at day 5 (Supplementary Fig. 3f–h). These findings underscore that LMO4 exerts some influence on the control of T-cell differentiation, even when expressed at low physiological levels. As T cells undergo differentiation into terminal effectors, they progressively lose the ability to secrete various types of cytokines, ultimately transitioning into monofunctional IFN-γ producers. To determine if LMO4 overexpression also affects CD8^+^ T-cell function, we measured IL-2, TNF-α, and IFN-γ production in memory T cells by intracellular cytokine staining (ICS). We found that LMO4-overexpressing cells not only exhibited increased frequencies of cytokine-producing cells but that most of these cells displayed stronger polyfunctionality (i.e., produced 2 or more cytokines) (Fig. 1l–o). Notably, the ability of LMO4-transduced CD8^+^ T cells to release IL-2 could serve as a potential mechanism for sustaining their proliferative advantage through autocrine or paracrine signals.^28^ Together, these findings underscore that increasing *Lmo4* expression in CD8^+^ T cells is an effective approach to enhance the generation of polyfunctional stem-like memory T cells.

### LMO4 overexpression enhances CD8^+^ T-cell recall responses

**Fig. 2:**
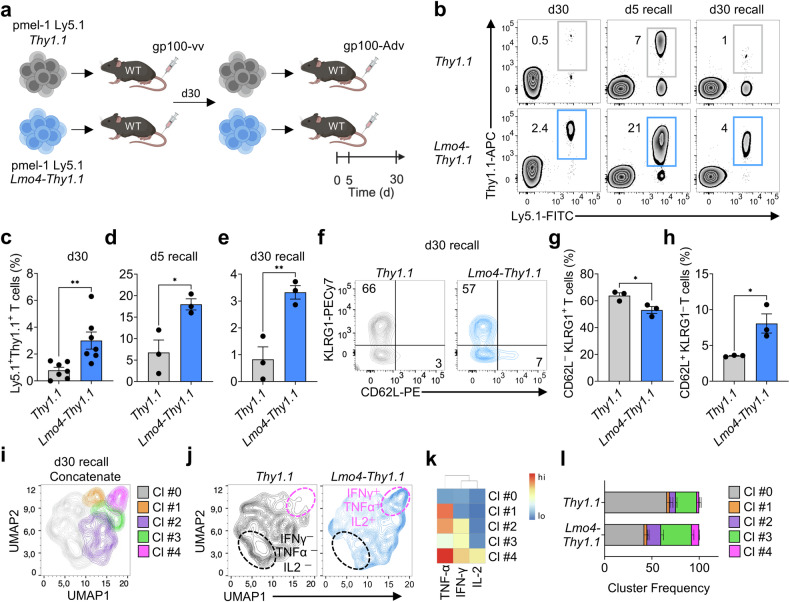
Increased expression of *Lmo4* augments CD8^+^ T cell recall responses. **a** Experimental design investigating the impact of *Lmo4* overexpression on CD8^+^ T cell secondary responses. **b**–**e** Flow cytometry analysis (**b**) and percentages (**c**–**e**) of splenic pmel-1 CD8^+^ T cells following transfer of either 10^5^ pmel-1 Ly5.1^+^ *Thy1.1*^+^ or *Lmo4-Thy1.1*^+^ CD8^+^ T cells into wild-type mice infected with gp100-vv (primary infection) or gp100-Adv (secondary infection). Assessment was conducted at various time points: d30 after primary infection and d5 or d30 after secondary infection (recall); with seven mice per group for d30 and three mice per group for d5 recall and d30 recall. **f**–**h** Flow cytometry analysis (**f**) and percentages of CD62L^−^KLRG1^+^ (**g**) and CD62L^+^KLRG1^−^ (**h**) splenic pmel-1 T cells 5 d after transfer as in (**b**–**e**). **i** UMAP plot of concatenated *Thy1.1*^+^ and *Lmo4-Thy1.1*^+^ pmel-1 CD8^+^ T cells isolated from spleens 30 d after secondary transfer as in (**b**–**e**) showing the distribution of clusters (Cl) identified by FlowSOM. **j** UMAP plot of *Thy1.1*^+^ and *Lmo4-Thy1.1*^+^ pmel-1 CD8^+^ T cells showing differences in cluster distributions. **k** Heatmap showing the relative expression levels of indicated cytokines in the FlowSOM clusters. **l** Bar plot of *Thy1.1*^+^ and *Lmo4-Thy1.1*^+^ pmel-1 CD8^+^ T cells isolated from spleens 5 d after treatment as in (**b**–**e**) quantifying the distribution of clusters assessed by FlowSOM. *P < 0.05, **P < 0.01 (unpaired two-tailed Student’s *t*-test). (**a**) was created with BioRender.com

Having established LMO4 as a key regulator of CD8^+^ T-cell differentiation and formation of stem-like memory T cells, we next investigated if LMO4-overexpression could promote more robust recall responses after secondary infection. Thirty days after primary infection with gp100-vv, we isolated pmel-1 T cells overexpressing *Lmo4-Thy1.1* or *Thy1.1* and transferred them in equal numbers into new wild-type hosts together with a type 2 adenovirus encoding gp100 (gp100-Adv) (Fig. 2a). Strikingly, as in primary infection, we observed an increased expansion of LMO4-overexpressing T cells at the peak of the recall response (day 5) and an even greater accumulation in the secondary memory phase (day 30) in all organs examined (Fig. 2b–e and Supplementary Fig. 2). Similar to primary infection, we detected marked differences in T-cell accumulation in lymph node reservoirs (Supplementary Fig. 2a, b). Repetitive antigen stimulation is recognized to drive CD8^+^ T cells towards senescence, while progressively reducing the pool of stem-like T cells.^29^ We found that enforced expression of *Lmo4* reduced the percentage of CD62L^−^KLRG1^+^ terminal effectors whereas it doubled the frequencies of stem-like CD62L^+^KLRG1^−^ T cells 30 days after secondary infection with gp100-Adv (Fig. 2f–h). Again, we did not observe significant defects in pmel-1 T-cell recall expansion when *Lmo4* was knocked out (Supplementary Fig. 3i, j), but we consistently observed greater differentiation into KLRG1^+^ effectors in pmel-1 *Lmo4*^Δ/Δ^ T cells compared to controls (Supplementary Fig. 3k–m). Similar to primary infections, we also observed that overexpression of LMO4 boosted secondary memory CD8^+^ T-cell polyfunctionality (Fig. 2i–l). Altogether, these results show that elevated levels of LMO4 enhance CD8^+^ T-cell recall responses and facilitate the maintenance of a robust pool of stem-like T cells.

### Enforced expression of LMO4 promotes superior CD8^+^ T-cell antitumor responses

**Fig. 3:**
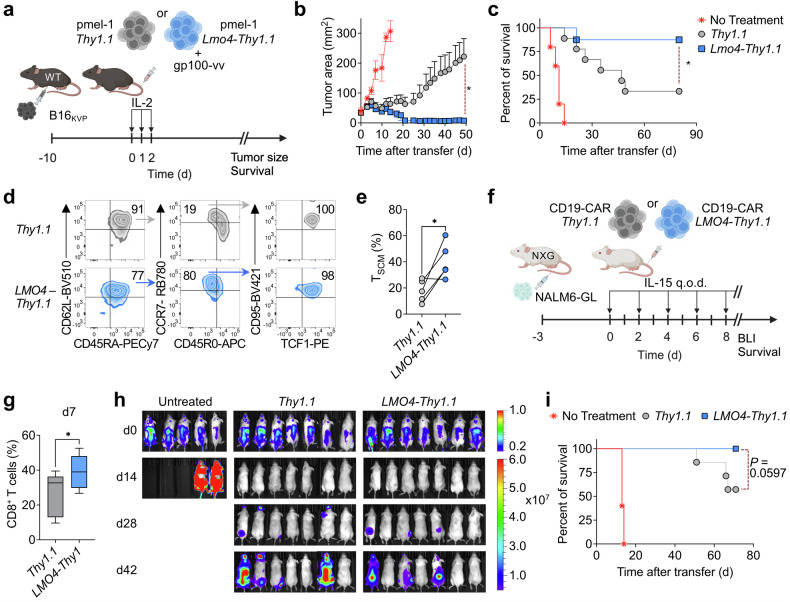
Enforced expression of *Lmo4* enhances CD8^+^ T cell antitumor immunity. **a** Experimental design investigating the effect of Lmo4 overexpression on the antitumor immune response of CD8^+^ T cells. **b**, **c** Tumor size (**b**) and survival curve (**c**) of B16_KVP_ tumor-bearing wild-type mice after transfer of either 3.5 × 10^5^ pmel-1 *Thy1.1*^+^ or *Lmo4-Thy1.1*^+^ CD8^+^ T cells into wild-type mice treated with gp100-vv and IL-2. **d** Representative flow cytometry plot depicting the gating strategy to assess the frequency of human T_SCM_ cells, defined as CD45RA^+^CD45R0^-^CD62L^+^CCR7^+^CD95^+^TCF1^+^ cells. **e** Percentage of T_SCM_ cells in *LMO4*-*Thy1.1* and *Thy1.1* transduced CD8^+^ T cells after activation by TransAct and subsequent culture in IL-7 and IL-21 for 7 days (*n* = 5). **f** Experimental design investigating the effect of LMO4 overexpression on the antitumor immune response of human CD19-CAR-modified CD8^+^ T cells (*n* = 5 to 7 mice/group). **g** Percentage of human CD8^+^ T cells in the peripheral blood of NXG mice bearing NALM6-GL leukemia 7 days after adoptive transfer of *LMO4*-*Thy1.1* or *Thy1.1* CD19-CAR CD8^+^ T cells in conjunction with recombinant human IL-15. **h** In vivo bioluminescent imaging and **i** survival of NALM6-GL-bearing NXG mice treated as in (**g**). (*P < 0.05. **g**, unpaired one-tailed Student’s *t*-test, **i**, *P* = 0.0597 log-rank (Mantel–Cox) test). (**a**, **f**) was created with BioRender.com

Given the ability of LMO4 overexpression to enhance stem-like T-cell formation, we sought to determine whether LMO4 overexpression could potentiate the therapeutic efficacy of pmel-1 T cells in a model of neoantigen-targeted adoptive immunotherapy. We adoptively transferred pmel-1 T cells overexpressing *Lmo4-Thy1.1* or *Thy1.1* control into wild-type mice bearing B16 melanomas carrying the mutated gp100 epitope (KVP).^30^ To enhance the expansion and function of transferred T cells, we co-delivered a gp100-vv vaccine intravenously and administered high doses of IL-2 intraperitoneally (Fig. 3a). We found that pmel-1 T cells overexpressing LMO4 were much more effective at controlling tumor growth than control cells (Fig. 3b). This robust antitumor effect led to curative responses in eight out of nine mice. In contrast, *Thy1.1* control cells cured only a small fraction of the animals (Fig. 3c). Next, we extended our investigation by examining the effects of LMO4 overexpression in an additional challenging solid tumor model, which relies on the targeting of LLC1-OVA lung carcinomas by OVA-specific OT-1 T cells (Supplementary Fig. 4a). Once more, we observed enhanced tumor control in mice receiving *Lmo4-Thy1.1* T cells (Supplementary Fig. 4b). However, under this specific experimental setting, the survival benefit did not achieve statistical significance at *P* < 0.05 (Supplementary Fig. 4c). To gauge the relevance of this approach in humans, we sought first to determine whether ectopic expression of LMO4 could be employed to enhance the generation of human stem-like T cells. We activated naive CD8^+^ T cells in the presence of IL-21 and IL-7, a combination of cytokines commonly used in the production of clinical-grade CAR stem-like T cells,^5^ and subsequently transduced them with either *LMO4*-*Thy1.1* or *Thy1.1* control. Strikingly, LMO4 overexpression significantly increased the generation of stem cell memory T cells (T_SCM_) across several donors (Fig. 3d, e). We next evaluated whether the LMO4 technology could be adopted to potentiate the efficacy of human CAR T cells in a humanized model of acute lymphoblasts leukemia (NALM6-GL)^5^ (Fig. 3f). Overexpression of LMO4 promoted the expansion of CD8^+^ T cells in the circulation of NALM6-bearing NXG mice seven days after adoptive transfer (Fig. 3g). This enhanced engraftment was associated with improved tumor control and extended survival of the animals (Fig. 3h, i and Supplementary Fig. 4d). These findings highlight the potential of LMO4 overexpression to enhance T-cell therapy, demonstrating efficacy not only in syngeneic tumor models but also in human xenograft settings.

### LMO4 overexpression promotes transcriptional programs regulating stemness

**Fig. 4:**
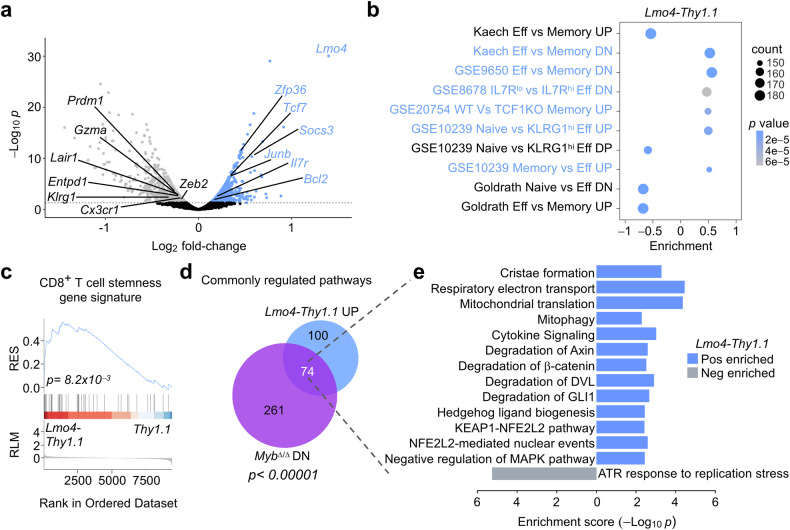
Ectopic expression of LMO4 enhances transcriptional programs that regulate stemness. **a** Volcano plot displaying changes in gene expression between pmel-1 *Thy1.1*^+^ and *Lmo4-Thy1.1*^+^ CD8^+^ T cells. Gene expression was evaluated by RNA-seq of pmel-1 CD62L^-^KLRG1^-^ T cells collected 5 d after transfer of 10^5^ pmel-1 *Thy1.1*^+^ and *Lmo4-Thy1.1*^+^ CD8^+^ T cells into wild-type mice infected with gp100-vv (*n* = 5 mice/group). **b** Bubble plot illustrating significantly enriched pathways related to CD8^+^ T cell memory and effector differentiation. **c** GSEA revealing positive enrichment of genes universally characteristic of stem-like T cells,^33^ in pmel-1 *Lmo4-Thy1.1*^+^ CD8^+^ T cells. **d** Venn diagram illustrating the overlap between pathways significantly upregulated in *Lmo4-Thy1.1*^+^ and downregulated in *Myb*^Δ/Δ^ pmel-1 CD8^+^ T cells, generated under identical experimental conditions, described in (**a**). **e** Bidirectional bar plot displaying the enrichment scores of selected enriched pathways in *Lmo4-Thy1.1* and *Myb*^Δ/Δ^ pmel-1 CD8^+^ T cells. *P*-value was calculated with **c** Kolmogorov–Smirnov test, **d** Fisher’s Exact Test

To uncover the underlying molecular mechanisms by which LMO4 influences CD8^+^ T-cell memory differentiation and antitumor immunity, we conducted RNA-seq analysis on *Lmo4*-overexpressing and control *Thy1.1* pmel-1 T cells isolated five days after transfer into mice infected with gp100-vv. To avoid potential misinterpretation arising from transcriptional variances due to the skewed composition of T-cell subsets in control and *Lmo4*-overexpressing T cells, we sorted a homogenous population of CD62L^−^KLRG1^−^cells with purities exceeding 99%. We found 889 upregulated and 776 downregulated genes (*P* < 0.05) between LMO4-overexpressing T cells and Thy1.1 (Supplementary Table 3). Even after phenotypic subset normalization *Lmo4-Thy1.1* transduced cells expressed higher levels of genes known to regulate memory T-cell differentiation. In particular, *Tcf7*, a key regulator of T-cell stemness and longevity,^10^ was highly expressed in *Lmo4*-overexpressing T cells compared to controls. Similarly, *Socs3*, a suppressor of cytokine signaling that has been shown to prevent terminal differentiation by shielding developing memory CD8^+^ T cells from inflammatory cytokines,^14^ was elevated in *Lmo4*-overexpressing T cells. *Lmo4*-overexpressing T cells also displayed increased mRNA levels of *Zfp36*, an RNA-binding protein that was recently shown to dampen CD8^+^ T-cell effector responses, facilitating the formation of memory precursors.^31^ The AP-1 transcription factor subunit *Junb* was upregulated in CD8^+^ T cells expressing heightened levels of LMO4. AP-1 has been mostly linked to effector differentiation,^12^ but recent evidence has demonstrated the role of its subunits for T-cell survival and stemness.^32^ Conversely, control cells displayed heightened expression of effector transcripts, such as *Prdm1*, *Zeb2*, and *Klrg1* (Fig. 4a). Accordingly, Gene Set Enrichment Analysis (GSEA) revealed that *Lmo4*-overexpressing cells exhibited enrichment for gene sets linked to naive and memory CD8^+^ T cells or IL-7R^hi^ memory precursors (Fig. 4b). By contrast, gene signatures associated with effectors as well as TCF1-deficient memory T cells, were negatively enriched (Fig. 4b). Remarkably, ectopic expression of LMO4 upregulated genes that are universally characteristic of stem-like T cells across various diseases, including acute and chronic viral infections as well as tumors^33^ (Fig. 4c, Supplementary Table 4).

We and others recently identified c-Myb as a key transcription factor promoting CD8^+^ T cell stemness.^9,34^ To assess the degree to which LMO4 and c-Myb regulate overlapping transcriptional programs associated with stemness, we conducted pathway analysis on *Lmo4*-overexpressing cells. We then compared these results with those obtained from pmel-1 CD8^+^ *Myb*^Δ/Δ^ T cells, which were previously generated under identical experimental conditions.^9^ Out of the 174 pathways significantly enriched in *Lmo4-Thy1.1*, 74 (42.5%) displayed opposite enrichment patterns in *Myb*-deficient cells (*P* < 0.00001) (Fig. 4d, Supplementary Table 5). Strikingly, many of the upregulated pathways in *Lmo4*-overexpressing cells were associated with stemness, including the regulation of WNT,^10^ Hedgehog,^35^ and NRF2 (also known as NFE2L2) signaling^36^ (Fig. 4e). In addition, pathways related to mitochondrial metabolism, vital for the fitness of stem-like memory cells,^37^ were also prominently upregulated (Fig. 4e). Inhibition of the MAPK pathway has similarly been associated with the development of stem-like T cells.^38^ Notably, ectopic expression of LMO4 led to the upregulation of gene sets associated with the negative regulation of this signaling pathway (Fig. 4e). Among the negatively enriched pathways in *Lmo4-Thy1.1* cells, only one was oppositely regulated in *Myb*^Δ/Δ^ T cells. This pathway relates to the ATR response to replication stress^39^ (Fig. 4e), suggesting that LMO4 overexpression promoted cellular quiescence during the peak of the immune response, which likely facilitates the commitment of cells to enter the memory pool.^40^ Taken together, these findings highlight the capacity of LMO4 to orchestrate several transcriptional programs associated with stemness and cellular fitness.

### LMO4 overexpression boosts IL-21-mediated STAT3 signaling to orchestrate a memory-promoting gene regulatory network

**Fig. 5:**
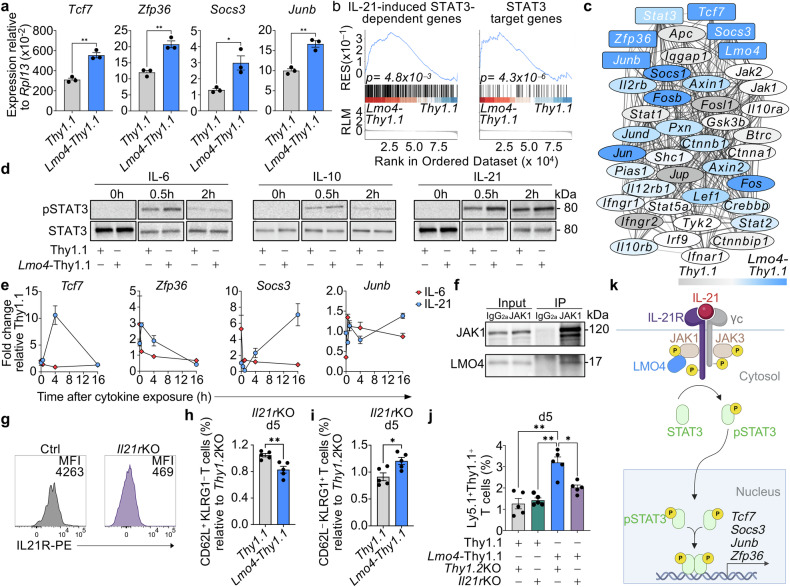
*Lmo4* overexpression enhances STAT3 phosphorylation and STAT3 target gene expression. **a** qPCR of *Tcf7*, *Zfp36*, *Socs3*, and *Junb* mRNA in pmel-1 *Thy1.1*^+^ and *Lmo4-Thy1.1*^+^ CD8^+^ T cells 5 d after transfer of 10^5^ pmel-1 CD8^+^ T cells transduced with either *Thy1.1*^+^ or *Lmo4-Thy1.1*^+^ into wild-type mice infected with gp100-vv. **b** GSEA showing positive enrichment of genes upregulated in response to IL-21 in CD4^+^ T cells^42^ (left panel) and genes displaying one or more STAT3 binding motifs (GSEA C3:STAT3_02) (right panel) in pmel-1 *Lmo4-Thy1.1*^+^ CD8^+^ T cells harvested as in (**a**). **c** Gene regulatory network that interfaces with *Stat3* in pmel-1 *Thy1.1*^+^ vs. *Lmo4-Thy1.1*^+^ CD8^+^ T cells. Genes upregulated in *Thy1.1* cells are shown in gray color gradient, whereas those overexpressed in *Lmo4-Thy1.1* cells are marked in blue gradient. Network was created using Cytoscape software and STRING database. Line thickness of network edges indicates the strength of data support for each interaction based on the information available in the STRING database. **d** Immunoblot of pSTAT3 and STAT3 (control) in *Thy1.1* and *Lmo4-Thy1.1* overexpressing T cells at 0, 0.5, and 2 h after adding IL-6 (left panel), IL-10 (middle panel) or IL-21 (right panel) to the cell culture. **e** q-PCR of *Tcf7* (left panel), *Zfp36* (middle left panel), *Socs3* (middle right panel), and *Junb* (right panel) mRNA in *Lmo4-Thy1.1* relative to *Thy1.1* overexpressing T cells after culture with IL-6 or IL-21 for the indicated time. **f** Immunoblot of *Lmo4*-*Thy1.1* CD8^+^ T cell lysates and anti-JAK1 immunoprecipitates blotted with JAK1 and LMO4 specific antibodies. Anti-IgG2a immunoprecipitates were used as controls. **g** Flow cytometry histograms showing IL21R expression in pmel-1 CD8^+^ T cells to assess the knockout efficiency compared to controls. **h**, **i** Percentages of CD62L^+^KLRG1^-^ (**h**) and CD62L^-^KLRG1^+^ (**i**) splenic pmel-1 CD8^+^ T cells 5 d after transfer of either 10^5^ *Thy1.1*^+^*Il21r*KO or *Lmo4-Thy1.1*^+^*Il21r*KO pmel-1 Ly5.1^+^ CD8^+^ T cells into wild-type mice infected with gp100-vv (*n* = 5 mice/group). Results are relative to *Thy1.1*^+^*Thy1.2*KO and *Lmo4-Thy1.1*^+^*Thy1.2*KO control cells, respectively. **j** Percentages of pmel-1 CD8^+^ T cells in the lymph nodes 5 d after transfer as in (**h**, **i**). **k** Cartoon depicting the potential interaction of LMO4 with the IL-21-STAT3 signaling pathway. LMO4 binds to JAK1, promoting STAT3 phosphorylation and the expression of target genes such as *Tcf7*, *Socs3*, *Junb*, and *Zfp36* to boost memory responses (*P < 0.05; ** *P* < 0.001, **h**, **i**, unpaired two-tailed Student’s *t*-test; **j**, ANOVA test, **b** Kolmogorov–Smirnov test). (**k**) was created with BioRender.com

Among the pathways influenced by both LMO4 and c-Myb manipulation, we also observed a highly significant enrichment of the Reactome pathway R-MMU-1280215, which relates to cytokine signaling (*P* < 0.001) and includes several molecules involved in STAT signaling (Fig. 4e, Supplementary Table 5). Interestingly, *Tcf7*, *Zfp36*, *Socs3*, and *Junb* are all known targets of STAT3,^41^ implying a potential involvement of the STAT3 pathway in the actions of LMO4. To substantiate this hypothesis, we first validated our RNA-seq results by q-PCR, confirming the upregulation of these pro-memory factors in *Lmo4*-overexpressing cells (Fig. 5a). We then conducted GSEA utilizing STAT3 datasets. We found that *Lmo4-Thy1.1* T cells exhibited an enrichment of STAT3-dependent genes that are upregulated in CD4^+^ T cells in response to IL-21,^42^ whereas *Myb*-deficient cells were negatively enriched (Fig. 5b, Supplementary Fig. 5, and Supplementary Table 6). Moreover, LMO4-overexpressing T cells were enriched with genes displaying one or more STAT3 binding motifs (GSEA C3:STAT3_02) (Fig. 5b, Supplementary Table 7). Notably, *Lmo4, Tcf7*, *Junb, Socs3*, and *Zfp36* were interconnected in a complex gene regulatory network that interfaces with *Stat3* (Fig. 5c). Taken together, these analyses further corroborate an enhancement of STAT3 signaling in LMO4-overexpressing CD8^+^ T cells.

LMO4 has been shown to enhance the activity of the STAT3 pathway in response to diverse STAT3-signaling molecules.^43,44^ IL-6,^45^ IL-10,^14^ and IL-21^21,24^ are well-established STAT3-signaling cytokines that promote CD8^+^ T-cell memory. To determine which of these cytokines had a predominant effect in our system, we stimulated *Lmo4-Thy1.1* or *Thy1.1* T cells with each of these cytokines and measured the level of phosphorylated STAT3 (pSTAT3) at different time points. IL-6 and IL-21 induced a more pronounced STAT3 phosphorylation in LMO4-overexpressing T cells compared to controls (Fig. 5d, and Supplementary Fig. 6). IL-10, on the other hand, induced pSTAT3 without noticeable differences between the two experimental groups (Fig. 5d, Supplementary Fig. 6), indicating that this cytokine does not play a major role in the pro-memory function of LMO4. To dissect the potential involvement of IL-6 and IL-21, we evaluated the impact of these two cytokines on the expression of *Tcf7*, *Zfp36, Socs3*, and *Junb* in LMO4-overexpressing T cells and controls. Strikingly, IL-21, but not IL-6, induced the expression of all these molecules, though with distinct kinetics (Fig. 5e). IL-6 was only able to trigger a rapid but transient induction of *Junb* (Fig. 5e, right panel).

LMO4 has been previously demonstrated to augment STAT3 signaling in response to IL-6, by associating with gp130 and JAK1, thereby stabilizing the IL-6 signaling complex.^43^ Given that JAK1 is also essential for IL-21 signaling,^21^ we hypothesized that LMO4 could promote IL-21-STAT3 signaling via JAK1 binding. Immunoprecipitation of JAK1 in *Lmo4*-*Thy1.1* T cells revealed that LMO4 directly interacts with JAK1 (Fig. 5f), providing a mechanistic link between LMO4 and IL-21-STAT3 signaling in primary CD8^+^ T cells.

To substantiate the involvement of IL-21 in LMO4-mediated stemness, we assessed the impact of deleting *Il21r* on the generation of CD62L^+^ memory T cell precursors in response to gp100-vv. Using CRISPR/Cas9, we profoundly downregulated IL21R expression on the surface of CD8^+^ T cells (Fig. 5g). Five days after pmel-1 T cell transfer, we found that knocking out *Il21r* reduced the frequencies of splenic CD62L^+^KLRG1^−^T cells in *Lmo4-Thy1.1* T cells relative to *Thy1.2* knockout controls (Fig. 5h). Conversely, the percentage of CD62L^−^KLRG1^+^terminal effectors was increased upon deletion of the IL-21 receptor (Fig. 5i). Notably knocking out *Il21r* did not have a significant impact on CD8^+^ T cells transduced with *Thy1.1* (Fig. 5h, i). The negative impact of *Il21r* deletion on the generation of CD62L^+^ memory T cell precursors abrogated the typical accumulation of *Lmo4-Thy1.1* T cells in lymph nodes, whereas it had no effects on *Thy.1.1* controls (Fig. 5j). While the involvement of other STAT3-signaling cytokines cannot be excluded, our findings highlight the pivotal role of IL-21 signaling in the setting of LMO4 overexpression. This supports a model by which LMO4 enhances T-cell stemness by boosting IL21-STAT3 signaling and its downstream pro-memory factors (Fig. 5k).

### LMO4 overexpression increases CD8^+^ T-cell stemness and antitumor responses in a STAT3-dependent manner

**Fig. 6:**
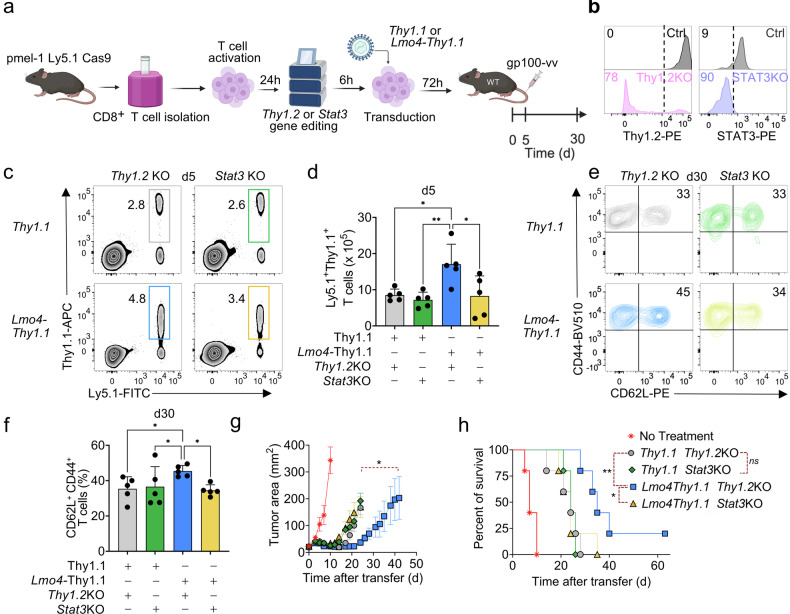
STAT3 is essential for LMO4-induced CD8^+^ T cell stemness and enhanced antitumor responses. **a** Experimental design to assess the impact of *Stat3* versus *Thy1.2* (control) deletion on *Lmo4-*induced stem-like T cell formation. **b** Flow cytometry histograms showing Thy1.2 (left panel) and STAT3 (right panel) to assess knockout efficiency compared to controls in pmel-1 CD8^+^ T cells. **c**, **d** Flow cytometry analysis (**c**) and percentages (**d**) of splenic pmel-1 CD8^+^ T cells 5 d after transfer of either 10^5^ pmel-1 Ly5.1^+^ *Thy1.1*^+^*Thy1.2*KO, *Thy1.1*^+^*Stat3*KO, *Lmo4-Thy1.1*^+^*Thy1.2*KO or *Lmo4-Thy1.1*^+^*Stat3*KO CD8^+^ T cells into wild-type mice infected with gp100-vv (five mice per group). **e**, **f** Flow cytometry analysis (**e**) and percentages (**f**) of CD62L^+^CD44^+^ splenic pmel-1 T cells 30 d after transfer as in (**c**, **d**). **g**, **h** Tumor size (**g**) and survival curve (**h**) of B16_KVP_ tumor-bearing wild-type mice after transfer of either 3.5 × 10^5^ pmel-1 *Thy1.1*^+^*Thy1.2*KO, *Thy1.1*^+^*Stat3*KO, *Lmo4-Thy1.1*^+^*Thy1.2*KO or *Lmo4-Thy1.1*^+^*Stat3*KO CD8^+^ T cells into wild-type mice treated with gp100-vv and IL-2. *P < 0.05, **P < 0.01 (**d**, **f**, unpaired two-tailed Student’s *t*-test, **g**, Wilcoxon rank-sum test; **h**, log-rank (Mantel–Cox) test). (**a**) was created with BioRender.com

To investigate if STAT3 signaling is a crucial mediator of the memory phenotype induced by LMO4, we next evaluated the effects of knocking out *Stat3* on the expansion and memory differentiation of pmel-1 *Lmo4-Thy1.1* T cells (Fig. 6a). We employed CRISPR/Cas9 technology to delete *Stat3* or *Thy1.2* control with knock-out efficiencies of 90% and 78%, respectively (Fig. 6b). We then adoptively transferred pmel-1 *Lmo4-Thy1.1* and pmel-1 *Thy1.1* T cells either deficient for *Stat3* or *Thy1.2* into wild-type mice infected with gp100-vv, and measured CD8^+^ T-cell expansion, and memory formation over time. We found that the deletion of *Stat3* abrogated the beneficial effects of LMO4 overexpression on T-cell expansion (Fig. 6c, d). Of note, we did not observe a major impact of *Stat3* removal on Thy1.1 control cells, highlighting the specific role of STAT3 in the context of LMO4 overexpression (Fig. 6c, d). Similarly, *Stat3* deficiency impaired the augmented formation of stem-like T cells observed in the setting of LMO4 overexpression (Fig. 6e, f). These findings demonstrate the central role of the STAT3 signaling pathway in LMO4-induced regulation of CD8^+^ T-cell responses and their development into stem-like cells.

To further determine if STAT3 signaling is also required for the antitumor effects of LMO4-overexpressing T cells, we adoptively transferred pmel-1Cas9 *Lmo4*-*Thy1.1* and *Thy1.1* T cells nucleofected with gRNAs specific for *Stat3* or *Thy1.2* into mice bearing subcutaneous B16_KVP_ melanomas in conjunction with gp100-vv and IL-2. Consistent with our results in the infection model, we found that the enhanced antitumor responses promoted by the heightened levels of LMO4 also depend on STAT3 (Fig. 6g, h). The reduced antitumor potency of LMO4-overexpressing T cells observed in this experiment might be due to incomplete recovery of cellular fitness following electroporation.^46^ Altogether, these findings underscore the essential role of STAT3 in mediating the effects of LMO4 in enhancing CD8^+^ T-cell stemness and antitumor immunity.

## Discussion

Strategies to enhance the stemness and persistence of antitumor T cells are being actively pursued given the central role of stem-like cells in eliciting productive antitumor responses.^3,8^ Several pathways and relevant players influencing the formation of these cells have been characterized in preclinical tumor models offering exploitable targets for potentiating T-cell therapy.^9–14^ By using CRISPRa gain-of-function screens, we aimed to broaden actionable targets to augment CD8^+^ T-cell stemness beyond normal physiology. This analysis led us to identify LMO4 as a potent modulator of CD8^+^ T-cell stemness and memory formation when ectopically expressed. LMO4 boosted CD8^+^ T-cell responses while preserving a substantial pool of stem-like memory T cells even following repetitive antigenic exposure. These results vividly illustrate how synthetic biology can be utilized to confer desired functions by borrowing molecules that regulate a specific function (i.e., cell fate control) in one cell type and introducing them into another type, where such regulation is not normally exerted by the particular molecule under physiological conditions.

Mechanistically, we found that LMO4, rather than directly modulating gene transcription, boosted STAT3 signaling in response to IL-21, activating a complex gene regulatory network that comprised the upregulation of several STAT3 target molecules known to shape CD8^+^ T-cell differentiation and stemness. Interestingly, many of the upregulated genes in this network, including *JUNB*, *JUN*, *FOS*, *FOSB*, and *ZFP36*, were recently found to be enriched in a subset of highly functional T cells isolated from human head and neck squamous cell carcinoma,^47^ underscoring its potential relevant role in enhancing the antitumor effectiveness of LMO4*-*overexpressing T cells.

Our findings have substantial implications for advancing the development of more potent T cell-based immunotherapies. We demonstrated that LMO4 overexpression enhances antitumor T cell responses across various models, encompassing solid cancers and hematologic malignancies, in both syngeneic and xenograft settings. While we found substantial overlap between LMO4 and MYB, the overall number of pathways regulated by LMO4 was much smaller, highlighting that LMO4 offers an option to control T cell differentiation in a more targeted fashion. The compact genetic footprint of *Lmo4*, spanning less than 500 nucleotides (nt), offers the additional advantage of easy integration in CAR and TCR vectors currently employed in the clinic compared to *Myb* (~2000 nt). It is worth noting that the relatively milder effect observed in the CD19-CAR T and OT-1 models could be a result of the lack of sources of IL-21 in vivo (e.g., activated CD4^+^ T cells), which are instead triggered by the vaccinia virus vaccine in the pmel-1 model. Indeed, NXG mice lack functional human immune cells, while the DC-based vaccine strategy used in the OT-1 model exclusively targets the stimulation of OT-1 T cells by the SIINFEKL peptide.

The involvement of LMO1 and LMO2 in T-ALL^19^ might raise some concerns regarding potential T-cell transformation due to LMO4 overexpression. Although the enforced expression of LMO4 induced massive CD8^+^ T-cell expansion, we did not observe unrestrained expansion or cell transformation in all our studies despite the continuous presence of the cognate antigen on melanocytes. These observations are in line with the evidence indicating that mature T cells are difficult to transform, even when well-known oncogenes are overexpressed.^48^ While we did not observe any overt oncogenicity (Supplementary Fig. 7), the translation of this technology in humans warrants caution. Suicide genes can be integrated to eliminate transferred cells in case of transformation, or inducible promoters could be utilized to enhance the safety of this approach.^49^

Ultimately, the “synthetic” expression of LMO4 could be a valuable addition to the immunotherapeutic arsenal for addressing solid and liquid tumors, especially when combined with therapies designed to trigger STAT3-signaling.

## Methods

### Mice

We obtained C57BL/6NCr, B6-Ly5.1/Cr and OT-1 mice from Charles River Frederick, Sulzfeld or Calco; pmel-1 (B6. Cg-Thy1^a^/Cy Tg (TcraTcrb)8Rest/J) mice were bought from the Jackson Laboratory; Cre-ER^T2^ (B6-Gt(ROSA)26Sor^tm9(cre/Esr1)Arte^) mice were from Taconic. *Lmo4*^fl/fl^ mice^50^ were obtained from Bogi Andersen and were backcrossed with C57BL/6NCr mice for more than 30 generations. Pmel-1 mice were crossed with *Lmo4*^fl/fl^ mice for the formation of pmel-1 *Lmo4*^fl/fl^ mice and were further crossed with Cre-ER^T2^ mice for the generation of pmel-1 Cre-ER^T2^ *Lmo4*^fl/fl^ mice. Immunodeficient NCG and NXG mice ages 6–8 weeks were obtained from Janvier Labs. All mouse experiments were performed with the approval of the National Cancer Institute, the National Heart, Lung, and Blood Institute Animal Care and Use Committees, the Institutional Animal Care and Use Committee programme (IACUC n° 1178 and 1208) and the Government of Lower Franconia. Experiments performed at NIH were done according to NIH guidelines for research using mice.

### Cell lines

Platinum-E cells were from Cell Biolabs, 293GP cells from ATCC, both being authenticated and validated mycoplasma free. B16 melanoma expressing human gp100 (B16_KVP_)^30^ were obtained from K.-I. Hanada (National Cancer Institute, Bethesda), Lewis Lung Carcinoma (LLC1) cells from ATCC, NALM6-GL cells were obtained from T. Fry (National Cancer Institute, Bethesda). All cell lines were validated by PCR-assay to be mycoplasma free.

### Antibodies, flow cytometry, and cell sorting

Anti-Ly5.1 (A20), Anti-Ly5.2 (104), anti-Thy1.1 (OX-7), anti-CD62L (MEL-14), anti-IFNγ (XMG1.2), anti-TNFα (MP6-XT2), anti-CD8 (HIT8a), anti-THY1.1 (HIS51), anti-CCR7 (2-L1-A), anti-CD45RA (5H9), anti-CD45R0 (UCHL1) were bought from BD Biosciences; anti-CD8α (53-6.7), anti-Thy1.2 (30-H12), anti-KLRG-1 (2F1), anti-CD44 (IM7), anti-IL-2 (JE56-5H4), anti-STAT3 (4G4B45), anti-IL-21R (4A9), anti-CD95 (DX2), anti-CD62L (DREG-56) were from Biolegend; anti-TCF1 (C63D9) was from Cell Signaling; LIVE/DEAD™ Fixable Far Red Dead Cell Stain from Invitrogen. CellTrace™ Violet Cell Proliferation Kit (Invitrogen) was employed to label cells for tracking multiple generations via dye dilution using flow cytometry. For intracellular staining of STAT3, cells were fixed and permeabilized (eBioscience, 00-5524). Leukocyte Activation Cocktail containing phorbol myristate acetate and ionomycin (BD Biosciences) was employed to activate CD8^+^ T cells for intercellular cytokine staining. A Fixation/Permeabilization Solution Kit (BD Biosciences) was used to fix and permeabilize the cells. Flow Cytometry Acquisition was done on an LSR II or BDFortessa (BD Biosciences) flow cytometer. The raw data were analyzed by using FlowJo software (TreeStar). For naive CD8^+^ T-cell enrichment, the Naive CD8^+^ T-cell isolation kit from Stem Cell Technology was used. All other T-cell isolations were done on FACSAria (BD Biosciences).

### Quantitative PCR

The Isolation of RNA was performed using the RNeasy Mini Kit (Qiagen). Complementary DNA (Applied Biosystems) was obtained by PCR. qPCR was done using primers from Applied Biosystems and a Quant Studio 3 (Applied Biosystems) using PowerUp SYBR Green Master Mix (Applied Biosystems). Findings are shown relative to *Actb* or *Rpl13* expression.

List of primers:

*ActbF*, *ActbR*: Primers from Applied Biosystems were used.

*Rpl13*F: CGAGGCATGCTGCCCCACAA

*Rpl13*R: AGCAGGGACCACCATCCGCT

*Tcf*7F: AGCTTTCTCCACTCTACGAACA

*Tcf*7R: AATCCAGAGAGATCGGGGGTC

*Socs3*F: ATGGTCACCCACAGCAAGTTT

*Socs3*R: TCCAGTAGAATCCGCTCTCCT

*Junb*F: TCACGACGACTCTTACGCAG

*Junb*R: CCTTGAGACCCCGATAGGGA

*Zfp36*F: CCACCTCCTCTCGATACAAGA

*Zfp36*R: GCTTGGCGAAGTTCACCCA

### Immunoblot and immunoprecipitation assays

Proteins were segregated by 4–12% SDS–PAGE, then standard immunoblot analysis was performed with anti-LMO4 (Cell Signaling Technology, clone D6V4Z), anti-ACTB (Cell Signaling, clone 8H10D10), anti-STAT3 (Cell Signaling Technology, clone 124H6), anti-pSTAT3(Tyr705) (Cell Signaling Technology, clone M9C6), horseradish peroxidase-conjugated goat anti-mouse IgG (sc-2031; Santa Cruz Biotechnology) and horseradish peroxidase-conjugated anti-rabbit IgG (sc-2030; Santa Cruz Biotechnology). Immunoprecipitation of JAK1 was performed using anti-JAK1 antibody (Cell Signaling Technology, clone D1T6W). IgG2a was used as negative control (Cell Signaling Technology, clone E5Y6Q). In brief, the cells were washed twice with PBS and lysed in ice-cold lysis buffer (50 mM Tris–Cl (pH 7.5), 150 mM NaCl, and 1% NP-40) with protease and phosphatase inhibitors (Cell Signaling). The cell lysates containing 1 to 3 mg of protein were pre-cleared with 30 μl protein A/G-Sepharose beads (Thermo Scientific) for 1 h at 4 °C and thereafter incubated with the appropriate antibody overnight at 4 °C, following another incubation with 30 μl of protein A/G-Sepharose beads for additional 2 h at 4 °C. The immunoprecipitates were washed three times with washing buffer (20 mM Tris–Cl (pH 7.5), 150 mM NaCl, and 0.1% NP-40), separated by SDS–polyacrylamide gel electrophoresis (SDS–PAGE) after being boiled in Laemmli buffer, and transferred to a 0.2 µm nitrocellulose membrane. The membrane was blocked with PBS containing 0.1% Tween-20 and 1% bovine serum albumin before it was incubated with the appropriate primary and secondary antibodies. The bound antibodies were visualized using Pierce ECL WB Substrate (Thermo Fisher).

### Retroviral vector generation and virus synthesis

*Lmo4* was cloned into the MSGV-1-*Thy1.1* vector as formerly reported.^13^ Platinum-E (mouse setting) or 293GP (human setting) cell lines were employed for gamma-retroviral vector generation by transfection with DNA plasmids using Lipofectamine 2000 (Invitrogen) and collecting the virus 40 h after transfection.

### Mouse CD8^+^ T-cell in vitro stimulation and transduction

Naive CD8^+^ T cells were stimulated in 24-well tissue culture plates coated with anti-CD3ε (2 µg ml^−1^; 145-2C11; BD Biosciences) and soluble anti-CD28 (1 µg ml^−1^; 37.51; BD Biosciences) in culture medium including recombinant human (rh) IL-2 (10 ng ml^−1^; Prometheus Laboratories). The virus was spun at 2000 × *g* for 2 h at 32 °C on non-tissue culture plates coated with retronectin (Takara). CD8^+^ T cells stimulated for 24 h were spun on these plates after aspiration of the viral supernatant. The transduction efficiency was measured after 48 h. For experiments evaluating STAT3 phosphorylation and target gene expression, CD8^+^ T cells were washed from rhIL-2 supplemented medium 3 days after stimulation and incubated o/n at 37 °C. CD8^+^ T cells were then exposed for 30 min to 20 ng ml^−1^ of mouse IL-6, IL-10, and IL-21 (all Miltenyi Biotec). LLC1 cells were transduced with virus-containing supernatant and selected for ∆LNGFr expression by magnetic beads (Dynabeads M-450, Dynal) coated with the LNGFr-specific mAb 20.4 (ATCC).

### Human CD8^+^ T cell in vitro stimulation and transduction

PBMCs (NIH, US and University Hospital Regensburg, Germany) were enriched for naive CD8^+^ T cells using naive CD8^+^ T cell isolation kit (Stem Cell Technologies) before freezing. To generate T_SCM_-enriched cells, naive CD8^+^ T cells were thawed and activated with GMP-grade TransAct (Miltenyi Biotec) in GMP-grade TexMACS medium (Miltenyi Biotec), 0.5% human AB serum (ZKT Tübingen, FSM/TV 276), supplemented with 1% PenStrep (Gibco) in the presence of 5 ng/mL IL-7 and 30 ng/mL IL-21 (both Miltenyi Biotec). Cells were transduced on days 2 and 3 and expanded for 5 more days in media containing IL-7 and IL-21. To generate T_SCM_-enriched cells modified with CD19-CAR, naive CD8^+^ T cells were thawed and stimulated using anti-CD3/CD28 beads (Dynabeads Human T-Expander CD3/CD28; Thermo Fisher Scientific) at a 1:1 bead-to-cell ratio in AIM-V (Gibco), supplemented with 5% human AB serum 2 mM GMAX (Gibco), 5 ng/mL IL-7 and 30 ng/mL IL-21. Transduction with the PG13 expressing CD19-CAR (FMC63-28-ζ) retrovirus was performed on days 2 and 3, followed by expansion in media containing IL-7 and IL-21 for an additional 5 days after the removal of activating beads on day 4.

### Generation of dendritic cell vaccine

Dendritic cells (DCs) were generated from bone marrow (BM) of femurs and tibias. BM was flushed out with RPMI, and a single-cell suspension was prepared by passing the BM solution through a 19-gauge needle. BM cells re-suspended at 5 × 10^5^ cells/ml in 6-well plates were cultured in IMDM 10% FBS, GM-CSF and IL-4 (PeproTech), both at 20 ng/ml. At day 2, cells were split 1:2 by adding fresh medium and cytokines. At day 5, we removed and discarded 2 ml of supernatant and added 2 ml of fresh medium with GM-CSF and IL-4 (both 40 ng/ml). At day 7, DCs were activated with LPS (1 mg/ml) for 16 h. The day after DCs were collected and pulsed with 5 μg/ml of the OVA-derived peptide SIINFEKL, for 1 h at 37 °C. Peptide-pulsed DCs were then washed and resuspended at 5 × 10^6^ cells/ml. We injected 100 μl of PBS containing peptide-pulsed DCs/mice s.c. the day after ACT.

### Tamoxifen injections, adoptive cell transfer, virus infection, and tumor inoculation

Cre-ER^T2^ activity was prompted by intraperitoneal injection of 2 mg tamoxifen (Sigma-Aldrich) dissolved in corn oil (Sigma-Aldrich) on 4 successive days. The adoptive transfer of pmel-1 CD8^+^ T cells (1.5 to 10 × 10^5^ cells) into 6- to 8-week-old C57BL/6 mice was performed together with the vaccination of 2 × 10^7^ PFU recombinant vaccinia virus expressing human gp100 (gp100-vv). For recall response,35 d after primary infection with gp100-vv pmel-1 memory T cells were sorted, normalized for cell numbers, and transferred into secondary C57BL/6 mice infected with 10^8^ PFU recombinant adenovirus type 2 expressing human gp100. To prevent potential rejection in conditional knockout experiments investigating the late contraction phase of the immune response and in secondary treatment experiments, we used recipient mice carrying the *Lmo4*^fl^ allele. In these experiments, as wild-type controls, we used pmel-1 CD8^+^ T cells isolated from tamoxifen-injected littermates carrying the *Lmo4*^fl^ allele but not Cre-ER^T2^.

#### Murine syngeneic melanoma model

2 × 10^5^ B16_KVP_ cells were inoculated subcutaneously into 6- to 8-week-old C57BL/6 mice. After 10 days mice were adoptively transferred with 3.5 × 10^5^ pmel-1 CD8^+^ T cells and vaccinated intravenously with 2 × 10^7^ PFU gp100-vv. Recombinant human IL-2 (2.4e5 IU per dose) was injected intraperitoneally twice a day for a total of 6 doses.

#### Murine syngeneic lung carcinoma model

Mice were injected subcutaneously with LLC1 cells (5 × 10^5^) expressing ovalbumin (LLC1-OVA). Seven days later, mice were injected intraperitoneally with a single dose of cyclophosphamide (CTX 3 mg/mouse). After 24 h, mice were randomized and adoptively transferred with OT-1 T cells transduced with *Thy1.1* or *Lmo4-Thy1.1* (10^6^ cells). The day after, mice were subcutaneously injected with dendritic cells (5 × 10^5^) loaded with the SIINFEKL peptide. Tumor volume was measured every 2 days.

#### Xenograft acute lymphoblastic leukemia model

NXG host mice received intravenous injections of NALM6-GL (8 × 10^5^) cells, followed by the administration of 1.25 × 10^5^ CD19-CAR^+^ CD8^+^ T cells after a 3-day interval. Recombinant human IL-15 (NCI) was administered intraperitoneally every other day at a dosage of 1 μg per mouse. Tumor burden was assessed using the IVIS Lumina III In Vivo Imaging System (PerkinElmer). After 7 days, blood samples were collected from the mice to estimate the engraftment of CD8^+^ T cells.

### Quantification of adoptively transferred cells

After processing the spleen, cells were counted by trypan blue exclusion of dead cells. The percentage of transferred CD8^+^ T cells was measured by analyzing the expression of CD8 and Thy1.1/Ly5.1 or Ly5.2 by flow cytometry. The absolute quantity of transferred cells was calculated by multiplying the total cell count by the frequency of CD8^+^Thy1.1^+^Ly5.1^+^ or CD8^+^Ly5.2^+^ cells.

### RNA-seq

Total cellular RNA was isolated from CD8^+^ T-cells using the RNeasy Mini Kit (Qiagen) according to the manufacturer’s instructions. The concentration and quality of the purified RNA was analyzed using the RNA ScreenTape Kit (Agilent). Generation of dsDNA libraries for Illumina sequencing from total cellular RNA was carried out using the TruSeq Stranded Total RNA Kit (Illumina) according to the manufacturer’s instructions. The quality of dsDNA libraries was analyzed using the High Sensitivity D1000 ScreenTape Kit (Agilent) and concentrations were assessed with the Qubit dsDNA HS Kit (Thermo Fisher Scientific). Sequencing was performed using an Illumina NextSeq550 sequencer. Sequenced reads were processed and aligned to the mm10 genome using splice-aware aligner TopHat 2.1.1. Then, raw mapped read counts were processed in R with edgeR (10.1093/bioinformatics/btp616) to generate normalized read counts and determine differentially expressed genes with log2 FC ≥ 0.3 and *P* < 0.05. Volcano plot was generated using EnhancedVolcano package (10.18129/B9.bioc.EnhancedVolcano). Volcano plot shows differentially expressed genes (*P* ≤ 0.05) in pmel-1 *Lmo4-Thy1.1*^+^ compared to *Thy1.1*^+^ CD8^+^ T cells. The position of the Lmo4 gene in the volcano plot was altered by capping its log2-FC values at a maximum threshold of 1.4, thereby constraining its positioning in the volcano plot.

### CRISPRa screen analysis

Genes that are deemed to be transcription factors or transcription regulators were filtered from the list of genes targeted in a genome-wide CRISPRa screen analysis.^17^ Filtering was done based on the Transcription Factor dataset available on Transcription Factor checkpoint 2.0 database (https://www.tfcheckpoint.org/index.php), which is a resource for Human, Mouse, and Rat Transcription Factors. The filtered gene list was used to generate a volcano plot with EnhancedVolcano package. Volcano plot shows median sgRNA log_2_-fold change (IFN-γ^hi^/^lo^ sorting bin counts) for each gene (*P* ≤ 0.05, log2 FC ≥ 0.52 and ≤−0.531).

### Cas9/RNP nucleofection

Pmel-1 CD8^+^ T cells isolated from *Cas9*^+^ C57BL/6 mice were stimulated 24 h before transfection. To prepare the crRNA-tracrRNA duplex each Alt-R crRNA and Alt-R tracrRNA (IDT) was reconstituted to 100 µM with nuclease-Free Duplex Buffer (IDT). After mixing the oligos at equimolar concentrations [0.75 µl Alt-R crRNA and 0.75 µl Alt-R tracrRNA per guide with 1.5 µl Buffer (Mirus Ingenio Electroporation)] in a sterile PCR tube, the mix was annealed by heating at 95°C for 5 min in PCR thermocycler and then slowly cooled down to room temperature. For CAS9/RNP precomplexing, resulted 3 µl crRNA-tracrRNA duplex and 1.2 µl TrueCut CAS9 Protein v2 (Thermo Fisher) were gently mixed and incubated at room temperature for 20 min. After prewarming 200 µl complete T-cell media containing rhIL-2 (10 ng ml^−1^) per well of a 96-well plate, 1 million CD8^+^ T cells were resuspended in 20 µl primary cell nucleofection solution (P4 Primary Cell 4D-Nucleofector X kit; 32 RCT; V4XP-4032, Lonza). T cells were then mixed and incubated with 4.2 µl RNP and 15.8 μl Buffer (Mirus) for a total volume of 20 µl at room temperature for 2 min in round bottom 96-well plate. Cell/RNP mix was transferred to nucleofection cuvette strips (4D-Nucleofector X kit S; Lonza) and electroporated using a 4D nucleofector (4D-Nucleofector X Unit: AAF-1002X, Lonza) and a CM137 pulse. Following nucleofection, transfected cells were transferred to 96-well plates containing 200 µl prewarmed complete T-cell media per well and incubated at 37 °C for 6 h, preceding retroviral transduction and adoptive transfer into C57BL/6 mice.

List of crRNAs used:

*Thy1.2*#1:

/AltR1/rCrCrU rUrGrG rUrGrU rUrArU rUrCrU rCrArU rGrGrG rUrUrU rUrArG rArGrC rUrArU rGrCrU/AltR2/

*Thy1.2*#2:

/AltR1/rGrArG rCrArG rGrArG rArGrC rGrArC rGrCrU rGrArG rUrUrU rUrArG rArGrC rUrArU rGrCrU /AltR2/

*Stat3*#1:

/AltR1/rCrArArCrArUrCrUrGrCrCrUrGrGrArCrCrGrUrCrGrUrUrUrUrArGrArGrCrUrArUrGrCrU/AltR2/

*Stat3*#2:

/AltR1/rArGrUrUrGrArArArUrCrArArArGrUrCrGrUrCrCrGrUrUrUrUrArGrArGrCrUrArUrGrCrU/AltR2/

*Il21r*#1:

/AltR1/rGrUrCrArArUrGrUrGrArCrGrGrArCrCrArGrUrCrGrUrUrUrUrArGrArGrCrUrArUrGrCrU/AltR2/

*Il21r*#2:

/AltR1/rCrCrCrUrCrCrArArCrUrArCrGrUrGrCrUrGrArGrGrUrUrUrUrArGrArGrCrUrArUrGrCrU/AltR2/

### Gene-set enrichment, STRING network, and pathway analyses

Mouse gene symbols were initially mapped to the orthologous human genes using the homology data from the MGI website (ftp://ftp.informatics.jax.org/pub/reports/HMD_HGNC_Accession.rpt) and were ranked by the fold changes of the gene expression as profiled by RNA-seq. Subsequently, gene-set enrichment analyses GSEA was performed using ClusterProfiler R package (10.1016/j.xinn.2021.100141). C7 immunologic signature gene set from MSigDB (https://www.gsea-msigdb.org/gsea/msigdb/), was employed for GSEA of naive/memory and effector signatures. Gene sets of interest were visualized on a bubble plot, generated using the ggplot2 R package. Pathway analysis was performed using Panther DB (https://www.pantherdb.org/). Significant pathways with *p*-value < 0.05 were selected for downstream analysis. Pathways of interest showing a reverse relationship between *Lmo4-Thy1.1* and *Myb*-deficient T cells were visualized on bidirectional bar plot. The *Stat3* gene regulatory network was created using Cytoscape software and STRING database together with our RNA-seq dataset.

### Statistical analyses

Two-tailed Student’s *t*-test was performed with Graphpad Prism 9 software for comparison of data such as gene expression levels, cell proliferation, and functionality (numbers and percentages). Comparisons between multiple groups were done with a one-way ANOVA test. Tumor growth graphs were analyzed using Wilcoxon rank-sum test on tumor growth slopes. For comparison of survival curves, a log-rank (Mantel–Cox) test was used. Fisher’s exact test was used to calculate the significance of the overlapping pathways in the Venn diagram. GSEA’s significance was assessed using the Kolmogorov–Smirnov test.

### Supplementary information

Supplementary Materials Word template

## Data Availability

Mouse Sequencing Data is available from GEO: GSE248171. All software and packages used were publicly accessible, and this study does not report original codes. Any additional information required to reanalyze the data reported in this paper is available from the lead contact upon request (luca.gattinoni@lit.eu).
