# Supplementary material for: LIM-domain-only 4 (LMO4) enhances CD8+ T-cell stemness and tumor rejection by boosting IL-21-STAT3 signaling
Source: Signal Transduct Target Ther. 2024 Aug 9;9:199. doi: 10.1038/s41392-024-01915-z (PMC11310520; doi:10.1038/s41392-024-01915-z)
Supplement: Supplementary file 1 — Supplementary Materials Word template [file 41392_2024_1915_MOESM1_ESM.docx]

Supplementary Materials for

LIM-domain-only 4 (LMO4) enhances CD8^+^ T-cell stemness and tumor rejection by boosting IL-21-STAT3 signaling

Roland C. Schelker, Jessica Fioravanti, Fabio Mastrogiovanni, Jeremy G. Baldwin, Nisha Rana, Peng Li, Ping Chen, Timea Vadász, Rosanne Spolski, Christoph Heuser-Loy, Dragana Slavkovic-Lukic, Pedro Noronha, Giuseppe Damiano, Laura Raccosta, Daniela Maggioni, Sree Pullugula, Jian-Xin Lin, Jangsuk Oh, Patrick Grandinetti, Mario Lecce, Leo Hesse, Emilia Kocks, Azucena Martín-Santos, Claudia Gebhard, William G. Telford, Yun Ji, Nicholas P. Restifo, Vincenzo Russo, Michael Rehli, Wolfgang Herr, Warren J. Leonard, Luca Gattinoni

Correspondence to: roland.schelker@ukr.de (RS), leonardw@nhlbi.nih.gov (LW) or luca.gattinoni@lit.eu (LG)

**This PDF file includes:**

Figures. S1 to S7

Tables S1 to S7

Captions for Figures S1 to S7

Supplementary Reference 51


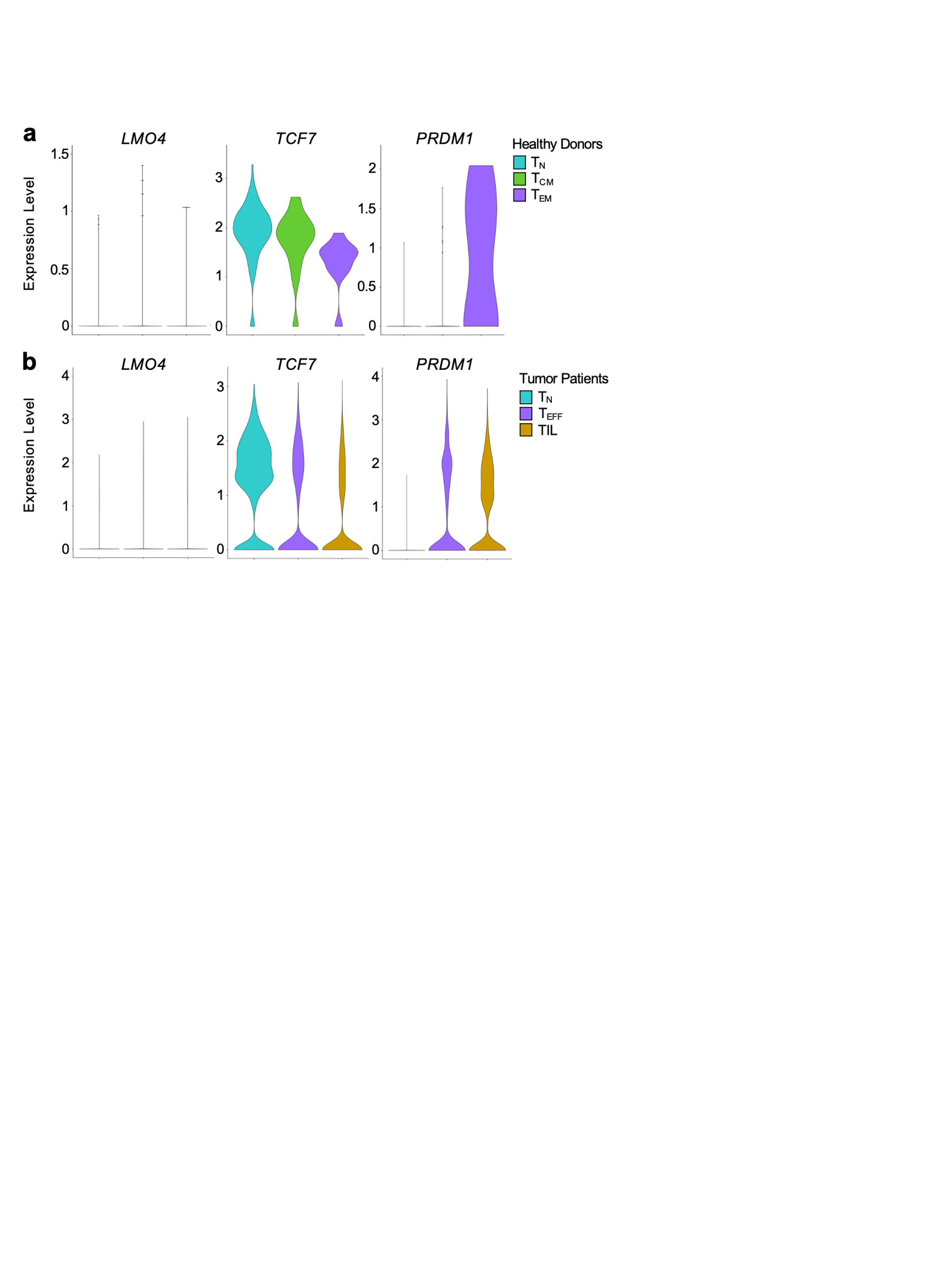
Figure S1.

**LMO4 is poorly expressed in human CD8^+^ T cells.** Violin plots depicting the expression levels of *LMO4*, *TCF7*, and *PRDM1* in (**a**) healthy donors (10X genomic database) or (**b**) HNSCC patients^51^.


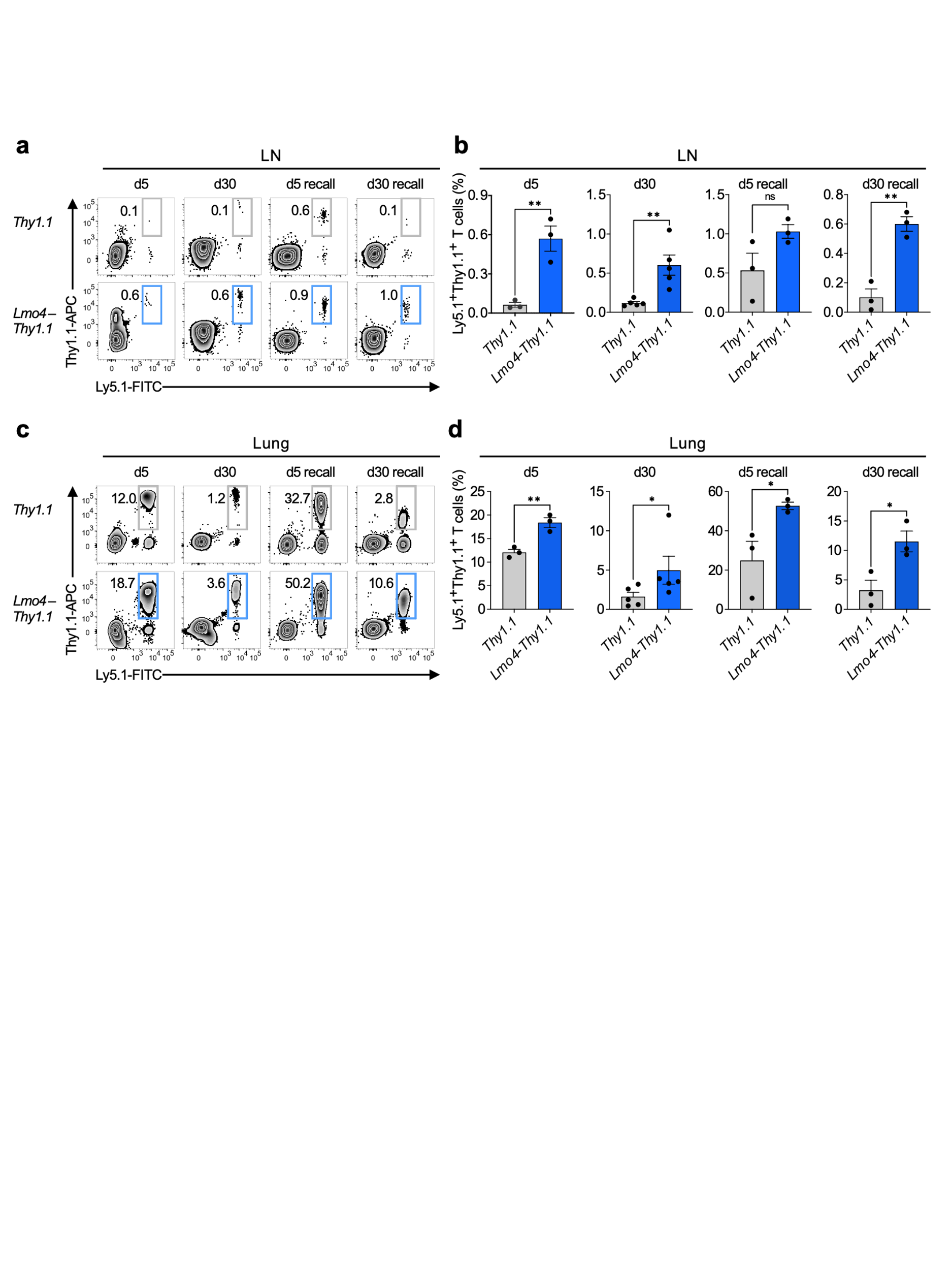
Figure S2.

***Lmo4* overexpression enhances CD8^+^ T cell expansion in lymph nodes and lungs.** (**a-d**) Flow cytometry analysis (**a, c**) and percentages (**b, d**) of lymphatic (**a, b**) and pulmonary (**c, d**) pmel-1 CD8^+^ T cells following transfer of either 10^5^ pmel-1 Ly5.1^+^ *Thy1.1^+^* or *Lmo4-Thy1.1^+^* CD8^+^ T cells into wild-type mice infected with gp100-vv. Assessment was conducted at various time points from 0 to 30 days post-transfer (*n*= 3-5/group). ns, not significant, **P* < 0.05, ***P* < 0.01, (unpaired two-tailed Student’s *t*-test).


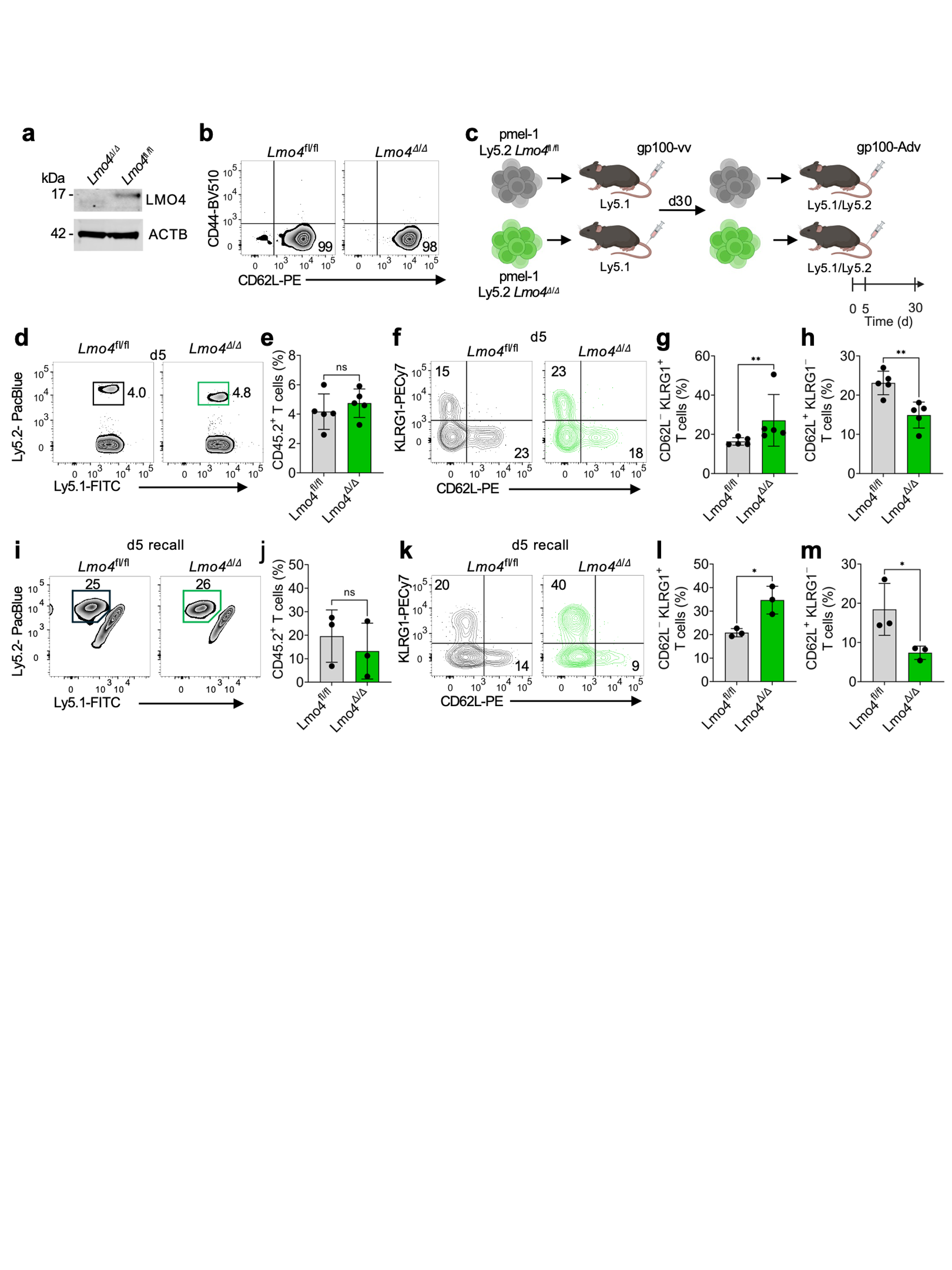
 Figure S3.

***Lmo4* facilitates the generation of stem-like memory T cells by restraining terminal differentiation.** (**a**) Immunoblot showing LMO4 in naive CD8^+^ T cells from pmel-1 *Lmo4^fl/fl^ Cre-ER^T2^* (*Lmo4*^Δ/Δ^) *and* pmel-1 *Lmo4^fl/fl^* mice 5 d after intraperitoneal treatment with tamoxifen. ACTB served as control*.* (**b**) Flow cytometry of pmel-1 *Lmo4^fl/fl^* and *Lmo4*^Δ/Δ^ CD8^+^ T cells after naive T cell isolation. (**c**) Experimental design investigating LMO4’s impact on pmel-1 CD8^+^ T cell primary and secondary immune responses. (**d, e**) Flow cytometry analysis (**d**) and quantification (**e**) of splenic pmel-1 CD8^+^ T cells 5 d after transfer of either 10^5^ pmel-1 Ly5.2^+^ *Lmo4^fl/fl^* or *Lmo4*^Δ/Δ^ CD8^+^ T cells into Ly5.1 mice infected with gp100-vv (*n*= 5 mice/group). (**f-h**) Flow cytometry analysis (**f**) and percentages of CD62L^-^KLRG1^+^ (**g**) and CD62L^+^KLRG1^-^ (**h**) splenic pmel-1 T cells 5 d after transfer as in (**d,e**). (**i, j**) Flow cytometry analysis (**i**) and quantification (**j**) of splenic pmel-1 CD8^+^ T cells 5 d after transfer of either 10^5^ pmel-1 Ly5.2^+^ *Lmo4^fl/fl^* or *Lmo4*^Δ/Δ^ CD8^+^ T cells into Ly5.1/Ly5.2 mice secondary infected with gp100-Adv (*n*= 3 mice/group). (**k-m**) Flow cytometry analysis (**k**) and percentages of CD62L^-^KLRG1^+^ (**l**) and CD62L^+^KLRG1^-^ (**m**) splenic pmel-1 T cells 5 d after transfer as in (**i,j**). ns, not significant, **P* < 0.05, ***P* < 0.01, (unpaired two-tailed Student’s *t*-test).

**
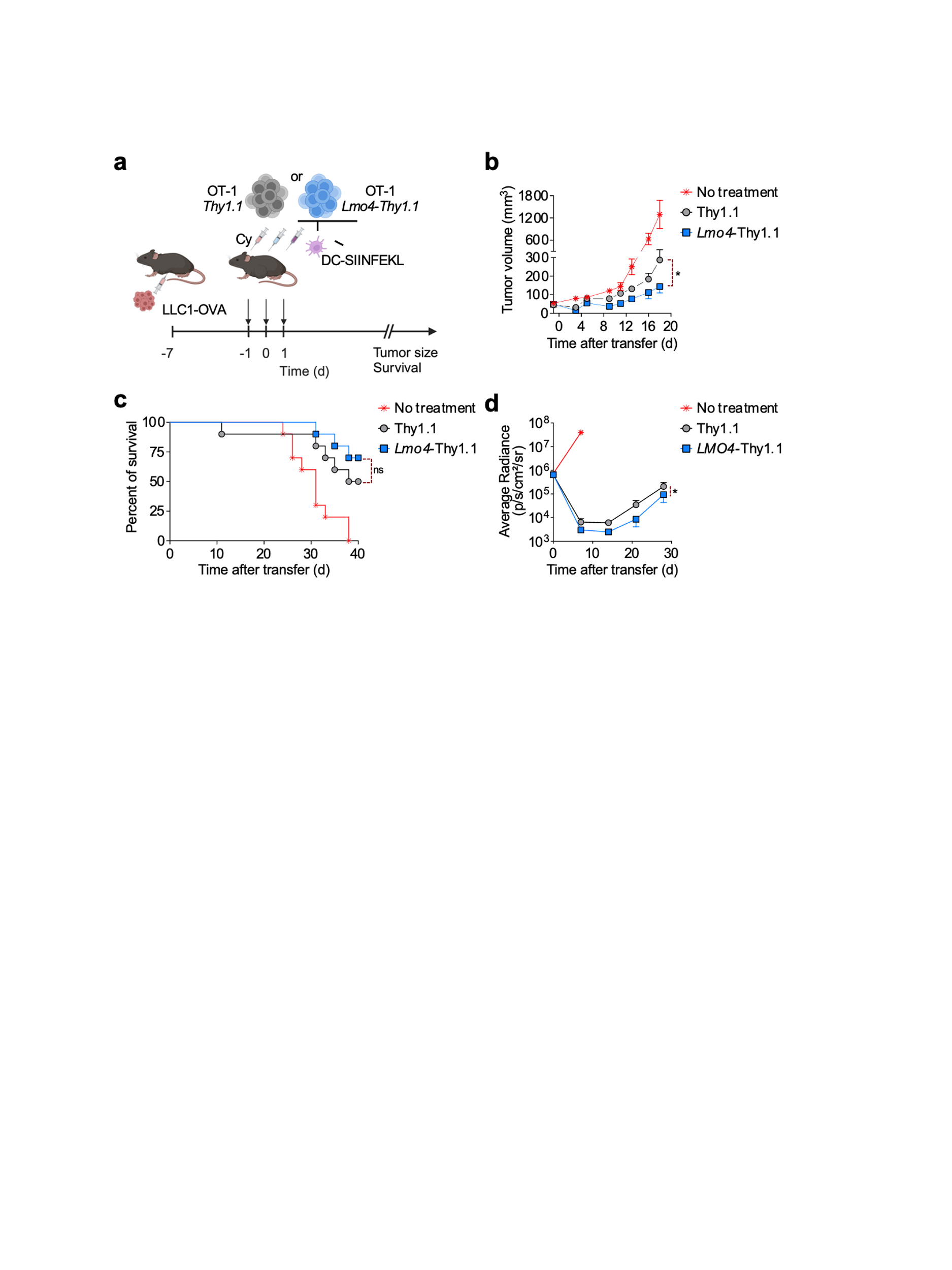
**

**Figure S4.**

**Enforced expression of *Lmo4* improve CD8^+^ T cell-mediated tumor control**. (**a**) Experimental design investigating the effect of *Lmo4* overexpression on the antitumor immune response of OT-1 CD8^+^ T cells. (**b, c**) Tumor size (**b**) and survival (**c**) of LLC1-OVA-bearing mice, after transfer of 10^6^ *Lmo4-Thy1.1 or Thy1.1* transduced OT-1 T cells into lymphodepleted mice and subsequent vaccination with OVA peptide-loaded dendritic cells (*n* = 9-10 mice/group). (**d**) Radiance of NXG mice bearing systemic NALM6-GL leukemia xenografts after adoptive transfer of LMO4-Thy1.1 or Thy1.1 CD19-CAR–modified human CD8^+^ T cells (10^6^cells/mouse) as described in **Fig. 3g** (*n* = 5-7 mice/group). [**P* < 0.05, Wilcoxon rank sum test, two-tailed (**b**), one-tailed (**d**); ns, not significant, log-rank (Mantel-Cox), **c**]. Cy, Cyclophosphamide, OVA, ovalbumin.


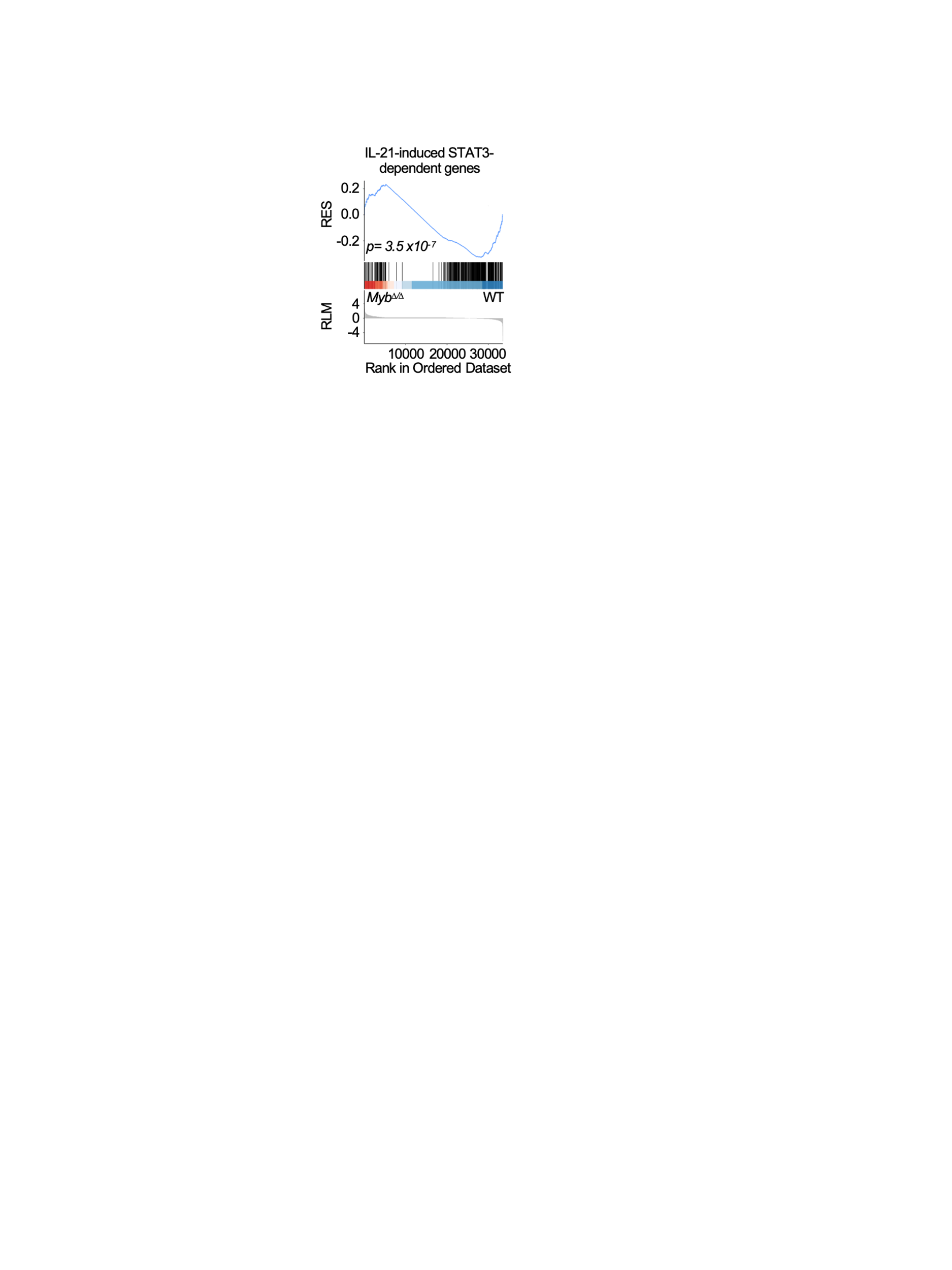
Figure S5.

***Myb* deficiency hinders STAT3 target gene expression.** GSEA showing negative enrichment of genes upregulated in response to IL-21 in CD4^+^ T cells^41^ in CD62L^-^KLRG1^-^*Myb*^Δ/Δ^ CD8^+^ T cells isolated 5 d after transfer of 10^5^ pmel-1 *Myb*^Δ/Δ^ CD8^+^ T cells into wild-type mice infected with gp100-vv. Results are shown in comparison to pmel-1 *Myb*^+/+^ CD8^+^ T cells (WT). P-value was calculated with the Kolmogorov-Smirnov test.


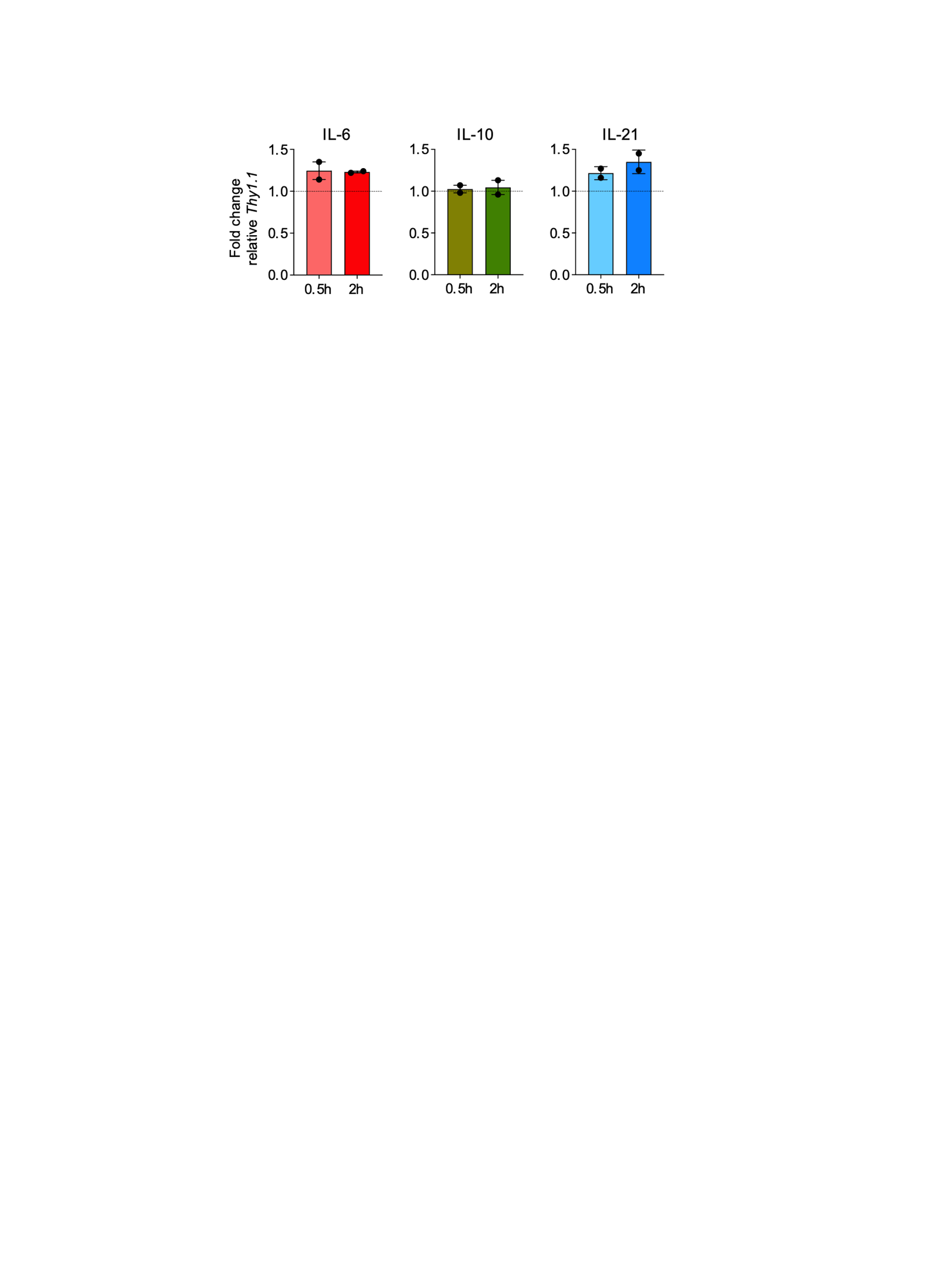


Figure S6.

**LMO4 enhances pSTAT3 in response to IL-6 and IL-21 but not IL-10.** Quantification of pSTAT3 immunoblots in *Lmo4-Thy1.1* T cells normalized by total STAT3 in two independent experiments. Results are presented relative to *Thy1.1* controls.


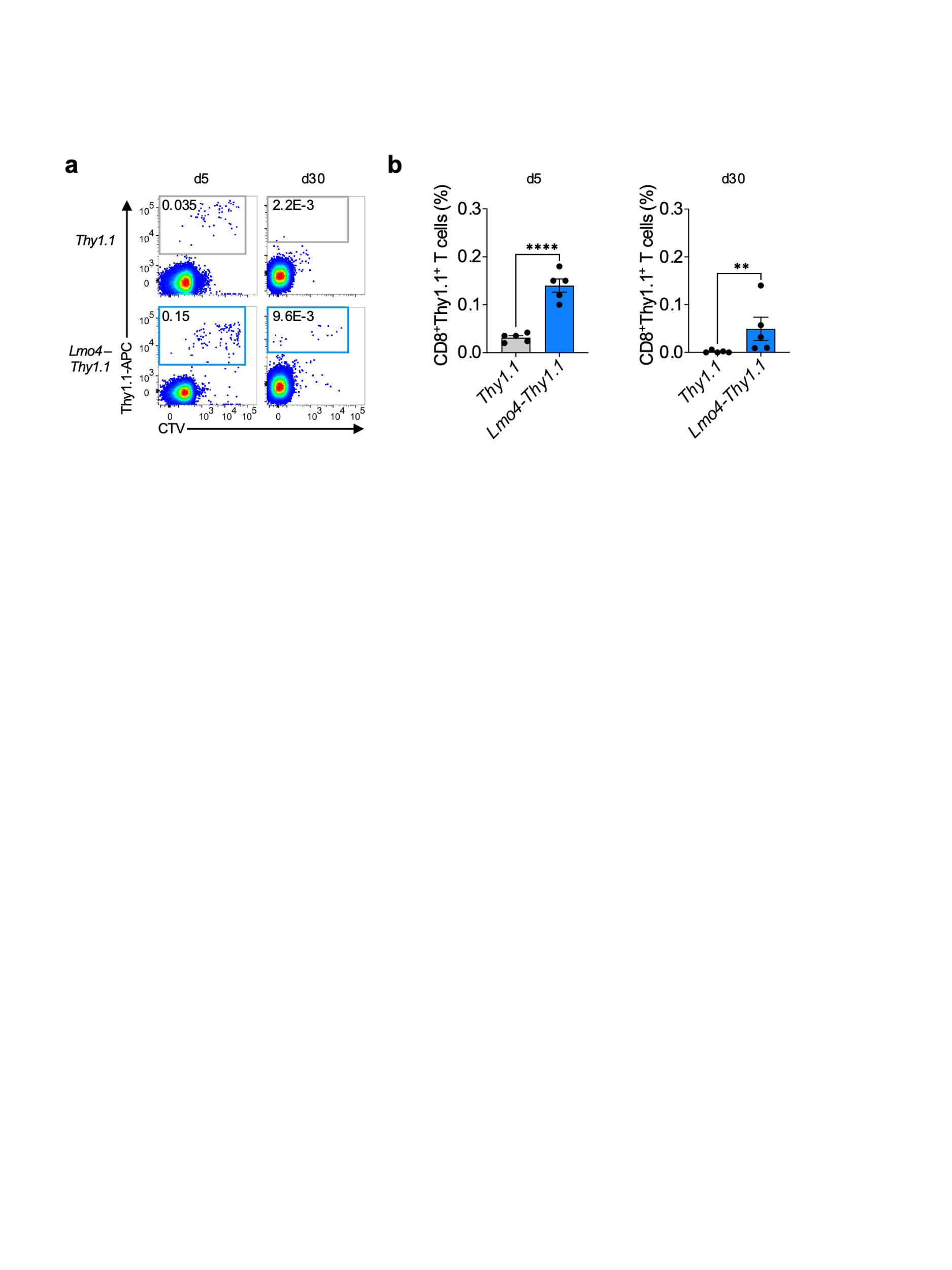


Figure S7.

***Lmo4* overexpression does not induce uncontrolled CD8^+^ T cell expansion.** (**a-b**) Flow cytometry analysis (**a**) and percentages (**b**) of splenic CD8^+^ T cells following transfer of either 2.5 x 10^5^ CTV-labeled *Lmo4-Thy1.1* or *Thy1.1* wild-type CD8^+^ T cells into C57BL/6 mice. Assessment was conducted at 5- and 30-days post-transfer. (*n* = 5 mice/group) ***P* < 0.01, *****P* < 0.0001, (unpaired two-tailed Student’s *t*-test).

Table. S1.

**Supplementary Table 1*.*** sgRNA log2-fold change (IFN- γ^hi/lo^ sorting bin counts) and FDR for each gene tested in a genome-wide CRISPRa screen^16^

| **Gene** | **Screen_Version** | **CRISPRa_or_i** | **CD4_or_CD8** | **Cytokine** | **LFC** | **FDR** |
| --- | --- | --- | --- | --- | --- | --- |
| MUC1 | Primary | CRISPRa | CD8 | IFNG | -2,1793 | 0,000081 |
| JMJD1C | Primary | CRISPRa | CD8 | IFNG | -2,1524 | 0,000081 |
| IKZF3 | Primary | CRISPRa | CD8 | IFNG | -1,8336 | 0,000081 |
| FOXF1 | Primary | CRISPRa | CD8 | IFNG | -1,726 | 0,000081 |
| FOXL2 | Primary | CRISPRa | CD8 | IFNG | -1,7083 | 0,000081 |
| GATA3 | Primary | CRISPRa | CD8 | IFNG | -1,7062 | 0,000081 |
| EBF2 | Primary | CRISPRa | CD8 | IFNG | -1,6911 | 0,000081 |
| GATA6 | Primary | CRISPRa | CD8 | IFNG | -1,4689 | 0,000081 |
| SETD1B | Primary | CRISPRa | CD8 | IFNG | -1,4086 | 0,000081 |
| CEBPB | Primary | CRISPRa | CD8 | IFNG | -1,353 | 0,000081 |
| NFKBIA | Primary | CRISPRa | CD8 | IFNG | -1,2571 | 0,000081 |
| FOXA3 | Primary | CRISPRa | CD8 | IFNG | -1,0691 | 0,000081 |
| TRIM28 | Primary | CRISPRa | CD8 | IFNG | -1,0523 | 0,000081 |
| IKZF1 | Primary | CRISPRa | CD8 | IFNG | -1,0515 | 0,000081 |
| POU2AF1 | Primary | CRISPRa | CD8 | IFNG | -1,0353 | 0,000081 |
| FOXF2 | Primary | CRISPRa | CD8 | IFNG | -1,0319 | 0,000081 |
| PCBP2 | Primary | CRISPRa | CD8 | IFNG | -1,008 | 0,000081 |
| LMO4 | Primary | CRISPRa | CD8 | IFNG | -0,99478 | 0,000081 |
| BTBD2 | Primary | CRISPRa | CD8 | IFNG | -0,56473 | 0,000081 |
| POGZ | Primary | CRISPRa | CD8 | IFNG | -0,093278 | 0,000081 |
| PRKD2 | Primary | CRISPRa | CD8 | IFNG | 0,97168 | 0,000118 |
| EOMES | Primary | CRISPRa | CD8 | IFNG | 1,1139 | 0,000118 |
| FOXD2 | Primary | CRISPRa | CD8 | IFNG | 1,1208 | 0,000118 |
| DEPDC7 | Primary | CRISPRa | CD8 | IFNG | 1,1532 | 0,000118 |
| FOXQ1 | Primary | CRISPRa | CD8 | IFNG | 1,26 | 0,000118 |
| TRIM21 | Primary | CRISPRa | CD8 | IFNG | 1,2796 | 0,000118 |
| WT1 | Primary | CRISPRa | CD8 | IFNG | 1,3288 | 0,000118 |
| FOSL1 | Primary | CRISPRa | CD8 | IFNG | 1,3619 | 0,000118 |
| HELZ2 | Primary | CRISPRa | CD8 | IFNG | 1,4235 | 0,000118 |
| TBX21 | Primary | CRISPRa | CD8 | IFNG | 1,5555 | 0,000118 |
| RELA | Primary | CRISPRa | CD8 | IFNG | 1,6201 | 0,000118 |
| NFATC1 | Primary | CRISPRa | CD8 | IFNG | -1,1648 | 0,000369 |
| CEBPA | Primary | CRISPRa | CD8 | IFNG | -1,1422 | 0,000664 |
| SMAD9 | Primary | CRISPRa | CD8 | IFNG | -1,0469 | 0,000664 |
| NKX2-1 | Primary | CRISPRa | CD8 | IFNG | -0,71765 | 0,000723 |
| FOSB | Primary | CRISPRa | CD8 | IFNG | 0,54855 | 0,000755 |
| TAB3 | Primary | CRISPRa | CD8 | IFNG | 0,56754 | 0,000755 |
| FOXD1 | Primary | CRISPRa | CD8 | IFNG | 0,77968 | 0,000755 |
| PLAGL1 | Primary | CRISPRa | CD8 | IFNG | 0,84927 | 0,000825 |
| MBD1 | Primary | CRISPRa | CD8 | IFNG | -0,78576 | 0,000915 |
| SOX18 | Primary | CRISPRa | CD8 | IFNG | -0,80393 | 0,001011 |
| EIF3K | Primary | CRISPRa | CD8 | IFNG | -0,68948 | 0,001186 |
| LDB2 | Primary | CRISPRa | CD8 | IFNG | 0,63885 | 0,001275 |
| ZC3HAV1 | Primary | CRISPRa | CD8 | IFNG | 1,1201 | 0,001363 |
| NOTO | Primary | CRISPRa | CD8 | IFNG | 0,68807 | 0,001464 |
| NFKB2 | Primary | CRISPRa | CD8 | IFNG | -0,98376 | 0,001465 |
| SOX13 | Primary | CRISPRa | CD8 | IFNG | -0,96429 | 0,001761 |
| PDLIM1 | Primary | CRISPRa | CD8 | IFNG | -0,0049872 | 0,001804 |
| NKX2-6 | Primary | CRISPRa | CD8 | IFNG | -0,59406 | 0,002007 |
| RBFOX2 | Primary | CRISPRa | CD8 | IFNG | -0,81926 | 0,002704 |
| SOX7 | Primary | CRISPRa | CD8 | IFNG | -0,96946 | 0,00318 |
| FOXI3 | Primary | CRISPRa | CD8 | IFNG | -0,79341 | 0,003313 |
| RAP2C | Primary | CRISPRa | CD8 | IFNG | -0,64413 | 0,003339 |
| SMYD3 | Primary | CRISPRa | CD8 | IFNG | -0,62609 | 0,003713 |
| ZBTB43 | Primary | CRISPRa | CD8 | IFNG | 0,17587 | 0,003989 |
| ZNF717 | Primary | CRISPRa | CD8 | IFNG | 0,20445 | 0,003989 |
| DEAF1 | Primary | CRISPRa | CD8 | IFNG | 0,45932 | 0,003989 |
| EP300 | Primary | CRISPRa | CD8 | IFNG | 0,46622 | 0,003989 |
| SAFB2 | Primary | CRISPRa | CD8 | IFNG | 0,46812 | 0,003989 |
| XPA | Primary | CRISPRa | CD8 | IFNG | 0,49838 | 0,003989 |
| PRKCB | Primary | CRISPRa | CD8 | IFNG | 0,50011 | 0,003989 |
| TRADD | Primary | CRISPRa | CD8 | IFNG | 0,6153 | 0,003989 |
| FOXO6 | Primary | CRISPRa | CD8 | IFNG | 0,78114 | 0,003989 |
| POU2F2 | Primary | CRISPRa | CD8 | IFNG | 0,82402 | 0,003989 |
| UBE2D1 | Primary | CRISPRa | CD8 | IFNG | 0,85623 | 0,003989 |
| PRDM1 | Primary | CRISPRa | CD8 | IFNG | 1,2512 | 0,003989 |
| BCL9 | Primary | CRISPRa | CD8 | IFNG | 0,47866 | 0,004199 |
| PAX2 | Primary | CRISPRa | CD8 | IFNG | 0,62766 | 0,004199 |
| CUL1 | Primary | CRISPRa | CD8 | IFNG | 0,69327 | 0,004199 |
| STAT4 | Primary | CRISPRa | CD8 | IFNG | 0,79746 | 0,004199 |
| EPAS1 | Primary | CRISPRa | CD8 | IFNG | 0,80478 | 0,004199 |
| TRIM8 | Primary | CRISPRa | CD8 | IFNG | -0,79767 | 0,004766 |
| CREB5 | Primary | CRISPRa | CD8 | IFNG | 0,88676 | 0,004781 |
| FOXJ1 | Primary | CRISPRa | CD8 | IFNG | -0,43636 | 0,005023 |
| FOXE1 | Primary | CRISPRa | CD8 | IFNG | -0,73219 | 0,005188 |
| ZNF474 | Primary | CRISPRa | CD8 | IFNG | -0,63605 | 0,005188 |
| ATF4 | Primary | CRISPRa | CD8 | IFNG | -0,45875 | 0,005188 |
| SMARCD1 | Primary | CRISPRa | CD8 | IFNG | 0,045092 | 0,005188 |
| BLM | Primary | CRISPRa | CD8 | IFNG | -0,8068 | 0,005615 |
| GATA4 | Primary | CRISPRa | CD8 | IFNG | -0,63721 | 0,005833 |
| NR4A1 | Primary | CRISPRa | CD8 | IFNG | -0,5444 | 0,006277 |
| MYB | Primary | CRISPRa | CD8 | IFNG | -0,73033 | 0,006357 |
| TCEAL2 | Primary | CRISPRa | CD8 | IFNG | -0,7107 | 0,006629 |
| ISL2 | Primary | CRISPRa | CD8 | IFNG | -0,70507 | 0,007331 |
| ZEB2 | Primary | CRISPRa | CD8 | IFNG | -0,69707 | 0,007852 |
| RORB | Primary | CRISPRa | CD8 | IFNG | -0,098568 | 0,008084 |
| CAMK2D | Primary | CRISPRa | CD8 | IFNG | -0,15372 | 0,009562 |
| EBF3 | Primary | CRISPRa | CD8 | IFNG | -0,68776 | 0,00978 |
| RBPMS | Primary | CRISPRa | CD8 | IFNG | -0,5549 | 0,00978 |
| BHLHE40 | Primary | CRISPRa | CD8 | IFNG | -0,50482 | 0,00978 |
| KLF2 | Primary | CRISPRa | CD8 | IFNG | -0,83088 | 0,010018 |
| NR2F1 | Primary | CRISPRa | CD8 | IFNG | -0,67346 | 0,010018 |
| EBF1 | Primary | CRISPRa | CD8 | IFNG | -0,52557 | 0,010107 |
| TLX1 | Primary | CRISPRa | CD8 | IFNG | -0,73239 | 0,010184 |
| KLF4 | Primary | CRISPRa | CD8 | IFNG | -0,39959 | 0,010184 |
| SMAD4 | Primary | CRISPRa | CD8 | IFNG | -0,63568 | 0,010423 |
| ZKSCAN4 | Primary | CRISPRa | CD8 | IFNG | -0,61026 | 0,010458 |
| BACH2 | Primary | CRISPRa | CD8 | IFNG | -0,58857 | 0,010458 |
| CIPC | Primary | CRISPRa | CD8 | IFNG | 0,064263 | 0,011939 |
| BCL9L | Primary | CRISPRa | CD8 | IFNG | 0,4067 | 0,011939 |
| TAF15 | Primary | CRISPRa | CD8 | IFNG | 0,51387 | 0,011939 |
| GSX1 | Primary | CRISPRa | CD8 | IFNG | 0,57205 | 0,011939 |
| SP3 | Primary | CRISPRa | CD8 | IFNG | 0,6684 | 0,011939 |
| CHD4 | Primary | CRISPRa | CD8 | IFNG | 0,70574 | 0,011939 |
| SIRT1 | Primary | CRISPRa | CD8 | IFNG | 0,7588 | 0,011939 |
| NFKB1 | Primary | CRISPRa | CD8 | IFNG | 0,84437 | 0,011939 |
| TBX2 | Primary | CRISPRa | CD8 | IFNG | -0,42866 | 0,012767 |
| TBX3 | Primary | CRISPRa | CD8 | IFNG | -0,79136 | 0,012795 |
| BAHD1 | Primary | CRISPRa | CD8 | IFNG | -0,37191 | 0,013843 |
| RREB1 | Primary | CRISPRa | CD8 | IFNG | -0,62163 | 0,014307 |
| BTRC | Primary | CRISPRa | CD8 | IFNG | -0,54763 | 0,014527 |
| ZNF416 | Primary | CRISPRa | CD8 | IFNG | -0,33694 | 0,014781 |
| CARF | Primary | CRISPRa | CD8 | IFNG | -0,58767 | 0,020934 |
| TNRC6A | Primary | CRISPRa | CD8 | IFNG | 0,27605 | 0,02229 |
| KAT2B | Primary | CRISPRa | CD8 | IFNG | 0,28564 | 0,02229 |
| ANKRD1 | Primary | CRISPRa | CD8 | IFNG | 0,56041 | 0,02229 |
| IRX5 | Primary | CRISPRa | CD8 | IFNG | 0,59461 | 0,02229 |
| SRC | Primary | CRISPRa | CD8 | IFNG | 0,60299 | 0,02229 |
| NFATC2 | Primary | CRISPRa | CD8 | IFNG | 0,63344 | 0,02229 |
| BAZ1A | Primary | CRISPRa | CD8 | IFNG | 0,73813 | 0,02229 |
| REL | Primary | CRISPRa | CD8 | IFNG | 0,72407 | 0,022577 |
| IVNS1ABP | Primary | CRISPRa | CD8 | IFNG | 0,15687 | 0,022613 |
| ZNF417 | Primary | CRISPRa | CD8 | IFNG | -0,24001 | 0,022614 |
| KDM5B | Primary | CRISPRa | CD8 | IFNG | 0,60451 | 0,022614 |
| PRDM13 | Primary | CRISPRa | CD8 | IFNG | 0,86783 | 0,022614 |
| ZNF630 | Primary | CRISPRa | CD8 | IFNG | 0,31001 | 0,022628 |
| MXD3 | Primary | CRISPRa | CD8 | IFNG | 0,68625 | 0,022628 |
| ZBTB25 | Primary | CRISPRa | CD8 | IFNG | -0,73016 | 0,022638 |
| ZIC2 | Primary | CRISPRa | CD8 | IFNG | -0,43909 | 0,022727 |
| ICE1 | Primary | CRISPRa | CD8 | IFNG | 0,23577 | 0,023666 |
| ZNF592 | Primary | CRISPRa | CD8 | IFNG | 0,60495 | 0,023666 |
| RNF8 | Primary | CRISPRa | CD8 | IFNG | 0,67983 | 0,023666 |
| NUP98 | Primary | CRISPRa | CD8 | IFNG | 0,55816 | 0,023917 |
| RFX6 | Primary | CRISPRa | CD8 | IFNG | 0,59285 | 0,024279 |
| SUMO1 | Primary | CRISPRa | CD8 | IFNG | 0,52471 | 0,024694 |
| ZNF580 | Primary | CRISPRa | CD8 | IFNG | -0,55768 | 0,025515 |
| SQSTM1 | Primary | CRISPRa | CD8 | IFNG | 0,30255 | 0,026493 |
| NR4A3 | Primary | CRISPRa | CD8 | IFNG | -0,59592 | 0,026697 |
| MLLT10 | Primary | CRISPRa | CD8 | IFNG | 0,58427 | 0,027606 |
| KLHL42 | Primary | CRISPRa | CD8 | IFNG | -0,62649 | 0,028672 |
| PRDM16 | Primary | CRISPRa | CD8 | IFNG | -0,47084 | 0,028672 |
| SOX10 | Primary | CRISPRa | CD8 | IFNG | -0,30702 | 0,028672 |
| ZEB1 | Primary | CRISPRa | CD8 | IFNG | -0,43314 | 0,028742 |
| ZNF106 | Primary | CRISPRa | CD8 | IFNG | -0,61397 | 0,029065 |
| CCDC88A | Primary | CRISPRa | CD8 | IFNG | -0,64438 | 0,0291 |
| ZNF445 | Primary | CRISPRa | CD8 | IFNG | -0,6392 | 0,0291 |
| POU4F2 | Primary | CRISPRa | CD8 | IFNG | -0,56505 | 0,029607 |
| HOXD11 | Primary | CRISPRa | CD8 | IFNG | -0,48021 | 0,029607 |
| THRSP | Primary | CRISPRa | CD8 | IFNG | 0,26091 | 0,030206 |
| KLHL5 | Primary | CRISPRa | CD8 | IFNG | 0,66839 | 0,030206 |
| EZR | Primary | CRISPRa | CD8 | IFNG | -0,29275 | 0,030237 |
| HLX | Primary | CRISPRa | CD8 | IFNG | 0,65386 | 0,030292 |
| GFI1 | Primary | CRISPRa | CD8 | IFNG | 0,45093 | 0,030449 |
| NOTCH1 | Primary | CRISPRa | CD8 | IFNG | 0,63613 | 0,030471 |
| PLEK2 | Primary | CRISPRa | CD8 | IFNG | 0,55708 | 0,030647 |
| SMYD2 | Primary | CRISPRa | CD8 | IFNG | -0,57563 | 0,030964 |
| TRIM15 | Primary | CRISPRa | CD8 | IFNG | -0,42013 | 0,030964 |
| HMGA2 | Primary | CRISPRa | CD8 | IFNG | -0,59479 | 0,031033 |
| KLF3 | Primary | CRISPRa | CD8 | IFNG | 0,48593 | 0,032105 |
| ZNF316 | Primary | CRISPRa | CD8 | IFNG | 0,19261 | 0,034168 |
| PRAME | Primary | CRISPRa | CD8 | IFNG | 0,51549 | 0,034168 |
| CHD3 | Primary | CRISPRa | CD8 | IFNG | 0,65575 | 0,034168 |
| NKRF | Primary | CRISPRa | CD8 | IFNG | 0,49718 | 0,034807 |
| BTK | Primary | CRISPRa | CD8 | IFNG | 0,65343 | 0,03493 |
| RUNX3 | Primary | CRISPRa | CD8 | IFNG | 0,62475 | 0,03501 |
| CCNA1 | Primary | CRISPRa | CD8 | IFNG | 0,178 | 0,035479 |
| MEF2B | Primary | CRISPRa | CD8 | IFNG | 0,37782 | 0,036299 |
| TIRAP | Primary | CRISPRa | CD8 | IFNG | 0,57491 | 0,036299 |
| ZBTB9 | Primary | CRISPRa | CD8 | IFNG | -0,51951 | 0,039843 |
| GABPB1 | Primary | CRISPRa | CD8 | IFNG | -0,35585 | 0,040394 |
| CBFA2T2 | Primary | CRISPRa | CD8 | IFNG | -0,70776 | 0,041465 |
| PLXNA4 | Primary | CRISPRa | CD8 | IFNG | -0,60125 | 0,041525 |
| GCM2 | Primary | CRISPRa | CD8 | IFNG | 0,11538 | 0,041525 |
| TCEAL8 | Primary | CRISPRa | CD8 | IFNG | -0,59573 | 0,041591 |
| NAIF1 | Primary | CRISPRa | CD8 | IFNG | 0,0063915 | 0,042854 |
| PITX1 | Primary | CRISPRa | CD8 | IFNG | 0,52924 | 0,042986 |
| TOB2 | Primary | CRISPRa | CD8 | IFNG | 0,53826 | 0,044325 |
| DCC | Primary | CRISPRa | CD8 | IFNG | -0,032281 | 0,044419 |
| ZGLP1 | Primary | CRISPRa | CD8 | IFNG | 0,30953 | 0,044618 |
| TUT1 | Primary | CRISPRa | CD8 | IFNG | 0,44052 | 0,045128 |
| ZNF16 | Primary | CRISPRa | CD8 | IFNG | 0,03252 | 0,045458 |
| FOXD3 | Primary | CRISPRa | CD8 | IFNG | 0,42335 | 0,045727 |
| SOX21 | Primary | CRISPRa | CD8 | IFNG | -0,056174 | 0,045939 |
| PRMT6 | Primary | CRISPRa | CD8 | IFNG | 0,60377 | 0,045939 |
| LARP1B | Primary | CRISPRa | CD8 | IFNG | 0,40325 | 0,046518 |
| ZNF395 | Primary | CRISPRa | CD8 | IFNG | 0,51584 | 0,046518 |
| PRKAA2 | Primary | CRISPRa | CD8 | IFNG | 0,52477 | 0,046681 |
| MSANTD3 | Primary | CRISPRa | CD8 | IFNG | -0,13662 | 0,046913 |
| EPC1 | Primary | CRISPRa | CD8 | IFNG | 0,34221 | 0,046913 |
| LHX6 | Primary | CRISPRa | CD8 | IFNG | 0,4123 | 0,046913 |
| SETSIP | Primary | CRISPRa | CD8 | IFNG | 0,53347 | 0,046913 |
| TSC22D3 | Primary | CRISPRa | CD8 | IFNG | 0,60552 | 0,046913 |
| HSF4 | Primary | CRISPRa | CD8 | IFNG | -0,019332 | 0,046963 |
| ZNF471 | Primary | CRISPRa | CD8 | IFNG | 0,11809 | 0,046963 |
| ZNF318 | Primary | CRISPRa | CD8 | IFNG | 0,25411 | 0,046963 |
| ETV2 | Primary | CRISPRa | CD8 | IFNG | 0,28619 | 0,046963 |
| ZNF214 | Primary | CRISPRa | CD8 | IFNG | 0,42428 | 0,046963 |
| LRRFIP2 | Primary | CRISPRa | CD8 | IFNG | 0,53162 | 0,046963 |
| PATZ1 | Primary | CRISPRa | CD8 | IFNG | 0,54316 | 0,046963 |
| CDKN1C | Primary | CRISPRa | CD8 | IFNG | 0,7565 | 0,046963 |
| SUB1 | Primary | CRISPRa | CD8 | IFNG | 0,48939 | 0,047531 |
| MYOG | Primary | CRISPRa | CD8 | IFNG | -0,34474 | 0,047751 |
| ARNT | Primary | CRISPRa | CD8 | IFNG | -0,0082459 | 0,048064 |
| IRX3 | Primary | CRISPRa | CD8 | IFNG | 0,44049 | 0,048064 |
| SOHLH2 | Primary | CRISPRa | CD8 | IFNG | 0,63877 | 0,048064 |
| IRF4 | Primary | CRISPRa | CD8 | IFNG | 0,53166 | 0,049061 |
| SSRP1 | Primary | CRISPRa | CD8 | IFNG | 0,10006 | 0,050685 |
| CHUK | Primary | CRISPRa | CD8 | IFNG | -0,52525 | 0,050804 |
| ENY2 | Primary | CRISPRa | CD8 | IFNG | -0,53468 | 0,051174 |
| ZNF713 | Primary | CRISPRa | CD8 | IFNG | -0,52126 | 0,051174 |
| RPS6KA3 | Primary | CRISPRa | CD8 | IFNG | 0,46538 | 0,051194 |
| MLLT6 | Primary | CRISPRa | CD8 | IFNG | 0,54643 | 0,051194 |
| POU2F1 | Primary | CRISPRa | CD8 | IFNG | 0,59339 | 0,051194 |
| ASCC2 | Primary | CRISPRa | CD8 | IFNG | 0,47536 | 0,051401 |
| HSPA5 | Primary | CRISPRa | CD8 | IFNG | -0,012747 | 0,051423 |
| FOXD4L6 | Primary | CRISPRa | CD8 | IFNG | 0,13155 | 0,051423 |
| ZNF774 | Primary | CRISPRa | CD8 | IFNG | 0,20161 | 0,051423 |
| RNF2 | Primary | CRISPRa | CD8 | IFNG | -0,3577 | 0,052853 |
| MAP3K10 | Primary | CRISPRa | CD8 | IFNG | -0,0017233 | 0,053545 |
| ING5 | Primary | CRISPRa | CD8 | IFNG | 0,09187 | 0,053545 |
| ZNF638 | Primary | CRISPRa | CD8 | IFNG | 0,38274 | 0,053545 |
| ZNF7 | Primary | CRISPRa | CD8 | IFNG | 0,47317 | 0,053545 |
| ZNF615 | Primary | CRISPRa | CD8 | IFNG | 0,49599 | 0,053545 |
| MAP2K6 | Primary | CRISPRa | CD8 | IFNG | 0,36073 | 0,05388 |
| ZBTB14 | Primary | CRISPRa | CD8 | IFNG | -0,70773 | 0,054603 |
| HNF1B | Primary | CRISPRa | CD8 | IFNG | -0,56137 | 0,054603 |
| MLXIP | Primary | CRISPRa | CD8 | IFNG | 0,41073 | 0,055281 |
| UBTF | Primary | CRISPRa | CD8 | IFNG | -0,19502 | 0,05562 |
| SP8 | Primary | CRISPRa | CD8 | IFNG | 0,58262 | 0,056022 |
| FOXA1 | Primary | CRISPRa | CD8 | IFNG | -0,59376 | 0,05615 |
| ZNF740 | Primary | CRISPRa | CD8 | IFNG | -0,56191 | 0,05615 |
| ACTB | Primary | CRISPRa | CD8 | IFNG | 0,54879 | 0,056211 |
| NFYB | Primary | CRISPRa | CD8 | IFNG | -0,29689 | 0,056226 |
| RFPL3 | Primary | CRISPRa | CD8 | IFNG | 0,44694 | 0,057463 |
| PSMC4 | Primary | CRISPRa | CD8 | IFNG | 0,053027 | 0,05814 |
| LMO3 | Primary | CRISPRa | CD8 | IFNG | -0,2159 | 0,058359 |
| CUL3 | Primary | CRISPRa | CD8 | IFNG | -0,13191 | 0,058842 |
| PPARG | Primary | CRISPRa | CD8 | IFNG | -0,51363 | 0,059937 |
| CDKN1B | Primary | CRISPRa | CD8 | IFNG | 0,50464 | 0,061168 |
| KCNIP1 | Primary | CRISPRa | CD8 | IFNG | 0,48604 | 0,064418 |
| RCOR3 | Primary | CRISPRa | CD8 | IFNG | -0,60937 | 0,064678 |
| PARP10 | Primary | CRISPRa | CD8 | IFNG | -0,55378 | 0,064678 |
| FOXL1 | Primary | CRISPRa | CD8 | IFNG | -0,52896 | 0,064678 |
| AEBP1 | Primary | CRISPRa | CD8 | IFNG | -0,48087 | 0,064678 |
| ZNF787 | Primary | CRISPRa | CD8 | IFNG | -0,41299 | 0,064678 |
| HLTF | Primary | CRISPRa | CD8 | IFNG | -0,36817 | 0,064678 |
| BRPF3 | Primary | CRISPRa | CD8 | IFNG | -0,35611 | 0,064678 |
| CIR1 | Primary | CRISPRa | CD8 | IFNG | -0,20527 | 0,064678 |
| FOXN3 | Primary | CRISPRa | CD8 | IFNG | -0,68652 | 0,065041 |
| ZNF319 | Primary | CRISPRa | CD8 | IFNG | -0,52819 | 0,065237 |
| NKX2-3 | Primary | CRISPRa | CD8 | IFNG | -0,38868 | 0,065237 |
| CCNT2 | Primary | CRISPRa | CD8 | IFNG | 0,032817 | 0,065237 |
| FOXK1 | Primary | CRISPRa | CD8 | IFNG | -0,22196 | 0,065402 |
| TGIF2 | Primary | CRISPRa | CD8 | IFNG | -0,63338 | 0,066697 |
| DEPDC4 | Primary | CRISPRa | CD8 | IFNG | -0,49633 | 0,066697 |
| GPBP1 | Primary | CRISPRa | CD8 | IFNG | -0,45238 | 0,066697 |
| TRIT1 | Primary | CRISPRa | CD8 | IFNG | -0,37641 | 0,066697 |
| MTPN | Primary | CRISPRa | CD8 | IFNG | -0,29306 | 0,066697 |
| ARNTL2 | Primary | CRISPRa | CD8 | IFNG | -0,048282 | 0,066697 |
| TNRC18 | Primary | CRISPRa | CD8 | IFNG | -0,065813 | 0,067328 |
| HLA-DQB2 | Primary | CRISPRa | CD8 | IFNG | -0,67245 | 0,067514 |
| PRMT3 | Primary | CRISPRa | CD8 | IFNG | -0,57614 | 0,067514 |
| NKX1-1 | Primary | CRISPRa | CD8 | IFNG | -0,3411 | 0,067514 |
| SOX15 | Primary | CRISPRa | CD8 | IFNG | -0,50958 | 0,067572 |
| TULP1 | Primary | CRISPRa | CD8 | IFNG | -0,58321 | 0,068039 |
| CALCOCO1 | Primary | CRISPRa | CD8 | IFNG | -0,20821 | 0,068662 |
| COMMD4 | Primary | CRISPRa | CD8 | IFNG | -0,53427 | 0,068784 |
| KLF10 | Primary | CRISPRa | CD8 | IFNG | -0,49296 | 0,068784 |
| TTC21B | Primary | CRISPRa | CD8 | IFNG | -0,47888 | 0,068784 |
| RORA | Primary | CRISPRa | CD8 | IFNG | -0,44546 | 0,068784 |
| SFMBT1 | Primary | CRISPRa | CD8 | IFNG | -0,42518 | 0,068784 |
| ARHGEF5 | Primary | CRISPRa | CD8 | IFNG | -0,36257 | 0,068784 |
| KMT2E | Primary | CRISPRa | CD8 | IFNG | -0,35848 | 0,068784 |
| ZNF878 | Primary | CRISPRa | CD8 | IFNG | 0,18839 | 0,070249 |
| EGFR | Primary | CRISPRa | CD8 | IFNG | -0,057167 | 0,072651 |
| BARX1 | Primary | CRISPRa | CD8 | IFNG | -0,25998 | 0,072738 |
| ZNF202 | Primary | CRISPRa | CD8 | IFNG | -0,28175 | 0,07444 |
| ID3 | Primary | CRISPRa | CD8 | IFNG | -0,43902 | 0,07453 |
| DMRTB1 | Primary | CRISPRa | CD8 | IFNG | -0,58063 | 0,074731 |
| CROCC | Primary | CRISPRa | CD8 | IFNG | 0,04724 | 0,074857 |
| TLE4 | Primary | CRISPRa | CD8 | IFNG | 0,42909 | 0,074857 |
| ZBED3 | Primary | CRISPRa | CD8 | IFNG | -0,5577 | 0,07542 |
| CD3D | Primary | CRISPRa | CD8 | IFNG | 0,37181 | 0,076181 |
| KAT6A | Primary | CRISPRa | CD8 | IFNG | 0,37891 | 0,076181 |
| PHF8 | Primary | CRISPRa | CD8 | IFNG | 0,45899 | 0,076181 |
| EMX2 | Primary | CRISPRa | CD8 | IFNG | -0,34343 | 0,077005 |
| IFRD1 | Primary | CRISPRa | CD8 | IFNG | -0,22371 | 0,077269 |
| ZNF595 | Primary | CRISPRa | CD8 | IFNG | 0,30299 | 0,077269 |
| TXNIP | Primary | CRISPRa | CD8 | IFNG | 0,56499 | 0,078291 |
| FARSB | Primary | CRISPRa | CD8 | IFNG | 0,53031 | 0,079133 |
| SRF | Primary | CRISPRa | CD8 | IFNG | -0,54622 | 0,079345 |
| ATF3 | Primary | CRISPRa | CD8 | IFNG | 0,51316 | 0,079638 |
| SNAI3 | Primary | CRISPRa | CD8 | IFNG | 0,53118 | 0,079813 |
| MST1R | Primary | CRISPRa | CD8 | IFNG | -0,080493 | 0,080177 |
| LMNA | Primary | CRISPRa | CD8 | IFNG | 0,32087 | 0,080177 |
| PYGO1 | Primary | CRISPRa | CD8 | IFNG | 0,37239 | 0,080177 |
| ZNF710 | Primary | CRISPRa | CD8 | IFNG | 0,4378 | 0,080177 |
| HNRNPAB | Primary | CRISPRa | CD8 | IFNG | 0,47133 | 0,080177 |
| AHCTF1 | Primary | CRISPRa | CD8 | IFNG | 0,48104 | 0,080177 |
| PAGE4 | Primary | CRISPRa | CD8 | IFNG | 0,48581 | 0,080177 |
| RC3H1 | Primary | CRISPRa | CD8 | IFNG | 0,49504 | 0,080177 |
| NFE2 | Primary | CRISPRa | CD8 | IFNG | 0,50432 | 0,080177 |
| MYCBP2 | Primary | CRISPRa | CD8 | IFNG | -0,51951 | 0,08079 |
| CENPF | Primary | CRISPRa | CD8 | IFNG | -0,69099 | 0,081223 |
| ZFP64 | Primary | CRISPRa | CD8 | IFNG | -0,52415 | 0,084664 |
| GLI3 | Primary | CRISPRa | CD8 | IFNG | -0,62534 | 0,087466 |
| S100A9 | Primary | CRISPRa | CD8 | IFNG | 0,036057 | 0,088428 |
| TFCP2L1 | Primary | CRISPRa | CD8 | IFNG | -0,48164 | 0,090154 |
| LEUTX | Primary | CRISPRa | CD8 | IFNG | 0,13265 | 0,090154 |
| TRIM33 | Primary | CRISPRa | CD8 | IFNG | -0,38699 | 0,094677 |
| HIC1 | Primary | CRISPRa | CD8 | IFNG | 0,13374 | 0,097627 |
| ZNF77 | Primary | CRISPRa | CD8 | IFNG | -0,42356 | 0,098407 |
| NCOR1 | Primary | CRISPRa | CD8 | IFNG | -0,59861 | 0,098486 |
| TSG101 | Primary | CRISPRa | CD8 | IFNG | -0,52329 | 0,098486 |
| MINK1 | Primary | CRISPRa | CD8 | IFNG | -0,46438 | 0,100325 |
| PFN1 | Primary | CRISPRa | CD8 | IFNG | -0,43537 | 0,100325 |
| ETV1 | Primary | CRISPRa | CD8 | IFNG | -0,13969 | 0,100325 |
| ZNF831 | Primary | CRISPRa | CD8 | IFNG | -0,50264 | 0,100757 |
| ATF6 | Primary | CRISPRa | CD8 | IFNG | -0,49836 | 0,100757 |
| EVX2 | Primary | CRISPRa | CD8 | IFNG | -0,43901 | 0,105355 |
| PSMC3IP | Primary | CRISPRa | CD8 | IFNG | -0,40912 | 0,106085 |
| ZNF473 | Primary | CRISPRa | CD8 | IFNG | 0,044886 | 0,10624 |
| BTG2 | Primary | CRISPRa | CD8 | IFNG | -0,022888 | 0,10753 |
| MED18 | Primary | CRISPRa | CD8 | IFNG | -0,53586 | 0,107801 |
| ZBTB20 | Primary | CRISPRa | CD8 | IFNG | -0,18659 | 0,107801 |
| THAP9 | Primary | CRISPRa | CD8 | IFNG | -0,43343 | 0,110328 |
| MID1 | Primary | CRISPRa | CD8 | IFNG | -0,48662 | 0,110626 |
| PAGR1 | Primary | CRISPRa | CD8 | IFNG | -0,46441 | 0,111429 |
| HMOX1 | Primary | CRISPRa | CD8 | IFNG | -0,57207 | 0,111479 |
| EVX1 | Primary | CRISPRa | CD8 | IFNG | -0,49343 | 0,111479 |
| ZNF212 | Primary | CRISPRa | CD8 | IFNG | -0,15968 | 0,111479 |
| KCNIP4 | Primary | CRISPRa | CD8 | IFNG | -0,46531 | 0,11432 |
| PASD1 | Primary | CRISPRa | CD8 | IFNG | -0,3825 | 0,11432 |
| ZNF853 | Primary | CRISPRa | CD8 | IFNG | -0,353 | 0,11432 |
| MLLT11 | Primary | CRISPRa | CD8 | IFNG | -0,16012 | 0,11432 |
| MAGEA1 | Primary | CRISPRa | CD8 | IFNG | 0,039214 | 0,11432 |
| MAPK14 | Primary | CRISPRa | CD8 | IFNG | -0,1875 | 0,114874 |
| NEUROG2 | Primary | CRISPRa | CD8 | IFNG | -0,58148 | 0,115838 |
| RORC | Primary | CRISPRa | CD8 | IFNG | -0,46343 | 0,115838 |
| PSMC5 | Primary | CRISPRa | CD8 | IFNG | -0,17187 | 0,115838 |
| HIPK2 | Primary | CRISPRa | CD8 | IFNG | -0,46516 | 0,120017 |
| GSK3B | Primary | CRISPRa | CD8 | IFNG | -0,1378 | 0,120542 |
| BAZ2A | Primary | CRISPRa | CD8 | IFNG | -0,044334 | 0,122418 |
| FOXC1 | Primary | CRISPRa | CD8 | IFNG | -0,5682 | 0,12592 |
| RFXANK | Primary | CRISPRa | CD8 | IFNG | -0,52526 | 0,12592 |
| SIX4 | Primary | CRISPRa | CD8 | IFNG | -0,40914 | 0,131015 |
| CAMK4 | Primary | CRISPRa | CD8 | IFNG | -0,32759 | 0,131015 |
| BEX1 | Primary | CRISPRa | CD8 | IFNG | -0,013855 | 0,131978 |
| AGAP2 | Primary | CRISPRa | CD8 | IFNG | -0,49569 | 0,133072 |
| NFIC | Primary | CRISPRa | CD8 | IFNG | -0,4121 | 0,134753 |
| BHLHB9 | Primary | CRISPRa | CD8 | IFNG | -0,12289 | 0,134851 |
| HDAC4 | Primary | CRISPRa | CD8 | IFNG | -0,46282 | 0,135159 |
| TGIF1 | Primary | CRISPRa | CD8 | IFNG | -0,52802 | 0,135827 |
| SETDB2 | Primary | CRISPRa | CD8 | IFNG | -0,48793 | 0,138055 |
| ANKZF1 | Primary | CRISPRa | CD8 | IFNG | -0,21614 | 0,138055 |
| ZNF296 | Primary | CRISPRa | CD8 | IFNG | -0,49773 | 0,13853 |
| NRK | Primary | CRISPRa | CD8 | IFNG | -0,3037 | 0,140848 |
| ATXN2 | Primary | CRISPRa | CD8 | IFNG | -0,27405 | 0,140848 |
| TCF3 | Primary | CRISPRa | CD8 | IFNG | -0,12409 | 0,140848 |
| CREBL2 | Primary | CRISPRa | CD8 | IFNG | -0,091293 | 0,140848 |
| ZKSCAN5 | Primary | CRISPRa | CD8 | IFNG | -0,49515 | 0,141404 |
| SETDB1 | Primary | CRISPRa | CD8 | IFNG | -0,25063 | 0,141404 |
| ELF1 | Primary | CRISPRa | CD8 | IFNG | -0,49784 | 0,143774 |
| MNT | Primary | CRISPRa | CD8 | IFNG | -0,47975 | 0,143774 |
| NAA15 | Primary | CRISPRa | CD8 | IFNG | -0,59647 | 0,14382 |
| CDX2 | Primary | CRISPRa | CD8 | IFNG | -0,52217 | 0,14382 |
| ZCCHC18 | Primary | CRISPRa | CD8 | IFNG | -0,5206 | 0,14382 |
| CDK8 | Primary | CRISPRa | CD8 | IFNG | -0,46266 | 0,14382 |
| SATB2 | Primary | CRISPRa | CD8 | IFNG | -0,43823 | 0,14382 |
| ZNF503 | Primary | CRISPRa | CD8 | IFNG | -0,38488 | 0,14382 |
| ATOH1 | Primary | CRISPRa | CD8 | IFNG | -0,27145 | 0,14382 |
| PLCB1 | Primary | CRISPRa | CD8 | IFNG | -0,16338 | 0,14382 |
| FOXP4 | Primary | CRISPRa | CD8 | IFNG | -0,50537 | 0,151197 |
| DLX4 | Primary | CRISPRa | CD8 | IFNG | -0,44833 | 0,152502 |
| ZNF546 | Primary | CRISPRa | CD8 | IFNG | -0,46272 | 0,15699 |
| NAB1 | Primary | CRISPRa | CD8 | IFNG | -0,37906 | 0,15699 |
| CDYL2 | Primary | CRISPRa | CD8 | IFNG | -0,51063 | 0,168571 |
| GRM6 | Primary | CRISPRa | CD8 | IFNG | -0,51016 | 0,168571 |
| ZNF614 | Primary | CRISPRa | CD8 | IFNG | -0,42644 | 0,168571 |
| ATF5 | Primary | CRISPRa | CD8 | IFNG | -0,39272 | 0,168571 |
| BRD9 | Primary | CRISPRa | CD8 | IFNG | -0,37186 | 0,168571 |
| SUPT6H | Primary | CRISPRa | CD8 | IFNG | -0,0083877 | 0,168571 |
| IER2 | Primary | CRISPRa | CD8 | IFNG | -0,51785 | 0,168644 |
| ARRB2 | Primary | CRISPRa | CD8 | IFNG | -0,37646 | 0,168644 |
| OLIG3 | Primary | CRISPRa | CD8 | IFNG | -0,35044 | 0,168644 |
| RAI1 | Primary | CRISPRa | CD8 | IFNG | -0,2759 | 0,168644 |
| ZNF12 | Primary | CRISPRa | CD8 | IFNG | -0,05473 | 0,169209 |
| NCOR2 | Primary | CRISPRa | CD8 | IFNG | -0,51939 | 0,170779 |
| ZNF346 | Primary | CRISPRa | CD8 | IFNG | -0,49268 | 0,170779 |
| IFI27 | Primary | CRISPRa | CD8 | IFNG | -0,073471 | 0,170779 |
| TRERF1 | Primary | CRISPRa | CD8 | IFNG | -0,48764 | 0,172206 |
| KDM2A | Primary | CRISPRa | CD8 | IFNG | -0,55084 | 0,175295 |
| ZNF600 | Primary | CRISPRa | CD8 | IFNG | -0,49853 | 0,175295 |
| HDX | Primary | CRISPRa | CD8 | IFNG | -0,49201 | 0,175295 |
| ZNF134 | Primary | CRISPRa | CD8 | IFNG | -0,44624 | 0,175295 |
| DLX3 | Primary | CRISPRa | CD8 | IFNG | -0,3224 | 0,175295 |
| RBMXL1 | Primary | CRISPRa | CD8 | IFNG | -0,3154 | 0,175295 |
| KLHL28 | Primary | CRISPRa | CD8 | IFNG | -0,28491 | 0,175295 |
| PRDM6 | Primary | CRISPRa | CD8 | IFNG | -0,19427 | 0,175295 |
| UTF1 | Primary | CRISPRa | CD8 | IFNG | -0,038668 | 0,175295 |
| CERS3 | Primary | CRISPRa | CD8 | IFNG | -0,077032 | 0,175672 |
| TNIP1 | Primary | CRISPRa | CD8 | IFNG | -0,46341 | 0,179561 |
| KDM2B | Primary | CRISPRa | CD8 | IFNG | -0,44681 | 0,179561 |
| PRDM11 | Primary | CRISPRa | CD8 | IFNG | -0,3811 | 0,179561 |
| MYCL | Primary | CRISPRa | CD8 | IFNG | -0,47527 | 0,179658 |
| YAP1 | Primary | CRISPRa | CD8 | IFNG | -0,36074 | 0,180947 |
| FGFR2 | Primary | CRISPRa | CD8 | IFNG | -0,42372 | 0,181432 |
| DAXX | Primary | CRISPRa | CD8 | IFNG | -0,4869 | 0,182929 |
| GATAD2B | Primary | CRISPRa | CD8 | IFNG | 0,14003 | 0,182929 |
| HAX1 | Primary | CRISPRa | CD8 | IFNG | 0,40492 | 0,18516 |
| CD36 | Primary | CRISPRa | CD8 | IFNG | -0,3692 | 0,185503 |
| HOXA4 | Primary | CRISPRa | CD8 | IFNG | -0,20563 | 0,185503 |
| HDAC6 | Primary | CRISPRa | CD8 | IFNG | -0,20012 | 0,185503 |
| CITED1 | Primary | CRISPRa | CD8 | IFNG | -0,17882 | 0,185503 |
| TFDP1 | Primary | CRISPRa | CD8 | IFNG | 0,018079 | 0,185503 |
| PADI4 | Primary | CRISPRa | CD8 | IFNG | -0,4823 | 0,186297 |
| ACTN1 | Primary | CRISPRa | CD8 | IFNG | -0,44092 | 0,186297 |
| ZNF532 | Primary | CRISPRa | CD8 | IFNG | 0,0058784 | 0,186297 |
| SATB1 | Primary | CRISPRa | CD8 | IFNG | 0,048446 | 0,186297 |
| GTF2H2C | Primary | CRISPRa | CD8 | IFNG | -0,54595 | 0,186299 |
| UBR4 | Primary | CRISPRa | CD8 | IFNG | -0,52445 | 0,187177 |
| CAND1 | Primary | CRISPRa | CD8 | IFNG | -0,50546 | 0,187177 |
| CBFA2T3 | Primary | CRISPRa | CD8 | IFNG | -0,49217 | 0,187177 |
| TRIB1 | Primary | CRISPRa | CD8 | IFNG | -0,44971 | 0,187177 |
| PHF2 | Primary | CRISPRa | CD8 | IFNG | -0,44766 | 0,187177 |
| MAFK | Primary | CRISPRa | CD8 | IFNG | -0,44259 | 0,187177 |
| EGLN1 | Primary | CRISPRa | CD8 | IFNG | -0,41732 | 0,187177 |
| PTPN14 | Primary | CRISPRa | CD8 | IFNG | -0,40537 | 0,187177 |
| UBE2I | Primary | CRISPRa | CD8 | IFNG | -0,39946 | 0,187177 |
| SPEN | Primary | CRISPRa | CD8 | IFNG | -0,22404 | 0,187177 |
| BRD4 | Primary | CRISPRa | CD8 | IFNG | -0,034176 | 0,187177 |
| ZIC1 | Primary | CRISPRa | CD8 | IFNG | -0,45306 | 0,189476 |
| TCF19 | Primary | CRISPRa | CD8 | IFNG | -0,55296 | 0,190449 |
| TIMELESS | Primary | CRISPRa | CD8 | IFNG | -0,23389 | 0,193353 |
| ZFP30 | Primary | CRISPRa | CD8 | IFNG | -0,033412 | 0,193435 |
| ZNF747 | Primary | CRISPRa | CD8 | IFNG | -0,38449 | 0,194425 |
| ZNF20 | Primary | CRISPRa | CD8 | IFNG | -0,44366 | 0,195343 |
| SOX4 | Primary | CRISPRa | CD8 | IFNG | -0,43603 | 0,195343 |
| CDYL | Primary | CRISPRa | CD8 | IFNG | -0,42513 | 0,195343 |
| TLX3 | Primary | CRISPRa | CD8 | IFNG | -0,40812 | 0,195343 |
| ZNF596 | Primary | CRISPRa | CD8 | IFNG | -0,29452 | 0,195343 |
| ZNF610 | Primary | CRISPRa | CD8 | IFNG | -0,14057 | 0,195343 |
| BLZF1 | Primary | CRISPRa | CD8 | IFNG | -0,40745 | 0,19689 |
| CDCA7L | Primary | CRISPRa | CD8 | IFNG | -0,36975 | 0,19689 |
| SETBP1 | Primary | CRISPRa | CD8 | IFNG | -0,29616 | 0,19689 |
| HIVEP2 | Primary | CRISPRa | CD8 | IFNG | -0,26039 | 0,19689 |
| UBE2D3 | Primary | CRISPRa | CD8 | IFNG | 0,034672 | 0,19689 |
| HOXA2 | Primary | CRISPRa | CD8 | IFNG | 0,16424 | 0,19689 |
| LITAF | Primary | CRISPRa | CD8 | IFNG | -0,32575 | 0,197873 |
| ZNF780A | Primary | CRISPRa | CD8 | IFNG | -0,12098 | 0,197873 |
| TFB1M | Primary | CRISPRa | CD8 | IFNG | -0,46715 | 0,199201 |
| ZBTB10 | Primary | CRISPRa | CD8 | IFNG | -0,40512 | 0,199509 |
| GREB1 | Primary | CRISPRa | CD8 | IFNG | -0,21983 | 0,202472 |
| CEBPD | Primary | CRISPRa | CD8 | IFNG | -0,48449 | 0,204177 |
| SOX12 | Primary | CRISPRa | CD8 | IFNG | -0,55782 | 0,208343 |
| PIAS3 | Primary | CRISPRa | CD8 | IFNG | -0,2202 | 0,208343 |
| ZNF33B | Primary | CRISPRa | CD8 | IFNG | -0,42784 | 0,216345 |
| ETV3 | Primary | CRISPRa | CD8 | IFNG | -0,18506 | 0,216345 |
| TRIM69 | Primary | CRISPRa | CD8 | IFNG | -0,027477 | 0,216381 |
| CTDSPL2 | Primary | CRISPRa | CD8 | IFNG | 0,012269 | 0,216381 |
| CXXC1 | Primary | CRISPRa | CD8 | IFNG | -0,34089 | 0,218667 |
| PHF1 | Primary | CRISPRa | CD8 | IFNG | -0,051876 | 0,218667 |
| ZFHX2 | Primary | CRISPRa | CD8 | IFNG | 0,016787 | 0,218667 |
| AMOT | Primary | CRISPRa | CD8 | IFNG | 0,050234 | 0,218667 |
| XPO1 | Primary | CRISPRa | CD8 | IFNG | 0,061332 | 0,218667 |
| COMMD9 | Primary | CRISPRa | CD8 | IFNG | 0,076662 | 0,218667 |
| ZNF136 | Primary | CRISPRa | CD8 | IFNG | 0,086697 | 0,218667 |
| GATAD2A | Primary | CRISPRa | CD8 | IFNG | 0,094956 | 0,218667 |
| TIGD4 | Primary | CRISPRa | CD8 | IFNG | 0,10263 | 0,218667 |
| MAMLD1 | Primary | CRISPRa | CD8 | IFNG | 0,10424 | 0,218667 |
| MED7 | Primary | CRISPRa | CD8 | IFNG | 0,10931 | 0,218667 |
| DDN | Primary | CRISPRa | CD8 | IFNG | 0,11898 | 0,218667 |
| NPM3 | Primary | CRISPRa | CD8 | IFNG | 0,12593 | 0,218667 |
| CUX2 | Primary | CRISPRa | CD8 | IFNG | 0,15746 | 0,218667 |
| RELL2 | Primary | CRISPRa | CD8 | IFNG | 0,18809 | 0,218667 |
| RBBP4 | Primary | CRISPRa | CD8 | IFNG | 0,2121 | 0,218667 |
| PPARGC1A | Primary | CRISPRa | CD8 | IFNG | 0,21252 | 0,218667 |
| ATF6B | Primary | CRISPRa | CD8 | IFNG | 0,26933 | 0,218667 |
| ZNF781 | Primary | CRISPRa | CD8 | IFNG | 0,29818 | 0,218667 |
| ZNF577 | Primary | CRISPRa | CD8 | IFNG | 0,30651 | 0,218667 |
| ZNF431 | Primary | CRISPRa | CD8 | IFNG | 0,31523 | 0,218667 |
| NELFCD | Primary | CRISPRa | CD8 | IFNG | 0,32199 | 0,218667 |
| PURA | Primary | CRISPRa | CD8 | IFNG | 0,32608 | 0,218667 |
| FEZF2 | Primary | CRISPRa | CD8 | IFNG | 0,34603 | 0,218667 |
| FABP4 | Primary | CRISPRa | CD8 | IFNG | 0,36113 | 0,218667 |
| ZNF22 | Primary | CRISPRa | CD8 | IFNG | 0,36289 | 0,218667 |
| TAF1 | Primary | CRISPRa | CD8 | IFNG | 0,37491 | 0,218667 |
| IRX4 | Primary | CRISPRa | CD8 | IFNG | 0,39221 | 0,218667 |
| FBXO5 | Primary | CRISPRa | CD8 | IFNG | 0,39358 | 0,218667 |
| LGR4 | Primary | CRISPRa | CD8 | IFNG | 0,40149 | 0,218667 |
| LIMA1 | Primary | CRISPRa | CD8 | IFNG | 0,40299 | 0,218667 |
| SF3A2 | Primary | CRISPRa | CD8 | IFNG | 0,40803 | 0,218667 |
| AKAP17A | Primary | CRISPRa | CD8 | IFNG | 0,40895 | 0,218667 |
| TRIM25 | Primary | CRISPRa | CD8 | IFNG | 0,41072 | 0,218667 |
| KAT6B | Primary | CRISPRa | CD8 | IFNG | 0,42883 | 0,218667 |
| HOXC8 | Primary | CRISPRa | CD8 | IFNG | 0,4441 | 0,218667 |
| ANKAR | Primary | CRISPRa | CD8 | IFNG | 0,45971 | 0,218667 |
| HOXD13 | Primary | CRISPRa | CD8 | IFNG | 0,46884 | 0,218667 |
| PRB3 | Primary | CRISPRa | CD8 | IFNG | 0,47496 | 0,218667 |
| TULP2 | Primary | CRISPRa | CD8 | IFNG | 0,49681 | 0,218667 |
| PARP12 | Primary | CRISPRa | CD8 | IFNG | 0,50713 | 0,218667 |
| NEDD4L | Primary | CRISPRa | CD8 | IFNG | 0,51113 | 0,218667 |
| MAP2K1 | Primary | CRISPRa | CD8 | IFNG | 0,51352 | 0,218667 |
| MEGF8 | Primary | CRISPRa | CD8 | IFNG | 0,52627 | 0,218667 |
| CSRNP3 | Primary | CRISPRa | CD8 | IFNG | 0,54089 | 0,218667 |
| FBXO41 | Primary | CRISPRa | CD8 | IFNG | 0,62886 | 0,218667 |
| TCF7L1 | Primary | CRISPRa | CD8 | IFNG | -0,29086 | 0,221039 |
| LMO7 | Primary | CRISPRa | CD8 | IFNG | -0,35556 | 0,221287 |
| TADA2B | Primary | CRISPRa | CD8 | IFNG | -0,15579 | 0,2213 |
| ZNF644 | Primary | CRISPRa | CD8 | IFNG | -0,15579 | 0,2213 |
| SIX2 | Primary | CRISPRa | CD8 | IFNG | 0,035303 | 0,2213 |
| CXXC4 | Primary | CRISPRa | CD8 | IFNG | 0,11722 | 0,2213 |
| RNF14 | Primary | CRISPRa | CD8 | IFNG | 0,11788 | 0,2213 |
| KDM4E | Primary | CRISPRa | CD8 | IFNG | 0,12662 | 0,2213 |
| BMP2 | Primary | CRISPRa | CD8 | IFNG | 0,1834 | 0,2213 |
| PIR | Primary | CRISPRa | CD8 | IFNG | 0,27017 | 0,2213 |
| UBA52 | Primary | CRISPRa | CD8 | IFNG | 0,34821 | 0,2213 |
| TCEA1 | Primary | CRISPRa | CD8 | IFNG | 0,377 | 0,2213 |
| MSL3 | Primary | CRISPRa | CD8 | IFNG | 0,45135 | 0,2213 |
| ATF7 | Primary | CRISPRa | CD8 | IFNG | 0,45649 | 0,2213 |
| CBX3 | Primary | CRISPRa | CD8 | IFNG | 0,50941 | 0,2213 |
| NPM1 | Primary | CRISPRa | CD8 | IFNG | 0,53798 | 0,2213 |
| NACC1 | Primary | CRISPRa | CD8 | IFNG | 0,16879 | 0,226042 |
| PAX5 | Primary | CRISPRa | CD8 | IFNG | -0,28349 | 0,227559 |
| ZHX3 | Primary | CRISPRa | CD8 | IFNG | 0,3744 | 0,227559 |
| AKT1 | Primary | CRISPRa | CD8 | IFNG | 0,44423 | 0,227559 |
| KRBOX4 | Primary | CRISPRa | CD8 | IFNG | 0,13378 | 0,227886 |
| PNRC2 | Primary | CRISPRa | CD8 | IFNG | -0,23579 | 0,227968 |
| PRDM8 | Primary | CRISPRa | CD8 | IFNG | -0,18362 | 0,227968 |
| HOXC13 | Primary | CRISPRa | CD8 | IFNG | -0,045113 | 0,227968 |
| NSD2 | Primary | CRISPRa | CD8 | IFNG | 0,19753 | 0,227968 |
| KLF14 | Primary | CRISPRa | CD8 | IFNG | 0,36035 | 0,227968 |
| DACT1 | Primary | CRISPRa | CD8 | IFNG | 0,53064 | 0,227968 |
| ZYX | Primary | CRISPRa | CD8 | IFNG | 0,19889 | 0,228222 |
| ZNF772 | Primary | CRISPRa | CD8 | IFNG | 0,49442 | 0,228222 |
| SAMD4B | Primary | CRISPRa | CD8 | IFNG | -0,5201 | 0,228543 |
| RSF1 | Primary | CRISPRa | CD8 | IFNG | -0,35927 | 0,228606 |
| ZNF778 | Primary | CRISPRa | CD8 | IFNG | -0,37698 | 0,234214 |
| ZNF804A | Primary | CRISPRa | CD8 | IFNG | -0,14236 | 0,235158 |
| SF3A3 | Primary | CRISPRa | CD8 | IFNG | 0,10613 | 0,235539 |
| PRAMEF33 | Primary | CRISPRa | CD8 | IFNG | -0,33101 | 0,23635 |
| KLHL36 | Primary | CRISPRa | CD8 | IFNG | -0,18036 | 0,23635 |
| TRIM9 | Primary | CRISPRa | CD8 | IFNG | -0,0063757 | 0,236692 |
| AHDC1 | Primary | CRISPRa | CD8 | IFNG | -0,46586 | 0,23752 |
| ZNF57 | Primary | CRISPRa | CD8 | IFNG | -0,42109 | 0,237714 |
| BTN3A3 | Primary | CRISPRa | CD8 | IFNG | -0,38074 | 0,237714 |
| PRKN | Primary | CRISPRa | CD8 | IFNG | 0,7262 | 0,295018 |
| SP6 | Primary | CRISPRa | CD8 | IFNG | -0,53263 | 0,296545 |
| C6orf89 | Primary | CRISPRa | CD8 | IFNG | -0,38199 | 0,296545 |
| KLHL3 | Primary | CRISPRa | CD8 | IFNG | -0,28008 | 0,296669 |
| HIPK3 | Primary | CRISPRa | CD8 | IFNG | -0,4203 | 0,298911 |
| CAMK1 | Primary | CRISPRa | CD8 | IFNG | -0,34903 | 0,298911 |
| ZNF720 | Primary | CRISPRa | CD8 | IFNG | -0,29619 | 0,298911 |
| TBL1XR1 | Primary | CRISPRa | CD8 | IFNG | -0,47277 | 0,299137 |
| GTF2H2 | Primary | CRISPRa | CD8 | IFNG | -0,3336 | 0,299372 |
| ZBTB6 | Primary | CRISPRa | CD8 | IFNG | -0,41863 | 0,300871 |
| GSC | Primary | CRISPRa | CD8 | IFNG | -0,4739 | 0,301098 |
| STAT6 | Primary | CRISPRa | CD8 | IFNG | -0,43878 | 0,301098 |
| RAX2 | Primary | CRISPRa | CD8 | IFNG | -0,43562 | 0,301098 |
| FARSA | Primary | CRISPRa | CD8 | IFNG | -0,40777 | 0,301098 |
| SON | Primary | CRISPRa | CD8 | IFNG | -0,40567 | 0,301098 |
| GATA5 | Primary | CRISPRa | CD8 | IFNG | -0,38427 | 0,301098 |
| CARM1 | Primary | CRISPRa | CD8 | IFNG | -0,35661 | 0,301098 |
| FOXD4L4 | Primary | CRISPRa | CD8 | IFNG | -0,35364 | 0,301098 |
| CERS2 | Primary | CRISPRa | CD8 | IFNG | -0,35116 | 0,301098 |
| ELL2 | Primary | CRISPRa | CD8 | IFNG | -0,33083 | 0,301098 |
| STAT2 | Primary | CRISPRa | CD8 | IFNG | -0,31817 | 0,301098 |
| ZNF555 | Primary | CRISPRa | CD8 | IFNG | -0,25634 | 0,301098 |
| RNF112 | Primary | CRISPRa | CD8 | IFNG | -0,048727 | 0,301098 |
| TOE1 | Primary | CRISPRa | CD8 | IFNG | -0,42074 | 0,301374 |
| BHLHA9 | Primary | CRISPRa | CD8 | IFNG | -0,35751 | 0,301374 |
| ZNF599 | Primary | CRISPRa | CD8 | IFNG | -0,34558 | 0,301374 |
| MESP2 | Primary | CRISPRa | CD8 | IFNG | -0,3282 | 0,301374 |
| AHRR | Primary | CRISPRa | CD8 | IFNG | -0,4836 | 0,303919 |
| REST | Primary | CRISPRa | CD8 | IFNG | -0,39282 | 0,303919 |
| SALL2 | Primary | CRISPRa | CD8 | IFNG | -0,26803 | 0,304835 |
| PAX6 | Primary | CRISPRa | CD8 | IFNG | -0,3611 | 0,305006 |
| BACH1 | Primary | CRISPRa | CD8 | IFNG | -0,34946 | 0,305006 |
| ZNF219 | Primary | CRISPRa | CD8 | IFNG | -0,20379 | 0,305006 |
| ZNF277 | Primary | CRISPRa | CD8 | IFNG | -0,18624 | 0,305006 |
| PHTF1 | Primary | CRISPRa | CD8 | IFNG | 0,0052136 | 0,30514 |
| ZSCAN21 | Primary | CRISPRa | CD8 | IFNG | 0,018818 | 0,30514 |
| STAT5B | Primary | CRISPRa | CD8 | IFNG | 0,16421 | 0,30514 |
| ZNF74 | Primary | CRISPRa | CD8 | IFNG | 0,18548 | 0,30514 |
| GPR155 | Primary | CRISPRa | CD8 | IFNG | 0,22539 | 0,30514 |
| DNAJC21 | Primary | CRISPRa | CD8 | IFNG | 0,25294 | 0,30514 |
| CNOT6 | Primary | CRISPRa | CD8 | IFNG | 0,31587 | 0,30514 |
| MAML2 | Primary | CRISPRa | CD8 | IFNG | 0,36124 | 0,30514 |
| ZNF140 | Primary | CRISPRa | CD8 | IFNG | 0,39259 | 0,30514 |
| IRF8 | Primary | CRISPRa | CD8 | IFNG | 0,39274 | 0,30514 |
| DPY30 | Primary | CRISPRa | CD8 | IFNG | 0,39501 | 0,30514 |
| SP110 | Primary | CRISPRa | CD8 | IFNG | 0,39855 | 0,30514 |
| NFKBIZ | Primary | CRISPRa | CD8 | IFNG | 0,40141 | 0,30514 |
| RUNX1 | Primary | CRISPRa | CD8 | IFNG | 0,40875 | 0,30514 |
| PARP14 | Primary | CRISPRa | CD8 | IFNG | 0,41713 | 0,30514 |
| ZNF181 | Primary | CRISPRa | CD8 | IFNG | 0,42476 | 0,30514 |
| HSF2BP | Primary | CRISPRa | CD8 | IFNG | 0,4494 | 0,30514 |
| MPHOSPH8 | Primary | CRISPRa | CD8 | IFNG | 0,46386 | 0,30514 |
| ZC3H18 | Primary | CRISPRa | CD8 | IFNG | 0,49174 | 0,30514 |
| ATF2 | Primary | CRISPRa | CD8 | IFNG | 0,15699 | 0,305946 |
| ZNF585B | Primary | CRISPRa | CD8 | IFNG | 0,381 | 0,305946 |
| ZKSCAN1 | Primary | CRISPRa | CD8 | IFNG | 0,054081 | 0,306237 |
| ZNF81 | Primary | CRISPRa | CD8 | IFNG | -0,28478 | 0,306489 |
| PPRC1 | Primary | CRISPRa | CD8 | IFNG | -0,17444 | 0,306489 |
| TAL2 | Primary | CRISPRa | CD8 | IFNG | -0,020845 | 0,306489 |
| HOXC12 | Primary | CRISPRa | CD8 | IFNG | -0,080134 | 0,306795 |
| TESC | Primary | CRISPRa | CD8 | IFNG | -0,098744 | 0,307296 |
| ZBTB37 | Primary | CRISPRa | CD8 | IFNG | 0,34939 | 0,308505 |
| ZNF304 | Primary | CRISPRa | CD8 | IFNG | 0,085825 | 0,30865 |
| ASCL4 | Primary | CRISPRa | CD8 | IFNG | 0,14066 | 0,30865 |
| NCOA4 | Primary | CRISPRa | CD8 | IFNG | 0,1554 | 0,30865 |
| ZNF384 | Primary | CRISPRa | CD8 | IFNG | 0,31901 | 0,30865 |
| NCOA3 | Primary | CRISPRa | CD8 | IFNG | 0,37458 | 0,30865 |
| LEO1 | Primary | CRISPRa | CD8 | IFNG | 0,40008 | 0,30865 |
| ZNF44 | Primary | CRISPRa | CD8 | IFNG | 0,40078 | 0,30865 |
| CREB3L1 | Primary | CRISPRa | CD8 | IFNG | 0,41376 | 0,30865 |
| THRB | Primary | CRISPRa | CD8 | IFNG | 0,17777 | 0,309076 |
| ZMIZ1 | Primary | CRISPRa | CD8 | IFNG | -0,44611 | 0,312049 |
| RRP8 | Primary | CRISPRa | CD8 | IFNG | -0,42461 | 0,312049 |
| WTIP | Primary | CRISPRa | CD8 | IFNG | -0,35547 | 0,312049 |
| SRSF10 | Primary | CRISPRa | CD8 | IFNG | -0,34785 | 0,312049 |
| ARNT2 | Primary | CRISPRa | CD8 | IFNG | -0,33368 | 0,312049 |
| CXXC5 | Primary | CRISPRa | CD8 | IFNG | -0,28792 | 0,312049 |
| AXIN2 | Primary | CRISPRa | CD8 | IFNG | -0,25989 | 0,312049 |
| ZNF785 | Primary | CRISPRa | CD8 | IFNG | -0,2524 | 0,312049 |
| ZNF578 | Primary | CRISPRa | CD8 | IFNG | -0,089416 | 0,312049 |
| RPS27A | Primary | CRISPRa | CD8 | IFNG | -0,43259 | 0,316051 |
| PCNA | Primary | CRISPRa | CD8 | IFNG | -0,41089 | 0,316051 |
| E4F1 | Primary | CRISPRa | CD8 | IFNG | -0,36733 | 0,316051 |
| MAFF | Primary | CRISPRa | CD8 | IFNG | -0,32577 | 0,316051 |
| BCL3 | Primary | CRISPRa | CD8 | IFNG | -0,52048 | 0,318253 |
| ZNF385D | Primary | CRISPRa | CD8 | IFNG | -0,43564 | 0,318253 |
| TRIM68 | Primary | CRISPRa | CD8 | IFNG | -0,43167 | 0,318253 |
| MBNL3 | Primary | CRISPRa | CD8 | IFNG | -0,41508 | 0,318253 |
| DHX36 | Primary | CRISPRa | CD8 | IFNG | -0,40132 | 0,318253 |
| SRCAP | Primary | CRISPRa | CD8 | IFNG | -0,33244 | 0,318253 |
| ZNF497 | Primary | CRISPRa | CD8 | IFNG | -0,30678 | 0,318253 |
| SUFU | Primary | CRISPRa | CD8 | IFNG | -0,19279 | 0,318253 |
| ADNP2 | Primary | CRISPRa | CD8 | IFNG | -0,15143 | 0,318253 |
| SMYD1 | Primary | CRISPRa | CD8 | IFNG | -0,1223 | 0,318253 |
| ZFHX3 | Primary | CRISPRa | CD8 | IFNG | -0,086592 | 0,318253 |
| LYL1 | Primary | CRISPRa | CD8 | IFNG | -0,069802 | 0,318253 |
| TGS1 | Primary | CRISPRa | CD8 | IFNG | -0,059031 | 0,318253 |
| ZNF660 | Primary | CRISPRa | CD8 | IFNG | -0,057247 | 0,318253 |
| MAP2K2 | Primary | CRISPRa | CD8 | IFNG | 0,0032679 | 0,318253 |
| PGBD1 | Primary | CRISPRa | CD8 | IFNG | 0,017975 | 0,318253 |
| ZNF850 | Primary | CRISPRa | CD8 | IFNG | 0,051457 | 0,318253 |
| ZNF429 | Primary | CRISPRa | CD8 | IFNG | 0,084771 | 0,318253 |
| ELOC | Primary | CRISPRa | CD8 | IFNG | 0,24221 | 0,318253 |
| IRX2 | Primary | CRISPRa | CD8 | IFNG | 0,2915 | 0,318253 |
| NLRP3 | Primary | CRISPRa | CD8 | IFNG | -0,45068 | 0,318627 |
| ZNF24 | Primary | CRISPRa | CD8 | IFNG | -0,3986 | 0,318627 |
| DLX6 | Primary | CRISPRa | CD8 | IFNG | -0,39412 | 0,318627 |
| ZBTB42 | Primary | CRISPRa | CD8 | IFNG | 0,046928 | 0,318627 |
| JADE3 | Primary | CRISPRa | CD8 | IFNG | 0,10204 | 0,318627 |
| TAF10 | Primary | CRISPRa | CD8 | IFNG | -0,3961 | 0,320058 |
| LHX9 | Primary | CRISPRa | CD8 | IFNG | -0,36343 | 0,320058 |
| CBX1 | Primary | CRISPRa | CD8 | IFNG | -0,29128 | 0,320058 |
| KMT2A | Primary | CRISPRa | CD8 | IFNG | -0,20263 | 0,320058 |
| SENP1 | Primary | CRISPRa | CD8 | IFNG | -0,19715 | 0,320058 |
| BRIP1 | Primary | CRISPRa | CD8 | IFNG | 0,12174 | 0,320058 |
| SIRT7 | Primary | CRISPRa | CD8 | IFNG | -0,41972 | 0,32133 |
| ANXA4 | Primary | CRISPRa | CD8 | IFNG | -0,040919 | 0,321749 |
| DMRT2 | Primary | CRISPRa | CD8 | IFNG | -0,39816 | 0,322093 |
| KLHL12 | Primary | CRISPRa | CD8 | IFNG | -0,14933 | 0,365033 |
| NKX2-8 | Primary | CRISPRa | CD8 | IFNG | -0,18863 | 0,365231 |
| MLX | Primary | CRISPRa | CD8 | IFNG | 0,20529 | 0,365231 |
| ZNF502 | Primary | CRISPRa | CD8 | IFNG | -0,5125 | 0,366149 |
| ZNF519 | Primary | CRISPRa | CD8 | IFNG | -0,025399 | 0,366149 |
| ZNF729 | Primary | CRISPRa | CD8 | IFNG | -0,25862 | 0,367119 |
| MECP2 | Primary | CRISPRa | CD8 | IFNG | -0,19156 | 0,367119 |
| CBX8 | Primary | CRISPRa | CD8 | IFNG | -0,16829 | 0,367119 |
| COMMD10 | Primary | CRISPRa | CD8 | IFNG | -0,10897 | 0,367119 |
| BMI1 | Primary | CRISPRa | CD8 | IFNG | 0,29767 | 0,367119 |
| PRAMEF20 | Primary | CRISPRa | CD8 | IFNG | -0,34385 | 0,36921 |
| CSRNP1 | Primary | CRISPRa | CD8 | IFNG | -0,27747 | 0,36921 |
| PYCARD | Primary | CRISPRa | CD8 | IFNG | -0,27513 | 0,36921 |
| ATN1 | Primary | CRISPRa | CD8 | IFNG | -0,1894 | 0,36921 |
| PLK1 | Primary | CRISPRa | CD8 | IFNG | -0,46434 | 0,369343 |
| NHLH2 | Primary | CRISPRa | CD8 | IFNG | -0,36858 | 0,369343 |
| TSN | Primary | CRISPRa | CD8 | IFNG | -0,34887 | 0,369343 |
| SUPT5H | Primary | CRISPRa | CD8 | IFNG | -0,33864 | 0,369343 |
| ZNF593 | Primary | CRISPRa | CD8 | IFNG | -0,3145 | 0,369343 |
| TRIM35 | Primary | CRISPRa | CD8 | IFNG | -0,27754 | 0,369343 |
| ZNF706 | Primary | CRISPRa | CD8 | IFNG | -0,24461 | 0,369343 |
| RNF135 | Primary | CRISPRa | CD8 | IFNG | -0,21835 | 0,369343 |
| DRAP1 | Primary | CRISPRa | CD8 | IFNG | -0,15973 | 0,369343 |
| L3MBTL4 | Primary | CRISPRa | CD8 | IFNG | -0,024962 | 0,369343 |
| KPNA6 | Primary | CRISPRa | CD8 | IFNG | 0,047539 | 0,369343 |
| NOL11 | Primary | CRISPRa | CD8 | IFNG | 0,062241 | 0,369343 |
| IRF3 | Primary | CRISPRa | CD8 | IFNG | 0,10385 | 0,369343 |
| PPARGC1B | Primary | CRISPRa | CD8 | IFNG | -0,45171 | 0,370909 |
| ILF3 | Primary | CRISPRa | CD8 | IFNG | -0,28723 | 0,370909 |
| AHR | Primary | CRISPRa | CD8 | IFNG | -0,26589 | 0,370909 |
| JMY | Primary | CRISPRa | CD8 | IFNG | -0,24983 | 0,370909 |
| RIPPLY1 | Primary | CRISPRa | CD8 | IFNG | -0,21798 | 0,370909 |
| ELOB | Primary | CRISPRa | CD8 | IFNG | -0,076443 | 0,370909 |
| ZNF80 | Primary | CRISPRa | CD8 | IFNG | 0,18281 | 0,370909 |
| YY1 | Primary | CRISPRa | CD8 | IFNG | 0,18411 | 0,370909 |
| TEAD1 | Primary | CRISPRa | CD8 | IFNG | 0,22504 | 0,370909 |
| ZNF337 | Primary | CRISPRa | CD8 | IFNG | -0,38606 | 0,371893 |
| ZNF250 | Primary | CRISPRa | CD8 | IFNG | -0,36691 | 0,371893 |
| ZBTB39 | Primary | CRISPRa | CD8 | IFNG | -0,069587 | 0,371893 |
| SNAPC4 | Primary | CRISPRa | CD8 | IFNG | 0,028466 | 0,371893 |
| TOX3 | Primary | CRISPRa | CD8 | IFNG | -0,26221 | 0,374629 |
| HNRNPD | Primary | CRISPRa | CD8 | IFNG | -0,15405 | 0,374629 |
| JRKL | Primary | CRISPRa | CD8 | IFNG | -0,13833 | 0,374629 |
| WIZ | Primary | CRISPRa | CD8 | IFNG | -0,001476 | 0,374629 |
| SIM1 | Primary | CRISPRa | CD8 | IFNG | 0,0019726 | 0,374629 |
| RGS9 | Primary | CRISPRa | CD8 | IFNG | 0,011439 | 0,374629 |
| PLXNB1 | Primary | CRISPRa | CD8 | IFNG | 0,012547 | 0,374629 |
| EID1 | Primary | CRISPRa | CD8 | IFNG | 0,012722 | 0,374629 |
| UTY | Primary | CRISPRa | CD8 | IFNG | 0,030071 | 0,374629 |
| BSX | Primary | CRISPRa | CD8 | IFNG | 0,037936 | 0,374629 |
| MPRIP | Primary | CRISPRa | CD8 | IFNG | 0,04999 | 0,374629 |
| PURG | Primary | CRISPRa | CD8 | IFNG | 0,08151 | 0,374629 |
| ZXDA | Primary | CRISPRa | CD8 | IFNG | 0,10455 | 0,374629 |
| ZNF17 | Primary | CRISPRa | CD8 | IFNG | 0,10583 | 0,374629 |
| SS18L1 | Primary | CRISPRa | CD8 | IFNG | 0,11318 | 0,374629 |
| CDK13 | Primary | CRISPRa | CD8 | IFNG | 0,14166 | 0,374629 |
| SKOR1 | Primary | CRISPRa | CD8 | IFNG | 0,154 | 0,374629 |
| FOXH1 | Primary | CRISPRa | CD8 | IFNG | 0,16889 | 0,374629 |
| KDM3A | Primary | CRISPRa | CD8 | IFNG | 0,19117 | 0,374629 |
| EMX1 | Primary | CRISPRa | CD8 | IFNG | 0,20045 | 0,374629 |
| MACF1 | Primary | CRISPRa | CD8 | IFNG | 0,20419 | 0,374629 |
| PRRX1 | Primary | CRISPRa | CD8 | IFNG | 0,20437 | 0,374629 |
| SMARCE1 | Primary | CRISPRa | CD8 | IFNG | 0,20438 | 0,374629 |
| RNASE2 | Primary | CRISPRa | CD8 | IFNG | 0,22954 | 0,374629 |
| YEATS4 | Primary | CRISPRa | CD8 | IFNG | 0,2395 | 0,374629 |
| ZNF618 | Primary | CRISPRa | CD8 | IFNG | 0,24032 | 0,374629 |
| TCERG1 | Primary | CRISPRa | CD8 | IFNG | 0,24492 | 0,374629 |
| TRIM17 | Primary | CRISPRa | CD8 | IFNG | 0,26436 | 0,374629 |
| ZNF697 | Primary | CRISPRa | CD8 | IFNG | 0,28034 | 0,374629 |
| ZNF141 | Primary | CRISPRa | CD8 | IFNG | 0,28383 | 0,374629 |
| RAF1 | Primary | CRISPRa | CD8 | IFNG | 0,29066 | 0,374629 |
| NCOA2 | Primary | CRISPRa | CD8 | IFNG | 0,29635 | 0,374629 |
| LRRC14 | Primary | CRISPRa | CD8 | IFNG | 0,29639 | 0,374629 |
| ZNF254 | Primary | CRISPRa | CD8 | IFNG | 0,29978 | 0,374629 |
| CCDC85B | Primary | CRISPRa | CD8 | IFNG | 0,30772 | 0,374629 |
| ZNF418 | Primary | CRISPRa | CD8 | IFNG | 0,31055 | 0,374629 |
| ELANE | Primary | CRISPRa | CD8 | IFNG | 0,31629 | 0,374629 |
| KLF15 | Primary | CRISPRa | CD8 | IFNG | 0,31821 | 0,374629 |
| ZNFX1 | Primary | CRISPRa | CD8 | IFNG | 0,32357 | 0,374629 |
| AIP | Primary | CRISPRa | CD8 | IFNG | 0,32658 | 0,374629 |
| ZNF484 | Primary | CRISPRa | CD8 | IFNG | 0,33133 | 0,374629 |
| NRIP2 | Primary | CRISPRa | CD8 | IFNG | 0,34125 | 0,374629 |
| CCNE1 | Primary | CRISPRa | CD8 | IFNG | 0,34255 | 0,374629 |
| ZNF233 | Primary | CRISPRa | CD8 | IFNG | 0,34458 | 0,374629 |
| PPHLN1 | Primary | CRISPRa | CD8 | IFNG | 0,35043 | 0,374629 |
| PDS5B | Primary | CRISPRa | CD8 | IFNG | 0,35045 | 0,374629 |
| SMARCD3 | Primary | CRISPRa | CD8 | IFNG | 0,36605 | 0,374629 |
| NUP107 | Primary | CRISPRa | CD8 | IFNG | 0,36662 | 0,374629 |
| STOX1 | Primary | CRISPRa | CD8 | IFNG | 0,36996 | 0,374629 |
| AASS | Primary | CRISPRa | CD8 | IFNG | 0,37355 | 0,374629 |
| NFE2L3 | Primary | CRISPRa | CD8 | IFNG | 0,37469 | 0,374629 |
| ARNTL | Primary | CRISPRa | CD8 | IFNG | 0,3754 | 0,374629 |
| ZNF333 | Primary | CRISPRa | CD8 | IFNG | 0,37915 | 0,374629 |
| ANHX | Primary | CRISPRa | CD8 | IFNG | 0,38706 | 0,374629 |
| HDGFL1 | Primary | CRISPRa | CD8 | IFNG | 0,38728 | 0,374629 |
| MPND | Primary | CRISPRa | CD8 | IFNG | 0,3874 | 0,374629 |
| GLI1 | Primary | CRISPRa | CD8 | IFNG | 0,38767 | 0,374629 |
| SFMBT2 | Primary | CRISPRa | CD8 | IFNG | 0,39033 | 0,374629 |
| NFKBID | Primary | CRISPRa | CD8 | IFNG | 0,39318 | 0,374629 |
| MYRFL | Primary | CRISPRa | CD8 | IFNG | 0,39774 | 0,374629 |
| PIAS1 | Primary | CRISPRa | CD8 | IFNG | 0,39872 | 0,374629 |
| RGS14 | Primary | CRISPRa | CD8 | IFNG | 0,40883 | 0,374629 |
| TP53 | Primary | CRISPRa | CD8 | IFNG | 0,41598 | 0,374629 |
| ZNF207 | Primary | CRISPRa | CD8 | IFNG | 0,41646 | 0,374629 |
| HMX3 | Primary | CRISPRa | CD8 | IFNG | 0,41822 | 0,374629 |
| CHD7 | Primary | CRISPRa | CD8 | IFNG | 0,42735 | 0,374629 |
| CCT4 | Primary | CRISPRa | CD8 | IFNG | 0,43156 | 0,374629 |
| HABP4 | Primary | CRISPRa | CD8 | IFNG | 0,44097 | 0,374629 |
| SART3 | Primary | CRISPRa | CD8 | IFNG | 0,44241 | 0,374629 |
| CREB3 | Primary | CRISPRa | CD8 | IFNG | 0,4587 | 0,374629 |
| HDAC7 | Primary | CRISPRa | CD8 | IFNG | 0,47031 | 0,374629 |
| HSFY1 | Primary | CRISPRa | CD8 | IFNG | 0,52065 | 0,374629 |
| FUS | Primary | CRISPRa | CD8 | IFNG | 0,2525 | 0,374939 |
| HOXB3 | Primary | CRISPRa | CD8 | IFNG | 0,1108 | 0,37543 |
| XIAP | Primary | CRISPRa | CD8 | IFNG | 0,18464 | 0,376901 |
| ZNF564 | Primary | CRISPRa | CD8 | IFNG | 0,2684 | 0,376901 |
| BUD23 | Primary | CRISPRa | CD8 | IFNG | -0,16715 | 0,377019 |
| POLE4 | Primary | CRISPRa | CD8 | IFNG | 0,37531 | 0,377019 |
| SNAPC1 | Primary | CRISPRa | CD8 | IFNG | 0,38697 | 0,377019 |
| SMARCA2 | Primary | CRISPRa | CD8 | IFNG | -0,28433 | 0,377179 |
| ETS1 | Primary | CRISPRa | CD8 | IFNG | 0,10844 | 0,377179 |
| FOXA2 | Primary | CRISPRa | CD8 | IFNG | -0,29917 | 0,377422 |
| ARGLU1 | Primary | CRISPRa | CD8 | IFNG | -0,1676 | 0,377422 |
| PRKD1 | Primary | CRISPRa | CD8 | IFNG | 0,21595 | 0,378356 |
| MED30 | Primary | CRISPRa | CD8 | IFNG | -0,17608 | 0,378975 |
| ZBTB5 | Primary | CRISPRa | CD8 | IFNG | -0,090191 | 0,378975 |
| PRNP | Primary | CRISPRa | CD8 | IFNG | 0,18585 | 0,378975 |
| ELP2 | Primary | CRISPRa | CD8 | IFNG | 0,20337 | 0,378975 |
| KLHL4 | Primary | CRISPRa | CD8 | IFNG | 0,22222 | 0,378975 |
| TCF7L2 | Primary | CRISPRa | CD8 | IFNG | 0,27535 | 0,378975 |
| FOXR2 | Primary | CRISPRa | CD8 | IFNG | 0,32329 | 0,378975 |
| POU5F2 | Primary | CRISPRa | CD8 | IFNG | 0,36649 | 0,378975 |
| ZNF362 | Primary | CRISPRa | CD8 | IFNG | 0,38157 | 0,378975 |
| TCF12 | Primary | CRISPRa | CD8 | IFNG | 0,44358 | 0,378975 |
| TFAP2A | Primary | CRISPRa | CD8 | IFNG | -0,20075 | 0,37915 |
| TRRAP | Primary | CRISPRa | CD8 | IFNG | -0,27738 | 0,380121 |
| PRDM12 | Primary | CRISPRa | CD8 | IFNG | 0,10746 | 0,380121 |
| LMO1 | Primary | CRISPRa | CD8 | IFNG | 0,15556 | 0,381474 |
| PACS2 | Primary | CRISPRa | CD8 | IFNG | 0,17293 | 0,381474 |
| ZNF570 | Primary | CRISPRa | CD8 | IFNG | 0,24296 | 0,381474 |
| ZNF280A | Primary | CRISPRa | CD8 | IFNG | 0,36122 | 0,381474 |
| KRTAP5-1 | Primary | CRISPRa | CD8 | IFNG | -0,32119 | 0,381938 |
| VAX2 | Primary | CRISPRa | CD8 | IFNG | -0,25905 | 0,381938 |
| HES3 | Primary | CRISPRa | CD8 | IFNG | -0,066777 | 0,381938 |
| EGLN2 | Primary | CRISPRa | CD8 | IFNG | -0,22676 | 0,382006 |
| NFIX | Primary | CRISPRa | CD8 | IFNG | 0,10361 | 0,382111 |
| FOXD4L1 | Primary | CRISPRa | CD8 | IFNG | 0,48679 | 0,382111 |
| DLX5 | Primary | CRISPRa | CD8 | IFNG | -0,23469 | 0,38272 |
| THAP11 | Primary | CRISPRa | CD8 | IFNG | -0,23414 | 0,38272 |
| ZNF506 | Primary | CRISPRa | CD8 | IFNG | -0,22588 | 0,38272 |
| SNAI2 | Primary | CRISPRa | CD8 | IFNG | -0,2068 | 0,38272 |
| SFPQ | Primary | CRISPRa | CD8 | IFNG | -0,20254 | 0,38272 |
| GTF3C3 | Primary | CRISPRa | CD8 | IFNG | -0,18354 | 0,38272 |
| PARP1 | Primary | CRISPRa | CD8 | IFNG | -0,16321 | 0,38272 |
| KLHL8 | Primary | CRISPRa | CD8 | IFNG | -0,14792 | 0,38272 |
| NPAS2 | Primary | CRISPRa | CD8 | IFNG | -0,12955 | 0,38272 |
| ZNF343 | Primary | CRISPRa | CD8 | IFNG | -0,12836 | 0,38272 |
| PSMD11 | Primary | CRISPRa | CD8 | IFNG | -0,12397 | 0,38272 |
| KDM5D | Primary | CRISPRa | CD8 | IFNG | -0,12148 | 0,38272 |
| MED23 | Primary | CRISPRa | CD8 | IFNG | -0,094671 | 0,38272 |
| PBX2 | Primary | CRISPRa | CD8 | IFNG | -0,093812 | 0,38272 |
| ZBTB3 | Primary | CRISPRa | CD8 | IFNG | -0,091468 | 0,38272 |
| RBBP9 | Primary | CRISPRa | CD8 | IFNG | -0,091398 | 0,38272 |
| IRF2 | Primary | CRISPRa | CD8 | IFNG | -0,089588 | 0,38272 |
| RIPK3 | Primary | CRISPRa | CD8 | IFNG | -0,081562 | 0,38272 |
| ZNF701 | Primary | CRISPRa | CD8 | IFNG | -0,077481 | 0,38272 |
| KMT2D | Primary | CRISPRa | CD8 | IFNG | -0,063598 | 0,38272 |
| ZNF18 | Primary | CRISPRa | CD8 | IFNG | -0,051169 | 0,38272 |
| ZNF562 | Primary | CRISPRa | CD8 | IFNG | -0,034601 | 0,38272 |
| GBX2 | Primary | CRISPRa | CD8 | IFNG | -0,030379 | 0,38272 |
| ATF7IP2 | Primary | CRISPRa | CD8 | IFNG | -0,016662 | 0,38272 |
| DEK | Primary | CRISPRa | CD8 | IFNG | -0,011342 | 0,38272 |
| IRX1 | Primary | CRISPRa | CD8 | IFNG | -0,0053174 | 0,38272 |
| ZNF425 | Primary | CRISPRa | CD8 | IFNG | -0,0043719 | 0,38272 |
| CEBPG | Primary | CRISPRa | CD8 | IFNG | -0,0039924 | 0,38272 |
| ECD | Primary | CRISPRa | CD8 | IFNG | -0,00381 | 0,38272 |
| POU3F3 | Primary | CRISPRa | CD8 | IFNG | -0,0037175 | 0,38272 |
| NRF1 | Primary | CRISPRa | CD8 | IFNG | -0,0012803 | 0,38272 |
| MORF4L1 | Primary | CRISPRa | CD8 | IFNG | 0,011746 | 0,38272 |
| SENP2 | Primary | CRISPRa | CD8 | IFNG | 0,026488 | 0,38272 |
| ZNF160 | Primary | CRISPRa | CD8 | IFNG | 0,038982 | 0,38272 |
| ZCCHC12 | Primary | CRISPRa | CD8 | IFNG | 0,041991 | 0,38272 |
| ZDHHC1 | Primary | CRISPRa | CD8 | IFNG | 0,044158 | 0,38272 |
| TCF4 | Primary | CRISPRa | CD8 | IFNG | 0,04967 | 0,38272 |
| GLIS2 | Primary | CRISPRa | CD8 | IFNG | 0,061149 | 0,38272 |
| CRAMP1 | Primary | CRISPRa | CD8 | IFNG | 0,062691 | 0,38272 |
| PRKCQ | Primary | CRISPRa | CD8 | IFNG | 0,063566 | 0,38272 |
| CDKN2B | Primary | CRISPRa | CD8 | IFNG | 0,065952 | 0,38272 |
| ZNF727 | Primary | CRISPRa | CD8 | IFNG | 0,066815 | 0,38272 |
| RUNX1T1 | Primary | CRISPRa | CD8 | IFNG | 0,06694 | 0,38272 |
| ZNF721 | Primary | CRISPRa | CD8 | IFNG | 0,07469 | 0,38272 |
| ZNF320 | Primary | CRISPRa | CD8 | IFNG | 0,075416 | 0,38272 |
| BTAF1 | Primary | CRISPRa | CD8 | IFNG | 0,075762 | 0,38272 |
| HMBOX1 | Primary | CRISPRa | CD8 | IFNG | 0,080527 | 0,38272 |
| GABPB2 | Primary | CRISPRa | CD8 | IFNG | 0,092245 | 0,38272 |
| ZNF566 | Primary | CRISPRa | CD8 | IFNG | 0,096071 | 0,38272 |
| AKIRIN2 | Primary | CRISPRa | CD8 | IFNG | 0,10552 | 0,38272 |
| YAF2 | Primary | CRISPRa | CD8 | IFNG | 0,10706 | 0,38272 |
| GTF2H4 | Primary | CRISPRa | CD8 | IFNG | 0,11216 | 0,38272 |
| MYT1 | Primary | CRISPRa | CD8 | IFNG | 0,12011 | 0,38272 |
| LPIN1 | Primary | CRISPRa | CD8 | IFNG | 0,12454 | 0,38272 |
| PYHIN1 | Primary | CRISPRa | CD8 | IFNG | 0,12487 | 0,38272 |
| TTF1 | Primary | CRISPRa | CD8 | IFNG | 0,12515 | 0,38272 |
| WWTR1 | Primary | CRISPRa | CD8 | IFNG | 0,12949 | 0,38272 |
| DHX34 | Primary | CRISPRa | CD8 | IFNG | 0,1299 | 0,38272 |
| MYSM1 | Primary | CRISPRa | CD8 | IFNG | 0,1328 | 0,38272 |
| HCLS1 | Primary | CRISPRa | CD8 | IFNG | 0,13353 | 0,38272 |
| BRDT | Primary | CRISPRa | CD8 | IFNG | 0,14186 | 0,38272 |
| GBX1 | Primary | CRISPRa | CD8 | IFNG | 0,14521 | 0,38272 |
| ZNF622 | Primary | CRISPRa | CD8 | IFNG | 0,14724 | 0,38272 |
| MED29 | Primary | CRISPRa | CD8 | IFNG | 0,15201 | 0,38272 |
| ZFY | Primary | CRISPRa | CD8 | IFNG | 0,15835 | 0,38272 |
| ZNF41 | Primary | CRISPRa | CD8 | IFNG | 0,16984 | 0,38272 |
| GTF2F1 | Primary | CRISPRa | CD8 | IFNG | 0,17382 | 0,38272 |
| TIAL1 | Primary | CRISPRa | CD8 | IFNG | 0,18326 | 0,38272 |
| TP53BP2 | Primary | CRISPRa | CD8 | IFNG | 0,18627 | 0,38272 |
| DMBX1 | Primary | CRISPRa | CD8 | IFNG | 0,18978 | 0,38272 |
| SMARCA5 | Primary | CRISPRa | CD8 | IFNG | 0,19662 | 0,38272 |
| ZBTB49 | Primary | CRISPRa | CD8 | IFNG | 0,19686 | 0,38272 |
| ZSCAN10 | Primary | CRISPRa | CD8 | IFNG | 0,1974 | 0,38272 |
| ZC3H7A | Primary | CRISPRa | CD8 | IFNG | 0,19871 | 0,38272 |
| ZNF180 | Primary | CRISPRa | CD8 | IFNG | 0,20072 | 0,38272 |
| E2F5 | Primary | CRISPRa | CD8 | IFNG | 0,2061 | 0,38272 |
| PRAMEF12 | Primary | CRISPRa | CD8 | IFNG | 0,20842 | 0,38272 |
| ZMAT3 | Primary | CRISPRa | CD8 | IFNG | 0,20842 | 0,38272 |
| SIM2 | Primary | CRISPRa | CD8 | IFNG | 0,21615 | 0,38272 |
| ZBTB45 | Primary | CRISPRa | CD8 | IFNG | 0,21953 | 0,38272 |
| NCK1 | Primary | CRISPRa | CD8 | IFNG | 0,23241 | 0,38272 |
| BCL10 | Primary | CRISPRa | CD8 | IFNG | 0,23752 | 0,38272 |
| ZNF513 | Primary | CRISPRa | CD8 | IFNG | 0,2376 | 0,38272 |
| PIN1 | Primary | CRISPRa | CD8 | IFNG | 0,24023 | 0,38272 |
| DIDO1 | Primary | CRISPRa | CD8 | IFNG | 0,2421 | 0,38272 |
| PSMC3 | Primary | CRISPRa | CD8 | IFNG | 0,24248 | 0,38272 |
| TRIM14 | Primary | CRISPRa | CD8 | IFNG | 0,24804 | 0,38272 |
| PIAS4 | Primary | CRISPRa | CD8 | IFNG | 0,2499 | 0,38272 |
| ZNF675 | Primary | CRISPRa | CD8 | IFNG | 0,25636 | 0,38272 |
| ZNF35 | Primary | CRISPRa | CD8 | IFNG | 0,26237 | 0,38272 |
| PLXND1 | Primary | CRISPRa | CD8 | IFNG | 0,26693 | 0,38272 |
| KHDRBS2 | Primary | CRISPRa | CD8 | IFNG | 0,26722 | 0,38272 |
| TRIM3 | Primary | CRISPRa | CD8 | IFNG | 0,26872 | 0,38272 |
| RIMS3 | Primary | CRISPRa | CD8 | IFNG | 0,27077 | 0,38272 |
| KRBOX1 | Primary | CRISPRa | CD8 | IFNG | 0,2746 | 0,38272 |
| SMAD6 | Primary | CRISPRa | CD8 | IFNG | 0,27952 | 0,38272 |
| ZNF776 | Primary | CRISPRa | CD8 | IFNG | 0,28039 | 0,38272 |
| EIF2AK2 | Primary | CRISPRa | CD8 | IFNG | 0,28642 | 0,38272 |
| HMGN2 | Primary | CRISPRa | CD8 | IFNG | 0,28819 | 0,38272 |
| CDC5L | Primary | CRISPRa | CD8 | IFNG | 0,29238 | 0,38272 |
| HOXB8 | Primary | CRISPRa | CD8 | IFNG | 0,29834 | 0,38272 |
| MEF2A | Primary | CRISPRa | CD8 | IFNG | 0,29866 | 0,38272 |
| SMARCAL1 | Primary | CRISPRa | CD8 | IFNG | 0,29945 | 0,38272 |
| WDR75 | Primary | CRISPRa | CD8 | IFNG | 0,29975 | 0,38272 |
| E2F3 | Primary | CRISPRa | CD8 | IFNG | 0,30399 | 0,38272 |
| FOXE3 | Primary | CRISPRa | CD8 | IFNG | 0,30892 | 0,38272 |
| PCSK4 | Primary | CRISPRa | CD8 | IFNG | 0,30946 | 0,38272 |
| MNX1 | Primary | CRISPRa | CD8 | IFNG | 0,312 | 0,38272 |
| ZBTB7C | Primary | CRISPRa | CD8 | IFNG | 0,31563 | 0,38272 |
| ZNF718 | Primary | CRISPRa | CD8 | IFNG | 0,31725 | 0,38272 |
| HMX1 | Primary | CRISPRa | CD8 | IFNG | 0,32037 | 0,38272 |
| CSRNP2 | Primary | CRISPRa | CD8 | IFNG | 0,32043 | 0,38272 |
| CHEK1 | Primary | CRISPRa | CD8 | IFNG | 0,32215 | 0,38272 |
| DDX20 | Primary | CRISPRa | CD8 | IFNG | 0,32275 | 0,38272 |
| INSM2 | Primary | CRISPRa | CD8 | IFNG | 0,32698 | 0,38272 |
| KLF7 | Primary | CRISPRa | CD8 | IFNG | 0,32915 | 0,38272 |
| MAP3K7 | Primary | CRISPRa | CD8 | IFNG | 0,33258 | 0,38272 |
| HMGB4 | Primary | CRISPRa | CD8 | IFNG | 0,33427 | 0,38272 |
| RLIM | Primary | CRISPRa | CD8 | IFNG | 0,33509 | 0,38272 |
| HEY1 | Primary | CRISPRa | CD8 | IFNG | 0,34358 | 0,38272 |
| PRMT2 | Primary | CRISPRa | CD8 | IFNG | 0,34539 | 0,38272 |
| ZKSCAN7 | Primary | CRISPRa | CD8 | IFNG | 0,3465 | 0,38272 |
| RPS6KA1 | Primary | CRISPRa | CD8 | IFNG | 0,34765 | 0,38272 |
| PHF21A | Primary | CRISPRa | CD8 | IFNG | 0,34856 | 0,38272 |
| PARK7 | Primary | CRISPRa | CD8 | IFNG | 0,35355 | 0,38272 |
| TERF2IP | Primary | CRISPRa | CD8 | IFNG | 0,36022 | 0,38272 |
| EGLN3 | Primary | CRISPRa | CD8 | IFNG | 0,36147 | 0,38272 |
| MDFI | Primary | CRISPRa | CD8 | IFNG | 0,36347 | 0,38272 |
| ASCL2 | Primary | CRISPRa | CD8 | IFNG | 0,37659 | 0,38272 |
| TRIM24 | Primary | CRISPRa | CD8 | IFNG | 0,38853 | 0,38272 |
| MYCBP | Primary | CRISPRa | CD8 | IFNG | 0,031468 | 0,384338 |
| NR2C1 | Primary | CRISPRa | CD8 | IFNG | -0,25561 | 0,386328 |
| PRDM14 | Primary | CRISPRa | CD8 | IFNG | -0,22227 | 0,386328 |
| DNAJC2 | Primary | CRISPRa | CD8 | IFNG | -0,063091 | 0,386328 |
| SIX5 | Primary | CRISPRa | CD8 | IFNG | 0,12475 | 0,386328 |
| LHX5 | Primary | CRISPRa | CD8 | IFNG | -0,41811 | 0,386814 |
| ZMYM6 | Primary | CRISPRa | CD8 | IFNG | -0,37378 | 0,386814 |
| ZNF583 | Primary | CRISPRa | CD8 | IFNG | -0,36655 | 0,386814 |
| NKX2-5 | Primary | CRISPRa | CD8 | IFNG | -0,3404 | 0,386814 |
| ELF3 | Primary | CRISPRa | CD8 | IFNG | -0,32636 | 0,386814 |
| PCGF1 | Primary | CRISPRa | CD8 | IFNG | -0,32035 | 0,386814 |
| ZC3H8 | Primary | CRISPRa | CD8 | IFNG | -0,31908 | 0,386814 |
| TRIM26 | Primary | CRISPRa | CD8 | IFNG | -0,31059 | 0,386814 |
| TAF3 | Primary | CRISPRa | CD8 | IFNG | -0,31058 | 0,386814 |
| ZC3H15 | Primary | CRISPRa | CD8 | IFNG | -0,30828 | 0,386814 |
| NEUROG3 | Primary | CRISPRa | CD8 | IFNG | -0,30101 | 0,386814 |
| ZBTB8B | Primary | CRISPRa | CD8 | IFNG | -0,29869 | 0,386814 |
| STK36 | Primary | CRISPRa | CD8 | IFNG | -0,27843 | 0,386814 |
| ID4 | Primary | CRISPRa | CD8 | IFNG | -0,22025 | 0,386814 |
| TBX10 | Primary | CRISPRa | CD8 | IFNG | -0,1725 | 0,386814 |
| TXN | Primary | CRISPRa | CD8 | IFNG | -0,17052 | 0,386814 |
| ONECUT3 | Primary | CRISPRa | CD8 | IFNG | -0,16126 | 0,386814 |
| NFE4 | Primary | CRISPRa | CD8 | IFNG | -0,13685 | 0,386814 |
| DVL3 | Primary | CRISPRa | CD8 | IFNG | -0,12526 | 0,386814 |
| SUZ12 | Primary | CRISPRa | CD8 | IFNG | -0,058349 | 0,386814 |
| CRABP2 | Primary | CRISPRa | CD8 | IFNG | -0,046612 | 0,386814 |
| ZNF84 | Primary | CRISPRa | CD8 | IFNG | -0,037351 | 0,386814 |
| ZNF239 | Primary | CRISPRa | CD8 | IFNG | -0,028556 | 0,386814 |
| FAM170A | Primary | CRISPRa | CD8 | IFNG | 0,036174 | 0,386814 |
| KNTC1 | Primary | CRISPRa | CD8 | IFNG | 0,049731 | 0,386814 |
| ZNF561 | Primary | CRISPRa | CD8 | IFNG | 0,050375 | 0,386814 |
| NLK | Primary | CRISPRa | CD8 | IFNG | 0,093972 | 0,386814 |
| PPP1R10 | Primary | CRISPRa | CD8 | IFNG | 0,11339 | 0,386814 |
| ELK3 | Primary | CRISPRa | CD8 | IFNG | 0,1424 | 0,386814 |
| FMNL2 | Primary | CRISPRa | CD8 | IFNG | 0,19437 | 0,387343 |
| ATRX | Primary | CRISPRa | CD8 | IFNG | 0,35216 | 0,387343 |
| TBL1Y | Primary | CRISPRa | CD8 | IFNG | -0,1369 | 0,38888 |
| CHD6 | Primary | CRISPRa | CD8 | IFNG | -0,035622 | 0,38888 |
| ZNF461 | Primary | CRISPRa | CD8 | IFNG | 0,12401 | 0,38888 |
| KHDRBS3 | Primary | CRISPRa | CD8 | IFNG | 0,21009 | 0,38888 |
| SCAPER | Primary | CRISPRa | CD8 | IFNG | -0,15802 | 0,389938 |
| RXRA | Primary | CRISPRa | CD8 | IFNG | -0,18513 | 0,38995 |
| PRMT5 | Primary | CRISPRa | CD8 | IFNG | 0,035454 | 0,38995 |
| PROP1 | Primary | CRISPRa | CD8 | IFNG | -0,17152 | 0,390246 |
| ZNF34 | Primary | CRISPRa | CD8 | IFNG | 0,057556 | 0,390246 |
| ELP3 | Primary | CRISPRa | CD8 | IFNG | 0,19468 | 0,390246 |
| DZIP1L | Primary | CRISPRa | CD8 | IFNG | -0,13526 | 0,390366 |
| RBM27 | Primary | CRISPRa | CD8 | IFNG | -0,35788 | 0,391106 |
| ZSCAN12 | Primary | CRISPRa | CD8 | IFNG | -0,22952 | 0,391106 |
| DDIT3 | Primary | CRISPRa | CD8 | IFNG | -0,14236 | 0,391106 |
| AFF4 | Primary | CRISPRa | CD8 | IFNG | 0,18053 | 0,391106 |
| PLSCR1 | Primary | CRISPRa | CD8 | IFNG | -0,40211 | 0,391539 |
| MTERF3 | Primary | CRISPRa | CD8 | IFNG | -0,35198 | 0,391539 |
| RFX5 | Primary | CRISPRa | CD8 | IFNG | -0,34703 | 0,391539 |
| ZNF589 | Primary | CRISPRa | CD8 | IFNG | 0,27811 | 0,39231 |
| DR1 | Primary | CRISPRa | CD8 | IFNG | -0,012175 | 0,393379 |
| RAPGEF3 | Primary | CRISPRa | CD8 | IFNG | -0,41176 | 0,397244 |
| SUPT4H1 | Primary | CRISPRa | CD8 | IFNG | -0,29307 | 0,397244 |
| DZIP1 | Primary | CRISPRa | CD8 | IFNG | 0,32915 | 0,424908 |
| TRIM4 | Primary | CRISPRa | CD8 | IFNG | -0,19708 | 0,425036 |
| TXLNG | Primary | CRISPRa | CD8 | IFNG | 0,058629 | 0,425036 |
| ZMYND8 | Primary | CRISPRa | CD8 | IFNG | 0,24672 | 0,425036 |
| ZNF679 | Primary | CRISPRa | CD8 | IFNG | 0,26166 | 0,425036 |
| ZBTB32 | Primary | CRISPRa | CD8 | IFNG | 0,33167 | 0,425036 |
| MTA3 | Primary | CRISPRa | CD8 | IFNG | 0,33869 | 0,425036 |
| RPS14 | Primary | CRISPRa | CD8 | IFNG | 0,18469 | 0,425758 |
| RNASEL | Primary | CRISPRa | CD8 | IFNG | 0,20541 | 0,425758 |
| MAPK11 | Primary | CRISPRa | CD8 | IFNG | 0,25967 | 0,425758 |
| DNAJB1 | Primary | CRISPRa | CD8 | IFNG | 0,2743 | 0,425843 |
| NR2E1 | Primary | CRISPRa | CD8 | IFNG | -0,0037155 | 0,427599 |
| TENM1 | Primary | CRISPRa | CD8 | IFNG | 0,16421 | 0,427599 |
| ZNF648 | Primary | CRISPRa | CD8 | IFNG | -0,18863 | 0,428791 |
| E2F7 | Primary | CRISPRa | CD8 | IFNG | 0,0053875 | 0,428791 |
| ZNF761 | Primary | CRISPRa | CD8 | IFNG | 0,11404 | 0,428791 |
| YOD1 | Primary | CRISPRa | CD8 | IFNG | 0,1372 | 0,428791 |
| IL31RA | Primary | CRISPRa | CD8 | IFNG | 0,15289 | 0,428791 |
| ASH2L | Primary | CRISPRa | CD8 | IFNG | 0,20155 | 0,428791 |
| NPAS4 | Primary | CRISPRa | CD8 | IFNG | 0,26508 | 0,428791 |
| LPIN3 | Primary | CRISPRa | CD8 | IFNG | 0,27022 | 0,428791 |
| ZBTB1 | Primary | CRISPRa | CD8 | IFNG | 0,29214 | 0,428791 |
| HMGB3 | Primary | CRISPRa | CD8 | IFNG | 0,29228 | 0,428791 |
| EGR2 | Primary | CRISPRa | CD8 | IFNG | 0,29675 | 0,428791 |
| CENPJ | Primary | CRISPRa | CD8 | IFNG | 0,30073 | 0,428791 |
| ZSCAN5B | Primary | CRISPRa | CD8 | IFNG | 0,30151 | 0,428791 |
| THAP10 | Primary | CRISPRa | CD8 | IFNG | 0,30893 | 0,428791 |
| ZNF100 | Primary | CRISPRa | CD8 | IFNG | 0,31864 | 0,428791 |
| PKN1 | Primary | CRISPRa | CD8 | IFNG | 0,33193 | 0,428791 |
| ZMYM2 | Primary | CRISPRa | CD8 | IFNG | 0,29296 | 0,429177 |
| TRIM22 | Primary | CRISPRa | CD8 | IFNG | 0,32595 | 0,429232 |
| MYNN | Primary | CRISPRa | CD8 | IFNG | -0,23496 | 0,429474 |
| JUN | Primary | CRISPRa | CD8 | IFNG | -0,10428 | 0,429474 |
| MYO6 | Primary | CRISPRa | CD8 | IFNG | 0,0068282 | 0,429474 |
| AJUBA | Primary | CRISPRa | CD8 | IFNG | -0,31215 | 0,430146 |
| ZSCAN16 | Primary | CRISPRa | CD8 | IFNG | -0,031504 | 0,430831 |
| KLHL1 | Primary | CRISPRa | CD8 | IFNG | 0,1357 | 0,430831 |
| BATF | Primary | CRISPRa | CD8 | IFNG | 0,2097 | 0,430831 |
| ZNF175 | Primary | CRISPRa | CD8 | IFNG | 0,22776 | 0,430831 |
| ZNF514 | Primary | CRISPRa | CD8 | IFNG | 0,23201 | 0,430831 |
| TBR1 | Primary | CRISPRa | CD8 | IFNG | 0,29165 | 0,431357 |
| TIGD6 | Primary | CRISPRa | CD8 | IFNG | -0,062281 | 0,433462 |
| LHX3 | Primary | CRISPRa | CD8 | IFNG | -0,37584 | 0,433622 |
| BHLHE41 | Primary | CRISPRa | CD8 | IFNG | -0,37264 | 0,433622 |
| AKAP8 | Primary | CRISPRa | CD8 | IFNG | -0,31487 | 0,433622 |
| ZNF625 | Primary | CRISPRa | CD8 | IFNG | -0,22664 | 0,433622 |
| RFX8 | Primary | CRISPRa | CD8 | IFNG | -0,20014 | 0,433622 |
| INSR | Primary | CRISPRa | CD8 | IFNG | -0,10261 | 0,433622 |
| IFT74 | Primary | CRISPRa | CD8 | IFNG | 0,00031553 | 0,433622 |
| THAP1 | Primary | CRISPRa | CD8 | IFNG | -0,26625 | 0,433666 |
| HIF3A | Primary | CRISPRa | CD8 | IFNG | -0,16266 | 0,433666 |
| ERBB2 | Primary | CRISPRa | CD8 | IFNG | -0,078065 | 0,433986 |
| HNF4A | Primary | CRISPRa | CD8 | IFNG | -0,09653 | 0,434012 |
| ARID1A | Primary | CRISPRa | CD8 | IFNG | -0,05975 | 0,434012 |
| SRSF2 | Primary | CRISPRa | CD8 | IFNG | -0,057578 | 0,434012 |
| CAMTA2 | Primary | CRISPRa | CD8 | IFNG | 0,015092 | 0,434012 |
| NOTCH3 | Primary | CRISPRa | CD8 | IFNG | 0,040573 | 0,434012 |
| ZFP42 | Primary | CRISPRa | CD8 | IFNG | 0,045969 | 0,434012 |
| ZNF266 | Primary | CRISPRa | CD8 | IFNG | 0,097099 | 0,434012 |
| MIER3 | Primary | CRISPRa | CD8 | IFNG | 0,22154 | 0,434012 |
| TRIM62 | Primary | CRISPRa | CD8 | IFNG | 0,24557 | 0,434012 |
| PPIE | Primary | CRISPRa | CD8 | IFNG | 0,30043 | 0,43436 |
| ZC3H12B | Primary | CRISPRa | CD8 | IFNG | -0,41014 | 0,434487 |
| ONECUT2 | Primary | CRISPRa | CD8 | IFNG | -0,38255 | 0,434487 |
| KCTD1 | Primary | CRISPRa | CD8 | IFNG | -0,28551 | 0,434487 |
| NPAT | Primary | CRISPRa | CD8 | IFNG | -0,21155 | 0,434487 |
| AEBP2 | Primary | CRISPRa | CD8 | IFNG | -0,17422 | 0,434487 |
| RAPGEF4 | Primary | CRISPRa | CD8 | IFNG | -0,16373 | 0,434487 |
| TAF4B | Primary | CRISPRa | CD8 | IFNG | -0,15709 | 0,434487 |
| SS18 | Primary | CRISPRa | CD8 | IFNG | -0,15181 | 0,434487 |
| WDR61 | Primary | CRISPRa | CD8 | IFNG | -0,1433 | 0,434487 |
| ZNF808 | Primary | CRISPRa | CD8 | IFNG | -0,14273 | 0,434487 |
| KLF16 | Primary | CRISPRa | CD8 | IFNG | 0,084791 | 0,434487 |
| BHLHE23 | Primary | CRISPRa | CD8 | IFNG | 0,11969 | 0,434487 |
| DACH1 | Primary | CRISPRa | CD8 | IFNG | 0,14565 | 0,434487 |
| TCF23 | Primary | CRISPRa | CD8 | IFNG | -0,36116 | 0,436423 |
| ADAMTS19 | Primary | CRISPRa | CD8 | IFNG | -0,27966 | 0,436423 |
| NCS1 | Primary | CRISPRa | CD8 | IFNG | -0,19782 | 0,436423 |
| RBM20 | Primary | CRISPRa | CD8 | IFNG | -0,11095 | 0,436423 |
| STAT1 | Primary | CRISPRa | CD8 | IFNG | -0,039219 | 0,436423 |
| FIZ1 | Primary | CRISPRa | CD8 | IFNG | -0,010619 | 0,436423 |
| ZNF423 | Primary | CRISPRa | CD8 | IFNG | -0,36495 | 0,436559 |
| MBNL1 | Primary | CRISPRa | CD8 | IFNG | -0,26695 | 0,437892 |
| ZNF276 | Primary | CRISPRa | CD8 | IFNG | 0,017346 | 0,437892 |
| IRAK1BP1 | Primary | CRISPRa | CD8 | IFNG | -0,058022 | 0,438707 |
| RIN3 | Primary | CRISPRa | CD8 | IFNG | 0,17066 | 0,43961 |
| ZKSCAN2 | Primary | CRISPRa | CD8 | IFNG | 0,26886 | 0,43961 |
| ZBTB2 | Primary | CRISPRa | CD8 | IFNG | -0,24706 | 0,439797 |
| RHOXF1 | Primary | CRISPRa | CD8 | IFNG | -0,11035 | 0,439797 |
| TFEC | Primary | CRISPRa | CD8 | IFNG | -0,058194 | 0,439797 |
| TSC22D4 | Primary | CRISPRa | CD8 | IFNG | 0,23508 | 0,439797 |
| TNKS | Primary | CRISPRa | CD8 | IFNG | -0,3257 | 0,44051 |
| RFX7 | Primary | CRISPRa | CD8 | IFNG | -0,22088 | 0,442147 |
| BHLHE22 | Primary | CRISPRa | CD8 | IFNG | 0,20149 | 0,442147 |
| TGIF2LX | Primary | CRISPRa | CD8 | IFNG | 0,28141 | 0,442147 |
| ZFPL1 | Primary | CRISPRa | CD8 | IFNG | 0,31609 | 0,442147 |
| PRKAA1 | Primary | CRISPRa | CD8 | IFNG | 0,0048043 | 0,443728 |
| SOHLH1 | Primary | CRISPRa | CD8 | IFNG | 0,12897 | 0,444429 |
| TLX2 | Primary | CRISPRa | CD8 | IFNG | -0,057436 | 0,445673 |
| ZNF683 | Primary | CRISPRa | CD8 | IFNG | 0,3217 | 0,449426 |
| ERCC3 | Primary | CRISPRa | CD8 | IFNG | -0,1212 | 0,450881 |
| HOXB13 | Primary | CRISPRa | CD8 | IFNG | 0,12104 | 0,450881 |
| ZMAT4 | Primary | CRISPRa | CD8 | IFNG | 0,2188 | 0,450881 |
| ENC1 | Primary | CRISPRa | CD8 | IFNG | 0,21924 | 0,450881 |
| PLEK | Primary | CRISPRa | CD8 | IFNG | 0,25281 | 0,450881 |
| NFX1 | Primary | CRISPRa | CD8 | IFNG | 0,26291 | 0,450881 |
| RALGAPA1 | Primary | CRISPRa | CD8 | IFNG | 0,27542 | 0,450881 |
| PHRF1 | Primary | CRISPRa | CD8 | IFNG | 0,29171 | 0,450881 |
| SUPT20HL1 | Primary | CRISPRa | CD8 | IFNG | -0,31827 | 0,451369 |
| ZNF324 | Primary | CRISPRa | CD8 | IFNG | 0,15887 | 0,454023 |
| RNF187 | Primary | CRISPRa | CD8 | IFNG | 0,20416 | 0,454023 |
| SETX | Primary | CRISPRa | CD8 | IFNG | 0,26458 | 0,454023 |
| ZNF678 | Primary | CRISPRa | CD8 | IFNG | 0,30589 | 0,454949 |
| ZC3H13 | Primary | CRISPRa | CD8 | IFNG | -0,04673 | 0,455429 |
| CCAR1 | Primary | CRISPRa | CD8 | IFNG | 0,047511 | 0,457346 |
| DVL2 | Primary | CRISPRa | CD8 | IFNG | -0,12621 | 0,458446 |
| MET | Primary | CRISPRa | CD8 | IFNG | 0,059098 | 0,458446 |
| MTDH | Primary | CRISPRa | CD8 | IFNG | 0,10434 | 0,458446 |
| HDAC5 | Primary | CRISPRa | CD8 | IFNG | 0,15038 | 0,458446 |
| SEBOX | Primary | CRISPRa | CD8 | IFNG | 0,15652 | 0,458446 |
| ZNF211 | Primary | CRISPRa | CD8 | IFNG | 0,19681 | 0,458446 |
| DMTF1 | Primary | CRISPRa | CD8 | IFNG | 0,23296 | 0,458446 |
| ASF1B | Primary | CRISPRa | CD8 | IFNG | 0,26828 | 0,458446 |
| CBFB | Primary | CRISPRa | CD8 | IFNG | 0,28204 | 0,458446 |
| PML | Primary | CRISPRa | CD8 | IFNG | 0,35793 | 0,458446 |
| RAD21 | Primary | CRISPRa | CD8 | IFNG | 0,0057672 | 0,459136 |
| ZNF569 | Primary | CRISPRa | CD8 | IFNG | 0,17556 | 0,459922 |
| MITF | Primary | CRISPRa | CD8 | IFNG | -0,20304 | 0,460099 |
| ZNF804B | Primary | CRISPRa | CD8 | IFNG | 0,36115 | 0,461528 |
| ANP32D | Primary | CRISPRa | CD8 | IFNG | 0,086573 | 0,461572 |
| FAM200B | Primary | CRISPRa | CD8 | IFNG | 0,30381 | 0,461572 |
| RUNX2 | Primary | CRISPRa | CD8 | IFNG | 0,33153 | 0,461572 |
| EYA2 | Primary | CRISPRa | CD8 | IFNG | 0,029041 | 0,464256 |
| TFE3 | Primary | CRISPRa | CD8 | IFNG | 0,068925 | 0,464256 |
| ZNF524 | Primary | CRISPRa | CD8 | IFNG | 0,082498 | 0,464256 |
| BRWD3 | Primary | CRISPRa | CD8 | IFNG | 0,1592 | 0,464256 |
| RBBP7 | Primary | CRISPRa | CD8 | IFNG | 0,18798 | 0,464256 |
| PBX1 | Primary | CRISPRa | CD8 | IFNG | 0,29956 | 0,464256 |
| ILF2 | Primary | CRISPRa | CD8 | IFNG | 0,31354 | 0,464256 |
| GLI2 | Primary | CRISPRa | CD8 | IFNG | 0,35015 | 0,464256 |
| KDM7A | Primary | CRISPRa | CD8 | IFNG | 0,1082 | 0,465046 |
| HOXB2 | Primary | CRISPRa | CD8 | IFNG | 0,28236 | 0,466584 |
| KAT2A | Primary | CRISPRa | CD8 | IFNG | -0,095446 | 0,467505 |
| KLHL14 | Primary | CRISPRa | CD8 | IFNG | 0,0092398 | 0,467505 |
| ZBTB7A | Primary | CRISPRa | CD8 | IFNG | 0,12612 | 0,467505 |
| CFAP20 | Primary | CRISPRa | CD8 | IFNG | -0,05297 | 0,46846 |
| CAMTA1 | Primary | CRISPRa | CD8 | IFNG | 0,015983 | 0,46846 |
| EWSR1 | Primary | CRISPRa | CD8 | IFNG | 0,19158 | 0,46846 |
| LARP6 | Primary | CRISPRa | CD8 | IFNG | 0,2246 | 0,46846 |
| CCDC17 | Primary | CRISPRa | CD8 | IFNG | 0,23785 | 0,46846 |
| ADAR | Primary | CRISPRa | CD8 | IFNG | 0,25757 | 0,46846 |
| SERTAD2 | Primary | CRISPRa | CD8 | IFNG | 0,2762 | 0,46846 |
| DRGX | Primary | CRISPRa | CD8 | IFNG | 0,35002 | 0,46846 |
| BRD7 | Primary | CRISPRa | CD8 | IFNG | 0,0075062 | 0,469998 |
| FOXN4 | Primary | CRISPRa | CD8 | IFNG | 0,11584 | 0,469998 |
| ING1 | Primary | CRISPRa | CD8 | IFNG | 0,14846 | 0,469998 |
| MED12 | Primary | CRISPRa | CD8 | IFNG | 0,20973 | 0,469998 |
| HOXA6 | Primary | CRISPRa | CD8 | IFNG | 0,29751 | 0,469998 |
| ZC3H7B | Primary | CRISPRa | CD8 | IFNG | 0,30316 | 0,469998 |
| BCAS3 | Primary | CRISPRa | CD8 | IFNG | 0,31804 | 0,469998 |
| UBE2O | Primary | CRISPRa | CD8 | IFNG | 0,32288 | 0,469998 |
| SMAD3 | Primary | CRISPRa | CD8 | IFNG | -0,46809 | 0,470325 |
| TAL1 | Primary | CRISPRa | CD8 | IFNG | -0,37154 | 0,470325 |
| HNRNPA2B1 | Primary | CRISPRa | CD8 | IFNG | -0,35492 | 0,470325 |
| OAZ1 | Primary | CRISPRa | CD8 | IFNG | -0,35341 | 0,470325 |
| TBX15 | Primary | CRISPRa | CD8 | IFNG | -0,3396 | 0,470325 |
| ZNF705A | Primary | CRISPRa | CD8 | IFNG | -0,32999 | 0,470325 |
| ZNF267 | Primary | CRISPRa | CD8 | IFNG | -0,30253 | 0,470325 |
| RBMX | Primary | CRISPRa | CD8 | IFNG | -0,29294 | 0,470325 |
| ZNF784 | Primary | CRISPRa | CD8 | IFNG | -0,2561 | 0,470325 |
| ZNF777 | Primary | CRISPRa | CD8 | IFNG | -0,22584 | 0,470325 |
| PID1 | Primary | CRISPRa | CD8 | IFNG | -0,21224 | 0,470325 |
| TCEAL5 | Primary | CRISPRa | CD8 | IFNG | -0,19003 | 0,470325 |
| PDPK1 | Primary | CRISPRa | CD8 | IFNG | -0,023241 | 0,470325 |
| KCNH8 | Primary | CRISPRa | CD8 | IFNG | 0,016361 | 0,470325 |
| ETS2 | Primary | CRISPRa | CD8 | IFNG | 0,18455 | 0,470325 |
| ZNF786 | Primary | CRISPRa | CD8 | IFNG | 0,20239 | 0,470325 |
| HCFC1 | Primary | CRISPRa | CD8 | IFNG | -0,4326 | 0,471462 |
| XBP1 | Primary | CRISPRa | CD8 | IFNG | -0,40495 | 0,471462 |
| SETD7 | Primary | CRISPRa | CD8 | IFNG | -0,39345 | 0,471462 |
| DNMT3A | Primary | CRISPRa | CD8 | IFNG | -0,38255 | 0,471462 |
| SP1 | Primary | CRISPRa | CD8 | IFNG | -0,38155 | 0,471462 |
| TOB1 | Primary | CRISPRa | CD8 | IFNG | -0,31956 | 0,471462 |
| ZNF530 | Primary | CRISPRa | CD8 | IFNG | -0,29025 | 0,471462 |
| TCF7 | Primary | CRISPRa | CD8 | IFNG | -0,28095 | 0,471462 |
| CIC | Primary | CRISPRa | CD8 | IFNG | -0,27027 | 0,471462 |
| RRM2 | Primary | CRISPRa | CD8 | IFNG | -0,25786 | 0,471462 |
| PLK3 | Primary | CRISPRa | CD8 | IFNG | -0,25226 | 0,471462 |
| DBX2 | Primary | CRISPRa | CD8 | IFNG | -0,17116 | 0,471462 |
| ZSCAN1 | Primary | CRISPRa | CD8 | IFNG | -0,10625 | 0,471462 |
| PCBP4 | Primary | CRISPRa | CD8 | IFNG | -0,08034 | 0,471462 |
| WWP1 | Primary | CRISPRa | CD8 | IFNG | -0,074742 | 0,471462 |
| KDM3B | Primary | CRISPRa | CD8 | IFNG | -0,065727 | 0,471462 |
| ZFP41 | Primary | CRISPRa | CD8 | IFNG | 0,20545 | 0,472584 |
| ZC3H10 | Primary | CRISPRa | CD8 | IFNG | -0,2853 | 0,472845 |
| ZSCAN31 | Primary | CRISPRa | CD8 | IFNG | -0,25869 | 0,472845 |
| CPXCR1 | Primary | CRISPRa | CD8 | IFNG | -0,19248 | 0,472845 |
| SMARCA4 | Primary | CRISPRa | CD8 | IFNG | -0,15291 | 0,472845 |
| DEPDC5 | Primary | CRISPRa | CD8 | IFNG | -0,12886 | 0,472845 |
| EP400 | Primary | CRISPRa | CD8 | IFNG | -0,31131 | 0,473319 |
| PMS1 | Primary | CRISPRa | CD8 | IFNG | -0,29582 | 0,473319 |
| ZNF823 | Primary | CRISPRa | CD8 | IFNG | -0,29554 | 0,473319 |
| DPF1 | Primary | CRISPRa | CD8 | IFNG | -0,22382 | 0,473319 |
| PCBD2 | Primary | CRISPRa | CD8 | IFNG | -0,21811 | 0,473319 |
| MBD3L4 | Primary | CRISPRa | CD8 | IFNG | -0,15049 | 0,473319 |
| ZBTB21 | Primary | CRISPRa | CD8 | IFNG | -0,068127 | 0,473319 |
| CHD1 | Primary | CRISPRa | CD8 | IFNG | 0,0039554 | 0,473319 |
| CCAR2 | Primary | CRISPRa | CD8 | IFNG | 0,22312 | 0,473602 |
| MAML1 | Primary | CRISPRa | CD8 | IFNG | -0,13397 | 0,473645 |
| DTX1 | Primary | CRISPRa | CD8 | IFNG | 0,11366 | 0,473645 |
| TCF21 | Primary | CRISPRa | CD8 | IFNG | -0,094111 | 0,474151 |
| CDC45 | Primary | CRISPRa | CD8 | IFNG | -0,0014378 | 0,474151 |
| ZNF841 | Primary | CRISPRa | CD8 | IFNG | -0,14905 | 0,47417 |
| ZNF736 | Primary | CRISPRa | CD8 | IFNG | 0,34261 | 0,475182 |
| MAFB | Primary | CRISPRa | CD8 | IFNG | 0,2848 | 0,476621 |
| PIK3R2 | Primary | CRISPRa | CD8 | IFNG | 0,018808 | 0,477276 |
| ZNF558 | Primary | CRISPRa | CD8 | IFNG | 0,030424 | 0,477276 |
| KLF17 | Primary | CRISPRa | CD8 | IFNG | 0,16242 | 0,477276 |
| TSC22D1 | Primary | CRISPRa | CD8 | IFNG | 0,23363 | 0,477276 |
| BRCA2 | Primary | CRISPRa | CD8 | IFNG | 0,070781 | 0,477293 |
| ZNF800 | Primary | CRISPRa | CD8 | IFNG | -0,46955 | 0,477697 |
| PLXNC1 | Primary | CRISPRa | CD8 | IFNG | -0,25833 | 0,477697 |
| ZNF124 | Primary | CRISPRa | CD8 | IFNG | 0,1561 | 0,478483 |
| ZNF672 | Primary | CRISPRa | CD8 | IFNG | -0,41261 | 0,478936 |
| CASZ1 | Primary | CRISPRa | CD8 | IFNG | -0,30166 | 0,478936 |
| LEF1 | Primary | CRISPRa | CD8 | IFNG | -0,25696 | 0,478936 |
| MAPK10 | Primary | CRISPRa | CD8 | IFNG | -0,21039 | 0,478936 |
| MAGEA2 | Primary | CRISPRa | CD8 | IFNG | -0,18624 | 0,478936 |
| CAVIN1 | Primary | CRISPRa | CD8 | IFNG | -0,14471 | 0,478936 |
| ZSCAN2 | Primary | CRISPRa | CD8 | IFNG | -0,12684 | 0,478936 |
| PIM1 | Primary | CRISPRa | CD8 | IFNG | 0,060606 | 0,478936 |
| FGF1 | Primary | CRISPRa | CD8 | IFNG | 0,093435 | 0,478936 |
| LRRFIP1 | Primary | CRISPRa | CD8 | IFNG | -0,1156 | 0,479261 |
| ZNF891 | Primary | CRISPRa | CD8 | IFNG | 0,2081 | 0,479261 |
| CNOT11 | Primary | CRISPRa | CD8 | IFNG | -0,090471 | 0,481893 |
| CHD9 | Primary | CRISPRa | CD8 | IFNG | -0,017997 | 0,481893 |
| VSX1 | Primary | CRISPRa | CD8 | IFNG | -0,2118 | 0,482028 |
| EN2 | Primary | CRISPRa | CD8 | IFNG | -0,19937 | 0,482028 |
| HOXB6 | Primary | CRISPRa | CD8 | IFNG | -0,11164 | 0,482028 |
| BRWD1 | Primary | CRISPRa | CD8 | IFNG | -0,058842 | 0,482028 |
| TAF1D | Primary | CRISPRa | CD8 | IFNG | -0,047877 | 0,482028 |
| ZKSCAN8 | Primary | CRISPRa | CD8 | IFNG | 0,067599 | 0,482028 |
| ZNF670 | Primary | CRISPRa | CD8 | IFNG | 0,071326 | 0,482028 |
| TIGD1 | Primary | CRISPRa | CD8 | IFNG | 0,075677 | 0,482028 |
| ZMAT5 | Primary | CRISPRa | CD8 | IFNG | 0,077037 | 0,482028 |
| RARB | Primary | CRISPRa | CD8 | IFNG | 0,081674 | 0,482028 |
| FOXO4 | Primary | CRISPRa | CD8 | IFNG | 0,16362 | 0,482028 |
| RCBTB1 | Primary | CRISPRa | CD8 | IFNG | 0,16696 | 0,482028 |
| ZNF486 | Primary | CRISPRa | CD8 | IFNG | 0,18337 | 0,482028 |
| NOC4L | Primary | CRISPRa | CD8 | IFNG | 0,21841 | 0,482028 |
| MSANTD1 | Primary | CRISPRa | CD8 | IFNG | 0,24883 | 0,482028 |
| ZNF883 | Primary | CRISPRa | CD8 | IFNG | 0,28446 | 0,482028 |
| FLYWCH1 | Primary | CRISPRa | CD8 | IFNG | -0,3869 | 0,482108 |
| TFAP2D | Primary | CRISPRa | CD8 | IFNG | -0,31183 | 0,482108 |
| JDP2 | Primary | CRISPRa | CD8 | IFNG | -0,18763 | 0,482108 |
| ESR1 | Primary | CRISPRa | CD8 | IFNG | -0,10582 | 0,482108 |
| FHL3 | Primary | CRISPRa | CD8 | IFNG | 0,0080599 | 0,482108 |
| FOXD4 | Primary | CRISPRa | CD8 | IFNG | 0,35232 | 0,482216 |
| FAM189B | Primary | CRISPRa | CD8 | IFNG | -0,21897 | 0,483019 |
| ZNF14 | Primary | CRISPRa | CD8 | IFNG | -0,19878 | 0,483019 |
| PRAMEF5 | Primary | CRISPRa | CD8 | IFNG | -0,15181 | 0,483812 |
| TSHZ1 | Primary | CRISPRa | CD8 | IFNG | -0,2569 | 0,48397 |
| CHD2 | Primary | CRISPRa | CD8 | IFNG | -0,23287 | 0,48397 |
| ZNF852 | Primary | CRISPRa | CD8 | IFNG | -0,03985 | 0,48397 |
| INF2 | Primary | CRISPRa | CD8 | IFNG | 0,037205 | 0,48397 |
| ZNF280D | Primary | CRISPRa | CD8 | IFNG | 0,14305 | 0,48397 |
| ZNF517 | Primary | CRISPRa | CD8 | IFNG | 0,25001 | 0,48397 |
| SLC22A4 | Primary | CRISPRa | CD8 | IFNG | 0,26422 | 0,48397 |
| ZNF608 | Primary | CRISPRa | CD8 | IFNG | -0,30841 | 0,484449 |
| ZBTB41 | Primary | CRISPRa | CD8 | IFNG | 0,23881 | 0,484504 |
| BCOR | Primary | CRISPRa | CD8 | IFNG | -0,24237 | 0,485746 |
| PLXNA1 | Primary | CRISPRa | CD8 | IFNG | -0,2247 | 0,485746 |
| FBXL19 | Primary | CRISPRa | CD8 | IFNG | -0,21308 | 0,485746 |
| ZDHHC11 | Primary | CRISPRa | CD8 | IFNG | -0,1223 | 0,485746 |
| PREX2 | Primary | CRISPRa | CD8 | IFNG | -0,099534 | 0,485746 |
| PKNOX1 | Primary | CRISPRa | CD8 | IFNG | -0,066335 | 0,485746 |
| ZNF354C | Primary | CRISPRa | CD8 | IFNG | 0,017396 | 0,485746 |
| WNT8B | Primary | CRISPRa | CD8 | IFNG | 0,11204 | 0,485746 |
| GABPA | Primary | CRISPRa | CD8 | IFNG | 0,17148 | 0,485746 |
| ZNF674 | Primary | CRISPRa | CD8 | IFNG | -0,39425 | 0,488606 |
| MED1 | Primary | CRISPRa | CD8 | IFNG | -0,28062 | 0,488606 |
| NFKBIE | Primary | CRISPRa | CD8 | IFNG | -0,23427 | 0,488606 |
| SYK | Primary | CRISPRa | CD8 | IFNG | -0,18704 | 0,488606 |
| MAP2K5 | Primary | CRISPRa | CD8 | IFNG | -0,17771 | 0,488606 |
| ZNF624 | Primary | CRISPRa | CD8 | IFNG | -0,14133 | 0,488606 |
| MSX2 | Primary | CRISPRa | CD8 | IFNG | -0,13493 | 0,488606 |
| ZC3H11A | Primary | CRISPRa | CD8 | IFNG | -0,12024 | 0,488606 |
| ZNF3 | Primary | CRISPRa | CD8 | IFNG | 0,075862 | 0,488606 |
| TRIM16 | Primary | CRISPRa | CD8 | IFNG | 0,080451 | 0,491424 |
| ZNF408 | Primary | CRISPRa | CD8 | IFNG | -0,19803 | 0,493283 |
| TOP1MT | Primary | CRISPRa | CD8 | IFNG | -0,26864 | 0,496187 |
| NKX3-2 | Primary | CRISPRa | CD8 | IFNG | -0,20664 | 0,496187 |
| DHX33 | Primary | CRISPRa | CD8 | IFNG | -0,1531 | 0,496187 |
| BANP | Primary | CRISPRa | CD8 | IFNG | -0,073747 | 0,496187 |
| TEAD4 | Primary | CRISPRa | CD8 | IFNG | 0,025398 | 0,496187 |
| SCRT2 | Primary | CRISPRa | CD8 | IFNG | 0,17862 | 0,496187 |
| TCP10L | Primary | CRISPRa | CD8 | IFNG | -0,37534 | 0,496299 |
| GMCL1 | Primary | CRISPRa | CD8 | IFNG | -0,24059 | 0,496348 |
| ZNF536 | Primary | CRISPRa | CD8 | IFNG | -0,034141 | 0,496348 |
| MYBL2 | Primary | CRISPRa | CD8 | IFNG | -0,41115 | 0,497013 |
| VGLL4 | Primary | CRISPRa | CD8 | IFNG | -0,3492 | 0,497013 |
| KIAA1549 | Primary | CRISPRa | CD8 | IFNG | 0,0017472 | 0,497013 |
| CTCF | Primary | CRISPRa | CD8 | IFNG | 0,07441 | 0,497013 |
| TIPARP | Primary | CRISPRa | CD8 | IFNG | -0,31301 | 0,499512 |
| PPP2R1A | Primary | CRISPRa | CD8 | IFNG | -0,28983 | 0,499512 |
| MORF4L2 | Primary | CRISPRa | CD8 | IFNG | -0,28822 | 0,499512 |
| CTR9 | Primary | CRISPRa | CD8 | IFNG | -0,27061 | 0,499512 |
| FOXC2 | Primary | CRISPRa | CD8 | IFNG | -0,25875 | 0,499512 |
| ZNF518B | Primary | CRISPRa | CD8 | IFNG | -0,10592 | 0,499704 |
| MAPK8 | Primary | CRISPRa | CD8 | IFNG | -0,038965 | 0,503129 |
| SMAD1 | Primary | CRISPRa | CD8 | IFNG | -0,21274 | 0,503398 |
| SOX8 | Primary | CRISPRa | CD8 | IFNG | -0,071971 | 0,503398 |
| ZNF619 | Primary | CRISPRa | CD8 | IFNG | -0,37312 | 0,505499 |
| DMRTA2 | Primary | CRISPRa | CD8 | IFNG | -0,35756 | 0,505499 |
| PPP3CB | Primary | CRISPRa | CD8 | IFNG | -0,22366 | 0,505499 |
| GATAD1 | Primary | CRISPRa | CD8 | IFNG | -0,38618 | 0,505676 |
| AIRE | Primary | CRISPRa | CD8 | IFNG | 0,091231 | 0,505676 |
| STK3 | Primary | CRISPRa | CD8 | IFNG | -0,29546 | 0,50633 |
| TRIP11 | Primary | CRISPRa | CD8 | IFNG | -0,27919 | 0,50633 |
| CBX5 | Primary | CRISPRa | CD8 | IFNG | 0,16091 | 0,506482 |
| ZNF326 | Primary | CRISPRa | CD8 | IFNG | 0,17612 | 0,506482 |
| OVOL1 | Primary | CRISPRa | CD8 | IFNG | 0,29939 | 0,506482 |
| HSPA1B | Primary | CRISPRa | CD8 | IFNG | 0,19414 | 0,506814 |
| SUPT3H | Primary | CRISPRa | CD8 | IFNG | 0,045677 | 0,507241 |
| ZNF385C | Primary | CRISPRa | CD8 | IFNG | 0,049774 | 0,507241 |
| WBP2 | Primary | CRISPRa | CD8 | IFNG | -0,3221 | 0,507555 |
| UBE2K | Primary | CRISPRa | CD8 | IFNG | -0,0038408 | 0,508886 |
| CBX6 | Primary | CRISPRa | CD8 | IFNG | 0,029789 | 0,508886 |
| WNT4 | Primary | CRISPRa | CD8 | IFNG | 0,14595 | 0,508886 |
| HMX2 | Primary | CRISPRa | CD8 | IFNG | 0,3143 | 0,508886 |
| MAPK1 | Primary | CRISPRa | CD8 | IFNG | 0,32671 | 0,508886 |
| YLPM1 | Primary | CRISPRa | CD8 | IFNG | -0,072108 | 0,50894 |
| UBE2V1 | Primary | CRISPRa | CD8 | IFNG | 0,0032292 | 0,50894 |
| TRIP10 | Primary | CRISPRa | CD8 | IFNG | 0,055831 | 0,50894 |
| CSRP1 | Primary | CRISPRa | CD8 | IFNG | 0,25444 | 0,50894 |
| NRBF2 | Primary | CRISPRa | CD8 | IFNG | 0,26389 | 0,50894 |
| ESRRA | Primary | CRISPRa | CD8 | IFNG | 0,23466 | 0,511271 |
| SP140L | Primary | CRISPRa | CD8 | IFNG | 0,23799 | 0,511271 |
| PPP2CA | Primary | CRISPRa | CD8 | IFNG | 0,27437 | 0,511523 |
| DHX9 | Primary | CRISPRa | CD8 | IFNG | -0,18062 | 0,511641 |
| ASH1L | Primary | CRISPRa | CD8 | IFNG | 0,18028 | 0,511641 |
| ZNF443 | Primary | CRISPRa | CD8 | IFNG | 0,15966 | 0,511865 |
| ID2 | Primary | CRISPRa | CD8 | IFNG | -0,00034338 | 0,51188 |
| CC2D1A | Primary | CRISPRa | CD8 | IFNG | 0,121 | 0,51188 |
| SCAND1 | Primary | CRISPRa | CD8 | IFNG | 0,16194 | 0,51188 |
| ZNF441 | Primary | CRISPRa | CD8 | IFNG | 0,20591 | 0,51188 |
| CHMP3 | Primary | CRISPRa | CD8 | IFNG | 0,30012 | 0,51188 |
| MED22 | Primary | CRISPRa | CD8 | IFNG | 0,10827 | 0,517106 |
| EZH2 | Primary | CRISPRa | CD8 | IFNG | -0,42866 | 0,517469 |
| PRR3 | Primary | CRISPRa | CD8 | IFNG | -0,28492 | 0,517469 |
| SCMH1 | Primary | CRISPRa | CD8 | IFNG | -0,25274 | 0,517469 |
| NIPBL | Primary | CRISPRa | CD8 | IFNG | -0,24532 | 0,517469 |
| RGS6 | Primary | CRISPRa | CD8 | IFNG | -0,17248 | 0,517469 |
| ZFP28 | Primary | CRISPRa | CD8 | IFNG | -0,16127 | 0,517469 |
| ZNF385B | Primary | CRISPRa | CD8 | IFNG | -0,13606 | 0,517469 |
| TRAPPC2 | Primary | CRISPRa | CD8 | IFNG | 0,0073869 | 0,517469 |
| RBSN | Primary | CRISPRa | CD8 | IFNG | 0,064427 | 0,517469 |
| ELF5 | Primary | CRISPRa | CD8 | IFNG | 0,08004 | 0,517469 |
| ZSCAN30 | Primary | CRISPRa | CD8 | IFNG | 0,1047 | 0,517469 |
| POU1F1 | Primary | CRISPRa | CD8 | IFNG | 0,22724 | 0,517964 |
| KLF8 | Primary | CRISPRa | CD8 | IFNG | 0,31438 | 0,517964 |
| HOXD1 | Primary | CRISPRa | CD8 | IFNG | -0,3685 | 0,518285 |
| SOX2 | Primary | CRISPRa | CD8 | IFNG | -0,33612 | 0,518285 |
| METTL3 | Primary | CRISPRa | CD8 | IFNG | -0,28656 | 0,518285 |
| SALL4 | Primary | CRISPRa | CD8 | IFNG | -0,25793 | 0,518285 |
| GTF2H3 | Primary | CRISPRa | CD8 | IFNG | -0,25292 | 0,518285 |
| ZIM2 | Primary | CRISPRa | CD8 | IFNG | -0,22056 | 0,518285 |
| TBPL1 | Primary | CRISPRa | CD8 | IFNG | -0,17177 | 0,518285 |
| ZFP92 | Primary | CRISPRa | CD8 | IFNG | -0,04885 | 0,518285 |
| MNAT1 | Primary | CRISPRa | CD8 | IFNG | 0,0052186 | 0,518285 |
| ZNF814 | Primary | CRISPRa | CD8 | IFNG | 0,15588 | 0,518285 |
| ZNF347 | Primary | CRISPRa | CD8 | IFNG | -0,31142 | 0,518623 |
| MECOM | Primary | CRISPRa | CD8 | IFNG | -0,2664 | 0,518623 |
| FOXP1 | Primary | CRISPRa | CD8 | IFNG | -0,13644 | 0,518623 |
| ETV4 | Primary | CRISPRa | CD8 | IFNG | -0,13561 | 0,518623 |
| ZNF773 | Primary | CRISPRa | CD8 | IFNG | -0,0067848 | 0,518623 |
| MLLT1 | Primary | CRISPRa | CD8 | IFNG | -0,10895 | 0,52033 |
| FLI1 | Primary | CRISPRa | CD8 | IFNG | 0,024167 | 0,52033 |
| THAP5 | Primary | CRISPRa | CD8 | IFNG | 0,077257 | 0,52033 |
| PKIG | Primary | CRISPRa | CD8 | IFNG | 0,15576 | 0,52033 |
| ARGFX | Primary | CRISPRa | CD8 | IFNG | 0,29677 | 0,52033 |
| GATA1 | Primary | CRISPRa | CD8 | IFNG | -0,39774 | 0,520652 |
| ZNF439 | Primary | CRISPRa | CD8 | IFNG | -0,38882 | 0,520652 |
| DPPA4 | Primary | CRISPRa | CD8 | IFNG | -0,36055 | 0,520652 |
| NEUROD2 | Primary | CRISPRa | CD8 | IFNG | -0,34153 | 0,520652 |
| MIXL1 | Primary | CRISPRa | CD8 | IFNG | -0,32133 | 0,520652 |
| MTF1 | Primary | CRISPRa | CD8 | IFNG | -0,32028 | 0,520652 |
| NKX2-2 | Primary | CRISPRa | CD8 | IFNG | -0,3115 | 0,520652 |
| SREBF1 | Primary | CRISPRa | CD8 | IFNG | -0,29721 | 0,520652 |
| UIMC1 | Primary | CRISPRa | CD8 | IFNG | -0,29674 | 0,520652 |
| GATA2 | Primary | CRISPRa | CD8 | IFNG | -0,29631 | 0,520652 |
| TFAP2C | Primary | CRISPRa | CD8 | IFNG | -0,27694 | 0,520652 |
| FHOD1 | Primary | CRISPRa | CD8 | IFNG | -0,26284 | 0,520652 |
| ALX3 | Primary | CRISPRa | CD8 | IFNG | -0,26239 | 0,520652 |
| KDM4A | Primary | CRISPRa | CD8 | IFNG | -0,25398 | 0,520652 |
| ZNF468 | Primary | CRISPRa | CD8 | IFNG | -0,23953 | 0,520652 |
| CDK9 | Primary | CRISPRa | CD8 | IFNG | -0,23186 | 0,520652 |
| PRR12 | Primary | CRISPRa | CD8 | IFNG | -0,22575 | 0,520652 |
| TPRX1 | Primary | CRISPRa | CD8 | IFNG | -0,16673 | 0,520652 |
| ZNF501 | Primary | CRISPRa | CD8 | IFNG | -0,16149 | 0,520652 |
| HOXD4 | Primary | CRISPRa | CD8 | IFNG | -0,14721 | 0,520652 |
| CIZ1 | Primary | CRISPRa | CD8 | IFNG | -0,14606 | 0,520652 |
| ZNF550 | Primary | CRISPRa | CD8 | IFNG | -0,12998 | 0,520652 |
| ZFAND5 | Primary | CRISPRa | CD8 | IFNG | -0,11245 | 0,520652 |
| ZSCAN23 | Primary | CRISPRa | CD8 | IFNG | -0,088885 | 0,520652 |
| ZNF275 | Primary | CRISPRa | CD8 | IFNG | -0,084158 | 0,520652 |
| JARID2 | Primary | CRISPRa | CD8 | IFNG | -0,080307 | 0,520652 |
| NKX6-1 | Primary | CRISPRa | CD8 | IFNG | -0,059476 | 0,520652 |
| PRAMEF19 | Primary | CRISPRa | CD8 | IFNG | -0,054734 | 0,520652 |
| TFEB | Primary | CRISPRa | CD8 | IFNG | -0,033297 | 0,520652 |
| THAP4 | Primary | CRISPRa | CD8 | IFNG | -0,016443 | 0,520652 |
| TRAK1 | Primary | CRISPRa | CD8 | IFNG | -0,016113 | 0,520652 |
| LARP4 | Primary | CRISPRa | CD8 | IFNG | -0,0095565 | 0,520652 |
| PAF1 | Primary | CRISPRa | CD8 | IFNG | 0,0017757 | 0,520652 |
| YBX3 | Primary | CRISPRa | CD8 | IFNG | 0,055876 | 0,520652 |
| SSBP2 | Primary | CRISPRa | CD8 | IFNG | 0,057517 | 0,520652 |
| ZFP69 | Primary | CRISPRa | CD8 | IFNG | 0,15273 | 0,520652 |
| RNF111 | Primary | CRISPRa | CD8 | IFNG | -0,040149 | 0,521199 |
| KLF12 | Primary | CRISPRa | CD8 | IFNG | 0,02262 | 0,521199 |
| EEF1A1 | Primary | CRISPRa | CD8 | IFNG | -0,14785 | 0,521764 |
| AATF | Primary | CRISPRa | CD8 | IFNG | -0,086357 | 0,521764 |
| CASP8AP2 | Primary | CRISPRa | CD8 | IFNG | 0,12461 | 0,521764 |
| NFRKB | Primary | CRISPRa | CD8 | IFNG | -0,044109 | 0,522293 |
| PBXIP1 | Primary | CRISPRa | CD8 | IFNG | 0,031703 | 0,522493 |
| AURKAIP1 | Primary | CRISPRa | CD8 | IFNG | 0,31846 | 0,523516 |
| FBXW11 | Primary | CRISPRa | CD8 | IFNG | -0,34019 | 0,524627 |
| SUPT20H | Primary | CRISPRa | CD8 | IFNG | -0,3156 | 0,524627 |
| ZMAT2 | Primary | CRISPRa | CD8 | IFNG | -0,25811 | 0,524627 |
| DENND4A | Primary | CRISPRa | CD8 | IFNG | -0,24708 | 0,524627 |
| U2AF1 | Primary | CRISPRa | CD8 | IFNG | -0,23171 | 0,524627 |
| RPRD1B | Primary | CRISPRa | CD8 | IFNG | -0,22828 | 0,524627 |
| PYGO2 | Primary | CRISPRa | CD8 | IFNG | -0,22462 | 0,524627 |
| KDM4C | Primary | CRISPRa | CD8 | IFNG | -0,21285 | 0,524627 |
| ZC3H6 | Primary | CRISPRa | CD8 | IFNG | -0,20515 | 0,524627 |
| SALL1 | Primary | CRISPRa | CD8 | IFNG | -0,1615 | 0,524627 |
| ITGB3BP | Primary | CRISPRa | CD8 | IFNG | -0,13001 | 0,524627 |
| BAZ2B | Primary | CRISPRa | CD8 | IFNG | -0,0811 | 0,524627 |
| WNT3A | Primary | CRISPRa | CD8 | IFNG | -0,068214 | 0,524627 |
| TOX4 | Primary | CRISPRa | CD8 | IFNG | 0,05334 | 0,524627 |
| TCF15 | Primary | CRISPRa | CD8 | IFNG | -0,086899 | 0,526616 |
| SIVA1 | Primary | CRISPRa | CD8 | IFNG | 0,14909 | 0,527589 |
| RPTOR | Primary | CRISPRa | CD8 | IFNG | 0,10358 | 0,528442 |
| MYBL1 | Primary | CRISPRa | CD8 | IFNG | -0,25821 | 0,528832 |
| ZBTB26 | Primary | CRISPRa | CD8 | IFNG | -0,14043 | 0,528832 |
| FOS | Primary | CRISPRa | CD8 | IFNG | 0,24265 | 0,528852 |
| TRIM23 | Primary | CRISPRa | CD8 | IFNG | 0,15909 | 0,529003 |
| PFDN5 | Primary | CRISPRa | CD8 | IFNG | 0,18056 | 0,529003 |
| RCOR2 | Primary | CRISPRa | CD8 | IFNG | 0,066649 | 0,529685 |
| KHDRBS1 | Primary | CRISPRa | CD8 | IFNG | -0,15029 | 0,529898 |
| EPC2 | Primary | CRISPRa | CD8 | IFNG | 0,074085 | 0,530247 |
| GTF2H1 | Primary | CRISPRa | CD8 | IFNG | 0,20357 | 0,530247 |
| CALR | Primary | CRISPRa | CD8 | IFNG | -0,030297 | 0,530711 |
| ZBP1 | Primary | CRISPRa | CD8 | IFNG | -0,12953 | 0,53243 |
| JPH2 | Primary | CRISPRa | CD8 | IFNG | -0,23777 | 0,532813 |
| DCAF1 | Primary | CRISPRa | CD8 | IFNG | -0,12185 | 0,532813 |
| RBPJ | Primary | CRISPRa | CD8 | IFNG | -0,28968 | 0,53294 |
| ONECUT1 | Primary | CRISPRa | CD8 | IFNG | -0,23818 | 0,53294 |
| FOXO3 | Primary | CRISPRa | CD8 | IFNG | 0,054956 | 0,53294 |
| PRDM7 | Primary | CRISPRa | CD8 | IFNG | 0,21457 | 0,53294 |
| TAF4 | Primary | CRISPRa | CD8 | IFNG | -0,08658 | 0,535136 |
| MAFA | Primary | CRISPRa | CD8 | IFNG | -0,080249 | 0,535136 |
| RUVBL1 | Primary | CRISPRa | CD8 | IFNG | -0,011649 | 0,535136 |
| NUP133 | Primary | CRISPRa | CD8 | IFNG | 0,02332 | 0,535136 |
| ZSCAN9 | Primary | CRISPRa | CD8 | IFNG | 0,082207 | 0,535136 |
| LOXL3 | Primary | CRISPRa | CD8 | IFNG | 0,09799 | 0,535136 |
| ZBED6 | Primary | CRISPRa | CD8 | IFNG | 0,17217 | 0,535136 |
| ARRB1 | Primary | CRISPRa | CD8 | IFNG | 0,20827 | 0,535136 |
| KDM5C | Primary | CRISPRa | CD8 | IFNG | 0,2309 | 0,535136 |
| ZNF705B | Primary | CRISPRa | CD8 | IFNG | 0,23481 | 0,535136 |
| LGALS9 | Primary | CRISPRa | CD8 | IFNG | -0,17876 | 0,535715 |
| MLXIPL | Primary | CRISPRa | CD8 | IFNG | -0,16793 | 0,535715 |
| SKIDA1 | Primary | CRISPRa | CD8 | IFNG | -0,084274 | 0,535715 |
| CNPY3 | Primary | CRISPRa | CD8 | IFNG | -0,0064903 | 0,535715 |
| HPCAL1 | Primary | CRISPRa | CD8 | IFNG | -0,0042368 | 0,535715 |
| LCORL | Primary | CRISPRa | CD8 | IFNG | 0,074879 | 0,535715 |
| ZBTB8A | Primary | CRISPRa | CD8 | IFNG | 0,08339 | 0,535715 |
| HEYL | Primary | CRISPRa | CD8 | IFNG | 0,12892 | 0,535715 |
| PLAGL2 | Primary | CRISPRa | CD8 | IFNG | 0,13146 | 0,535715 |
| ZNF791 | Primary | CRISPRa | CD8 | IFNG | 0,15491 | 0,535715 |
| KHSRP | Primary | CRISPRa | CD8 | IFNG | 0,18434 | 0,535715 |
| TCEAL1 | Primary | CRISPRa | CD8 | IFNG | -0,2309 | 0,536772 |
| HYAL2 | Primary | CRISPRa | CD8 | IFNG | -0,055906 | 0,536772 |
| SAP30 | Primary | CRISPRa | CD8 | IFNG | 0,12564 | 0,536772 |
| C1D | Primary | CRISPRa | CD8 | IFNG | 0,13457 | 0,536772 |
| JUP | Primary | CRISPRa | CD8 | IFNG | 0,2113 | 0,536772 |
| MIS18BP1 | Primary | CRISPRa | CD8 | IFNG | 0,24147 | 0,536772 |
| PPARD | Primary | CRISPRa | CD8 | IFNG | 0,27317 | 0,536772 |
| FBP1 | Primary | CRISPRa | CD8 | IFNG | 0,28421 | 0,536772 |
| PADI2 | Primary | CRISPRa | CD8 | IFNG | 0,30273 | 0,536772 |
| ZNF547 | Primary | CRISPRa | CD8 | IFNG | -0,22105 | 0,537211 |
| YBX2 | Primary | CRISPRa | CD8 | IFNG | -0,081714 | 0,537211 |
| ZNF334 | Primary | CRISPRa | CD8 | IFNG | -0,18295 | 0,537421 |
| KAT5 | Primary | CRISPRa | CD8 | IFNG | -0,14193 | 0,537421 |
| SP4 | Primary | CRISPRa | CD8 | IFNG | 0,043441 | 0,537887 |
| ZNF526 | Primary | CRISPRa | CD8 | IFNG | 0,023933 | 0,53853 |
| ID1 | Primary | CRISPRa | CD8 | IFNG | 0,091072 | 0,53853 |
| NFE2L2 | Primary | CRISPRa | CD8 | IFNG | 0,1601 | 0,53853 |
| MAML3 | Primary | CRISPRa | CD8 | IFNG | -0,19997 | 0,539307 |
| HOXA11 | Primary | CRISPRa | CD8 | IFNG | -0,18432 | 0,539307 |
| IKBKB | Primary | CRISPRa | CD8 | IFNG | 0,2319 | 0,539307 |
| TRPS1 | Primary | CRISPRa | CD8 | IFNG | 0,24005 | 0,539307 |
| HESX1 | Primary | CRISPRa | CD8 | IFNG | 0,24362 | 0,539307 |
| RASD1 | Primary | CRISPRa | CD8 | IFNG | 0,25103 | 0,539307 |
| CARHSP1 | Primary | CRISPRa | CD8 | IFNG | 0,10647 | 0,541397 |
| RBL2 | Primary | CRISPRa | CD8 | IFNG | -0,057709 | 0,541533 |
| SOX17 | Primary | CRISPRa | CD8 | IFNG | -0,052805 | 0,541533 |
| FIGLA | Primary | CRISPRa | CD8 | IFNG | -0,028479 | 0,541533 |
| CREM | Primary | CRISPRa | CD8 | IFNG | 0,0094798 | 0,541533 |
| TDG | Primary | CRISPRa | CD8 | IFNG | 0,013016 | 0,541533 |
| SRA1 | Primary | CRISPRa | CD8 | IFNG | 0,031201 | 0,541533 |
| NUPR1 | Primary | CRISPRa | CD8 | IFNG | 0,049314 | 0,541533 |
| ZNF300 | Primary | CRISPRa | CD8 | IFNG | 0,067516 | 0,541533 |
| BRF1 | Primary | CRISPRa | CD8 | IFNG | 0,073461 | 0,541533 |
| SUPT16H | Primary | CRISPRa | CD8 | IFNG | 0,16319 | 0,541533 |
| POU3F1 | Primary | CRISPRa | CD8 | IFNG | 0,1783 | 0,541533 |
| NOBOX | Primary | CRISPRa | CD8 | IFNG | 0,21449 | 0,541533 |
| ZNF611 | Primary | CRISPRa | CD8 | IFNG | 0,21533 | 0,541533 |
| KEAP1 | Primary | CRISPRa | CD8 | IFNG | 0,23886 | 0,541533 |
| LDOC1 | Primary | CRISPRa | CD8 | IFNG | 0,25562 | 0,541533 |
| LIN9 | Primary | CRISPRa | CD8 | IFNG | 0,26047 | 0,541533 |
| ZFPM2 | Primary | CRISPRa | CD8 | IFNG | 0,2833 | 0,541533 |
| HELZ | Primary | CRISPRa | CD8 | IFNG | 0,31087 | 0,541533 |
| GMEB1 | Primary | CRISPRa | CD8 | IFNG | 0,35282 | 0,541533 |
| ZNF668 | Primary | CRISPRa | CD8 | IFNG | 0,39259 | 0,541533 |
| BIRC2 | Primary | CRISPRa | CD8 | IFNG | 0,14465 | 0,542812 |
| SORBS3 | Primary | CRISPRa | CD8 | IFNG | -0,024173 | 0,545552 |
| FGF2 | Primary | CRISPRa | CD8 | IFNG | 0,24148 | 0,545552 |
| CTBP2 | Primary | CRISPRa | CD8 | IFNG | 0,10318 | 0,54895 |
| ZNF681 | Primary | CRISPRa | CD8 | IFNG | 0,14365 | 0,54895 |
| HOXA10 | Primary | CRISPRa | CD8 | IFNG | 0,18892 | 0,54895 |
| ZNF655 | Primary | CRISPRa | CD8 | IFNG | -0,09997 | 0,549449 |
| POLR3C | Primary | CRISPRa | CD8 | IFNG | 0,13524 | 0,549449 |
| MAK | Primary | CRISPRa | CD8 | IFNG | 0,27261 | 0,549449 |
| MAP2K3 | Primary | CRISPRa | CD8 | IFNG | -0,092547 | 0,55066 |
| ZNF236 | Primary | CRISPRa | CD8 | IFNG | 0,18485 | 0,55066 |
| DUSP12 | Primary | CRISPRa | CD8 | IFNG | 0,27906 | 0,551044 |
| ZSCAN5A | Primary | CRISPRa | CD8 | IFNG | 0,037676 | 0,551195 |
| TAB1 | Primary | CRISPRa | CD8 | IFNG | 0,1962 | 0,551828 |
| FEV | Primary | CRISPRa | CD8 | IFNG | -0,11338 | 0,55325 |
| ZFP37 | Primary | CRISPRa | CD8 | IFNG | 0,029837 | 0,55325 |
| ACTL6A | Primary | CRISPRa | CD8 | IFNG | 0,10475 | 0,55325 |
| SF1 | Primary | CRISPRa | CD8 | IFNG | 0,23668 | 0,55325 |
| FOXI2 | Primary | CRISPRa | CD8 | IFNG | -0,39172 | 0,553858 |
| ZNF329 | Primary | CRISPRa | CD8 | IFNG | -0,36184 | 0,553858 |
| DNTTIP1 | Primary | CRISPRa | CD8 | IFNG | -0,33895 | 0,553858 |
| USP34 | Primary | CRISPRa | CD8 | IFNG | -0,31264 | 0,553858 |
| LANCL2 | Primary | CRISPRa | CD8 | IFNG | -0,24125 | 0,553858 |
| LTN1 | Primary | CRISPRa | CD8 | IFNG | -0,18589 | 0,553858 |
| CNOT3 | Primary | CRISPRa | CD8 | IFNG | -0,18227 | 0,553858 |
| SSBP4 | Primary | CRISPRa | CD8 | IFNG | -0,12869 | 0,553858 |
| BARHL2 | Primary | CRISPRa | CD8 | IFNG | 0,038925 | 0,553858 |
| TSC22D2 | Primary | CRISPRa | CD8 | IFNG | -0,0071824 | 0,554442 |
| ZNF444 | Primary | CRISPRa | CD8 | IFNG | -0,26804 | 0,554844 |
| ZPR1 | Primary | CRISPRa | CD8 | IFNG | 0,03369 | 0,554844 |
| ANKRA2 | Primary | CRISPRa | CD8 | IFNG | 0,084683 | 0,554844 |
| CITED2 | Primary | CRISPRa | CD8 | IFNG | 0,21499 | 0,554844 |
| ZNF430 | Primary | CRISPRa | CD8 | IFNG | 0,29952 | 0,554844 |
| APEX1 | Primary | CRISPRa | CD8 | IFNG | 0,078804 | 0,555673 |
| DNMT3B | Primary | CRISPRa | CD8 | IFNG | 0,080054 | 0,555673 |
| CBX4 | Primary | CRISPRa | CD8 | IFNG | 0,18095 | 0,555673 |
| CDIP1 | Primary | CRISPRa | CD8 | IFNG | 0,068516 | 0,556499 |
| MYEF2 | Primary | CRISPRa | CD8 | IFNG | 0,06606 | 0,556711 |
| TOX2 | Primary | CRISPRa | CD8 | IFNG | -0,090532 | 0,557514 |
| TRIM52 | Primary | CRISPRa | CD8 | IFNG | 0,10153 | 0,557514 |
| GCFC2 | Primary | CRISPRa | CD8 | IFNG | 0,15252 | 0,557514 |
| TET3 | Primary | CRISPRa | CD8 | IFNG | -0,36117 | 0,557922 |
| SUMO2 | Primary | CRISPRa | CD8 | IFNG | -0,2225 | 0,557922 |
| ZNF865 | Primary | CRISPRa | CD8 | IFNG | -0,21214 | 0,557922 |
| GLIS3 | Primary | CRISPRa | CD8 | IFNG | -0,12472 | 0,557922 |
| ZNF511 | Primary | CRISPRa | CD8 | IFNG | -0,040201 | 0,557922 |
| CPEB1 | Primary | CRISPRa | CD8 | IFNG | -0,21696 | 0,558536 |
| ZNF99 | Primary | CRISPRa | CD8 | IFNG | -0,10397 | 0,558536 |
| AP5Z1 | Primary | CRISPRa | CD8 | IFNG | -0,28915 | 0,558835 |
| TP53INP1 | Primary | CRISPRa | CD8 | IFNG | -0,15747 | 0,558835 |
| DDX54 | Primary | CRISPRa | CD8 | IFNG | -0,12579 | 0,558835 |
| SIX3 | Primary | CRISPRa | CD8 | IFNG | -0,10827 | 0,558835 |
| MAPK7 | Primary | CRISPRa | CD8 | IFNG | -0,037491 | 0,558835 |
| BRD2 | Primary | CRISPRa | CD8 | IFNG | 0,044977 | 0,558835 |
| SMARCB1 | Primary | CRISPRa | CD8 | IFNG | 0,050882 | 0,558835 |
| THAP3 | Primary | CRISPRa | CD8 | IFNG | 0,11158 | 0,558835 |
| HDAC3 | Primary | CRISPRa | CD8 | IFNG | 0,12186 | 0,558835 |
| SSX1 | Primary | CRISPRa | CD8 | IFNG | 0,1498 | 0,558835 |
| ZNF398 | Primary | CRISPRa | CD8 | IFNG | 0,18091 | 0,558835 |
| ZNF829 | Primary | CRISPRa | CD8 | IFNG | 0,28359 | 0,558835 |
| PRDM9 | Primary | CRISPRa | CD8 | IFNG | -0,20607 | 0,559871 |
| ZFP69B | Primary | CRISPRa | CD8 | IFNG | -0,073077 | 0,559871 |
| TGFB1 | Primary | CRISPRa | CD8 | IFNG | 0,031147 | 0,559871 |
| ZBTB16 | Primary | CRISPRa | CD8 | IFNG | 0,043286 | 0,559871 |
| PINK1 | Primary | CRISPRa | CD8 | IFNG | 0,18583 | 0,559871 |
| CRX | Primary | CRISPRa | CD8 | IFNG | -0,014289 | 0,561333 |
| COMMD8 | Primary | CRISPRa | CD8 | IFNG | 0,22221 | 0,561333 |
| DYRK1A | Primary | CRISPRa | CD8 | IFNG | 0,25827 | 0,561333 |
| ZBTB46 | Primary | CRISPRa | CD8 | IFNG | -0,17006 | 0,561443 |
| ZNF90 | Primary | CRISPRa | CD8 | IFNG | 0,1729 | 0,561443 |
| HNRNPCL2 | Primary | CRISPRa | CD8 | IFNG | 0,3532 | 0,561821 |
| KDM8 | Primary | CRISPRa | CD8 | IFNG | -0,24958 | 0,572051 |
| ZNF726 | Primary | CRISPRa | CD8 | IFNG | -0,2495 | 0,572051 |
| TAF1C | Primary | CRISPRa | CD8 | IFNG | -0,20707 | 0,572051 |
| ZNF860 | Primary | CRISPRa | CD8 | IFNG | -0,15776 | 0,572051 |
| TFAP2B | Primary | CRISPRa | CD8 | IFNG | -0,042112 | 0,572051 |
| SKIL | Primary | CRISPRa | CD8 | IFNG | 0,1707 | 0,574905 |
| WDR77 | Primary | CRISPRa | CD8 | IFNG | -0,10135 | 0,575963 |
| PPID | Primary | CRISPRa | CD8 | IFNG | 0,098855 | 0,575963 |
| ANAPC2 | Primary | CRISPRa | CD8 | IFNG | 0,12444 | 0,575963 |
| CEBPE | Primary | CRISPRa | CD8 | IFNG | -0,17219 | 0,576308 |
| CRTC1 | Primary | CRISPRa | CD8 | IFNG | -0,16139 | 0,576308 |
| RNF114 | Primary | CRISPRa | CD8 | IFNG | -0,11989 | 0,576308 |
| ZNF138 | Primary | CRISPRa | CD8 | IFNG | -0,10938 | 0,576308 |
| JAK2 | Primary | CRISPRa | CD8 | IFNG | -0,066352 | 0,576308 |
| FOXB2 | Primary | CRISPRa | CD8 | IFNG | 0,057522 | 0,576308 |
| ARID4A | Primary | CRISPRa | CD8 | IFNG | 0,14607 | 0,576308 |
| AFF2 | Primary | CRISPRa | CD8 | IFNG | 0,26308 | 0,576308 |
| CRK | Primary | CRISPRa | CD8 | IFNG | 0,27669 | 0,576308 |
| RNF25 | Primary | CRISPRa | CD8 | IFNG | -0,28849 | 0,57647 |
| DBP | Primary | CRISPRa | CD8 | IFNG | 0,20552 | 0,57647 |
| HOXC10 | Primary | CRISPRa | CD8 | IFNG | -0,36018 | 0,576857 |
| POU3F4 | Primary | CRISPRa | CD8 | IFNG | -0,32124 | 0,576857 |
| ZNF449 | Primary | CRISPRa | CD8 | IFNG | 0,069634 | 0,576857 |
| TERB1 | Primary | CRISPRa | CD8 | IFNG | 0,14097 | 0,578408 |
| ZNF302 | Primary | CRISPRa | CD8 | IFNG | 0,10539 | 0,578763 |
| CENPB | Primary | CRISPRa | CD8 | IFNG | -0,079127 | 0,579149 |
| C20orf194 | Primary | CRISPRa | CD8 | IFNG | 0,15967 | 0,579288 |
| LENG9 | Primary | CRISPRa | CD8 | IFNG | -0,3797 | 0,579765 |
| DPPA2 | Primary | CRISPRa | CD8 | IFNG | -0,12233 | 0,579765 |
| BOLA3 | Primary | CRISPRa | CD8 | IFNG | -0,33897 | 0,580623 |
| SLTM | Primary | CRISPRa | CD8 | IFNG | -0,23526 | 0,580623 |
| MED31 | Primary | CRISPRa | CD8 | IFNG | -0,37465 | 0,582389 |
| PROX1 | Primary | CRISPRa | CD8 | IFNG | -0,23274 | 0,582389 |
| DHX57 | Primary | CRISPRa | CD8 | IFNG | -0,19592 | 0,582389 |
| TUB | Primary | CRISPRa | CD8 | IFNG | -0,077437 | 0,582389 |
| POU5F1 | Primary | CRISPRa | CD8 | IFNG | -0,060405 | 0,582389 |
| ZNF607 | Primary | CRISPRa | CD8 | IFNG | -0,032441 | 0,582389 |
| SLC2A4RG | Primary | CRISPRa | CD8 | IFNG | 0,0061823 | 0,582389 |
| HOXA7 | Primary | CRISPRa | CD8 | IFNG | 0,012592 | 0,582389 |
| MTA1 | Primary | CRISPRa | CD8 | IFNG | 0,042765 | 0,582389 |
| ZNF836 | Primary | CRISPRa | CD8 | IFNG | 0,061044 | 0,582389 |
| XRCC6 | Primary | CRISPRa | CD8 | IFNG | 0,095676 | 0,582389 |
| HOXB7 | Primary | CRISPRa | CD8 | IFNG | 0,10509 | 0,582389 |
| VPS36 | Primary | CRISPRa | CD8 | IFNG | 0,12699 | 0,582389 |
| ING4 | Primary | CRISPRa | CD8 | IFNG | 0,14239 | 0,582389 |
| TRIB3 | Primary | CRISPRa | CD8 | IFNG | 0,14435 | 0,582389 |
| PBRM1 | Primary | CRISPRa | CD8 | IFNG | 0,14764 | 0,582389 |
| ZFPM1 | Primary | CRISPRa | CD8 | IFNG | 0,15631 | 0,582389 |
| ZNF235 | Primary | CRISPRa | CD8 | IFNG | 0,2836 | 0,582389 |
| CHD1L | Primary | CRISPRa | CD8 | IFNG | -0,082265 | 0,583219 |
| FUBP3 | Primary | CRISPRa | CD8 | IFNG | 0,042509 | 0,583219 |
| RERE | Primary | CRISPRa | CD8 | IFNG | 0,089081 | 0,583219 |
| ZC3H14 | Primary | CRISPRa | CD8 | IFNG | 0,18347 | 0,583219 |
| FANCD2 | Primary | CRISPRa | CD8 | IFNG | 0,12659 | 0,583383 |
| TLE1 | Primary | CRISPRa | CD8 | IFNG | -0,086722 | 0,585347 |
| POGK | Primary | CRISPRa | CD8 | IFNG | -0,1727 | 0,585551 |
| ZIM3 | Primary | CRISPRa | CD8 | IFNG | -0,37476 | 0,586112 |
| ZNRF1 | Primary | CRISPRa | CD8 | IFNG | -0,36057 | 0,586112 |
| CTH | Primary | CRISPRa | CD8 | IFNG | -0,35759 | 0,586112 |
| ZNF703 | Primary | CRISPRa | CD8 | IFNG | -0,33675 | 0,586112 |
| ZNF93 | Primary | CRISPRa | CD8 | IFNG | -0,31986 | 0,586112 |
| NACC2 | Primary | CRISPRa | CD8 | IFNG | -0,29271 | 0,586112 |
| HDGF | Primary | CRISPRa | CD8 | IFNG | -0,28864 | 0,586112 |
| ZNF682 | Primary | CRISPRa | CD8 | IFNG | -0,25459 | 0,586112 |
| FAM83G | Primary | CRISPRa | CD8 | IFNG | -0,25216 | 0,586112 |
| MSX1 | Primary | CRISPRa | CD8 | IFNG | -0,22937 | 0,586112 |
| YBX1 | Primary | CRISPRa | CD8 | IFNG | -0,21476 | 0,586112 |
| NFKBIB | Primary | CRISPRa | CD8 | IFNG | -0,19055 | 0,586112 |
| DMRT1 | Primary | CRISPRa | CD8 | IFNG | -0,16769 | 0,586112 |
| E2F1 | Primary | CRISPRa | CD8 | IFNG | -0,16732 | 0,586112 |
| RNF24 | Primary | CRISPRa | CD8 | IFNG | -0,15746 | 0,586112 |
| TEAD3 | Primary | CRISPRa | CD8 | IFNG | -0,15035 | 0,586112 |
| ISL1 | Primary | CRISPRa | CD8 | IFNG | -0,14182 | 0,586112 |
| NFIB | Primary | CRISPRa | CD8 | IFNG | -0,13993 | 0,586112 |
| IRF7 | Primary | CRISPRa | CD8 | IFNG | -0,13245 | 0,586112 |
| UBE3A | Primary | CRISPRa | CD8 | IFNG | -0,13111 | 0,586112 |
| ZNF649 | Primary | CRISPRa | CD8 | IFNG | -0,12083 | 0,586112 |
| IRF5 | Primary | CRISPRa | CD8 | IFNG | -0,11547 | 0,586112 |
| TRIM50 | Primary | CRISPRa | CD8 | IFNG | -0,08294 | 0,586112 |
| ZNF260 | Primary | CRISPRa | CD8 | IFNG | -0,079326 | 0,586112 |
| BTG1 | Primary | CRISPRa | CD8 | IFNG | -0,068743 | 0,586112 |
| DNAJA3 | Primary | CRISPRa | CD8 | IFNG | -0,065421 | 0,586112 |
| SNAI1 | Primary | CRISPRa | CD8 | IFNG | -0,060374 | 0,586112 |
| ZFX | Primary | CRISPRa | CD8 | IFNG | -0,04879 | 0,586112 |
| ZNF341 | Primary | CRISPRa | CD8 | IFNG | -0,040474 | 0,586112 |
| LMCD1 | Primary | CRISPRa | CD8 | IFNG | -0,030053 | 0,586112 |
| FOXR1 | Primary | CRISPRa | CD8 | IFNG | -0,015482 | 0,586112 |
| CDK5 | Primary | CRISPRa | CD8 | IFNG | 0,031363 | 0,586112 |
| ARID4B | Primary | CRISPRa | CD8 | IFNG | 0,049887 | 0,586112 |
| ZNF345 | Primary | CRISPRa | CD8 | IFNG | 0,052319 | 0,586112 |
| ZFYVE26 | Primary | CRISPRa | CD8 | IFNG | 0,061842 | 0,586112 |
| ZNF32 | Primary | CRISPRa | CD8 | IFNG | 0,10268 | 0,586112 |
| ANP32A | Primary | CRISPRa | CD8 | IFNG | 0,11924 | 0,586112 |
| TP63 | Primary | CRISPRa | CD8 | IFNG | -0,25393 | 0,586183 |
| PCBP1 | Primary | CRISPRa | CD8 | IFNG | -0,27014 | 0,587547 |
| TAF12 | Primary | CRISPRa | CD8 | IFNG | -0,16257 | 0,587547 |
| GTF2I | Primary | CRISPRa | CD8 | IFNG | -0,18019 | 0,58774 |
| TFAP2E | Primary | CRISPRa | CD8 | IFNG | -0,07678 | 0,58774 |
| ZNF23 | Primary | CRISPRa | CD8 | IFNG | -0,076385 | 0,58774 |
| MED6 | Primary | CRISPRa | CD8 | IFNG | -0,038612 | 0,589077 |
| NANOG | Primary | CRISPRa | CD8 | IFNG | -0,090273 | 0,590694 |
| SGSM2 | Primary | CRISPRa | CD8 | IFNG | -0,34804 | 0,593194 |
| PRAMEF10 | Primary | CRISPRa | CD8 | IFNG | -0,49642 | 0,59437 |
| ANKRD49 | Primary | CRISPRa | CD8 | IFNG | -0,092626 | 0,596838 |
| TGFB1I1 | Primary | CRISPRa | CD8 | IFNG | -0,20248 | 0,597163 |
| GLIS1 | Primary | CRISPRa | CD8 | IFNG | -0,097528 | 0,597163 |
| HAT1 | Primary | CRISPRa | CD8 | IFNG | -0,041903 | 0,597163 |
| HOXB9 | Primary | CRISPRa | CD8 | IFNG | 0,017963 | 0,597163 |
| PDCD4 | Primary | CRISPRa | CD8 | IFNG | 0,019443 | 0,597163 |
| ZBTB33 | Primary | CRISPRa | CD8 | IFNG | 0,029904 | 0,597163 |
| ZNF221 | Primary | CRISPRa | CD8 | IFNG | 0,049946 | 0,597163 |
| ADNP | Primary | CRISPRa | CD8 | IFNG | 0,12251 | 0,597163 |
| ZNF563 | Primary | CRISPRa | CD8 | IFNG | 0,12277 | 0,597163 |
| MXI1 | Primary | CRISPRa | CD8 | IFNG | 0,13935 | 0,597163 |
| RBM39 | Primary | CRISPRa | CD8 | IFNG | 0,20286 | 0,597163 |
| PARP15 | Primary | CRISPRa | CD8 | IFNG | 0,23869 | 0,597163 |
| DEPDC1 | Primary | CRISPRa | CD8 | IFNG | -0,058395 | 0,600092 |
| ZNF541 | Primary | CRISPRa | CD8 | IFNG | 0,015652 | 0,601305 |
| HPCA | Primary | CRISPRa | CD8 | IFNG | -0,32679 | 0,602228 |
| NOTCH4 | Primary | CRISPRa | CD8 | IFNG | -0,28639 | 0,602228 |
| MYRF | Primary | CRISPRa | CD8 | IFNG | -0,012566 | 0,602228 |
| ZNF107 | Primary | CRISPRa | CD8 | IFNG | 0,018047 | 0,602228 |
| KANK1 | Primary | CRISPRa | CD8 | IFNG | 0,042722 | 0,602228 |
| LZTR1 | Primary | CRISPRa | CD8 | IFNG | 0,07657 | 0,602228 |
| VPS25 | Primary | CRISPRa | CD8 | IFNG | 0,079162 | 0,602228 |
| NCOA1 | Primary | CRISPRa | CD8 | IFNG | 0,079522 | 0,602228 |
| PHF14 | Primary | CRISPRa | CD8 | IFNG | 0,095001 | 0,602228 |
| ZBTB38 | Primary | CRISPRa | CD8 | IFNG | 0,13666 | 0,602228 |
| ZNF879 | Primary | CRISPRa | CD8 | IFNG | 0,13997 | 0,602228 |
| MEOX2 | Primary | CRISPRa | CD8 | IFNG | 0,15362 | 0,602228 |
| LHX8 | Primary | CRISPRa | CD8 | IFNG | 0,2096 | 0,602228 |
| MEF2C | Primary | CRISPRa | CD8 | IFNG | 0,21237 | 0,602228 |
| BOLA2 | Primary | CRISPRa | CD8 | IFNG | 0,25838 | 0,602228 |
| VAX1 | Primary | CRISPRa | CD8 | IFNG | 0,2715 | 0,602228 |
| SP9 | Primary | CRISPRa | CD8 | IFNG | 0,27881 | 0,602228 |
| ERF | Primary | CRISPRa | CD8 | IFNG | 0,30982 | 0,602228 |
| MGA | Primary | CRISPRa | CD8 | IFNG | 0,25067 | 0,603229 |
| MCRS1 | Primary | CRISPRa | CD8 | IFNG | -0,30724 | 0,603486 |
| ZNF223 | Primary | CRISPRa | CD8 | IFNG | -0,161 | 0,603486 |
| RIOX2 | Primary | CRISPRa | CD8 | IFNG | -0,1392 | 0,603486 |
| ZNF664 | Primary | CRISPRa | CD8 | IFNG | -0,017515 | 0,603486 |
| MED25 | Primary | CRISPRa | CD8 | IFNG | -0,0049746 | 0,603486 |
| ZSCAN25 | Primary | CRISPRa | CD8 | IFNG | -0,0049439 | 0,603486 |
| TWIST2 | Primary | CRISPRa | CD8 | IFNG | 0,01407 | 0,603486 |
| ATAD2 | Primary | CRISPRa | CD8 | IFNG | 0,05111 | 0,603486 |
| PRAMEF17 | Primary | CRISPRa | CD8 | IFNG | 0,068065 | 0,603486 |
| NFIA | Primary | CRISPRa | CD8 | IFNG | 0,088224 | 0,603486 |
| FOXK2 | Primary | CRISPRa | CD8 | IFNG | 0,10197 | 0,603486 |
| CENPX | Primary | CRISPRa | CD8 | IFNG | 0,16731 | 0,603486 |
| ZNF358 | Primary | CRISPRa | CD8 | IFNG | 0,25952 | 0,603486 |
| TAF9B | Primary | CRISPRa | CD8 | IFNG | 0,27334 | 0,603486 |
| MTA2 | Primary | CRISPRa | CD8 | IFNG | 0,31192 | 0,603486 |
| TSNAX | Primary | CRISPRa | CD8 | IFNG | -0,24874 | 0,604191 |
| SUV39H1 | Primary | CRISPRa | CD8 | IFNG | -0,21676 | 0,604191 |
| TACC1 | Primary | CRISPRa | CD8 | IFNG | -0,12862 | 0,604191 |
| PRKCZ | Primary | CRISPRa | CD8 | IFNG | -0,12755 | 0,604191 |
| UBP1 | Primary | CRISPRa | CD8 | IFNG | -0,12353 | 0,604191 |
| ZNF782 | Primary | CRISPRa | CD8 | IFNG | 0,028959 | 0,604191 |
| HDAC8 | Primary | CRISPRa | CD8 | IFNG | 0,16819 | 0,604267 |
| SS18L2 | Primary | CRISPRa | CD8 | IFNG | 0,28884 | 0,604896 |
| ZNF750 | Primary | CRISPRa | CD8 | IFNG | -0,30508 | 0,607402 |
| CENPBD1 | Primary | CRISPRa | CD8 | IFNG | -0,30449 | 0,607402 |
| ZNF775 | Primary | CRISPRa | CD8 | IFNG | -0,28843 | 0,607402 |
| GTF2A2 | Primary | CRISPRa | CD8 | IFNG | -0,28566 | 0,607402 |
| NR1H4 | Primary | CRISPRa | CD8 | IFNG | -0,27439 | 0,607402 |
| PKD1 | Primary | CRISPRa | CD8 | IFNG | -0,26547 | 0,607402 |
| RRN3 | Primary | CRISPRa | CD8 | IFNG | -0,26368 | 0,607402 |
| SCML2 | Primary | CRISPRa | CD8 | IFNG | -0,2635 | 0,607402 |
| BARHL1 | Primary | CRISPRa | CD8 | IFNG | -0,25224 | 0,607402 |
| TSPYL2 | Primary | CRISPRa | CD8 | IFNG | -0,23703 | 0,607402 |
| GNL3 | Primary | CRISPRa | CD8 | IFNG | -0,20533 | 0,607402 |
| JADE1 | Primary | CRISPRa | CD8 | IFNG | -0,2012 | 0,607402 |
| DNMT3L | Primary | CRISPRa | CD8 | IFNG | -0,19866 | 0,607402 |
| NEUROD4 | Primary | CRISPRa | CD8 | IFNG | -0,19039 | 0,607402 |
| DNMT1 | Primary | CRISPRa | CD8 | IFNG | -0,17944 | 0,607402 |
| USE1 | Primary | CRISPRa | CD8 | IFNG | -0,16717 | 0,607402 |
| KLF1 | Primary | CRISPRa | CD8 | IFNG | -0,15323 | 0,607402 |
| FOXN2 | Primary | CRISPRa | CD8 | IFNG | -0,14438 | 0,607402 |
| MEIS1 | Primary | CRISPRa | CD8 | IFNG | -0,12288 | 0,607402 |
| TSHZ2 | Primary | CRISPRa | CD8 | IFNG | -0,11943 | 0,607402 |
| KDM5A | Primary | CRISPRa | CD8 | IFNG | -0,10497 | 0,607402 |
| PER2 | Primary | CRISPRa | CD8 | IFNG | -0,092832 | 0,607402 |
| CREB3L2 | Primary | CRISPRa | CD8 | IFNG | -0,089296 | 0,607402 |
| RARG | Primary | CRISPRa | CD8 | IFNG | -0,08818 | 0,607402 |
| POLR3F | Primary | CRISPRa | CD8 | IFNG | -0,087988 | 0,607402 |
| PTTG1IP | Primary | CRISPRa | CD8 | IFNG | -0,084442 | 0,607402 |
| CHCHD3 | Primary | CRISPRa | CD8 | IFNG | -0,083728 | 0,607402 |
| GZF1 | Primary | CRISPRa | CD8 | IFNG | -0,075327 | 0,607402 |
| SSH3 | Primary | CRISPRa | CD8 | IFNG | -0,062147 | 0,607402 |
| NR2E3 | Primary | CRISPRa | CD8 | IFNG | -0,051381 | 0,607402 |
| ZNF704 | Primary | CRISPRa | CD8 | IFNG | -0,041482 | 0,607402 |
| PHC1 | Primary | CRISPRa | CD8 | IFNG | -0,03209 | 0,607402 |
| ZNF792 | Primary | CRISPRa | CD8 | IFNG | -0,021598 | 0,607402 |
| USP7 | Primary | CRISPRa | CD8 | IFNG | -0,020145 | 0,607402 |
| KMT2C | Primary | CRISPRa | CD8 | IFNG | -0,013707 | 0,607402 |
| VPS72 | Primary | CRISPRa | CD8 | IFNG | -0,010021 | 0,607402 |
| ZNF862 | Primary | CRISPRa | CD8 | IFNG | 0,016306 | 0,607402 |
| ZNF598 | Primary | CRISPRa | CD8 | IFNG | 0,032532 | 0,607402 |
| NFE2L1 | Primary | CRISPRa | CD8 | IFNG | 0,046087 | 0,607402 |
| LGALS12 | Primary | CRISPRa | CD8 | IFNG | 0,052175 | 0,607402 |
| ZNF436 | Primary | CRISPRa | CD8 | IFNG | 0,060373 | 0,607402 |
| ZNF354B | Primary | CRISPRa | CD8 | IFNG | 0,07081 | 0,607402 |
| ZNF705D | Primary | CRISPRa | CD8 | IFNG | 0,072426 | 0,607402 |
| LARP4B | Primary | CRISPRa | CD8 | IFNG | 0,094499 | 0,607402 |
| PRAMEF27 | Primary | CRISPRa | CD8 | IFNG | -0,37036 | 0,609935 |
| ZNF189 | Primary | CRISPRa | CD8 | IFNG | -0,2381 | 0,611663 |
| CUL4B | Primary | CRISPRa | CD8 | IFNG | -0,048818 | 0,61505 |
| CCNH | Primary | CRISPRa | CD8 | IFNG | 0,067114 | 0,61505 |
| ZNF765 | Primary | CRISPRa | CD8 | IFNG | 0,087911 | 0,61505 |
| DPRX | Primary | CRISPRa | CD8 | IFNG | 0,16898 | 0,61505 |
| SERTAD3 | Primary | CRISPRa | CD8 | IFNG | 0,24575 | 0,61505 |
| SMAD2 | Primary | CRISPRa | CD8 | IFNG | -0,16794 | 0,616351 |
| TENM2 | Primary | CRISPRa | CD8 | IFNG | -0,11895 | 0,616351 |
| ACTN4 | Primary | CRISPRa | CD8 | IFNG | 0,14595 | 0,616709 |
| CNOT1 | Primary | CRISPRa | CD8 | IFNG | -0,32923 | 0,617154 |
| KRBA2 | Primary | CRISPRa | CD8 | IFNG | -0,23994 | 0,617154 |
| CPSF4L | Primary | CRISPRa | CD8 | IFNG | -0,20013 | 0,617154 |
| ZNF652 | Primary | CRISPRa | CD8 | IFNG | -0,11283 | 0,617154 |
| NME2 | Primary | CRISPRa | CD8 | IFNG | -0,041484 | 0,617154 |
| TBX5 | Primary | CRISPRa | CD8 | IFNG | -0,0041201 | 0,617154 |
| ARID5B | Primary | CRISPRa | CD8 | IFNG | -0,0032277 | 0,617154 |
| ALX4 | Primary | CRISPRa | CD8 | IFNG | 0,13488 | 0,617154 |
| ZSCAN22 | Primary | CRISPRa | CD8 | IFNG | 0,14335 | 0,617154 |
| SSX7 | Primary | CRISPRa | CD8 | IFNG | 0,15693 | 0,617154 |
| LMO2 | Primary | CRISPRa | CD8 | IFNG | 0,18642 | 0,617154 |
| ZNF410 | Primary | CRISPRa | CD8 | IFNG | 0,19291 | 0,617154 |
| BOLA1 | Primary | CRISPRa | CD8 | IFNG | 0,21069 | 0,617154 |
| SHOX2 | Primary | CRISPRa | CD8 | IFNG | 0,21592 | 0,617154 |
| C1QBP | Primary | CRISPRa | CD8 | IFNG | 0,22657 | 0,617154 |
| NCL | Primary | CRISPRa | CD8 | IFNG | 0,25558 | 0,617154 |
| ELF2 | Primary | CRISPRa | CD8 | IFNG | 0,28734 | 0,617154 |
| CCDC124 | Primary | CRISPRa | CD8 | IFNG | 0,31847 | 0,617154 |
| RBM10 | Primary | CRISPRa | CD8 | IFNG | -0,31663 | 0,617699 |
| PLXNB3 | Primary | CRISPRa | CD8 | IFNG | 0,008854 | 0,617699 |
| ZNF623 | Primary | CRISPRa | CD8 | IFNG | 0,04746 | 0,617699 |
| NKAP | Primary | CRISPRa | CD8 | IFNG | -0,31033 | 0,617788 |
| UBC | Primary | CRISPRa | CD8 | IFNG | -0,23572 | 0,617788 |
| WBP2NL | Primary | CRISPRa | CD8 | IFNG | 0,10956 | 0,623212 |
| TLE3 | Primary | CRISPRa | CD8 | IFNG | -0,20276 | 0,62415 |
| INO80C | Primary | CRISPRa | CD8 | IFNG | 0,081359 | 0,62415 |
| LRPPRC | Primary | CRISPRa | CD8 | IFNG | 0,15383 | 0,625578 |
| TRIM48 | Primary | CRISPRa | CD8 | IFNG | -0,16776 | 0,62738 |
| ZNF446 | Primary | CRISPRa | CD8 | IFNG | 0,21731 | 0,62917 |
| MEN1 | Primary | CRISPRa | CD8 | IFNG | -0,21824 | 0,63048 |
| TAF5L | Primary | CRISPRa | CD8 | IFNG | -0,052702 | 0,63048 |
| SRY | Primary | CRISPRa | CD8 | IFNG | -0,047565 | 0,63048 |
| TRIM41 | Primary | CRISPRa | CD8 | IFNG | 0,012468 | 0,63048 |
| IL16 | Primary | CRISPRa | CD8 | IFNG | 0,12052 | 0,63048 |
| PRAMEF2 | Primary | CRISPRa | CD8 | IFNG | 0,26754 | 0,63048 |
| EGR4 | Primary | CRISPRa | CD8 | IFNG | 0,070359 | 0,630722 |
| AKAP9 | Primary | CRISPRa | CD8 | IFNG | 0,07684 | 0,631669 |
| DTX2 | Primary | CRISPRa | CD8 | IFNG | 0,11537 | 0,631669 |
| CERS5 | Primary | CRISPRa | CD8 | IFNG | 0,29686 | 0,631669 |
| TRIM10 | Primary | CRISPRa | CD8 | IFNG | -0,27794 | 0,632428 |
| EZH1 | Primary | CRISPRa | CD8 | IFNG | -0,020566 | 0,632428 |
| DNAJC1 | Primary | CRISPRa | CD8 | IFNG | -0,28782 | 0,632559 |
| DMRTC2 | Primary | CRISPRa | CD8 | IFNG | 0,091751 | 0,632559 |
| HES5 | Primary | CRISPRa | CD8 | IFNG | -0,18121 | 0,642423 |
| EME2 | Primary | CRISPRa | CD8 | IFNG | 0,084634 | 0,642423 |
| TAF9 | Primary | CRISPRa | CD8 | IFNG | 0,30862 | 0,642423 |
| CREB3L3 | Primary | CRISPRa | CD8 | IFNG | -0,36981 | 0,642607 |
| IRAK1 | Primary | CRISPRa | CD8 | IFNG | -0,3398 | 0,642607 |
| NFIL3 | Primary | CRISPRa | CD8 | IFNG | -0,32924 | 0,642607 |
| KLF13 | Primary | CRISPRa | CD8 | IFNG | -0,30512 | 0,642607 |
| HMGA1 | Primary | CRISPRa | CD8 | IFNG | -0,19373 | 0,642607 |
| ERCC2 | Primary | CRISPRa | CD8 | IFNG | -0,070289 | 0,642607 |
| LCOR | Primary | CRISPRa | CD8 | IFNG | 0,029407 | 0,642607 |
| MBNL2 | Primary | CRISPRa | CD8 | IFNG | -0,0038916 | 0,643029 |
| YEATS2 | Primary | CRISPRa | CD8 | IFNG | 0,026926 | 0,643029 |
| CREBBP | Primary | CRISPRa | CD8 | IFNG | 0,093326 | 0,643029 |
| RELB | Primary | CRISPRa | CD8 | IFNG | 0,24422 | 0,643029 |
| OTX2 | Primary | CRISPRa | CD8 | IFNG | -0,20946 | 0,645884 |
| ZNF467 | Primary | CRISPRa | CD8 | IFNG | -0,1762 | 0,645884 |
| PQBP1 | Primary | CRISPRa | CD8 | IFNG | -0,096154 | 0,645884 |
| TRIM38 | Primary | CRISPRa | CD8 | IFNG | 0,12829 | 0,647218 |
| POU5F1B | Primary | CRISPRa | CD8 | IFNG | -0,36662 | 0,647418 |
| OGT | Primary | CRISPRa | CD8 | IFNG | -0,33937 | 0,647418 |
| PAX7 | Primary | CRISPRa | CD8 | IFNG | -0,23034 | 0,647418 |
| MTERF4 | Primary | CRISPRa | CD8 | IFNG | 0,063737 | 0,647418 |
| EDF1 | Primary | CRISPRa | CD8 | IFNG | 0,1677 | 0,647418 |
| MED9 | Primary | CRISPRa | CD8 | IFNG | -0,26987 | 0,647767 |
| HES7 | Primary | CRISPRa | CD8 | IFNG | -0,22206 | 0,647767 |
| ZNF451 | Primary | CRISPRa | CD8 | IFNG | -0,10786 | 0,647767 |
| ZMIZ2 | Primary | CRISPRa | CD8 | IFNG | 0,021816 | 0,647767 |
| DYNLL1 | Primary | CRISPRa | CD8 | IFNG | -0,19767 | 0,648357 |
| LPIN2 | Primary | CRISPRa | CD8 | IFNG | 0,082887 | 0,648499 |
| SLC26A10 | Primary | CRISPRa | CD8 | IFNG | 0,30909 | 0,648736 |
| URI1 | Primary | CRISPRa | CD8 | IFNG | -0,10963 | 0,6502 |
| ZNF677 | Primary | CRISPRa | CD8 | IFNG | -0,035479 | 0,6502 |
| OSR1 | Primary | CRISPRa | CD8 | IFNG | 0,02379 | 0,6502 |
| CBL | Primary | CRISPRa | CD8 | IFNG | 0,050674 | 0,6502 |
| VENTX | Primary | CRISPRa | CD8 | IFNG | 0,057142 | 0,6502 |
| PKM | Primary | CRISPRa | CD8 | IFNG | 0,1395 | 0,6502 |
| ZNF414 | Primary | CRISPRa | CD8 | IFNG | 0,14968 | 0,6502 |
| PITX3 | Primary | CRISPRa | CD8 | IFNG | 0,18265 | 0,6502 |
| ZNF671 | Primary | CRISPRa | CD8 | IFNG | -0,047451 | 0,650255 |
| OVOL2 | Primary | CRISPRa | CD8 | IFNG | 0,020094 | 0,650255 |
| ZNF557 | Primary | CRISPRa | CD8 | IFNG | 0,16465 | 0,650255 |
| EDRF1 | Primary | CRISPRa | CD8 | IFNG | 0,29311 | 0,650255 |
| PCGF2 | Primary | CRISPRa | CD8 | IFNG | -0,043388 | 0,650461 |
| RAN | Primary | CRISPRa | CD8 | IFNG | 0,16411 | 0,650967 |
| ADAMTS17 | Primary | CRISPRa | CD8 | IFNG | -0,24655 | 0,653938 |
| ESRRB | Primary | CRISPRa | CD8 | IFNG | -0,23092 | 0,653938 |
| RAPGEF5 | Primary | CRISPRa | CD8 | IFNG | -0,19799 | 0,653938 |
| NHLH1 | Primary | CRISPRa | CD8 | IFNG | -0,1664 | 0,653938 |
| TIGD3 | Primary | CRISPRa | CD8 | IFNG | -0,09567 | 0,653938 |
| HOXB1 | Primary | CRISPRa | CD8 | IFNG | -0,087618 | 0,653938 |
| DNM2 | Primary | CRISPRa | CD8 | IFNG | -0,082128 | 0,653938 |
| UBE2B | Primary | CRISPRa | CD8 | IFNG | -0,037048 | 0,653938 |
| RBM15B | Primary | CRISPRa | CD8 | IFNG | 0,020621 | 0,653938 |
| ZFP14 | Primary | CRISPRa | CD8 | IFNG | 0,022219 | 0,653938 |
| DNAJB5 | Primary | CRISPRa | CD8 | IFNG | 0,03114 | 0,653938 |
| HDAC11 | Primary | CRISPRa | CD8 | IFNG | 0,052666 | 0,653938 |
| SIAH2 | Primary | CRISPRa | CD8 | IFNG | 0,099013 | 0,653938 |
| ATXN7L3 | Primary | CRISPRa | CD8 | IFNG | 0,11 | 0,653938 |
| ZNF317 | Primary | CRISPRa | CD8 | IFNG | 0,1113 | 0,653938 |
| GRIP1 | Primary | CRISPRa | CD8 | IFNG | 0,13011 | 0,653938 |
| ZNF709 | Primary | CRISPRa | CD8 | IFNG | 0,13052 | 0,653938 |
| CCNK | Primary | CRISPRa | CD8 | IFNG | 0,13561 | 0,653938 |
| TCERG1L | Primary | CRISPRa | CD8 | IFNG | 0,15392 | 0,653938 |
| FOXM1 | Primary | CRISPRa | CD8 | IFNG | 0,15885 | 0,653938 |
| SMARCA1 | Primary | CRISPRa | CD8 | IFNG | 0,16966 | 0,653938 |
| TRIM32 | Primary | CRISPRa | CD8 | IFNG | 0,1742 | 0,653938 |
| ZFHX4 | Primary | CRISPRa | CD8 | IFNG | 0,18316 | 0,653938 |
| RIT2 | Primary | CRISPRa | CD8 | IFNG | 0,23421 | 0,653938 |
| HHEX | Primary | CRISPRa | CD8 | IFNG | 0,25372 | 0,653938 |
| SP140 | Primary | CRISPRa | CD8 | IFNG | 0,25632 | 0,653938 |
| HSF2 | Primary | CRISPRa | CD8 | IFNG | 0,26516 | 0,653938 |
| FEZF1 | Primary | CRISPRa | CD8 | IFNG | 0,29271 | 0,653938 |
| GSC2 | Primary | CRISPRa | CD8 | IFNG | 0,29651 | 0,653938 |
| KCNIP2 | Primary | CRISPRa | CD8 | IFNG | 0,3488 | 0,653938 |
| NR2F6 | Primary | CRISPRa | CD8 | IFNG | 0,0032901 | 0,654548 |
| ZNF586 | Primary | CRISPRa | CD8 | IFNG | 0,12715 | 0,654548 |
| DSP | Primary | CRISPRa | CD8 | IFNG | 0,30116 | 0,654548 |
| CUL2 | Primary | CRISPRa | CD8 | IFNG | -0,01166 | 0,654922 |
| MYT1L | Primary | CRISPRa | CD8 | IFNG | 0,092315 | 0,654922 |
| RC3H2 | Primary | CRISPRa | CD8 | IFNG | 0,18418 | 0,654922 |
| LIN54 | Primary | CRISPRa | CD8 | IFNG | -0,12109 | 0,655806 |
| FBN1 | Primary | CRISPRa | CD8 | IFNG | -0,07535 | 0,655806 |
| WDHD1 | Primary | CRISPRa | CD8 | IFNG | -0,055821 | 0,655806 |
| FOXJ2 | Primary | CRISPRa | CD8 | IFNG | 0,038369 | 0,655806 |
| JUND | Primary | CRISPRa | CD8 | IFNG | 0,34186 | 0,655806 |
| MATR3 | Primary | CRISPRa | CD8 | IFNG | 0,37844 | 0,655806 |
| ZNF707 | Primary | CRISPRa | CD8 | IFNG | -0,37212 | 0,656823 |
| ZFAND6 | Primary | CRISPRa | CD8 | IFNG | -0,33888 | 0,656823 |
| MESP1 | Primary | CRISPRa | CD8 | IFNG | -0,31735 | 0,656823 |
| MYF5 | Primary | CRISPRa | CD8 | IFNG | -0,29584 | 0,656823 |
| ZNF845 | Primary | CRISPRa | CD8 | IFNG | -0,28532 | 0,656823 |
| TOX | Primary | CRISPRa | CD8 | IFNG | -0,28158 | 0,656823 |
| ELL | Primary | CRISPRa | CD8 | IFNG | -0,27755 | 0,656823 |
| ZNF155 | Primary | CRISPRa | CD8 | IFNG | -0,27266 | 0,656823 |
| ZFR2 | Primary | CRISPRa | CD8 | IFNG | -0,26885 | 0,656823 |
| MED11 | Primary | CRISPRa | CD8 | IFNG | -0,25959 | 0,656823 |
| ZNF507 | Primary | CRISPRa | CD8 | IFNG | -0,22118 | 0,656823 |
| CREG1 | Primary | CRISPRa | CD8 | IFNG | -0,22089 | 0,656823 |
| NR1I2 | Primary | CRISPRa | CD8 | IFNG | -0,19117 | 0,656823 |
| RFXAP | Primary | CRISPRa | CD8 | IFNG | -0,18831 | 0,656823 |
| BBX | Primary | CRISPRa | CD8 | IFNG | -0,1861 | 0,656823 |
| ZNF766 | Primary | CRISPRa | CD8 | IFNG | -0,17108 | 0,656823 |
| ACTR5 | Primary | CRISPRa | CD8 | IFNG | -0,15658 | 0,656823 |
| BRMS1 | Primary | CRISPRa | CD8 | IFNG | -0,14853 | 0,656823 |
| NACA | Primary | CRISPRa | CD8 | IFNG | -0,14006 | 0,656823 |
| INO80B | Primary | CRISPRa | CD8 | IFNG | -0,13751 | 0,656823 |
| BNC1 | Primary | CRISPRa | CD8 | IFNG | -0,12075 | 0,656823 |
| AGO2 | Primary | CRISPRa | CD8 | IFNG | -0,096917 | 0,656823 |
| DHFR | Primary | CRISPRa | CD8 | IFNG | -0,090263 | 0,656823 |
| NRG1 | Primary | CRISPRa | CD8 | IFNG | -0,084847 | 0,656823 |
| AKAP8L | Primary | CRISPRa | CD8 | IFNG | -0,082813 | 0,656823 |
| PARP9 | Primary | CRISPRa | CD8 | IFNG | -0,075152 | 0,656823 |
| TBX20 | Primary | CRISPRa | CD8 | IFNG | -0,0023193 | 0,656823 |
| RB1 | Primary | CRISPRa | CD8 | IFNG | 0,011985 | 0,656823 |
| ZNF768 | Primary | CRISPRa | CD8 | IFNG | 0,049039 | 0,656823 |
| ZNF594 | Primary | CRISPRa | CD8 | IFNG | 0,055467 | 0,656823 |
| RUVBL2 | Primary | CRISPRa | CD8 | IFNG | 0,20048 | 0,656823 |
| CDK12 | Primary | CRISPRa | CD8 | IFNG | -0,12638 | 0,65794 |
| ZNF573 | Primary | CRISPRa | CD8 | IFNG | -0,32646 | 0,661305 |
| TOPORS | Primary | CRISPRa | CD8 | IFNG | -0,24568 | 0,661305 |
| BPNT1 | Primary | CRISPRa | CD8 | IFNG | -0,093769 | 0,661305 |
| PER3 | Primary | CRISPRa | CD8 | IFNG | 0,11419 | 0,661305 |
| BRPF1 | Primary | CRISPRa | CD8 | IFNG | 0,18359 | 0,661305 |
| SERTAD1 | Primary | CRISPRa | CD8 | IFNG | 0,2564 | 0,661305 |
| TET2 | Primary | CRISPRa | CD8 | IFNG | 0,26161 | 0,661305 |
| CDK11A | Primary | CRISPRa | CD8 | IFNG | 0,29703 | 0,661305 |
| PSMA6 | Primary | CRISPRa | CD8 | IFNG | 0,31984 | 0,661305 |
| BUD31 | Primary | CRISPRa | CD8 | IFNG | -0,31757 | 0,661648 |
| IRF2BP1 | Primary | CRISPRa | CD8 | IFNG | -0,21218 | 0,661648 |
| NFATC3 | Primary | CRISPRa | CD8 | IFNG | 0,25311 | 0,661819 |
| ZGPAT | Primary | CRISPRa | CD8 | IFNG | 0,23309 | 0,662483 |
| POU6F2 | Primary | CRISPRa | CD8 | IFNG | 0,18688 | 0,663054 |
| CUL5 | Primary | CRISPRa | CD8 | IFNG | 0,21253 | 0,666228 |
| STAG1 | Primary | CRISPRa | CD8 | IFNG | -0,16654 | 0,666585 |
| VSX2 | Primary | CRISPRa | CD8 | IFNG | -0,36551 | 0,668449 |
| PRMT8 | Primary | CRISPRa | CD8 | IFNG | -0,30014 | 0,668449 |
| KDM4B | Primary | CRISPRa | CD8 | IFNG | -0,22933 | 0,668449 |
| FHL1 | Primary | CRISPRa | CD8 | IFNG | -0,22462 | 0,668449 |
| ATF7IP | Primary | CRISPRa | CD8 | IFNG | -0,21765 | 0,668449 |
| PELP1 | Primary | CRISPRa | CD8 | IFNG | -0,18219 | 0,668449 |
| ZNF283 | Primary | CRISPRa | CD8 | IFNG | -0,14914 | 0,668449 |
| CTCFL | Primary | CRISPRa | CD8 | IFNG | -0,096484 | 0,668449 |
| CCR7 | Primary | CRISPRa | CD8 | IFNG | -0,024357 | 0,668449 |
| TRIM13 | Primary | CRISPRa | CD8 | IFNG | 0,24096 | 0,670088 |
| SLC4A10 | Primary | CRISPRa | CD8 | IFNG | 0,2666 | 0,670088 |
| U2AF1L4 | Primary | CRISPRa | CD8 | IFNG | 0,32015 | 0,670088 |
| PURB | Primary | CRISPRa | CD8 | IFNG | -0,30497 | 0,670425 |
| MSANTD4 | Primary | CRISPRa | CD8 | IFNG | -0,2317 | 0,670425 |
| HINT1 | Primary | CRISPRa | CD8 | IFNG | 0,023036 | 0,67106 |
| ZNF597 | Primary | CRISPRa | CD8 | IFNG | -0,16592 | 0,671335 |
| ARID2 | Primary | CRISPRa | CD8 | IFNG | 0,038149 | 0,671335 |
| JMJD6 | Primary | CRISPRa | CD8 | IFNG | -0,20042 | 0,672756 |
| ZNF460 | Primary | CRISPRa | CD8 | IFNG | 0,10897 | 0,672756 |
| ZNF554 | Primary | CRISPRa | CD8 | IFNG | 0,11036 | 0,672756 |
| CDX1 | Primary | CRISPRa | CD8 | IFNG | 0,13603 | 0,672756 |
| DDX1 | Primary | CRISPRa | CD8 | IFNG | -0,28036 | 0,675016 |
| ZNF621 | Primary | CRISPRa | CD8 | IFNG | -0,037077 | 0,675016 |
| PCGF3 | Primary | CRISPRa | CD8 | IFNG | -0,0022496 | 0,675016 |
| DBX1 | Primary | CRISPRa | CD8 | IFNG | 0,22815 | 0,676072 |
| XRN2 | Primary | CRISPRa | CD8 | IFNG | 0,23601 | 0,676072 |
| EID2 | Primary | CRISPRa | CD8 | IFNG | 0,072783 | 0,67705 |
| MRRF | Primary | CRISPRa | CD8 | IFNG | -0,24167 | 0,677144 |
| RPA2 | Primary | CRISPRa | CD8 | IFNG | -0,33588 | 0,678089 |
| ZBTB17 | Primary | CRISPRa | CD8 | IFNG | 0,29647 | 0,678089 |
| SET | Primary | CRISPRa | CD8 | IFNG | -0,38266 | 0,678192 |
| TBX1 | Primary | CRISPRa | CD8 | IFNG | -0,29457 | 0,678192 |
| UBE2L3 | Primary | CRISPRa | CD8 | IFNG | -0,29143 | 0,678192 |
| KLHL40 | Primary | CRISPRa | CD8 | IFNG | -0,27559 | 0,678192 |
| TAF11 | Primary | CRISPRa | CD8 | IFNG | -0,24324 | 0,678192 |
| USP22 | Primary | CRISPRa | CD8 | IFNG | -0,22086 | 0,678192 |
| GTF3C5 | Primary | CRISPRa | CD8 | IFNG | -0,20108 | 0,678192 |
| REXO4 | Primary | CRISPRa | CD8 | IFNG | -0,17523 | 0,678192 |
| PIKFYVE | Primary | CRISPRa | CD8 | IFNG | -0,16781 | 0,678192 |
| LHX2 | Primary | CRISPRa | CD8 | IFNG | -0,15002 | 0,678192 |
| HOXA1 | Primary | CRISPRa | CD8 | IFNG | -0,13716 | 0,678192 |
| RNF10 | Primary | CRISPRa | CD8 | IFNG | -0,12239 | 0,678192 |
| FEM1A | Primary | CRISPRa | CD8 | IFNG | -0,039579 | 0,678192 |
| UHRF1 | Primary | CRISPRa | CD8 | IFNG | -0,031066 | 0,678192 |
| RNF115 | Primary | CRISPRa | CD8 | IFNG | -0,0049421 | 0,678192 |
| TADA3 | Primary | CRISPRa | CD8 | IFNG | 0,1131 | 0,678192 |
| SPIB | Primary | CRISPRa | CD8 | IFNG | 0,12291 | 0,678192 |
| SAP30BP | Primary | CRISPRa | CD8 | IFNG | 0,16082 | 0,678192 |
| DEPTOR | Primary | CRISPRa | CD8 | IFNG | 0,25088 | 0,6856 |
| CBY1 | Primary | CRISPRa | CD8 | IFNG | -0,29822 | 0,686779 |
| CHAMP1 | Primary | CRISPRa | CD8 | IFNG | -0,16972 | 0,686779 |
| NFKBIL1 | Primary | CRISPRa | CD8 | IFNG | -0,15099 | 0,686779 |
| ZFAND3 | Primary | CRISPRa | CD8 | IFNG | -0,074978 | 0,686779 |
| PIH1D1 | Primary | CRISPRa | CD8 | IFNG | -0,045214 | 0,686779 |
| KMT2B | Primary | CRISPRa | CD8 | IFNG | -0,0021829 | 0,686779 |
| CHTOP | Primary | CRISPRa | CD8 | IFNG | 0,06701 | 0,686779 |
| E2F6 | Primary | CRISPRa | CD8 | IFNG | 0,088085 | 0,686779 |
| CORO1A | Primary | CRISPRa | CD8 | IFNG | -0,18078 | 0,686926 |
| HIVEP1 | Primary | CRISPRa | CD8 | IFNG | -0,13972 | 0,686926 |
| ZNF572 | Primary | CRISPRa | CD8 | IFNG | 0,21726 | 0,686926 |
| ZNF665 | Primary | CRISPRa | CD8 | IFNG | -0,12024 | 0,68696 |
| PTOV1 | Primary | CRISPRa | CD8 | IFNG | -0,08737 | 0,68696 |
| ZNF534 | Primary | CRISPRa | CD8 | IFNG | -0,026616 | 0,687098 |
| ZNF69 | Primary | CRISPRa | CD8 | IFNG | 0,06805 | 0,687098 |
| NEUROD6 | Primary | CRISPRa | CD8 | IFNG | 0,1456 | 0,687098 |
| ZNF483 | Primary | CRISPRa | CD8 | IFNG | -0,031609 | 0,687305 |
| RNF125 | Primary | CRISPRa | CD8 | IFNG | 0,14333 | 0,687305 |
| ZNF480 | Primary | CRISPRa | CD8 | IFNG | 0,21383 | 0,687305 |
| EID2B | Primary | CRISPRa | CD8 | IFNG | 0,096334 | 0,687525 |
| GCM1 | Primary | CRISPRa | CD8 | IFNG | 0,13672 | 0,687525 |
| CDK1 | Primary | CRISPRa | CD8 | IFNG | 0,25101 | 0,687525 |
| ZNF217 | Primary | CRISPRa | CD8 | IFNG | -0,14474 | 0,688357 |
| DUSP26 | Primary | CRISPRa | CD8 | IFNG | 0,13763 | 0,688357 |
| ACTR8 | Primary | CRISPRa | CD8 | IFNG | 0,015834 | 0,688683 |
| ZNF691 | Primary | CRISPRa | CD8 | IFNG | 0,20648 | 0,688683 |
| ARID5A | Primary | CRISPRa | CD8 | IFNG | 0,054227 | 0,689409 |
| TRIP13 | Primary | CRISPRa | CD8 | IFNG | -0,11814 | 0,691246 |
| ATXN7 | Primary | CRISPRa | CD8 | IFNG | -0,37731 | 0,691952 |
| ZIC5 | Primary | CRISPRa | CD8 | IFNG | -0,3477 | 0,691952 |
| FGFR1 | Primary | CRISPRa | CD8 | IFNG | -0,31097 | 0,691952 |
| ARC | Primary | CRISPRa | CD8 | IFNG | -0,2782 | 0,691952 |
| ZNF404 | Primary | CRISPRa | CD8 | IFNG | -0,25957 | 0,691952 |
| SAV1 | Primary | CRISPRa | CD8 | IFNG | -0,25238 | 0,691952 |
| S100A1 | Primary | CRISPRa | CD8 | IFNG | -0,2279 | 0,691952 |
| ZMYM3 | Primary | CRISPRa | CD8 | IFNG | -0,22521 | 0,691952 |
| PSMC1 | Primary | CRISPRa | CD8 | IFNG | -0,22194 | 0,691952 |
| CNOT7 | Primary | CRISPRa | CD8 | IFNG | -0,16322 | 0,691952 |
| PICALM | Primary | CRISPRa | CD8 | IFNG | -0,15586 | 0,691952 |
| NSD3 | Primary | CRISPRa | CD8 | IFNG | -0,12664 | 0,691952 |
| ZNF620 | Primary | CRISPRa | CD8 | IFNG | -0,12005 | 0,691952 |
| UCHL5 | Primary | CRISPRa | CD8 | IFNG | -0,10558 | 0,691952 |
| ZNF662 | Primary | CRISPRa | CD8 | IFNG | -0,10145 | 0,691952 |
| EHMT1 | Primary | CRISPRa | CD8 | IFNG | -0,097845 | 0,691952 |
| CTBP1 | Primary | CRISPRa | CD8 | IFNG | -0,041874 | 0,691952 |
| KLHL6 | Primary | CRISPRa | CD8 | IFNG | 0,080646 | 0,691952 |
| ZNF669 | Primary | CRISPRa | CD8 | IFNG | 0,30329 | 0,692082 |
| ZNF485 | Primary | CRISPRa | CD8 | IFNG | 0,16381 | 0,692546 |
| ZNF639 | Primary | CRISPRa | CD8 | IFNG | 0,17987 | 0,692546 |
| ZXDB | Primary | CRISPRa | CD8 | IFNG | -0,045196 | 0,694769 |
| GRHL1 | Primary | CRISPRa | CD8 | IFNG | 0,029182 | 0,694769 |
| ZNF257 | Primary | CRISPRa | CD8 | IFNG | 0,035984 | 0,694769 |
| CELF3 | Primary | CRISPRa | CD8 | IFNG | 0,050167 | 0,694769 |
| ZNF543 | Primary | CRISPRa | CD8 | IFNG | 0,13105 | 0,694769 |
| ANKRD30A | Primary | CRISPRa | CD8 | IFNG | 0,13631 | 0,694769 |
| ZBED9 | Primary | CRISPRa | CD8 | IFNG | 0,19726 | 0,694769 |
| MIER2 | Primary | CRISPRa | CD8 | IFNG | 0,27213 | 0,695447 |
| SEMA4A | Primary | CRISPRa | CD8 | IFNG | -0,23709 | 0,695543 |
| ZNF680 | Primary | CRISPRa | CD8 | IFNG | -0,087636 | 0,695543 |
| MICALL1 | Primary | CRISPRa | CD8 | IFNG | 0,074035 | 0,695543 |
| RBM15 | Primary | CRISPRa | CD8 | IFNG | -0,19144 | 0,695563 |
| CERS4 | Primary | CRISPRa | CD8 | IFNG | -0,081207 | 0,695563 |
| HTATSF1 | Primary | CRISPRa | CD8 | IFNG | 0,11379 | 0,695563 |
| LMX1B | Primary | CRISPRa | CD8 | IFNG | -0,27167 | 0,695867 |
| BCL6 | Primary | CRISPRa | CD8 | IFNG | -0,019221 | 0,695867 |
| MXD1 | Primary | CRISPRa | CD8 | IFNG | -0,0016661 | 0,697326 |
| RIOX1 | Primary | CRISPRa | CD8 | IFNG | -0,12128 | 0,697711 |
| TRIM56 | Primary | CRISPRa | CD8 | IFNG | -0,18216 | 0,701085 |
| NR0B2 | Primary | CRISPRa | CD8 | IFNG | -0,10056 | 0,701085 |
| ZNF419 | Primary | CRISPRa | CD8 | IFNG | -0,053273 | 0,701085 |
| GRHL2 | Primary | CRISPRa | CD8 | IFNG | -0,049843 | 0,701085 |
| KLHL21 | Primary | CRISPRa | CD8 | IFNG | -0,03512 | 0,701085 |
| MRPL28 | Primary | CRISPRa | CD8 | IFNG | 0,06554 | 0,701085 |
| TFAM | Primary | CRISPRa | CD8 | IFNG | 0,1144 | 0,701085 |
| ZNF234 | Primary | CRISPRa | CD8 | IFNG | 0,13699 | 0,701085 |
| PRPF6 | Primary | CRISPRa | CD8 | IFNG | 0,1725 | 0,701085 |
| PER1 | Primary | CRISPRa | CD8 | IFNG | 0,28319 | 0,701085 |
| ZNF227 | Primary | CRISPRa | CD8 | IFNG | 0,28952 | 0,701085 |
| PRDM10 | Primary | CRISPRa | CD8 | IFNG | 0,32393 | 0,701085 |
| TFCP2 | Primary | CRISPRa | CD8 | IFNG | -0,18512 | 0,701714 |
| PRAP1 | Primary | CRISPRa | CD8 | IFNG | -0,062339 | 0,701714 |
| ZNF806 | Primary | CRISPRa | CD8 | IFNG | -0,024876 | 0,701714 |
| DAB2 | Primary | CRISPRa | CD8 | IFNG | 0,056536 | 0,701714 |
| S100A8 | Primary | CRISPRa | CD8 | IFNG | 0,058886 | 0,701714 |
| ELOF1 | Primary | CRISPRa | CD8 | IFNG | 0,09002 | 0,701714 |
| ASCL3 | Primary | CRISPRa | CD8 | IFNG | 0,11568 | 0,701714 |
| UXT | Primary | CRISPRa | CD8 | IFNG | 0,11587 | 0,701714 |
| SLC26A3 | Primary | CRISPRa | CD8 | IFNG | 0,17783 | 0,701714 |
| ZNF394 | Primary | CRISPRa | CD8 | IFNG | -0,11127 | 0,703795 |
| FLNA | Primary | CRISPRa | CD8 | IFNG | -0,09487 | 0,703795 |
| TRIOBP | Primary | CRISPRa | CD8 | IFNG | 0,0075714 | 0,703795 |
| SIN3A | Primary | CRISPRa | CD8 | IFNG | -0,0033969 | 0,704256 |
| MYPOP | Primary | CRISPRa | CD8 | IFNG | 0,21034 | 0,704256 |
| PPP1R13L | Primary | CRISPRa | CD8 | IFNG | 0,25866 | 0,704256 |
| SLC25A2 | Primary | CRISPRa | CD8 | IFNG | -0,014826 | 0,704468 |
| MYCN | Primary | CRISPRa | CD8 | IFNG | 0,053471 | 0,704719 |
| FRK | Primary | CRISPRa | CD8 | IFNG | 0,067403 | 0,704719 |
| IRX6 | Primary | CRISPRa | CD8 | IFNG | 0,15191 | 0,705227 |
| TAF7 | Primary | CRISPRa | CD8 | IFNG | -0,048992 | 0,706351 |
| IRAK3 | Primary | CRISPRa | CD8 | IFNG | -0,071953 | 0,707061 |
| RBBP8 | Primary | CRISPRa | CD8 | IFNG | 0,044172 | 0,707061 |
| CAMK2A | Primary | CRISPRa | CD8 | IFNG | 0,12086 | 0,707061 |
| TFAP4 | Primary | CRISPRa | CD8 | IFNG | 0,17619 | 0,707061 |
| EAPP | Primary | CRISPRa | CD8 | IFNG | -0,23787 | 0,707364 |
| SIX6 | Primary | CRISPRa | CD8 | IFNG | 0,16715 | 0,707364 |
| TGIF2LY | Primary | CRISPRa | CD8 | IFNG | 0,17288 | 0,707364 |
| BOD1L1 | Primary | CRISPRa | CD8 | IFNG | 0,2229 | 0,708242 |
| TMEM260 | Primary | CRISPRa | CD8 | IFNG | -0,28189 | 0,709095 |
| TAF6L | Primary | CRISPRa | CD8 | IFNG | -0,2422 | 0,709095 |
| PIK3R1 | Primary | CRISPRa | CD8 | IFNG | -0,2227 | 0,709095 |
| HR | Primary | CRISPRa | CD8 | IFNG | -0,18821 | 0,709095 |
| HIPK1 | Primary | CRISPRa | CD8 | IFNG | -0,18548 | 0,709095 |
| RNF103 | Primary | CRISPRa | CD8 | IFNG | -0,14787 | 0,709095 |
| CCNA2 | Primary | CRISPRa | CD8 | IFNG | -0,12158 | 0,709095 |
| PRAMEF25 | Primary | CRISPRa | CD8 | IFNG | -0,10531 | 0,709095 |
| ZNF628 | Primary | CRISPRa | CD8 | IFNG | -0,10325 | 0,709095 |
| RBX1 | Primary | CRISPRa | CD8 | IFNG | -0,093021 | 0,709095 |
| HOXD8 | Primary | CRISPRa | CD8 | IFNG | -0,059724 | 0,709095 |
| ZBTB7B | Primary | CRISPRa | CD8 | IFNG | 0,089748 | 0,709095 |
| KAT8 | Primary | CRISPRa | CD8 | IFNG | -0,31579 | 0,709781 |
| OSR2 | Primary | CRISPRa | CD8 | IFNG | -0,10118 | 0,710034 |
| RNF141 | Primary | CRISPRa | CD8 | IFNG | -0,062975 | 0,710034 |
| CAPN15 | Primary | CRISPRa | CD8 | IFNG | -0,047471 | 0,710034 |
| HLA-DQB1 | Primary | CRISPRa | CD8 | IFNG | -0,043707 | 0,710034 |
| ZNF730 | Primary | CRISPRa | CD8 | IFNG | 0,041577 | 0,710034 |
| USP16 | Primary | CRISPRa | CD8 | IFNG | 0,061527 | 0,710034 |
| NFAT5 | Primary | CRISPRa | CD8 | IFNG | 0,29026 | 0,710034 |
| MTF2 | Primary | CRISPRa | CD8 | IFNG | 0,25711 | 0,710489 |
| ZNF653 | Primary | CRISPRa | CD8 | IFNG | 0,034878 | 0,710773 |
| ZRSR2 | Primary | CRISPRa | CD8 | IFNG | 0,23301 | 0,710773 |
| ANP32E | Primary | CRISPRa | CD8 | IFNG | -0,35909 | 0,711668 |
| PAX1 | Primary | CRISPRa | CD8 | IFNG | -0,29093 | 0,711668 |
| ZNF43 | Primary | CRISPRa | CD8 | IFNG | -0,25089 | 0,711668 |
| FOXN1 | Primary | CRISPRa | CD8 | IFNG | -0,22713 | 0,711668 |
| TCEAL3 | Primary | CRISPRa | CD8 | IFNG | -0,19346 | 0,711668 |
| SPDEF | Primary | CRISPRa | CD8 | IFNG | -0,16271 | 0,711668 |
| ZNF629 | Primary | CRISPRa | CD8 | IFNG | -0,1334 | 0,711668 |
| ZNF287 | Primary | CRISPRa | CD8 | IFNG | -0,1081 | 0,711668 |
| FOXG1 | Primary | CRISPRa | CD8 | IFNG | -0,012093 | 0,711668 |
| TCEA2 | Primary | CRISPRa | CD8 | IFNG | 0,051534 | 0,711668 |
| PRAMEF6 | Primary | CRISPRa | CD8 | IFNG | 0,13201 | 0,711668 |
| ZBTB40 | Primary | CRISPRa | CD8 | IFNG | 0,13765 | 0,711668 |
| AURKB | Primary | CRISPRa | CD8 | IFNG | 0,23687 | 0,711668 |
| SCX | Primary | CRISPRa | CD8 | IFNG | 0,24009 | 0,711668 |
| NR4A2 | Primary | CRISPRa | CD8 | IFNG | -0,22214 | 0,71289 |
| SP7 | Primary | CRISPRa | CD8 | IFNG | -0,34041 | 0,717018 |
| LBH | Primary | CRISPRa | CD8 | IFNG | -0,30644 | 0,717018 |
| DDX5 | Primary | CRISPRa | CD8 | IFNG | -0,29845 | 0,717018 |
| PRICKLE3 | Primary | CRISPRa | CD8 | IFNG | -0,28153 | 0,717018 |
| MXD4 | Primary | CRISPRa | CD8 | IFNG | -0,28152 | 0,717018 |
| MBD6 | Primary | CRISPRa | CD8 | IFNG | -0,25965 | 0,717018 |
| MED21 | Primary | CRISPRa | CD8 | IFNG | -0,24758 | 0,717018 |
| ZNF789 | Primary | CRISPRa | CD8 | IFNG | -0,22355 | 0,717018 |
| TWIST1 | Primary | CRISPRa | CD8 | IFNG | -0,20477 | 0,717018 |
| NKX3-1 | Primary | CRISPRa | CD8 | IFNG | -0,20248 | 0,717018 |
| CSRP2 | Primary | CRISPRa | CD8 | IFNG | -0,20123 | 0,717018 |
| FOXD4L3 | Primary | CRISPRa | CD8 | IFNG | -0,19192 | 0,717018 |
| ZNF516 | Primary | CRISPRa | CD8 | IFNG | -0,18479 | 0,717018 |
| CNOT4 | Primary | CRISPRa | CD8 | IFNG | -0,18258 | 0,717018 |
| ANKRD2 | Primary | CRISPRa | CD8 | IFNG | -0,18033 | 0,717018 |
| LOXL2 | Primary | CRISPRa | CD8 | IFNG | -0,17927 | 0,717018 |
| OLIG2 | Primary | CRISPRa | CD8 | IFNG | -0,16949 | 0,717018 |
| ARID3C | Primary | CRISPRa | CD8 | IFNG | -0,16016 | 0,717018 |
| CLOCK | Primary | CRISPRa | CD8 | IFNG | -0,13822 | 0,717018 |
| PSMD9 | Primary | CRISPRa | CD8 | IFNG | -0,13769 | 0,717018 |
| ZNF85 | Primary | CRISPRa | CD8 | IFNG | -0,13731 | 0,717018 |
| RNF113A | Primary | CRISPRa | CD8 | IFNG | -0,1281 | 0,717018 |
| LARP1 | Primary | CRISPRa | CD8 | IFNG | -0,09718 | 0,717018 |
| ZFP3 | Primary | CRISPRa | CD8 | IFNG | -0,094478 | 0,717018 |
| PCBD1 | Primary | CRISPRa | CD8 | IFNG | -0,078074 | 0,717018 |
| LPXN | Primary | CRISPRa | CD8 | IFNG | -0,076265 | 0,717018 |
| USP47 | Primary | CRISPRa | CD8 | IFNG | -0,071023 | 0,717018 |
| HNRNPK | Primary | CRISPRa | CD8 | IFNG | -0,063144 | 0,717018 |
| PCSK6 | Primary | CRISPRa | CD8 | IFNG | -0,047234 | 0,717018 |
| HIF1AN | Primary | CRISPRa | CD8 | IFNG | -0,041702 | 0,717018 |
| TRIM27 | Primary | CRISPRa | CD8 | IFNG | -0,038766 | 0,717018 |
| MEAF6 | Primary | CRISPRa | CD8 | IFNG | -0,037733 | 0,717018 |
| KCMF1 | Primary | CRISPRa | CD8 | IFNG | -0,022671 | 0,717018 |
| GTF3C4 | Primary | CRISPRa | CD8 | IFNG | -0,015054 | 0,717018 |
| RNF20 | Primary | CRISPRa | CD8 | IFNG | -0,013392 | 0,717018 |
| COPS5 | Primary | CRISPRa | CD8 | IFNG | -0,009618 | 0,717018 |
| ZNF496 | Primary | CRISPRa | CD8 | IFNG | 0,0064973 | 0,717018 |
| VEGFA | Primary | CRISPRa | CD8 | IFNG | 0,052744 | 0,717018 |
| OLIG1 | Primary | CRISPRa | CD8 | IFNG | 0,062828 | 0,717018 |
| HSFX1 | Primary | CRISPRa | CD8 | IFNG | 0,063056 | 0,717018 |
| NELFA | Primary | CRISPRa | CD8 | IFNG | 0,067817 | 0,717018 |
| BCL6B | Primary | CRISPRa | CD8 | IFNG | 0,068602 | 0,717018 |
| CITED4 | Primary | CRISPRa | CD8 | IFNG | 0,070741 | 0,717018 |
| ATF1 | Primary | CRISPRa | CD8 | IFNG | 0,14993 | 0,717018 |
| CSRP3 | Primary | CRISPRa | CD8 | IFNG | 0,15647 | 0,717018 |
| AFDN | Primary | CRISPRa | CD8 | IFNG | 0,18492 | 0,717808 |
| CCND1 | Primary | CRISPRa | CD8 | IFNG | -0,096614 | 0,71812 |
| NPAS1 | Primary | CRISPRa | CD8 | IFNG | -0,023408 | 0,71812 |
| TIGD7 | Primary | CRISPRa | CD8 | IFNG | -0,021958 | 0,718778 |
| MBD4 | Primary | CRISPRa | CD8 | IFNG | 0,04398 | 0,718778 |
| PHTF2 | Primary | CRISPRa | CD8 | IFNG | 0,072294 | 0,718778 |
| ZNF844 | Primary | CRISPRa | CD8 | IFNG | 0,094351 | 0,718778 |
| TAF1L | Primary | CRISPRa | CD8 | IFNG | 0,0986 | 0,718778 |
| SKAP1 | Primary | CRISPRa | CD8 | IFNG | -0,011 | 0,719872 |
| AUTS2 | Primary | CRISPRa | CD8 | IFNG | 0,039779 | 0,719872 |
| ZNF148 | Primary | CRISPRa | CD8 | IFNG | -0,15444 | 0,72003 |
| LMX1A | Primary | CRISPRa | CD8 | IFNG | -0,079496 | 0,72003 |
| STAT5A | Primary | CRISPRa | CD8 | IFNG | -0,085313 | 0,721111 |
| NKX6-3 | Primary | CRISPRa | CD8 | IFNG | -0,020922 | 0,721111 |
| ZNF587B | Primary | CRISPRa | CD8 | IFNG | 0,0090987 | 0,721111 |
| ZNF396 | Primary | CRISPRa | CD8 | IFNG | 0,059787 | 0,721111 |
| TRAF6 | Primary | CRISPRa | CD8 | IFNG | 0,070029 | 0,721111 |
| KLF9 | Primary | CRISPRa | CD8 | IFNG | 0,078777 | 0,721111 |
| PHF12 | Primary | CRISPRa | CD8 | IFNG | 0,10894 | 0,721111 |
| BCLAF1 | Primary | CRISPRa | CD8 | IFNG | 0,12587 | 0,721111 |
| RNF144A | Primary | CRISPRa | CD8 | IFNG | 0,22587 | 0,721111 |
| ELK4 | Primary | CRISPRa | CD8 | IFNG | 0,11588 | 0,722809 |
| KAT7 | Primary | CRISPRa | CD8 | IFNG | 0,093346 | 0,723131 |
| DMRTA1 | Primary | CRISPRa | CD8 | IFNG | -0,27009 | 0,724369 |
| HOMEZ | Primary | CRISPRa | CD8 | IFNG | -0,16805 | 0,724369 |
| AIM2 | Primary | CRISPRa | CD8 | IFNG | -0,067026 | 0,724369 |
| NR0B1 | Primary | CRISPRa | CD8 | IFNG | -0,062276 | 0,724369 |
| SSH2 | Primary | CRISPRa | CD8 | IFNG | -0,056927 | 0,724369 |
| VHL | Primary | CRISPRa | CD8 | IFNG | 0,10603 | 0,724369 |
| ENO1 | Primary | CRISPRa | CD8 | IFNG | -0,25485 | 0,725063 |
| GON4L | Primary | CRISPRa | CD8 | IFNG | -0,031201 | 0,725063 |
| HNF1A | Primary | CRISPRa | CD8 | IFNG | -0,020012 | 0,725063 |
| NONO | Primary | CRISPRa | CD8 | IFNG | 0,01575 | 0,726331 |
| ZNF26 | Primary | CRISPRa | CD8 | IFNG | -0,1606 | 0,72722 |
| ETV7 | Primary | CRISPRa | CD8 | IFNG | -0,14967 | 0,72722 |
| HRAS | Primary | CRISPRa | CD8 | IFNG | -0,09646 | 0,72722 |
| FHL5 | Primary | CRISPRa | CD8 | IFNG | -0,085552 | 0,72722 |
| PIAS2 | Primary | CRISPRa | CD8 | IFNG | 0,061341 | 0,72722 |
| SPAG8 | Primary | CRISPRa | CD8 | IFNG | 0,069855 | 0,72722 |
| TAF13 | Primary | CRISPRa | CD8 | IFNG | 0,095861 | 0,72722 |
| SMARCC2 | Primary | CRISPRa | CD8 | IFNG | 0,18732 | 0,72722 |
| SIX1 | Primary | CRISPRa | CD8 | IFNG | 0,33282 | 0,72722 |
| MBD2 | Primary | CRISPRa | CD8 | IFNG | -0,18075 | 0,72802 |
| VGLL3 | Primary | CRISPRa | CD8 | IFNG | -0,030319 | 0,72802 |
| ZNF843 | Primary | CRISPRa | CD8 | IFNG | -0,17869 | 0,729042 |
| ARX | Primary | CRISPRa | CD8 | IFNG | -0,11266 | 0,729042 |
| ZNF605 | Primary | CRISPRa | CD8 | IFNG | -0,069723 | 0,729042 |
| ZNF30 | Primary | CRISPRa | CD8 | IFNG | -0,035261 | 0,729042 |
| ENG | Primary | CRISPRa | CD8 | IFNG | -0,023832 | 0,729042 |
| MID2 | Primary | CRISPRa | CD8 | IFNG | 0,014768 | 0,729042 |
| STON1 | Primary | CRISPRa | CD8 | IFNG | 0,040745 | 0,729042 |
| NR6A1 | Primary | CRISPRa | CD8 | IFNG | 0,051872 | 0,729042 |
| PITX2 | Primary | CRISPRa | CD8 | IFNG | 0,12298 | 0,729042 |
| PRMT7 | Primary | CRISPRa | CD8 | IFNG | 0,13855 | 0,729042 |
| ZNF835 | Primary | CRISPRa | CD8 | IFNG | 0,14988 | 0,729042 |
| ZNF33A | Primary | CRISPRa | CD8 | IFNG | 0,15481 | 0,729042 |
| XPC | Primary | CRISPRa | CD8 | IFNG | 0,23343 | 0,729042 |
| ZNF705G | Primary | CRISPRa | CD8 | IFNG | 0,24074 | 0,729042 |
| SAFB | Primary | CRISPRa | CD8 | IFNG | 0,28002 | 0,729042 |
| BARX2 | Primary | CRISPRa | CD8 | IFNG | -0,037667 | 0,731172 |
| ZNF322 | Primary | CRISPRa | CD8 | IFNG | -0,11934 | 0,731357 |
| SNAPC5 | Primary | CRISPRa | CD8 | IFNG | -0,019544 | 0,731357 |
| HLA-DRB3 | Primary | CRISPRa | CD8 | IFNG | 0,17607 | 0,731357 |
| HMG20A | Primary | CRISPRa | CD8 | IFNG | 0,19198 | 0,731357 |
| ASXL1 | Primary | CRISPRa | CD8 | IFNG | 0,2225 | 0,731357 |
| PRDM2 | Primary | CRISPRa | CD8 | IFNG | -0,28894 | 0,732729 |
| MAZ | Primary | CRISPRa | CD8 | IFNG | -0,25077 | 0,732729 |
| ZNF385A | Primary | CRISPRa | CD8 | IFNG | -0,21289 | 0,732729 |
| NOC3L | Primary | CRISPRa | CD8 | IFNG | -0,12441 | 0,732729 |
| POLR2L | Primary | CRISPRa | CD8 | IFNG | 0,054346 | 0,732729 |
| PRAMEF11 | Primary | CRISPRa | CD8 | IFNG | 0,055212 | 0,737004 |
| REPIN1 | Primary | CRISPRa | CD8 | IFNG | 0,045468 | 0,737734 |
| NDN | Primary | CRISPRa | CD8 | IFNG | -0,29655 | 0,739448 |
| PTTG1 | Primary | CRISPRa | CD8 | IFNG | -0,17154 | 0,739448 |
| SHOX | Primary | CRISPRa | CD8 | IFNG | -0,11276 | 0,739448 |
| RPRD1A | Primary | CRISPRa | CD8 | IFNG | 0,077557 | 0,739448 |
| SOX6 | Primary | CRISPRa | CD8 | IFNG | -0,10454 | 0,740141 |
| ZNF500 | Primary | CRISPRa | CD8 | IFNG | -0,0024903 | 0,740141 |
| SSX5 | Primary | CRISPRa | CD8 | IFNG | 0,124 | 0,740141 |
| MALT1 | Primary | CRISPRa | CD8 | IFNG | 0,14837 | 0,740141 |
| NFXL1 | Primary | CRISPRa | CD8 | IFNG | -0,20861 | 0,741265 |
| HCFC2 | Primary | CRISPRa | CD8 | IFNG | -0,032426 | 0,74299 |
| NR2F2 | Primary | CRISPRa | CD8 | IFNG | -0,24098 | 0,743036 |
| ZIC4 | Primary | CRISPRa | CD8 | IFNG | -0,23618 | 0,743036 |
| MTERF2 | Primary | CRISPRa | CD8 | IFNG | -0,22781 | 0,743036 |
| DYDC1 | Primary | CRISPRa | CD8 | IFNG | -0,075034 | 0,743036 |
| SSH1 | Primary | CRISPRa | CD8 | IFNG | -0,07162 | 0,743036 |
| SSX3 | Primary | CRISPRa | CD8 | IFNG | -0,16346 | 0,744889 |
| ZRANB2 | Primary | CRISPRa | CD8 | IFNG | -0,12652 | 0,747134 |
| APTX | Primary | CRISPRa | CD8 | IFNG | -0,083079 | 0,747134 |
| SPI1 | Primary | CRISPRa | CD8 | IFNG | -0,064156 | 0,747134 |
| ACTR6 | Primary | CRISPRa | CD8 | IFNG | -0,022051 | 0,747134 |
| SORBS2 | Primary | CRISPRa | CD8 | IFNG | 0,1085 | 0,747134 |
| HMGB1 | Primary | CRISPRa | CD8 | IFNG | 0,14657 | 0,747134 |
| TEF | Primary | CRISPRa | CD8 | IFNG | 0,23516 | 0,747134 |
| MTOR | Primary | CRISPRa | CD8 | IFNG | -0,10984 | 0,748638 |
| MED4 | Primary | CRISPRa | CD8 | IFNG | -0,10605 | 0,748638 |
| SERPIND1 | Primary | CRISPRa | CD8 | IFNG | -0,060921 | 0,748638 |
| CRIP2 | Primary | CRISPRa | CD8 | IFNG | 0,020848 | 0,748638 |
| ZNF70 | Primary | CRISPRa | CD8 | IFNG | 0,21909 | 0,748638 |
| SMURF2 | Primary | CRISPRa | CD8 | IFNG | -0,3442 | 0,750736 |
| SOX11 | Primary | CRISPRa | CD8 | IFNG | -0,29921 | 0,750736 |
| ZNF512B | Primary | CRISPRa | CD8 | IFNG | -0,29891 | 0,750736 |
| THRA | Primary | CRISPRa | CD8 | IFNG | -0,29525 | 0,750736 |
| PRKDC | Primary | CRISPRa | CD8 | IFNG | -0,28213 | 0,750736 |
| PPM1A | Primary | CRISPRa | CD8 | IFNG | -0,26147 | 0,750736 |
| ZBTB48 | Primary | CRISPRa | CD8 | IFNG | -0,20717 | 0,750736 |
| PLRG1 | Primary | CRISPRa | CD8 | IFNG | -0,19958 | 0,750736 |
| PHF21B | Primary | CRISPRa | CD8 | IFNG | -0,17523 | 0,750736 |
| ZNF492 | Primary | CRISPRa | CD8 | IFNG | -0,1709 | 0,750736 |
| RLF | Primary | CRISPRa | CD8 | IFNG | -0,14476 | 0,750736 |
| PROX2 | Primary | CRISPRa | CD8 | IFNG | -0,1385 | 0,750736 |
| TLE2 | Primary | CRISPRa | CD8 | IFNG | -0,12667 | 0,750736 |
| SARNP | Primary | CRISPRa | CD8 | IFNG | -0,072072 | 0,750736 |
| ZNF256 | Primary | CRISPRa | CD8 | IFNG | -0,050039 | 0,750736 |
| LIN28A | Primary | CRISPRa | CD8 | IFNG | 0,01253 | 0,750736 |
| NMI | Primary | CRISPRa | CD8 | IFNG | 0,14566 | 0,750736 |
| SALL3 | Primary | CRISPRa | CD8 | IFNG | -0,1753 | 0,751479 |
| ZHX2 | Primary | CRISPRa | CD8 | IFNG | -0,021755 | 0,751715 |
| BCL11B | Primary | CRISPRa | CD8 | IFNG | -0,2176 | 0,755092 |
| MAPK9 | Primary | CRISPRa | CD8 | IFNG | -0,11046 | 0,755092 |
| ZNF714 | Primary | CRISPRa | CD8 | IFNG | -0,050456 | 0,755092 |
| MEIS2 | Primary | CRISPRa | CD8 | IFNG | -0,057941 | 0,755543 |
| ZNF746 | Primary | CRISPRa | CD8 | IFNG | -0,17066 | 0,756051 |
| HAND1 | Primary | CRISPRa | CD8 | IFNG | -0,11683 | 0,756051 |
| BTBD3 | Primary | CRISPRa | CD8 | IFNG | 0,016401 | 0,757639 |
| ZBTB44 | Primary | CRISPRa | CD8 | IFNG | 0,061805 | 0,757639 |
| ZBED4 | Primary | CRISPRa | CD8 | IFNG | 0,18862 | 0,757639 |
| PHB2 | Primary | CRISPRa | CD8 | IFNG | 0,23564 | 0,758199 |
| ZNF208 | Primary | CRISPRa | CD8 | IFNG | -0,15684 | 0,758204 |
| ZSCAN29 | Primary | CRISPRa | CD8 | IFNG | 0,086772 | 0,758452 |
| KDM1A | Primary | CRISPRa | CD8 | IFNG | -0,24059 | 0,758795 |
| OTOP3 | Primary | CRISPRa | CD8 | IFNG | 0,058893 | 0,7588 |
| TRMT1 | Primary | CRISPRa | CD8 | IFNG | 0,098799 | 0,758996 |
| AGO1 | Primary | CRISPRa | CD8 | IFNG | -0,27124 | 0,760174 |
| NKX6-2 | Primary | CRISPRa | CD8 | IFNG | -0,17311 | 0,76021 |
| UBB | Primary | CRISPRa | CD8 | IFNG | -0,11513 | 0,76021 |
| TPR | Primary | CRISPRa | CD8 | IFNG | 0,035625 | 0,76021 |
| PLXNA2 | Primary | CRISPRa | CD8 | IFNG | -0,39299 | 0,76134 |
| MAFG | Primary | CRISPRa | CD8 | IFNG | -0,36278 | 0,76134 |
| MYOCD | Primary | CRISPRa | CD8 | IFNG | -0,26419 | 0,76134 |
| MBD3L1 | Primary | CRISPRa | CD8 | IFNG | -0,1963 | 0,76134 |
| N4BP2L2 | Primary | CRISPRa | CD8 | IFNG | -0,15296 | 0,76134 |
| TULP4 | Primary | CRISPRa | CD8 | IFNG | -0,14154 | 0,76134 |
| ZNF135 | Primary | CRISPRa | CD8 | IFNG | -0,13999 | 0,76134 |
| ZC3H3 | Primary | CRISPRa | CD8 | IFNG | -0,13531 | 0,76134 |
| ZNF8 | Primary | CRISPRa | CD8 | IFNG | -0,10331 | 0,76134 |
| E2F4 | Primary | CRISPRa | CD8 | IFNG | -0,08194 | 0,76134 |
| NUCKS1 | Primary | CRISPRa | CD8 | IFNG | -0,029056 | 0,76134 |
| SNIP1 | Primary | CRISPRa | CD8 | IFNG | -0,014279 | 0,76134 |
| EAF1 | Primary | CRISPRa | CD8 | IFNG | -0,013779 | 0,76134 |
| RBBP5 | Primary | CRISPRa | CD8 | IFNG | 0,038769 | 0,76134 |
| DUSP22 | Primary | CRISPRa | CD8 | IFNG | 0,042957 | 0,76134 |
| IFI16 | Primary | CRISPRa | CD8 | IFNG | 0,086312 | 0,76134 |
| CNBP | Primary | CRISPRa | CD8 | IFNG | -0,27722 | 0,761653 |
| ZNF268 | Primary | CRISPRa | CD8 | IFNG | -0,26508 | 0,761653 |
| ANKRD33 | Primary | CRISPRa | CD8 | IFNG | -0,23559 | 0,761653 |
| IKZF4 | Primary | CRISPRa | CD8 | IFNG | -0,21152 | 0,761653 |
| GTF2H5 | Primary | CRISPRa | CD8 | IFNG | -0,16524 | 0,761653 |
| ZNF518A | Primary | CRISPRa | CD8 | IFNG | -0,12132 | 0,761653 |
| ZNF225 | Primary | CRISPRa | CD8 | IFNG | -0,11952 | 0,761653 |
| ZNF692 | Primary | CRISPRa | CD8 | IFNG | -0,1155 | 0,761653 |
| ARHGAP5 | Primary | CRISPRa | CD8 | IFNG | -0,11436 | 0,761653 |
| LBX1 | Primary | CRISPRa | CD8 | IFNG | -0,10534 | 0,761653 |
| NSD1 | Primary | CRISPRa | CD8 | IFNG | -0,104 | 0,761653 |
| MKRN2 | Primary | CRISPRa | CD8 | IFNG | -0,063247 | 0,761653 |
| EN1 | Primary | CRISPRa | CD8 | IFNG | -0,054732 | 0,761653 |
| TRAFD1 | Primary | CRISPRa | CD8 | IFNG | -0,032442 | 0,761653 |
| YY2 | Primary | CRISPRa | CD8 | IFNG | -0,022967 | 0,761653 |
| ZNF551 | Primary | CRISPRa | CD8 | IFNG | -0,013521 | 0,761653 |
| ZMYM5 | Primary | CRISPRa | CD8 | IFNG | 0,0048178 | 0,761653 |
| UFL1 | Primary | CRISPRa | CD8 | IFNG | 0,02117 | 0,761653 |
| NFATC2IP | Primary | CRISPRa | CD8 | IFNG | 0,023877 | 0,761653 |
| CDR2 | Primary | CRISPRa | CD8 | IFNG | 0,025123 | 0,761653 |
| CHCHD2 | Primary | CRISPRa | CD8 | IFNG | 0,069223 | 0,761653 |
| NPM2 | Primary | CRISPRa | CD8 | IFNG | 0,1197 | 0,761653 |
| ZNF114 | Primary | CRISPRa | CD8 | IFNG | -0,13762 | 0,76197 |
| RNF166 | Primary | CRISPRa | CD8 | IFNG | 0,016002 | 0,76197 |
| ZNF527 | Primary | CRISPRa | CD8 | IFNG | 0,039791 | 0,763591 |
| YWHAB | Primary | CRISPRa | CD8 | IFNG | 0,14738 | 0,763591 |
| PPARA | Primary | CRISPRa | CD8 | IFNG | -0,14218 | 0,764344 |
| ASCC1 | Primary | CRISPRa | CD8 | IFNG | 0,085806 | 0,764463 |
| ARIH2 | Primary | CRISPRa | CD8 | IFNG | -0,12076 | 0,764491 |
| TTLL5 | Primary | CRISPRa | CD8 | IFNG | -0,071567 | 0,764491 |
| PNRC1 | Primary | CRISPRa | CD8 | IFNG | -0,06569 | 0,764491 |
| CNOT8 | Primary | CRISPRa | CD8 | IFNG | 0,18104 | 0,764491 |
| INPP5K | Primary | CRISPRa | CD8 | IFNG | 0,004825 | 0,764625 |
| ZBTB24 | Primary | CRISPRa | CD8 | IFNG | 0,043028 | 0,764625 |
| TRIM5 | Primary | CRISPRa | CD8 | IFNG | 0,042133 | 0,765408 |
| KCNIP3 | Primary | CRISPRa | CD8 | IFNG | -0,2386 | 0,76561 |
| WAC | Primary | CRISPRa | CD8 | IFNG | -0,085964 | 0,76561 |
| ATMIN | Primary | CRISPRa | CD8 | IFNG | -0,048762 | 0,76561 |
| BEND6 | Primary | CRISPRa | CD8 | IFNG | 0,000069877 | 0,76561 |
| ZNF91 | Primary | CRISPRa | CD8 | IFNG | 0,0033812 | 0,76561 |
| SIRT3 | Primary | CRISPRa | CD8 | IFNG | 0,010495 | 0,76561 |
| ZNF732 | Primary | CRISPRa | CD8 | IFNG | 0,037269 | 0,76561 |
| ZMAT1 | Primary | CRISPRa | CD8 | IFNG | 0,087465 | 0,76561 |
| PBX3 | Primary | CRISPRa | CD8 | IFNG | 0,17091 | 0,76561 |
| CERS6 | Primary | CRISPRa | CD8 | IFNG | 0,28263 | 0,76561 |
| TET1 | Primary | CRISPRa | CD8 | IFNG | -0,20164 | 0,766218 |
| IRF2BP2 | Primary | CRISPRa | CD8 | IFNG | -0,19806 | 0,766218 |
| BBS7 | Primary | CRISPRa | CD8 | IFNG | -0,1477 | 0,766218 |
| CUL4A | Primary | CRISPRa | CD8 | IFNG | -0,12526 | 0,766218 |
| ZNF324B | Primary | CRISPRa | CD8 | IFNG | -0,11365 | 0,766218 |
| RBM5 | Primary | CRISPRa | CD8 | IFNG | -0,016074 | 0,766218 |
| HEXIM1 | Primary | CRISPRa | CD8 | IFNG | -0,0083988 | 0,766218 |
| ZNF521 | Primary | CRISPRa | CD8 | IFNG | 0,043647 | 0,766218 |
| RXRG | Primary | CRISPRa | CD8 | IFNG | 0,1212 | 0,766218 |
| KLF6 | Primary | CRISPRa | CD8 | IFNG | 0,12353 | 0,766218 |
| CEP290 | Primary | CRISPRa | CD8 | IFNG | 0,25785 | 0,766218 |
| ZNF585A | Primary | CRISPRa | CD8 | IFNG | 0,27205 | 0,766218 |
| TIGD2 | Primary | CRISPRa | CD8 | IFNG | -0,29584 | 0,770485 |
| ZNF440 | Primary | CRISPRa | CD8 | IFNG | -0,17268 | 0,770485 |
| ARID3A | Primary | CRISPRa | CD8 | IFNG | -0,15677 | 0,770485 |
| TAF5 | Primary | CRISPRa | CD8 | IFNG | -0,12128 | 0,770485 |
| MBD3L2 | Primary | CRISPRa | CD8 | IFNG | -0,11407 | 0,770485 |
| ZNF606 | Primary | CRISPRa | CD8 | IFNG | -0,10249 | 0,770485 |
| CCDC71 | Primary | CRISPRa | CD8 | IFNG | -0,093098 | 0,770485 |
| MTERF1 | Primary | CRISPRa | CD8 | IFNG | -0,081882 | 0,770485 |
| RNF4 | Primary | CRISPRa | CD8 | IFNG | 0,0096283 | 0,770485 |
| CDK7 | Primary | CRISPRa | CD8 | IFNG | 0,015449 | 0,770485 |
| KBTBD7 | Primary | CRISPRa | CD8 | IFNG | 0,016135 | 0,770485 |
| SND1 | Primary | CRISPRa | CD8 | IFNG | 0,023309 | 0,770485 |
| ZNF45 | Primary | CRISPRa | CD8 | IFNG | 0,046261 | 0,770485 |
| MED24 | Primary | CRISPRa | CD8 | IFNG | 0,051654 | 0,770485 |
| BTF3 | Primary | CRISPRa | CD8 | IFNG | 0,057511 | 0,770485 |
| TNNI2 | Primary | CRISPRa | CD8 | IFNG | 0,12803 | 0,770485 |
| MAF | Primary | CRISPRa | CD8 | IFNG | 0,14388 | 0,770485 |
| MED12L | Primary | CRISPRa | CD8 | IFNG | 0,039323 | 0,771495 |
| MEF2D | Primary | CRISPRa | CD8 | IFNG | 0,087285 | 0,771495 |
| HP1BP3 | Primary | CRISPRa | CD8 | IFNG | 0,21352 | 0,771495 |
| USF2 | Primary | CRISPRa | CD8 | IFNG | 0,23368 | 0,771495 |
| MZF1 | Primary | CRISPRa | CD8 | IFNG | 0,031874 | 0,771608 |
| LHX1 | Primary | CRISPRa | CD8 | IFNG | -0,17091 | 0,77198 |
| ZNF79 | Primary | CRISPRa | CD8 | IFNG | -0,14583 | 0,77198 |
| GTF2IRD1 | Primary | CRISPRa | CD8 | IFNG | 0,13761 | 0,77198 |
| WWP2 | Primary | CRISPRa | CD8 | IFNG | -0,18419 | 0,773452 |
| PPP2R3B | Primary | CRISPRa | CD8 | IFNG | 0,072322 | 0,773452 |
| PRAMEF9 | Primary | CRISPRa | CD8 | IFNG | -0,07634 | 0,77444 |
| PHIP | Primary | CRISPRa | CD8 | IFNG | -0,12617 | 0,77742 |
| TERF2 | Primary | CRISPRa | CD8 | IFNG | -0,11985 | 0,77742 |
| ZSCAN4 | Primary | CRISPRa | CD8 | IFNG | 0,14746 | 0,77742 |
| ZNF544 | Primary | CRISPRa | CD8 | IFNG | 0,090424 | 0,778029 |
| RBL1 | Primary | CRISPRa | CD8 | IFNG | -0,17298 | 0,778351 |
| NIF3L1 | Primary | CRISPRa | CD8 | IFNG | 0,07071 | 0,778351 |
| JRK | Primary | CRISPRa | CD8 | IFNG | 0,16382 | 0,778351 |
| MED10 | Primary | CRISPRa | CD8 | IFNG | 0,027988 | 0,778712 |
| ACTN2 | Primary | CRISPRa | CD8 | IFNG | 0,12539 | 0,778712 |
| CREB3L4 | Primary | CRISPRa | CD8 | IFNG | -0,25325 | 0,77929 |
| DDB2 | Primary | CRISPRa | CD8 | IFNG | -0,2125 | 0,77929 |
| ZNF667 | Primary | CRISPRa | CD8 | IFNG | -0,01174 | 0,780579 |
| TRIP4 | Primary | CRISPRa | CD8 | IFNG | 0,026898 | 0,780579 |
| SCRT1 | Primary | CRISPRa | CD8 | IFNG | -0,030075 | 0,780635 |
| RGS20 | Primary | CRISPRa | CD8 | IFNG | -0,021973 | 0,780635 |
| ZNF540 | Primary | CRISPRa | CD8 | IFNG | 0,012932 | 0,780635 |
| ZNF420 | Primary | CRISPRa | CD8 | IFNG | -0,064587 | 0,782146 |
| DPF2 | Primary | CRISPRa | CD8 | IFNG | -0,19832 | 0,784408 |
| ESX1 | Primary | CRISPRa | CD8 | IFNG | -0,40357 | 0,785206 |
| DLX1 | Primary | CRISPRa | CD8 | IFNG | -0,33964 | 0,785206 |
| DMRT3 | Primary | CRISPRa | CD8 | IFNG | -0,3214 | 0,785206 |
| BAZ1B | Primary | CRISPRa | CD8 | IFNG | -0,32 | 0,785206 |
| AFF1 | Primary | CRISPRa | CD8 | IFNG | -0,26127 | 0,785206 |
| POLR3GL | Primary | CRISPRa | CD8 | IFNG | -0,26112 | 0,785206 |
| HELLS | Primary | CRISPRa | CD8 | IFNG | -0,25131 | 0,785206 |
| EED | Primary | CRISPRa | CD8 | IFNG | -0,23243 | 0,785206 |
| HAND2 | Primary | CRISPRa | CD8 | IFNG | -0,22534 | 0,785206 |
| ABL1 | Primary | CRISPRa | CD8 | IFNG | -0,21814 | 0,785206 |
| ZDHHC19 | Primary | CRISPRa | CD8 | IFNG | -0,21631 | 0,785206 |
| SIRT5 | Primary | CRISPRa | CD8 | IFNG | -0,21074 | 0,785206 |
| MED14 | Primary | CRISPRa | CD8 | IFNG | -0,19168 | 0,785206 |
| HLF | Primary | CRISPRa | CD8 | IFNG | -0,17712 | 0,785206 |
| MAF1 | Primary | CRISPRa | CD8 | IFNG | -0,17634 | 0,785206 |
| ZNF230 | Primary | CRISPRa | CD8 | IFNG | -0,15712 | 0,785206 |
| CC2D1B | Primary | CRISPRa | CD8 | IFNG | -0,11234 | 0,785206 |
| GTF2F2 | Primary | CRISPRa | CD8 | IFNG | -0,11193 | 0,785206 |
| MCIDAS | Primary | CRISPRa | CD8 | IFNG | -0,10059 | 0,785206 |
| RALY | Primary | CRISPRa | CD8 | IFNG | -0,074991 | 0,785206 |
| POU4F1 | Primary | CRISPRa | CD8 | IFNG | -0,063718 | 0,785206 |
| NR5A1 | Primary | CRISPRa | CD8 | IFNG | -0,057065 | 0,785206 |
| ZNF684 | Primary | CRISPRa | CD8 | IFNG | -0,05349 | 0,785206 |
| ZNF182 | Primary | CRISPRa | CD8 | IFNG | -0,0088045 | 0,785206 |
| BRMS1L | Primary | CRISPRa | CD8 | IFNG | -0,0018743 | 0,785206 |
| TRIM72 | Primary | CRISPRa | CD8 | IFNG | -0,0017465 | 0,785206 |
| EYA4 | Primary | CRISPRa | CD8 | IFNG | 0,0067514 | 0,785206 |
| ERG | Primary | CRISPRa | CD8 | IFNG | 0,0095632 | 0,785206 |
| CCDC62 | Primary | CRISPRa | CD8 | IFNG | 0,011353 | 0,785206 |
| PAX3 | Primary | CRISPRa | CD8 | IFNG | 0,013765 | 0,785206 |
| EYA3 | Primary | CRISPRa | CD8 | IFNG | 0,016152 | 0,785206 |
| SUDS3 | Primary | CRISPRa | CD8 | IFNG | 0,049619 | 0,785206 |
| ZNF382 | Primary | CRISPRa | CD8 | IFNG | 0,073747 | 0,785206 |
| MLIP | Primary | CRISPRa | CD8 | IFNG | 0,19557 | 0,785206 |
| LYAR | Primary | CRISPRa | CD8 | IFNG | 0,1962 | 0,785206 |
| MED8 | Primary | CRISPRa | CD8 | IFNG | 0,1393 | 0,785354 |
| ZNF490 | Primary | CRISPRa | CD8 | IFNG | -0,1658 | 0,785698 |
| NOTCH2 | Primary | CRISPRa | CD8 | IFNG | -0,035507 | 0,78572 |
| ZMYM4 | Primary | CRISPRa | CD8 | IFNG | -0,10581 | 0,787408 |
| MLLT3 | Primary | CRISPRa | CD8 | IFNG | -0,052107 | 0,787408 |
| ARL2BP | Primary | CRISPRa | CD8 | IFNG | 0,0004693 | 0,787408 |
| PRAMEF18 | Primary | CRISPRa | CD8 | IFNG | 0,032625 | 0,787408 |
| ZIK1 | Primary | CRISPRa | CD8 | IFNG | 0,0927 | 0,787408 |
| MAMSTR | Primary | CRISPRa | CD8 | IFNG | 0,09666 | 0,787408 |
| EXOC2 | Primary | CRISPRa | CD8 | IFNG | 0,1042 | 0,787408 |
| SSBP3 | Primary | CRISPRa | CD8 | IFNG | 0,12856 | 0,787408 |
| LDB1 | Primary | CRISPRa | CD8 | IFNG | 0,26326 | 0,787408 |
| RPS6KA4 | Primary | CRISPRa | CD8 | IFNG | -0,10913 | 0,787549 |
| MAEL | Primary | CRISPRa | CD8 | IFNG | -0,13027 | 0,78871 |
| FOXJ3 | Primary | CRISPRa | CD8 | IFNG | 0,072689 | 0,78871 |
| RFX4 | Primary | CRISPRa | CD8 | IFNG | 0,00057775 | 0,789789 |
| ORC2 | Primary | CRISPRa | CD8 | IFNG | -0,13079 | 0,791421 |
| ZNF37A | Primary | CRISPRa | CD8 | IFNG | 0,11824 | 0,791421 |
| ING3 | Primary | CRISPRa | CD8 | IFNG | -0,19428 | 0,791528 |
| ZNF165 | Primary | CRISPRa | CD8 | IFNG | -0,133 | 0,791528 |
| SSX4 | Primary | CRISPRa | CD8 | IFNG | 0,0090038 | 0,791528 |
| NR3C1 | Primary | CRISPRa | CD8 | IFNG | 0,028891 | 0,791528 |
| TRIM49 | Primary | CRISPRa | CD8 | IFNG | -0,0887 | 0,793656 |
| HNRNPU | Primary | CRISPRa | CD8 | IFNG | -0,02902 | 0,793656 |
| MKRN3 | Primary | CRISPRa | CD8 | IFNG | 0,059246 | 0,793656 |
| CREB1 | Primary | CRISPRa | CD8 | IFNG | 0,099718 | 0,793656 |
| DCAF6 | Primary | CRISPRa | CD8 | IFNG | -0,17979 | 0,794536 |
| HDAC1 | Primary | CRISPRa | CD8 | IFNG | -0,16226 | 0,794536 |
| ZNF397 | Primary | CRISPRa | CD8 | IFNG | -0,10349 | 0,794536 |
| CHD8 | Primary | CRISPRa | CD8 | IFNG | -0,037727 | 0,794536 |
| EAF2 | Primary | CRISPRa | CD8 | IFNG | 0,013051 | 0,794536 |
| THAP6 | Primary | CRISPRa | CD8 | IFNG | -0,016283 | 0,797585 |
| SP5 | Primary | CRISPRa | CD8 | IFNG | 0,16251 | 0,798017 |
| POU2F3 | Primary | CRISPRa | CD8 | IFNG | 0,18955 | 0,798017 |
| SNW1 | Primary | CRISPRa | CD8 | IFNG | -0,1615 | 0,799482 |
| ZFP91 | Primary | CRISPRa | CD8 | IFNG | 0,08425 | 0,799482 |
| NRL | Primary | CRISPRa | CD8 | IFNG | 0,14227 | 0,799482 |
| SMYD5 | Primary | CRISPRa | CD8 | IFNG | -0,26263 | 0,799517 |
| NUFIP1 | Primary | CRISPRa | CD8 | IFNG | -0,23205 | 0,799517 |
| MED17 | Primary | CRISPRa | CD8 | IFNG | -0,17779 | 0,799517 |
| PLXNA3 | Primary | CRISPRa | CD8 | IFNG | -0,076574 | 0,799517 |
| TCF24 | Primary | CRISPRa | CD8 | IFNG | -0,049077 | 0,799517 |
| HOXB5 | Primary | CRISPRa | CD8 | IFNG | -0,20729 | 0,802615 |
| ZNF646 | Primary | CRISPRa | CD8 | IFNG | -0,17038 | 0,802615 |
| EGR3 | Primary | CRISPRa | CD8 | IFNG | -0,13656 | 0,802615 |
| BRF2 | Primary | CRISPRa | CD8 | IFNG | -0,11118 | 0,802615 |
| ZBTB12 | Primary | CRISPRa | CD8 | IFNG | -0,086639 | 0,802615 |
| ZNF609 | Primary | CRISPRa | CD8 | IFNG | -0,069317 | 0,802615 |
| TOP1 | Primary | CRISPRa | CD8 | IFNG | -0,064038 | 0,802615 |
| WDR5 | Primary | CRISPRa | CD8 | IFNG | -0,040882 | 0,802615 |
| JUNB | Primary | CRISPRa | CD8 | IFNG | -0,028903 | 0,802615 |
| INO80D | Primary | CRISPRa | CD8 | IFNG | -0,013714 | 0,802615 |
| ZNF783 | Primary | CRISPRa | CD8 | IFNG | 0,010901 | 0,802615 |
| ZNF708 | Primary | CRISPRa | CD8 | IFNG | 0,034611 | 0,802615 |
| WWC1 | Primary | CRISPRa | CD8 | IFNG | 0,041379 | 0,802615 |
| FOXP2 | Primary | CRISPRa | CD8 | IFNG | 0,068362 | 0,802615 |
| PKNOX2 | Primary | CRISPRa | CD8 | IFNG | 0,085172 | 0,802615 |
| LBX2 | Primary | CRISPRa | CD8 | IFNG | 0,092218 | 0,802615 |
| PKIA | Primary | CRISPRa | CD8 | IFNG | 0,13512 | 0,802615 |
| LRIF1 | Primary | CRISPRa | CD8 | IFNG | 0,19272 | 0,802615 |
| GLMP | Primary | CRISPRa | CD8 | IFNG | -0,096119 | 0,802964 |
| ZNF676 | Primary | CRISPRa | CD8 | IFNG | -0,014837 | 0,802964 |
| RBCK1 | Primary | CRISPRa | CD8 | IFNG | 0,02809 | 0,802964 |
| BEND3 | Primary | CRISPRa | CD8 | IFNG | 0,12848 | 0,802964 |
| STOX2 | Primary | CRISPRa | CD8 | IFNG | -0,022848 | 0,802975 |
| TRIM34 | Primary | CRISPRa | CD8 | IFNG | -0,01396 | 0,802975 |
| ZNF560 | Primary | CRISPRa | CD8 | IFNG | 0,022937 | 0,802975 |
| UBE2N | Primary | CRISPRa | CD8 | IFNG | 0,034638 | 0,802975 |
| DACH2 | Primary | CRISPRa | CD8 | IFNG | 0,094344 | 0,802975 |
| RECQL5 | Primary | CRISPRa | CD8 | IFNG | 0,18447 | 0,802975 |
| ASCL5 | Primary | CRISPRa | CD8 | IFNG | -0,26 | 0,803357 |
| PRAMEF4 | Primary | CRISPRa | CD8 | IFNG | -0,20204 | 0,803357 |
| ZNF462 | Primary | CRISPRa | CD8 | IFNG | -0,19064 | 0,803357 |
| NR1H2 | Primary | CRISPRa | CD8 | IFNG | -0,14189 | 0,803357 |
| ZNF131 | Primary | CRISPRa | CD8 | IFNG | -0,14144 | 0,803357 |
| ZNF205 | Primary | CRISPRa | CD8 | IFNG | -0,094685 | 0,803357 |
| PGR | Primary | CRISPRa | CD8 | IFNG | -0,057187 | 0,803357 |
| MED13 | Primary | CRISPRa | CD8 | IFNG | -0,027503 | 0,803357 |
| FOXP3 | Primary | CRISPRa | CD8 | IFNG | 0,0018384 | 0,803357 |
| ZFAT | Primary | CRISPRa | CD8 | IFNG | 0,062863 | 0,803357 |
| TFDP2 | Primary | CRISPRa | CD8 | IFNG | 0,15878 | 0,803389 |
| BAHCC1 | Primary | CRISPRa | CD8 | IFNG | -0,078727 | 0,803889 |
| HDAC2 | Primary | CRISPRa | CD8 | IFNG | 0,04419 | 0,803889 |
| ZNF157 | Primary | CRISPRa | CD8 | IFNG | 0,062966 | 0,803889 |
| SNCA | Primary | CRISPRa | CD8 | IFNG | 0,065172 | 0,803889 |
| USP39 | Primary | CRISPRa | CD8 | IFNG | -0,10772 | 0,804848 |
| PRAMEF14 | Primary | CRISPRa | CD8 | IFNG | 0,0027965 | 0,804848 |
| SAP18 | Primary | CRISPRa | CD8 | IFNG | -0,10838 | 0,805049 |
| KLHL13 | Primary | CRISPRa | CD8 | IFNG | -0,048663 | 0,805049 |
| MDM2 | Primary | CRISPRa | CD8 | IFNG | 0,10669 | 0,805049 |
| CDC73 | Primary | CRISPRa | CD8 | IFNG | -0,031478 | 0,808891 |
| PRAMEF7 | Primary | CRISPRa | CD8 | IFNG | -0,060701 | 0,809542 |
| LIMD1 | Primary | CRISPRa | CD8 | IFNG | -0,055729 | 0,809542 |
| ZNF830 | Primary | CRISPRa | CD8 | IFNG | 0,13697 | 0,809542 |
| ZBTB11 | Primary | CRISPRa | CD8 | IFNG | 0,095769 | 0,810055 |
| SLC30A9 | Primary | CRISPRa | CD8 | IFNG | 0,11262 | 0,810055 |
| PRMT1 | Primary | CRISPRa | CD8 | IFNG | 0,13494 | 0,810055 |
| UNCX | Primary | CRISPRa | CD8 | IFNG | -0,14158 | 0,810772 |
| PRAMEF13 | Primary | CRISPRa | CD8 | IFNG | -0,23918 | 0,81283 |
| HMG20B | Primary | CRISPRa | CD8 | IFNG | -0,22592 | 0,81283 |
| MEIS3 | Primary | CRISPRa | CD8 | IFNG | -0,13864 | 0,81283 |
| SMAD7 | Primary | CRISPRa | CD8 | IFNG | -0,094855 | 0,81283 |
| SPZ1 | Primary | CRISPRa | CD8 | IFNG | -0,088984 | 0,81283 |
| PSMC2 | Primary | CRISPRa | CD8 | IFNG | -0,060483 | 0,81283 |
| CSDC2 | Primary | CRISPRa | CD8 | IFNG | -0,036217 | 0,81283 |
| SIAH1 | Primary | CRISPRa | CD8 | IFNG | 0,057526 | 0,81283 |
| PHF20 | Primary | CRISPRa | CD8 | IFNG | 0,071075 | 0,81283 |
| ZAR1 | Primary | CRISPRa | CD8 | IFNG | -0,16399 | 0,812962 |
| DHRS7B | Primary | CRISPRa | CD8 | IFNG | -0,07227 | 0,812962 |
| NELFE | Primary | CRISPRa | CD8 | IFNG | 0,0082105 | 0,814401 |
| E2F2 | Primary | CRISPRa | CD8 | IFNG | -0,10717 | 0,815373 |
| ZNF274 | Primary | CRISPRa | CD8 | IFNG | -0,082053 | 0,815373 |
| FAM171B | Primary | CRISPRa | CD8 | IFNG | -0,06161 | 0,815373 |
| ZNF479 | Primary | CRISPRa | CD8 | IFNG | 0,207 | 0,815373 |
| ALX1 | Primary | CRISPRa | CD8 | IFNG | 0,25921 | 0,815373 |
| ZBTB22 | Primary | CRISPRa | CD8 | IFNG | -0,20303 | 0,81619 |
| ZNF711 | Primary | CRISPRa | CD8 | IFNG | -0,22829 | 0,817227 |
| FOXB1 | Primary | CRISPRa | CD8 | IFNG | -0,20863 | 0,817227 |
| TRIM40 | Primary | CRISPRa | CD8 | IFNG | -0,13996 | 0,817227 |
| KDM4D | Primary | CRISPRa | CD8 | IFNG | -0,12039 | 0,817227 |
| GTF3A | Primary | CRISPRa | CD8 | IFNG | -0,047866 | 0,817227 |
| ZNF432 | Primary | CRISPRa | CD8 | IFNG | -0,036833 | 0,817622 |
| CIITA | Primary | CRISPRa | CD8 | IFNG | 0,0067596 | 0,819876 |
| ZNF292 | Primary | CRISPRa | CD8 | IFNG | 0,10319 | 0,819876 |
| PELI1 | Primary | CRISPRa | CD8 | IFNG | -0,1309 | 0,821309 |
| ZNF117 | Primary | CRISPRa | CD8 | IFNG | -0,021038 | 0,821309 |
| HOXD12 | Primary | CRISPRa | CD8 | IFNG | 0,073928 | 0,821309 |
| ZNF98 | Primary | CRISPRa | CD8 | IFNG | 0,075225 | 0,821309 |
| THAP2 | Primary | CRISPRa | CD8 | IFNG | -0,047616 | 0,824441 |
| ZNF177 | Primary | CRISPRa | CD8 | IFNG | -0,038969 | 0,824441 |
| L3MBTL3 | Primary | CRISPRa | CD8 | IFNG | -0,082817 | 0,826335 |
| SETD3 | Primary | CRISPRa | CD8 | IFNG | -0,092425 | 0,826408 |
| NUDT5 | Primary | CRISPRa | CD8 | IFNG | 0,018344 | 0,826408 |
| HBP1 | Primary | CRISPRa | CD8 | IFNG | -0,060757 | 0,828957 |
| BASP1 | Primary | CRISPRa | CD8 | IFNG | -0,15428 | 0,829672 |
| PTEN | Primary | CRISPRa | CD8 | IFNG | -0,064219 | 0,830528 |
| NAA16 | Primary | CRISPRa | CD8 | IFNG | 0,015369 | 0,830528 |
| ZNF626 | Primary | CRISPRa | CD8 | IFNG | -0,2175 | 0,830777 |
| PSPC1 | Primary | CRISPRa | CD8 | IFNG | -0,13605 | 0,830777 |
| COPS2 | Primary | CRISPRa | CD8 | IFNG | 0,0022683 | 0,830777 |
| FOSL2 | Primary | CRISPRa | CD8 | IFNG | 0,021164 | 0,830777 |
| TRIP6 | Primary | CRISPRa | CD8 | IFNG | 0,042736 | 0,830777 |
| LIN28B | Primary | CRISPRa | CD8 | IFNG | 0,084873 | 0,830777 |
| CCNT1 | Primary | CRISPRa | CD8 | IFNG | 0,15955 | 0,830777 |
| ZFP2 | Primary | CRISPRa | CD8 | IFNG | -0,30849 | 0,836616 |
| UNK | Primary | CRISPRa | CD8 | IFNG | -0,26737 | 0,836616 |
| PHOX2B | Primary | CRISPRa | CD8 | IFNG | -0,2401 | 0,836616 |
| RASSF7 | Primary | CRISPRa | CD8 | IFNG | -0,13865 | 0,836616 |
| NPAS3 | Primary | CRISPRa | CD8 | IFNG | -0,1284 | 0,836616 |
| ZNF154 | Primary | CRISPRa | CD8 | IFNG | 0,026642 | 0,836616 |
| TRIM39 | Primary | CRISPRa | CD8 | IFNG | -0,17904 | 0,836705 |
| HSPA8 | Primary | CRISPRa | CD8 | IFNG | -0,072079 | 0,836705 |
| ZNF248 | Primary | CRISPRa | CD8 | IFNG | -0,023754 | 0,836705 |
| CHD5 | Primary | CRISPRa | CD8 | IFNG | 0,17974 | 0,836705 |
| HES2 | Primary | CRISPRa | CD8 | IFNG | 0,12435 | 0,838409 |
| CSNK2A1 | Primary | CRISPRa | CD8 | IFNG | 0,27405 | 0,838409 |
| NKX2-4 | Primary | CRISPRa | CD8 | IFNG | -0,039185 | 0,838613 |
| DDX17 | Primary | CRISPRa | CD8 | IFNG | 0,07467 | 0,838849 |
| NCALD | Primary | CRISPRa | CD8 | IFNG | -0,074841 | 0,842733 |
| BTBD1 | Primary | CRISPRa | CD8 | IFNG | -0,064594 | 0,842733 |
| NR2C2 | Primary | CRISPRa | CD8 | IFNG | -0,12857 | 0,843561 |
| BNC2 | Primary | CRISPRa | CD8 | IFNG | -0,006 | 0,843561 |
| EHMT2 | Primary | CRISPRa | CD8 | IFNG | 0,03135 | 0,843561 |
| ZNF839 | Primary | CRISPRa | CD8 | IFNG | -0,25066 | 0,844191 |
| HSBP1L1 | Primary | CRISPRa | CD8 | IFNG | -0,11154 | 0,844191 |
| CTDSP1 | Primary | CRISPRa | CD8 | IFNG | -0,10073 | 0,844191 |
| POLE3 | Primary | CRISPRa | CD8 | IFNG | -0,020138 | 0,844191 |
| SPIC | Primary | CRISPRa | CD8 | IFNG | 0,0057831 | 0,844191 |
| ZNF780B | Primary | CRISPRa | CD8 | IFNG | 0,02152 | 0,844191 |
| PCGF5 | Primary | CRISPRa | CD8 | IFNG | 0,03411 | 0,844191 |
| RGCC | Primary | CRISPRa | CD8 | IFNG | 0,053991 | 0,844191 |
| EID3 | Primary | CRISPRa | CD8 | IFNG | 0,078106 | 0,844191 |
| SKOR2 | Primary | CRISPRa | CD8 | IFNG | 0,080391 | 0,844191 |
| NR1I3 | Primary | CRISPRa | CD8 | IFNG | 0,096176 | 0,844191 |
| CDK5R1 | Primary | CRISPRa | CD8 | IFNG | 0,11271 | 0,844191 |
| PCGF6 | Primary | CRISPRa | CD8 | IFNG | 0,11306 | 0,844191 |
| NUFIP2 | Primary | CRISPRa | CD8 | IFNG | 0,22736 | 0,844191 |
| NELFB | Primary | CRISPRa | CD8 | IFNG | -0,29679 | 0,844374 |
| TAF6 | Primary | CRISPRa | CD8 | IFNG | -0,23623 | 0,844374 |
| NUP85 | Primary | CRISPRa | CD8 | IFNG | -0,23201 | 0,844374 |
| CBX2 | Primary | CRISPRa | CD8 | IFNG | -0,10998 | 0,844374 |
| MYF6 | Primary | CRISPRa | CD8 | IFNG | -0,037127 | 0,844374 |
| JADE2 | Primary | CRISPRa | CD8 | IFNG | -0,026713 | 0,844374 |
| ZNF366 | Primary | CRISPRa | CD8 | IFNG | 0,0015313 | 0,844374 |
| ZFP82 | Primary | CRISPRa | CD8 | IFNG | 0,0091446 | 0,844374 |
| HMGXB4 | Primary | CRISPRa | CD8 | IFNG | 0,035793 | 0,844374 |
| ZNF133 | Primary | CRISPRa | CD8 | IFNG | -0,3807 | 0,845241 |
| ZNF197 | Primary | CRISPRa | CD8 | IFNG | -0,16447 | 0,845241 |
| ZNF285 | Primary | CRISPRa | CD8 | IFNG | -0,13728 | 0,845241 |
| CEBPZ | Primary | CRISPRa | CD8 | IFNG | -0,11365 | 0,845241 |
| ZNF143 | Primary | CRISPRa | CD8 | IFNG | -0,05895 | 0,845241 |
| TP53BP1 | Primary | CRISPRa | CD8 | IFNG | -0,022286 | 0,845241 |
| HMGXB3 | Primary | CRISPRa | CD8 | IFNG | 0,062402 | 0,845241 |
| RFX3 | Primary | CRISPRa | CD8 | IFNG | 0,095 | 0,845241 |
| ZNF350 | Primary | CRISPRa | CD8 | IFNG | -0,14448 | 0,84536 |
| TBX22 | Primary | CRISPRa | CD8 | IFNG | -0,061395 | 0,84536 |
| ITCH | Primary | CRISPRa | CD8 | IFNG | -0,31999 | 0,84673 |
| MYC | Primary | CRISPRa | CD8 | IFNG | -0,27153 | 0,84673 |
| SLC6A3 | Primary | CRISPRa | CD8 | IFNG | -0,27149 | 0,84673 |
| ZNF28 | Primary | CRISPRa | CD8 | IFNG | -0,17399 | 0,84673 |
| ZNF695 | Primary | CRISPRa | CD8 | IFNG | -0,15952 | 0,84673 |
| SIRT6 | Primary | CRISPRa | CD8 | IFNG | -0,10536 | 0,84673 |
| BATF2 | Primary | CRISPRa | CD8 | IFNG | -0,0959 | 0,84673 |
| DGKQ | Primary | CRISPRa | CD8 | IFNG | -0,074656 | 0,84673 |
| DVL1 | Primary | CRISPRa | CD8 | IFNG | -0,05473 | 0,84673 |
| OTX1 | Primary | CRISPRa | CD8 | IFNG | -0,047232 | 0,84673 |
| ZNF383 | Primary | CRISPRa | CD8 | IFNG | -0,23727 | 0,848595 |
| TEAD2 | Primary | CRISPRa | CD8 | IFNG | -0,094446 | 0,848595 |
| SETD2 | Primary | CRISPRa | CD8 | IFNG | -0,13728 | 0,848908 |
| TBX4 | Primary | CRISPRa | CD8 | IFNG | 0,12142 | 0,848908 |
| CRTC2 | Primary | CRISPRa | CD8 | IFNG | -0,19264 | 0,851068 |
| TCFL5 | Primary | CRISPRa | CD8 | IFNG | -0,0028274 | 0,852501 |
| TDP2 | Primary | CRISPRa | CD8 | IFNG | 0,094119 | 0,852501 |
| PSMD10 | Primary | CRISPRa | CD8 | IFNG | -0,22306 | 0,854407 |
| TAF1B | Primary | CRISPRa | CD8 | IFNG | -0,10625 | 0,854407 |
| ZNF470 | Primary | CRISPRa | CD8 | IFNG | -0,1036 | 0,854407 |
| ZNF174 | Primary | CRISPRa | CD8 | IFNG | -0,08972 | 0,854407 |
| PRDM4 | Primary | CRISPRa | CD8 | IFNG | -0,073002 | 0,854407 |
| HNF4G | Primary | CRISPRa | CD8 | IFNG | -0,067034 | 0,854407 |
| ZNF613 | Primary | CRISPRa | CD8 | IFNG | -0,052452 | 0,854407 |
| MSGN1 | Primary | CRISPRa | CD8 | IFNG | -0,034993 | 0,854407 |
| ZNF821 | Primary | CRISPRa | CD8 | IFNG | -0,031727 | 0,854407 |
| L3MBTL1 | Primary | CRISPRa | CD8 | IFNG | -0,0043395 | 0,854407 |
| ZNF790 | Primary | CRISPRa | CD8 | IFNG | 0,0021176 | 0,854407 |
| BLOC1S1 | Primary | CRISPRa | CD8 | IFNG | 0,010753 | 0,854407 |
| CDK11B | Primary | CRISPRa | CD8 | IFNG | 0,022606 | 0,854407 |
| ZNF567 | Primary | CRISPRa | CD8 | IFNG | 0,056877 | 0,854407 |
| CBX7 | Primary | CRISPRa | CD8 | IFNG | 0,060135 | 0,854407 |
| TADA2A | Primary | CRISPRa | CD8 | IFNG | 0,070549 | 0,854407 |
| ZNF229 | Primary | CRISPRa | CD8 | IFNG | 0,077528 | 0,854407 |
| ZNF286B | Primary | CRISPRa | CD8 | IFNG | 0,11568 | 0,854407 |
| ZNF442 | Primary | CRISPRa | CD8 | IFNG | 0,12114 | 0,854407 |
| RNF168 | Primary | CRISPRa | CD8 | IFNG | 0,15142 | 0,854407 |
| GTF2E2 | Primary | CRISPRa | CD8 | IFNG | 0,1628 | 0,854407 |
| FOXD4L5 | Primary | CRISPRa | CD8 | IFNG | 0,16444 | 0,854407 |
| EGR1 | Primary | CRISPRa | CD8 | IFNG | 0,18012 | 0,854407 |
| ZNF426 | Primary | CRISPRa | CD8 | IFNG | 0,20533 | 0,854407 |
| BCL11A | Primary | CRISPRa | CD8 | IFNG | 0,20735 | 0,854407 |
| APBB1 | Primary | CRISPRa | CD8 | IFNG | 0,0038498 | 0,854785 |
| PUF60 | Primary | CRISPRa | CD8 | IFNG | 0,23425 | 0,856453 |
| ZMYND11 | Primary | CRISPRa | CD8 | IFNG | 0,060321 | 0,857171 |
| OPTN | Primary | CRISPRa | CD8 | IFNG | -0,087435 | 0,857888 |
| ELK1 | Primary | CRISPRa | CD8 | IFNG | 0,086858 | 0,857888 |
| FOXO1 | Primary | CRISPRa | CD8 | IFNG | -0,22392 | 0,858538 |
| ZNF75A | Primary | CRISPRa | CD8 | IFNG | -0,0012819 | 0,858538 |
| ZBED2 | Primary | CRISPRa | CD8 | IFNG | 0,024362 | 0,861455 |
| BRCA1 | Primary | CRISPRa | CD8 | IFNG | -0,34263 | 0,861638 |
| ILK | Primary | CRISPRa | CD8 | IFNG | -0,073377 | 0,861638 |
| IKBKG | Primary | CRISPRa | CD8 | IFNG | -0,26112 | 0,862287 |
| INSM1 | Primary | CRISPRa | CD8 | IFNG | -0,16137 | 0,862287 |
| SAP130 | Primary | CRISPRa | CD8 | IFNG | -0,12789 | 0,862287 |
| PLEKHA4 | Primary | CRISPRa | CD8 | IFNG | -0,093787 | 0,862287 |
| SETD1A | Primary | CRISPRa | CD8 | IFNG | -0,068599 | 0,862287 |
| INO80 | Primary | CRISPRa | CD8 | IFNG | -0,039813 | 0,862287 |
| TAF2 | Primary | CRISPRa | CD8 | IFNG | -0,024982 | 0,862287 |
| HOXA3 | Primary | CRISPRa | CD8 | IFNG | 0,047933 | 0,862287 |
| PEX2 | Primary | CRISPRa | CD8 | IFNG | -0,23493 | 0,862289 |
| FUBP1 | Primary | CRISPRa | CD8 | IFNG | -0,14051 | 0,862289 |
| SCML1 | Primary | CRISPRa | CD8 | IFNG | 0,093193 | 0,862289 |
| VDR | Primary | CRISPRa | CD8 | IFNG | -0,23894 | 0,86499 |
| ZFR | Primary | CRISPRa | CD8 | IFNG | -0,17878 | 0,86499 |
| TARDBP | Primary | CRISPRa | CD8 | IFNG | -0,17516 | 0,86499 |
| CNOT10 | Primary | CRISPRa | CD8 | IFNG | -0,16372 | 0,86499 |
| ZNF335 | Primary | CRISPRa | CD8 | IFNG | -0,15051 | 0,86499 |
| HSF5 | Primary | CRISPRa | CD8 | IFNG | -0,14947 | 0,86499 |
| PKN2 | Primary | CRISPRa | CD8 | IFNG | -0,029083 | 0,86499 |
| HSBP1 | Primary | CRISPRa | CD8 | IFNG | 0,023322 | 0,86499 |
| GTF2A1L | Primary | CRISPRa | CD8 | IFNG | 0,029167 | 0,86499 |
| KLF5 | Primary | CRISPRa | CD8 | IFNG | 0,041352 | 0,86499 |
| ZKSCAN3 | Primary | CRISPRa | CD8 | IFNG | 0,10072 | 0,86499 |
| ZNF121 | Primary | CRISPRa | CD8 | IFNG | -0,010544 | 0,867509 |
| BDP1 | Primary | CRISPRa | CD8 | IFNG | -0,19769 | 0,868199 |
| MAPK3 | Primary | CRISPRa | CD8 | IFNG | -0,0064171 | 0,868199 |
| ZSCAN32 | Primary | CRISPRa | CD8 | IFNG | 0,0031454 | 0,868199 |
| PEX14 | Primary | CRISPRa | CD8 | IFNG | 0,0067451 | 0,868199 |
| PCBP3 | Primary | CRISPRa | CD8 | IFNG | -0,098493 | 0,869481 |
| INO80E | Primary | CRISPRa | CD8 | IFNG | 0,022672 | 0,869481 |
| RNF113B | Primary | CRISPRa | CD8 | IFNG | 0,037401 | 0,87015 |
| ASF1A | Primary | CRISPRa | CD8 | IFNG | 0,085421 | 0,87015 |
| ZNF552 | Primary | CRISPRa | CD8 | IFNG | 0,12397 | 0,872974 |
| ZNF273 | Primary | CRISPRa | CD8 | IFNG | -0,19849 | 0,876503 |
| HOXC9 | Primary | CRISPRa | CD8 | IFNG | -0,16819 | 0,876503 |
| CDR2L | Primary | CRISPRa | CD8 | IFNG | -0,14262 | 0,876503 |
| ZNF311 | Primary | CRISPRa | CD8 | IFNG | -0,11054 | 0,876503 |
| SHPRH | Primary | CRISPRa | CD8 | IFNG | -0,070418 | 0,876503 |
| CRY1 | Primary | CRISPRa | CD8 | IFNG | -0,051831 | 0,876503 |
| HOXA9 | Primary | CRISPRa | CD8 | IFNG | -0,047001 | 0,876503 |
| SOX3 | Primary | CRISPRa | CD8 | IFNG | -0,028039 | 0,876503 |
| ZNF493 | Primary | CRISPRa | CD8 | IFNG | -0,013669 | 0,876503 |
| CNOT2 | Primary | CRISPRa | CD8 | IFNG | 0,015061 | 0,876503 |
| ZNF827 | Primary | CRISPRa | CD8 | IFNG | 0,075385 | 0,876503 |
| CTNNB1 | Primary | CRISPRa | CD8 | IFNG | 0,075464 | 0,876503 |
| PAX9 | Primary | CRISPRa | CD8 | IFNG | 0,079971 | 0,876503 |
| ZNF799 | Primary | CRISPRa | CD8 | IFNG | 0,085232 | 0,876503 |
| NKX1-2 | Primary | CRISPRa | CD8 | IFNG | 0,10241 | 0,876503 |
| ZNF184 | Primary | CRISPRa | CD8 | IFNG | 0,11135 | 0,876503 |
| HEATR1 | Primary | CRISPRa | CD8 | IFNG | 0,12442 | 0,876503 |
| ZXDC | Primary | CRISPRa | CD8 | IFNG | 0,14255 | 0,876503 |
| RCVRN | Primary | CRISPRa | CD8 | IFNG | 0,17015 | 0,876503 |
| SMAD5 | Primary | CRISPRa | CD8 | IFNG | 0,18601 | 0,876503 |
| HES1 | Primary | CRISPRa | CD8 | IFNG | 0,20467 | 0,876503 |
| DMAP1 | Primary | CRISPRa | CD8 | IFNG | 0,22403 | 0,876503 |
| ADRB2 | Primary | CRISPRa | CD8 | IFNG | 0,23553 | 0,876503 |
| HOXC5 | Primary | CRISPRa | CD8 | IFNG | 0,2566 | 0,876503 |
| RFX2 | Primary | CRISPRa | CD8 | IFNG | -0,17393 | 0,876711 |
| NFYC | Primary | CRISPRa | CD8 | IFNG | -0,16396 | 0,876711 |
| PRAMEF1 | Primary | CRISPRa | CD8 | IFNG | -0,13444 | 0,876711 |
| CPSF4 | Primary | CRISPRa | CD8 | IFNG | 0,0030195 | 0,876711 |
| ZNF76 | Primary | CRISPRa | CD8 | IFNG | 0,041037 | 0,876711 |
| ESRRG | Primary | CRISPRa | CD8 | IFNG | 0,049986 | 0,876711 |
| MAD2L2 | Primary | CRISPRa | CD8 | IFNG | 0,19605 | 0,876711 |
| HNRNPUL1 | Primary | CRISPRa | CD8 | IFNG | -0,27332 | 0,878841 |
| NFYA | Primary | CRISPRa | CD8 | IFNG | -0,19887 | 0,878841 |
| CUX1 | Primary | CRISPRa | CD8 | IFNG | -0,19725 | 0,878841 |
| ZNF616 | Primary | CRISPRa | CD8 | IFNG | -0,18181 | 0,878841 |
| ZNF581 | Primary | CRISPRa | CD8 | IFNG | -0,17694 | 0,878841 |
| NDUFA13 | Primary | CRISPRa | CD8 | IFNG | -0,17242 | 0,878841 |
| EXOSC9 | Primary | CRISPRa | CD8 | IFNG | -0,17208 | 0,878841 |
| ZNF770 | Primary | CRISPRa | CD8 | IFNG | -0,16044 | 0,878841 |
| DDX39B | Primary | CRISPRa | CD8 | IFNG | -0,1569 | 0,878841 |
| LRRK2 | Primary | CRISPRa | CD8 | IFNG | -0,15145 | 0,878841 |
| MYBBP1A | Primary | CRISPRa | CD8 | IFNG | -0,14091 | 0,878841 |
| ZNF574 | Primary | CRISPRa | CD8 | IFNG | -0,13166 | 0,878841 |
| PLXNB2 | Primary | CRISPRa | CD8 | IFNG | -0,129 | 0,878841 |
| LTF | Primary | CRISPRa | CD8 | IFNG | -0,12566 | 0,878841 |
| ZIC3 | Primary | CRISPRa | CD8 | IFNG | -0,12163 | 0,878841 |
| ATXN3L | Primary | CRISPRa | CD8 | IFNG | -0,12093 | 0,878841 |
| NLRP12 | Primary | CRISPRa | CD8 | IFNG | -0,11834 | 0,878841 |
| ELP4 | Primary | CRISPRa | CD8 | IFNG | -0,10559 | 0,878841 |
| NR5A2 | Primary | CRISPRa | CD8 | IFNG | -0,080752 | 0,878841 |
| RBM26 | Primary | CRISPRa | CD8 | IFNG | -0,036995 | 0,878841 |
| BIRC5 | Primary | CRISPRa | CD8 | IFNG | -0,030857 | 0,878841 |
| UBE2D2 | Primary | CRISPRa | CD8 | IFNG | -0,029437 | 0,878841 |
| SOX14 | Primary | CRISPRa | CD8 | IFNG | -0,0052864 | 0,878841 |
| L3MBTL2 | Primary | CRISPRa | CD8 | IFNG | -0,0050707 | 0,878841 |
| MKRN1 | Primary | CRISPRa | CD8 | IFNG | 0,013081 | 0,878841 |
| RBM6 | Primary | CRISPRa | CD8 | IFNG | 0,021082 | 0,878841 |
| ZNF771 | Primary | CRISPRa | CD8 | IFNG | 0,021298 | 0,878841 |
| FER | Primary | CRISPRa | CD8 | IFNG | 0,035902 | 0,878841 |
| JAZF1 | Primary | CRISPRa | CD8 | IFNG | 0,060826 | 0,878841 |
| TBPL2 | Primary | CRISPRa | CD8 | IFNG | 0,082616 | 0,878841 |
| TRIM11 | Primary | CRISPRa | CD8 | IFNG | 0,11574 | 0,878841 |
| BATF3 | Primary | CRISPRa | CD8 | IFNG | 0,12578 | 0,878841 |
| HOXC11 | Primary | CRISPRa | CD8 | IFNG | 0,14491 | 0,878841 |
| IGHMBP2 | Primary | CRISPRa | CD8 | IFNG | 0,16438 | 0,878841 |
| OVOL3 | Primary | CRISPRa | CD8 | IFNG | 0,18165 | 0,878841 |
| HNRNPC | Primary | CRISPRa | CD8 | IFNG | 0,23517 | 0,878841 |
| MBTD1 | Primary | CRISPRa | CD8 | IFNG | 0,12231 | 0,879319 |
| WDTC1 | Primary | CRISPRa | CD8 | IFNG | -0,067879 | 0,879748 |
| KIN | Primary | CRISPRa | CD8 | IFNG | 0,077888 | 0,879748 |
| CRY2 | Primary | CRISPRa | CD8 | IFNG | -0,28709 | 0,880705 |
| EEA1 | Primary | CRISPRa | CD8 | IFNG | -0,24155 | 0,880705 |
| HIP1 | Primary | CRISPRa | CD8 | IFNG | -0,1688 | 0,880705 |
| CIDEA | Primary | CRISPRa | CD8 | IFNG | -0,11868 | 0,880705 |
| DEPDC1B | Primary | CRISPRa | CD8 | IFNG | -0,0005119 | 0,880705 |
| RHOA | Primary | CRISPRa | CD8 | IFNG | 0,026346 | 0,880705 |
| RNF13 | Primary | CRISPRa | CD8 | IFNG | -0,20072 | 0,881231 |
| AFF3 | Primary | CRISPRa | CD8 | IFNG | 0,14866 | 0,881334 |
| PRRX2 | Primary | CRISPRa | CD8 | IFNG | -0,28958 | 0,88135 |
| SUPT7L | Primary | CRISPRa | CD8 | IFNG | -0,086177 | 0,881888 |
| AR | Primary | CRISPRa | CD8 | IFNG | -0,20093 | 0,883502 |
| ZSCAN26 | Primary | CRISPRa | CD8 | IFNG | -0,19887 | 0,883502 |
| IRAK2 | Primary | CRISPRa | CD8 | IFNG | -0,1959 | 0,883502 |
| ZNF146 | Primary | CRISPRa | CD8 | IFNG | -0,14872 | 0,883502 |
| HINFP | Primary | CRISPRa | CD8 | IFNG | -0,11587 | 0,883502 |
| PHF5A | Primary | CRISPRa | CD8 | IFNG | -0,10456 | 0,883502 |
| ZNF728 | Primary | CRISPRa | CD8 | IFNG | -0,10393 | 0,883502 |
| LARP7 | Primary | CRISPRa | CD8 | IFNG | -0,19638 | 0,883969 |
| ZNF529 | Primary | CRISPRa | CD8 | IFNG | -0,070457 | 0,884425 |
| ZNF705E | Primary | CRISPRa | CD8 | IFNG | -0,066624 | 0,884425 |
| DOT1L | Primary | CRISPRa | CD8 | IFNG | -0,060029 | 0,884425 |
| DMRTC1B | Primary | CRISPRa | CD8 | IFNG | -0,033316 | 0,884425 |
| ALYREF | Primary | CRISPRa | CD8 | IFNG | -0,031536 | 0,884425 |
| AXIN1 | Primary | CRISPRa | CD8 | IFNG | 0,036121 | 0,884425 |
| RAX | Primary | CRISPRa | CD8 | IFNG | 0,037174 | 0,884425 |
| ELL3 | Primary | CRISPRa | CD8 | IFNG | 0,038641 | 0,884425 |
| ESF1 | Primary | CRISPRa | CD8 | IFNG | 0,045563 | 0,884425 |
| RNF6 | Primary | CRISPRa | CD8 | IFNG | 0,049574 | 0,884425 |
| TBP | Primary | CRISPRa | CD8 | IFNG | 0,055286 | 0,884425 |
| PXN | Primary | CRISPRa | CD8 | IFNG | 0,063128 | 0,884425 |
| ERBB4 | Primary | CRISPRa | CD8 | IFNG | 0,071616 | 0,884425 |
| BHLHA15 | Primary | CRISPRa | CD8 | IFNG | 0,073935 | 0,884425 |
| ZZZ3 | Primary | CRISPRa | CD8 | IFNG | 0,077301 | 0,884425 |
| CDK5RAP3 | Primary | CRISPRa | CD8 | IFNG | 0,080527 | 0,884425 |
| ALPK3 | Primary | CRISPRa | CD8 | IFNG | 0,082153 | 0,884425 |
| GLI4 | Primary | CRISPRa | CD8 | IFNG | 0,10298 | 0,884425 |
| KRBA1 | Primary | CRISPRa | CD8 | IFNG | 0,11178 | 0,884425 |
| FHIT | Primary | CRISPRa | CD8 | IFNG | 0,131 | 0,884425 |
| ZNF224 | Primary | CRISPRa | CD8 | IFNG | 0,13147 | 0,884425 |
| IKZF2 | Primary | CRISPRa | CD8 | IFNG | 0,1824 | 0,884425 |
| ZNF763 | Primary | CRISPRa | CD8 | IFNG | 0,18764 | 0,884425 |
| MED19 | Primary | CRISPRa | CD8 | IFNG | 0,20292 | 0,884425 |
| KLF11 | Primary | CRISPRa | CD8 | IFNG | 0,089308 | 0,884562 |
| MEFV | Primary | CRISPRa | CD8 | IFNG | -0,051985 | 0,885289 |
| LHX4 | Primary | CRISPRa | CD8 | IFNG | -0,04059 | 0,885643 |
| NR3C2 | Primary | CRISPRa | CD8 | IFNG | -0,12303 | 0,885932 |
| ZNF699 | Primary | CRISPRa | CD8 | IFNG | 0,081582 | 0,885932 |
| COMMD6 | Primary | CRISPRa | CD8 | IFNG | 0,11054 | 0,885932 |
| TRAK2 | Primary | CRISPRa | CD8 | IFNG | -0,21678 | 0,886883 |
| ZNF195 | Primary | CRISPRa | CD8 | IFNG | -0,2194 | 0,88735 |
| ZNF415 | Primary | CRISPRa | CD8 | IFNG | 0,094432 | 0,88735 |
| UBTFL1 | Primary | CRISPRa | CD8 | IFNG | -0,21515 | 0,887383 |
| PHOX2A | Primary | CRISPRa | CD8 | IFNG | -0,19077 | 0,887383 |
| TBL1X | Primary | CRISPRa | CD8 | IFNG | 0,0076599 | 0,887383 |
| GRHL3 | Primary | CRISPRa | CD8 | IFNG | -0,12701 | 0,887922 |
| HOXA5 | Primary | CRISPRa | CD8 | IFNG | -0,073525 | 0,888366 |
| SMARCC1 | Primary | CRISPRa | CD8 | IFNG | 0,00499 | 0,888366 |
| STAT3 | Primary | CRISPRa | CD8 | IFNG | -0,024576 | 0,888574 |
| TLE6 | Primary | CRISPRa | CD8 | IFNG | 0,0042295 | 0,888574 |
| NEUROG1 | Primary | CRISPRa | CD8 | IFNG | 0,074924 | 0,888574 |
| ZFP62 | Primary | CRISPRa | CD8 | IFNG | -0,29559 | 0,889039 |
| MBD3 | Primary | CRISPRa | CD8 | IFNG | -0,27037 | 0,889039 |
| ZNF428 | Primary | CRISPRa | CD8 | IFNG | -0,25275 | 0,889039 |
| PRAMEF8 | Primary | CRISPRa | CD8 | IFNG | -0,23169 | 0,889039 |
| ZNF19 | Primary | CRISPRa | CD8 | IFNG | -0,22936 | 0,889039 |
| IRF2BPL | Primary | CRISPRa | CD8 | IFNG | -0,22383 | 0,889039 |
| TSTD1 | Primary | CRISPRa | CD8 | IFNG | -0,19361 | 0,889039 |
| NRIP1 | Primary | CRISPRa | CD8 | IFNG | -0,18925 | 0,889039 |
| CTDP1 | Primary | CRISPRa | CD8 | IFNG | -0,17816 | 0,889039 |
| NACA2 | Primary | CRISPRa | CD8 | IFNG | -0,17511 | 0,889039 |
| THRAP3 | Primary | CRISPRa | CD8 | IFNG | -0,17214 | 0,889039 |
| ZNF837 | Primary | CRISPRa | CD8 | IFNG | -0,17071 | 0,889039 |
| MED16 | Primary | CRISPRa | CD8 | IFNG | -0,16608 | 0,889039 |
| ESR2 | Primary | CRISPRa | CD8 | IFNG | -0,1648 | 0,889039 |
| CASK | Primary | CRISPRa | CD8 | IFNG | -0,16326 | 0,889039 |
| MAPK8IP1 | Primary | CRISPRa | CD8 | IFNG | -0,1628 | 0,889039 |
| HOXA13 | Primary | CRISPRa | CD8 | IFNG | -0,16275 | 0,889039 |
| CDX4 | Primary | CRISPRa | CD8 | IFNG | -0,16222 | 0,889039 |
| DUS3L | Primary | CRISPRa | CD8 | IFNG | -0,15423 | 0,889039 |
| CTNND1 | Primary | CRISPRa | CD8 | IFNG | -0,15163 | 0,889039 |
| TCF20 | Primary | CRISPRa | CD8 | IFNG | -0,14368 | 0,889039 |
| CRYM | Primary | CRISPRa | CD8 | IFNG | -0,14132 | 0,889039 |
| ZNF627 | Primary | CRISPRa | CD8 | IFNG | -0,14124 | 0,889039 |
| SUPT20HL2 | Primary | CRISPRa | CD8 | IFNG | -0,13769 | 0,889039 |
| PRDM15 | Primary | CRISPRa | CD8 | IFNG | -0,12602 | 0,889039 |
| ETV5 | Primary | CRISPRa | CD8 | IFNG | -0,12528 | 0,889039 |
| CNOT9 | Primary | CRISPRa | CD8 | IFNG | -0,11804 | 0,889039 |
| ZNF169 | Primary | CRISPRa | CD8 | IFNG | -0,11382 | 0,889039 |
| ZNF696 | Primary | CRISPRa | CD8 | IFNG | -0,11126 | 0,889039 |
| HOXB4 | Primary | CRISPRa | CD8 | IFNG | -0,10998 | 0,889039 |
| THAP7 | Primary | CRISPRa | CD8 | IFNG | -0,10915 | 0,889039 |
| PABPC1L | Primary | CRISPRa | CD8 | IFNG | -0,095395 | 0,889039 |
| CTNND2 | Primary | CRISPRa | CD8 | IFNG | -0,093473 | 0,889039 |
| ICE2 | Primary | CRISPRa | CD8 | IFNG | -0,083035 | 0,889039 |
| HMGB2 | Primary | CRISPRa | CD8 | IFNG | -0,066062 | 0,889039 |
| POU3F2 | Primary | CRISPRa | CD8 | IFNG | -0,06334 | 0,889039 |
| ZNF282 | Primary | CRISPRa | CD8 | IFNG | -0,05609 | 0,889039 |
| CCNC | Primary | CRISPRa | CD8 | IFNG | -0,029585 | 0,889039 |
| MED28 | Primary | CRISPRa | CD8 | IFNG | -0,027227 | 0,889039 |
| ZNF645 | Primary | CRISPRa | CD8 | IFNG | -0,02488 | 0,889039 |
| SOX1 | Primary | CRISPRa | CD8 | IFNG | -0,018566 | 0,889039 |
| UBR2 | Primary | CRISPRa | CD8 | IFNG | -0,018199 | 0,889039 |
| SLC39A10 | Primary | CRISPRa | CD8 | IFNG | -0,016043 | 0,889039 |
| ZNF232 | Primary | CRISPRa | CD8 | IFNG | -0,013639 | 0,889039 |
| PTF1A | Primary | CRISPRa | CD8 | IFNG | -0,013531 | 0,889039 |
| HEY2 | Primary | CRISPRa | CD8 | IFNG | -0,01293 | 0,889039 |
| NANOGNB | Primary | CRISPRa | CD8 | IFNG | 0,0039912 | 0,889039 |
| TMF1 | Primary | CRISPRa | CD8 | IFNG | 0,017233 | 0,889039 |
| ZNF391 | Primary | CRISPRa | CD8 | IFNG | 0,019496 | 0,889039 |
| TAF7L | Primary | CRISPRa | CD8 | IFNG | 0,064051 | 0,889039 |
| ZNF689 | Primary | CRISPRa | CD8 | IFNG | 0,066093 | 0,889039 |
| MAX | Primary | CRISPRa | CD8 | IFNG | 0,08501 | 0,889039 |
| SRFBP1 | Primary | CRISPRa | CD8 | IFNG | 0,091116 | 0,889039 |
| ZNF142 | Primary | CRISPRa | CD8 | IFNG | 0,093115 | 0,889039 |
| ZNF71 | Primary | CRISPRa | CD8 | IFNG | 0,094266 | 0,889039 |
| GPBP1L1 | Primary | CRISPRa | CD8 | IFNG | 0,10665 | 0,889039 |
| ZNF793 | Primary | CRISPRa | CD8 | IFNG | 0,11207 | 0,889039 |
| TFDP3 | Primary | CRISPRa | CD8 | IFNG | 0,12551 | 0,889039 |
| UNKL | Primary | CRISPRa | CD8 | IFNG | 0,13477 | 0,889039 |
| PAXBP1 | Primary | CRISPRa | CD8 | IFNG | -0,089741 | 0,889858 |
| TAX1BP1 | Primary | CRISPRa | CD8 | IFNG | 0,013331 | 0,889858 |
| YY1AP1 | Primary | CRISPRa | CD8 | IFNG | 0,022962 | 0,889858 |
| CREBRF | Primary | CRISPRa | CD8 | IFNG | 0,068295 | 0,889858 |
| MAPK12 | Primary | CRISPRa | CD8 | IFNG | 0,078304 | 0,889858 |
| RING1 | Primary | CRISPRa | CD8 | IFNG | 0,13518 | 0,889858 |
| POU4F3 | Primary | CRISPRa | CD8 | IFNG | 0,0094915 | 0,889974 |
| ZNF575 | Primary | CRISPRa | CD8 | IFNG | 0,073789 | 0,889974 |
| HMGN3 | Primary | CRISPRa | CD8 | IFNG | 0,10688 | 0,889974 |
| CSDE1 | Primary | CRISPRa | CD8 | IFNG | 0,10942 | 0,889974 |
| PIDD1 | Primary | CRISPRa | CD8 | IFNG | 0,11583 | 0,889974 |
| PSMD12 | Primary | CRISPRa | CD8 | IFNG | -0,15045 | 0,890069 |
| ZNF548 | Primary | CRISPRa | CD8 | IFNG | -0,11219 | 0,890069 |
| PPP3CA | Primary | CRISPRa | CD8 | IFNG | -0,10345 | 0,890069 |
| ZNF215 | Primary | CRISPRa | CD8 | IFNG | -0,061597 | 0,890069 |
| ZNF253 | Primary | CRISPRa | CD8 | IFNG | -0,033799 | 0,890069 |
| DNTTIP2 | Primary | CRISPRa | CD8 | IFNG | 0,026312 | 0,890069 |
| CECR2 | Primary | CRISPRa | CD8 | IFNG | 0,087761 | 0,890069 |
| GTF3C1 | Primary | CRISPRa | CD8 | IFNG | 0,12617 | 0,890069 |
| CREBZF | Primary | CRISPRa | CD8 | IFNG | 0,13149 | 0,890069 |
| ZC3H4 | Primary | CRISPRa | CD8 | IFNG | 0,15082 | 0,890069 |
| DSCAM | Primary | CRISPRa | CD8 | IFNG | -0,18167 | 0,890158 |
| NCOA5 | Primary | CRISPRa | CD8 | IFNG | 0,068167 | 0,890338 |
| SAP30L | Primary | CRISPRa | CD8 | IFNG | -0,33839 | 0,892353 |
| ZC3H12A | Primary | CRISPRa | CD8 | IFNG | -0,0042226 | 0,892848 |
| PRKCI | Primary | CRISPRa | CD8 | IFNG | -0,25275 | 0,893072 |
| PHF10 | Primary | CRISPRa | CD8 | IFNG | -0,1257 | 0,893072 |
| CBLL1 | Primary | CRISPRa | CD8 | IFNG | -0,14697 | 0,895198 |
| HTATIP2 | Primary | CRISPRa | CD8 | IFNG | -0,097853 | 0,895303 |
| HES6 | Primary | CRISPRa | CD8 | IFNG | 0,045938 | 0,895303 |
| FGD1 | Primary | CRISPRa | CD8 | IFNG | 0,034447 | 0,895901 |
| ZNF101 | Primary | CRISPRa | CD8 | IFNG | -0,17547 | 0,897057 |
| SETD4 | Primary | CRISPRa | CD8 | IFNG | -0,024422 | 0,897057 |
| PHB | Primary | CRISPRa | CD8 | IFNG | -0,13978 | 0,897059 |
| GPATCH8 | Primary | CRISPRa | CD8 | IFNG | -0,088063 | 0,897059 |
| FHL2 | Primary | CRISPRa | CD8 | IFNG | -0,014972 | 0,897059 |
| CDY2A | Primary | CRISPRa | CD8 | IFNG | -0,058768 | 0,898449 |
| SOX9 | Primary | CRISPRa | CD8 | IFNG | -0,053354 | 0,89868 |
| EYA1 | Primary | CRISPRa | CD8 | IFNG | -0,17062 | 0,8996 |
| THOC5 | Primary | CRISPRa | CD8 | IFNG | -0,11559 | 0,8996 |
| ADGRA1 | Primary | CRISPRa | CD8 | IFNG | -0,025215 | 0,8996 |
| GTF3C2 | Primary | CRISPRa | CD8 | IFNG | 0,053393 | 0,8996 |
| BAG1 | Primary | CRISPRa | CD8 | IFNG | 0,069529 | 0,8996 |
| MIER1 | Primary | CRISPRa | CD8 | IFNG | 0,12015 | 0,8996 |
| ZNF263 | Primary | CRISPRa | CD8 | IFNG | -0,057365 | 0,899762 |
| TCEAL4 | Primary | CRISPRa | CD8 | IFNG | 0,10569 | 0,900015 |
| ZNF438 | Primary | CRISPRa | CD8 | IFNG | 0,20521 | 0,900015 |
| ZNF25 | Primary | CRISPRa | CD8 | IFNG | 0,10929 | 0,900097 |
| TAX1BP3 | Primary | CRISPRa | CD8 | IFNG | 0,16495 | 0,900097 |
| ZNF226 | Primary | CRISPRa | CD8 | IFNG | 0,19326 | 0,900097 |
| ACTL6B | Primary | CRISPRa | CD8 | IFNG | -0,13776 | 0,904664 |
| ZNF83 | Primary | CRISPRa | CD8 | IFNG | 0,14527 | 0,905956 |
| EBF4 | Primary | CRISPRa | CD8 | IFNG | -0,081716 | 0,908383 |
| MED27 | Primary | CRISPRa | CD8 | IFNG | 0,023269 | 0,908383 |
| ZNF565 | Primary | CRISPRa | CD8 | IFNG | -0,16752 | 0,909151 |
| ZNF2 | Primary | CRISPRa | CD8 | IFNG | -0,052782 | 0,909151 |
| GRHPR | Primary | CRISPRa | CD8 | IFNG | 0,059743 | 0,909151 |
| VGLL2 | Primary | CRISPRa | CD8 | IFNG | 0,017098 | 0,91155 |
| CENPT | Primary | CRISPRa | CD8 | IFNG | 0,13573 | 0,912374 |
| GTF2A1 | Primary | CRISPRa | CD8 | IFNG | 0,23604 | 0,913768 |
| HSPA1A | Primary | CRISPRa | CD8 | IFNG | -0,073421 | 0,918412 |
| PAXIP1 | Primary | CRISPRa | CD8 | IFNG | -0,030416 | 0,919859 |
| ZNF200 | Primary | CRISPRa | CD8 | IFNG | -0,23403 | 0,920044 |
| DUX4 | Primary | CRISPRa | CD8 | IFNG | -0,20786 | 0,920044 |
| HSPH1 | Primary | CRISPRa | CD8 | IFNG | 0,09601 | 0,920044 |
| SNAPC2 | Primary | CRISPRa | CD8 | IFNG | 0,19107 | 0,920044 |
| EFCAB6 | Primary | CRISPRa | CD8 | IFNG | -0,012929 | 0,920675 |
| PDX1 | Primary | CRISPRa | CD8 | IFNG | 0,13675 | 0,921092 |
| OTP | Primary | CRISPRa | CD8 | IFNG | -0,043237 | 0,921357 |
| ZNF407 | Primary | CRISPRa | CD8 | IFNG | -0,11144 | 0,922557 |
| PRB4 | Primary | CRISPRa | CD8 | IFNG | -0,076402 | 0,922613 |
| ZBTB18 | Primary | CRISPRa | CD8 | IFNG | -0,15263 | 0,922931 |
| NFATC4 | Primary | CRISPRa | CD8 | IFNG | 0,00037537 | 0,922931 |
| SMARCD2 | Primary | CRISPRa | CD8 | IFNG | 0,030416 | 0,922931 |
| CGGBP1 | Primary | CRISPRa | CD8 | IFNG | 0,15352 | 0,922931 |
| NUP35 | Primary | CRISPRa | CD8 | IFNG | 0,24291 | 0,922931 |
| HIF1A | Primary | CRISPRa | CD8 | IFNG | -0,17739 | 0,923343 |
| KDM1B | Primary | CRISPRa | CD8 | IFNG | -0,063367 | 0,923343 |
| SIRT2 | Primary | CRISPRa | CD8 | IFNG | -0,17535 | 0,924586 |
| HIC2 | Primary | CRISPRa | CD8 | IFNG | 0,037804 | 0,924586 |
| USP2 | Primary | CRISPRa | CD8 | IFNG | -0,080182 | 0,924605 |
| GTF2IRD2 | Primary | CRISPRa | CD8 | IFNG | -0,11054 | 0,92465 |
| ZNF185 | Primary | CRISPRa | CD8 | IFNG | -0,038242 | 0,925818 |
| ISX | Primary | CRISPRa | CD8 | IFNG | 0,05911 | 0,925818 |
| RPAP2 | Primary | CRISPRa | CD8 | IFNG | -0,019663 | 0,927456 |
| RBM22 | Primary | CRISPRa | CD8 | IFNG | 0,052959 | 0,927456 |
| COPS3 | Primary | CRISPRa | CD8 | IFNG | 0,18551 | 0,927456 |
| LZTS1 | Primary | CRISPRa | CD8 | IFNG | -0,24775 | 0,927459 |
| KDM6A | Primary | CRISPRa | CD8 | IFNG | -0,16641 | 0,927459 |
| TERF1 | Primary | CRISPRa | CD8 | IFNG | -0,10998 | 0,927459 |
| SOX5 | Primary | CRISPRa | CD8 | IFNG | -0,082967 | 0,927459 |
| SFR1 | Primary | CRISPRa | CD8 | IFNG | -0,073861 | 0,927459 |
| MGMT | Primary | CRISPRa | CD8 | IFNG | -0,040663 | 0,927459 |
| FLII | Primary | CRISPRa | CD8 | IFNG | -0,010803 | 0,927459 |
| HOXD10 | Primary | CRISPRa | CD8 | IFNG | 0,0032752 | 0,927459 |
| BPTF | Primary | CRISPRa | CD8 | IFNG | 0,077851 | 0,927459 |
| SP100 | Primary | CRISPRa | CD8 | IFNG | 0,010201 | 0,927793 |
| FOXS1 | Primary | CRISPRa | CD8 | IFNG | -0,15148 | 0,928449 |
| HELT | Primary | CRISPRa | CD8 | IFNG | -0,050161 | 0,929484 |
| RHOXF2B | Primary | CRISPRa | CD8 | IFNG | -0,0094543 | 0,929484 |
| SNX6 | Primary | CRISPRa | CD8 | IFNG | -0,26275 | 0,93144 |
| RWDD3 | Primary | CRISPRa | CD8 | IFNG | -0,18959 | 0,93144 |
| CLU | Primary | CRISPRa | CD8 | IFNG | -0,10406 | 0,93144 |
| TNP1 | Primary | CRISPRa | CD8 | IFNG | -0,088776 | 0,93144 |
| PPP1R12A | Primary | CRISPRa | CD8 | IFNG | -0,071711 | 0,93144 |
| ST18 | Primary | CRISPRa | CD8 | IFNG | -0,040308 | 0,93144 |
| MED26 | Primary | CRISPRa | CD8 | IFNG | -0,024812 | 0,93144 |
| ZNF284 | Primary | CRISPRa | CD8 | IFNG | -0,00031523 | 0,93144 |
| ELF4 | Primary | CRISPRa | CD8 | IFNG | 0,0015196 | 0,93144 |
| ZNF658 | Primary | CRISPRa | CD8 | IFNG | 0,06728 | 0,93144 |
| ZFP1 | Primary | CRISPRa | CD8 | IFNG | 0,08285 | 0,93144 |
| USF1 | Primary | CRISPRa | CD8 | IFNG | 0,093145 | 0,931886 |
| COPS4 | Primary | CRISPRa | CD8 | IFNG | 0,087747 | 0,932663 |
| BRD3 | Primary | CRISPRa | CD8 | IFNG | 0,17383 | 0,932849 |
| SSB | Primary | CRISPRa | CD8 | IFNG | -0,0037917 | 0,933615 |
| ZNF92 | Primary | CRISPRa | CD8 | IFNG | -0,12438 | 0,933646 |
| NCOA6 | Primary | CRISPRa | CD8 | IFNG | -0,10117 | 0,933646 |
| ERN1 | Primary | CRISPRa | CD8 | IFNG | -0,056409 | 0,933646 |
| HDAC9 | Primary | CRISPRa | CD8 | IFNG | -0,05263 | 0,933646 |
| RAG1 | Primary | CRISPRa | CD8 | IFNG | -0,034386 | 0,933646 |
| TCF25 | Primary | CRISPRa | CD8 | IFNG | -0,032856 | 0,933646 |
| ZNF687 | Primary | CRISPRa | CD8 | IFNG | 0,015212 | 0,933646 |
| ZSCAN20 | Primary | CRISPRa | CD8 | IFNG | 0,016901 | 0,933646 |
| TAF1A | Primary | CRISPRa | CD8 | IFNG | 0,020205 | 0,933646 |
| MAGED1 | Primary | CRISPRa | CD8 | IFNG | 0,022102 | 0,933646 |
| KLHL41 | Primary | CRISPRa | CD8 | IFNG | 0,022644 | 0,933646 |
| HOPX | Primary | CRISPRa | CD8 | IFNG | 0,023002 | 0,933646 |
| TFB2M | Primary | CRISPRa | CD8 | IFNG | 0,049009 | 0,933646 |
| ZHX1 | Primary | CRISPRa | CD8 | IFNG | 0,067242 | 0,933646 |
| ZNF264 | Primary | CRISPRa | CD8 | IFNG | 0,069767 | 0,933646 |
| ZFP36L1 | Primary | CRISPRa | CD8 | IFNG | 0,075316 | 0,933646 |
| HEXIM2 | Primary | CRISPRa | CD8 | IFNG | 0,081121 | 0,933646 |
| ZNF367 | Primary | CRISPRa | CD8 | IFNG | 0,11036 | 0,933646 |
| HIVEP3 | Primary | CRISPRa | CD8 | IFNG | 0,14211 | 0,933646 |
| HSF1 | Primary | CRISPRa | CD8 | IFNG | 0,23539 | 0,933646 |
| TRIM47 | Primary | CRISPRa | CD8 | IFNG | -0,04859 | 0,933785 |
| ZNF286A | Primary | CRISPRa | CD8 | IFNG | 0,13115 | 0,934082 |
| RFX1 | Primary | CRISPRa | CD8 | IFNG | -0,13114 | 0,934294 |
| MSC | Primary | CRISPRa | CD8 | IFNG | -0,24192 | 0,934945 |
| GTF2B | Primary | CRISPRa | CD8 | IFNG | -0,088917 | 0,934945 |
| CDC6 | Primary | CRISPRa | CD8 | IFNG | 0,17752 | 0,93499 |
| NEUROD1 | Primary | CRISPRa | CD8 | IFNG | -0,12595 | 0,935562 |
| PRDX5 | Primary | CRISPRa | CD8 | IFNG | -0,048014 | 0,935562 |
| PSMC6 | Primary | CRISPRa | CD8 | IFNG | -0,027808 | 0,935562 |
| SREBF2 | Primary | CRISPRa | CD8 | IFNG | 0,0037928 | 0,935562 |
| ZNF568 | Primary | CRISPRa | CD8 | IFNG | 0,026852 | 0,935562 |
| POLR3G | Primary | CRISPRa | CD8 | IFNG | 0,080382 | 0,935562 |
| KIAA0040 | Primary | CRISPRa | CD8 | IFNG | -0,040958 | 0,93658 |
| MKX | Primary | CRISPRa | CD8 | IFNG | -0,034417 | 0,937071 |
| WWOX | Primary | CRISPRa | CD8 | IFNG | 0,044598 | 0,937098 |
| BTBD6 | Primary | CRISPRa | CD8 | IFNG | 0,13382 | 0,937427 |
| ZNF433 | Primary | CRISPRa | CD8 | IFNG | -0,15404 | 0,937999 |
| SKI | Primary | CRISPRa | CD8 | IFNG | -0,11811 | 0,937999 |
| NAB2 | Primary | CRISPRa | CD8 | IFNG | -0,24217 | 0,938371 |
| ZNF512 | Primary | CRISPRa | CD8 | IFNG | -0,23064 | 0,938371 |
| RBPJL | Primary | CRISPRa | CD8 | IFNG | -0,20751 | 0,938371 |
| HOXD3 | Primary | CRISPRa | CD8 | IFNG | -0,12336 | 0,938371 |
| ZNF654 | Primary | CRISPRa | CD8 | IFNG | -0,068842 | 0,938371 |
| MED20 | Primary | CRISPRa | CD8 | IFNG | -0,059641 | 0,938371 |
| ATOH8 | Primary | CRISPRa | CD8 | IFNG | 0,053861 | 0,938371 |
| ZNF764 | Primary | CRISPRa | CD8 | IFNG | 0,073159 | 0,938371 |
| PSEN1 | Primary | CRISPRa | CD8 | IFNG | 0,095477 | 0,938371 |
| PAK6 | Primary | CRISPRa | CD8 | IFNG | 0,12941 | 0,938371 |
| GMEB2 | Primary | CRISPRa | CD8 | IFNG | 0,053461 | 0,939897 |
| RYBP | Primary | CRISPRa | CD8 | IFNG | 0,096232 | 0,939897 |
| TRIM37 | Primary | CRISPRa | CD8 | IFNG | -0,13809 | 0,940077 |
| ZNF579 | Primary | CRISPRa | CD8 | IFNG | 0,064584 | 0,940077 |
| SETD6 | Primary | CRISPRa | CD8 | IFNG | 0,14026 | 0,940077 |
| COPRS | Primary | CRISPRa | CD8 | IFNG | -0,088486 | 0,940177 |
| KDM6B | Primary | CRISPRa | CD8 | IFNG | 0,0042656 | 0,940177 |
| TRMT1L | Primary | CRISPRa | CD8 | IFNG | 0,021722 | 0,941738 |
| ATOH7 | Primary | CRISPRa | CD8 | IFNG | -0,10213 | 0,942906 |
| GSX2 | Primary | CRISPRa | CD8 | IFNG | 0,10057 | 0,943606 |
| CENPU | Primary | CRISPRa | CD8 | IFNG | -0,086537 | 0,943736 |
| MBD5 | Primary | CRISPRa | CD8 | IFNG | -0,043351 | 0,94705 |
| TBX6 | Primary | CRISPRa | CD8 | IFNG | 0,23147 | 0,94705 |
| IRF9 | Primary | CRISPRa | CD8 | IFNG | -0,23607 | 0,947298 |
| UBA3 | Primary | CRISPRa | CD8 | IFNG | -0,23256 | 0,947298 |
| DUXA | Primary | CRISPRa | CD8 | IFNG | -0,21617 | 0,947298 |
| TAF8 | Primary | CRISPRa | CD8 | IFNG | -0,15558 | 0,947298 |
| ARID3B | Primary | CRISPRa | CD8 | IFNG | -0,14311 | 0,947298 |
| ARHGAP35 | Primary | CRISPRa | CD8 | IFNG | -0,10708 | 0,947298 |
| ZNF491 | Primary | CRISPRa | CD8 | IFNG | -0,06013 | 0,947298 |
| DPF3 | Primary | CRISPRa | CD8 | IFNG | -0,040936 | 0,947298 |
| ZNF641 | Primary | CRISPRa | CD8 | IFNG | -0,03991 | 0,947298 |
| ZFP90 | Primary | CRISPRa | CD8 | IFNG | -0,02828 | 0,947298 |
| PKHD1L1 | Primary | CRISPRa | CD8 | IFNG | -0,021198 | 0,947298 |
| HOXD9 | Primary | CRISPRa | CD8 | IFNG | -0,01738 | 0,947298 |
| ZNF735 | Primary | CRISPRa | CD8 | IFNG | -0,0053265 | 0,947298 |
| IRF6 | Primary | CRISPRa | CD8 | IFNG | -0,0030849 | 0,947298 |
| DAPK3 | Primary | CRISPRa | CD8 | IFNG | 0,0060473 | 0,947298 |
| WASL | Primary | CRISPRa | CD8 | IFNG | 0,054137 | 0,947298 |
| PPP3R1 | Primary | CRISPRa | CD8 | IFNG | 0,1375 | 0,947298 |
| THYN1 | Primary | CRISPRa | CD8 | IFNG | -0,06378 | 0,94771 |
| ZNF251 | Primary | CRISPRa | CD8 | IFNG | 0,03973 | 0,94771 |
| SGK1 | Primary | CRISPRa | CD8 | IFNG | -0,12914 | 0,948033 |
| CHRAC1 | Primary | CRISPRa | CD8 | IFNG | 0,12151 | 0,948033 |
| ZNF280B | Primary | CRISPRa | CD8 | IFNG | -0,18568 | 0,94863 |
| PRDM5 | Primary | CRISPRa | CD8 | IFNG | -0,12753 | 0,949252 |
| RTF1 | Primary | CRISPRa | CD8 | IFNG | -0,10737 | 0,949252 |
| AKT2 | Primary | CRISPRa | CD8 | IFNG | -0,047887 | 0,949252 |
| ZNF48 | Primary | CRISPRa | CD8 | IFNG | -0,045085 | 0,949252 |
| PAX8 | Primary | CRISPRa | CD8 | IFNG | 0,015945 | 0,949252 |
| SMARCAD1 | Primary | CRISPRa | CD8 | IFNG | 0,020043 | 0,949252 |
| SMURF1 | Primary | CRISPRa | CD8 | IFNG | 0,083492 | 0,949252 |
| MMS19 | Primary | CRISPRa | CD8 | IFNG | 0,17533 | 0,949252 |
| TRIM66 | Primary | CRISPRa | CD8 | IFNG | -0,25819 | 0,949298 |
| PA2G4 | Primary | CRISPRa | CD8 | IFNG | -0,00068941 | 0,951195 |
| ZNF354A | Primary | CRISPRa | CD8 | IFNG | -0,21417 | 0,952016 |
| COMMD1 | Primary | CRISPRa | CD8 | IFNG | -0,16898 | 0,952016 |
| TIGD5 | Primary | CRISPRa | CD8 | IFNG | -0,14193 | 0,952016 |
| DMC1 | Primary | CRISPRa | CD8 | IFNG | -0,093286 | 0,952016 |
| RXRB | Primary | CRISPRa | CD8 | IFNG | -0,082038 | 0,952016 |
| ETV3L | Primary | CRISPRa | CD8 | IFNG | -0,080282 | 0,952016 |
| NCOA7 | Primary | CRISPRa | CD8 | IFNG | -0,076615 | 0,952016 |
| TTF2 | Primary | CRISPRa | CD8 | IFNG | -0,073846 | 0,952016 |
| USP21 | Primary | CRISPRa | CD8 | IFNG | -0,053118 | 0,952016 |
| TBX18 | Primary | CRISPRa | CD8 | IFNG | -0,026981 | 0,952016 |
| ZNF528 | Primary | CRISPRa | CD8 | IFNG | -0,026701 | 0,952016 |
| ZNF213 | Primary | CRISPRa | CD8 | IFNG | -0,02458 | 0,952016 |
| TARBP1 | Primary | CRISPRa | CD8 | IFNG | 0,030803 | 0,952016 |
| AKIRIN1 | Primary | CRISPRa | CD8 | IFNG | 0,032922 | 0,952016 |
| SUV39H2 | Primary | CRISPRa | CD8 | IFNG | 0,04372 | 0,952016 |
| GTF2IRD2B | Primary | CRISPRa | CD8 | IFNG | 0,064607 | 0,952016 |
| ANP32B | Primary | CRISPRa | CD8 | IFNG | 0,065416 | 0,952016 |
| RCOR1 | Primary | CRISPRa | CD8 | IFNG | 0,071427 | 0,952016 |
| CCDC59 | Primary | CRISPRa | CD8 | IFNG | 0,075213 | 0,952016 |
| MYOD1 | Primary | CRISPRa | CD8 | IFNG | 0,077391 | 0,952016 |
| RB1CC1 | Primary | CRISPRa | CD8 | IFNG | 0,099335 | 0,952016 |
| E2F8 | Primary | CRISPRa | CD8 | IFNG | 0,11887 | 0,952016 |
| HES4 | Primary | CRISPRa | CD8 | IFNG | 0,12276 | 0,952016 |
| NOC2L | Primary | CRISPRa | CD8 | IFNG | 0,15545 | 0,952016 |
| HDAC10 | Primary | CRISPRa | CD8 | IFNG | 0,042132 | 0,95255 |
| VEZF1 | Primary | CRISPRa | CD8 | IFNG | 0,11075 | 0,95255 |
| ZFP57 | Primary | CRISPRa | CD8 | IFNG | -0,16623 | 0,952728 |
| TRIM31 | Primary | CRISPRa | CD8 | IFNG | -0,15854 | 0,952728 |
| ZBTB47 | Primary | CRISPRa | CD8 | IFNG | -0,0090459 | 0,952728 |
| FBXW7 | Primary | CRISPRa | CD8 | IFNG | 0,038167 | 0,952903 |
| ZNF813 | Primary | CRISPRa | CD8 | IFNG | 0,17213 | 0,954945 |
| SIN3B | Primary | CRISPRa | CD8 | IFNG | 0,0039708 | 0,956416 |
| FERD3L | Primary | CRISPRa | CD8 | IFNG | 0,16557 | 0,956416 |
| ZNF132 | Primary | CRISPRa | CD8 | IFNG | 0,079315 | 0,958621 |
| BRD8 | Primary | CRISPRa | CD8 | IFNG | 0,091468 | 0,960208 |
| ASCC3 | Primary | CRISPRa | CD8 | IFNG | 0,058172 | 0,961258 |
| ZNF749 | Primary | CRISPRa | CD8 | IFNG | 0,10496 | 0,961258 |
| MED13L | Primary | CRISPRa | CD8 | IFNG | 0,036652 | 0,962083 |
| TRIM29 | Primary | CRISPRa | CD8 | IFNG | -0,11195 | 0,962278 |
| NR1D1 | Primary | CRISPRa | CD8 | IFNG | 0,048439 | 0,962278 |
| ZNF584 | Primary | CRISPRa | CD8 | IFNG | 0,070858 | 0,962278 |
| SSU72 | Primary | CRISPRa | CD8 | IFNG | 0,087845 | 0,962278 |
| POLR2A | Primary | CRISPRa | CD8 | IFNG | -0,0031574 | 0,962302 |
| PKHD1 | Primary | CRISPRa | CD8 | IFNG | 0,049131 | 0,963203 |
| MRGBP | Primary | CRISPRa | CD8 | IFNG | 0,20366 | 0,963656 |
| ZNF805 | Primary | CRISPRa | CD8 | IFNG | -0,14001 | 0,963853 |
| CHMP1A | Primary | CRISPRa | CD8 | IFNG | 0,0076468 | 0,963853 |
| ZNF281 | Primary | CRISPRa | CD8 | IFNG | 0,011386 | 0,963853 |
| ABRA | Primary | CRISPRa | CD8 | IFNG | 0,036158 | 0,963853 |
| NR1H3 | Primary | CRISPRa | CD8 | IFNG | 0,070984 | 0,963853 |
| ETV6 | Primary | CRISPRa | CD8 | IFNG | 0,089642 | 0,963853 |
| ZFP36L2 | Primary | CRISPRa | CD8 | IFNG | -0,086676 | 0,964578 |
| GMNN | Primary | CRISPRa | CD8 | IFNG | -0,017227 | 0,965067 |
| TONSL | Primary | CRISPRa | CD8 | IFNG | 0,012327 | 0,965067 |
| HCK | Primary | CRISPRa | CD8 | IFNG | 0,062612 | 0,965067 |
| IRF1 | Primary | CRISPRa | CD8 | IFNG | -0,15422 | 0,965564 |
| SFSWAP | Primary | CRISPRa | CD8 | IFNG | -0,073926 | 0,965564 |
| ZNF75D | Primary | CRISPRa | CD8 | IFNG | -0,029453 | 0,965564 |
| TNIP2 | Primary | CRISPRa | CD8 | IFNG | 0,0063522 | 0,965564 |
| CSNK2A2 | Primary | CRISPRa | CD8 | IFNG | 0,0068378 | 0,965564 |
| CRIP1 | Primary | CRISPRa | CD8 | IFNG | 0,022851 | 0,965564 |
| RGS7 | Primary | CRISPRa | CD8 | IFNG | 0,12273 | 0,965564 |
| ZNF556 | Primary | CRISPRa | CD8 | IFNG | 0,1686 | 0,965564 |
| ZNF880 | Primary | CRISPRa | CD8 | IFNG | -0,043786 | 0,965609 |
| DYRK1B | Primary | CRISPRa | CD8 | IFNG | 0,058312 | 0,967781 |
| PSIP1 | Primary | CRISPRa | CD8 | IFNG | -0,31134 | 0,968345 |
| RNF138 | Primary | CRISPRa | CD8 | IFNG | -0,16093 | 0,968345 |
| TSHZ3 | Primary | CRISPRa | CD8 | IFNG | -0,05525 | 0,968345 |
| CTNNBIP1 | Primary | CRISPRa | CD8 | IFNG | 0,067868 | 0,968345 |
| ZNF280C | Primary | CRISPRa | CD8 | IFNG | 0,14068 | 0,968345 |
| SAMD11 | Primary | CRISPRa | CD8 | IFNG | -0,22199 | 0,969144 |
| AKNA | Primary | CRISPRa | CD8 | IFNG | 0,00098539 | 0,970522 |
| TTLL4 | Primary | CRISPRa | CD8 | IFNG | 0,016697 | 0,971502 |
| ZNF469 | Primary | CRISPRa | CD8 | IFNG | -0,077911 | 0,971723 |
| STRN3 | Primary | CRISPRa | CD8 | IFNG | 0,19516 | 0,972119 |
| ZNF846 | Primary | CRISPRa | CD8 | IFNG | -0,080878 | 0,972608 |
| CHAF1B | Primary | CRISPRa | CD8 | IFNG | -0,044943 | 0,972608 |
| POU6F1 | Primary | CRISPRa | CD8 | IFNG | 0,030288 | 0,972608 |
| CNOT6L | Primary | CRISPRa | CD8 | IFNG | 0,10487 | 0,973718 |
| SDR16C5 | Primary | CRISPRa | CD8 | IFNG | 0,069975 | 0,975009 |
| VGLL1 | Primary | CRISPRa | CD8 | IFNG | 0,20472 | 0,975009 |
| FOXI1 | Primary | CRISPRa | CD8 | IFNG | 0,11821 | 0,975513 |
| ZSCAN18 | Primary | CRISPRa | CD8 | IFNG | -0,098035 | 0,979409 |
| SCML4 | Primary | CRISPRa | CD8 | IFNG | 0,062039 | 0,979409 |
| MED15 | Primary | CRISPRa | CD8 | IFNG | -0,25193 | 0,979465 |
| BRD1 | Primary | CRISPRa | CD8 | IFNG | -0,17041 | 0,979465 |
| ZNF331 | Primary | CRISPRa | CD8 | IFNG | -0,18401 | 0,982321 |
| ZNF365 | Primary | CRISPRa | CD8 | IFNG | -0,19232 | 0,985405 |
| GPS2 | Primary | CRISPRa | CD8 | IFNG | 0,040365 | 0,985405 |
| EHF | Primary | CRISPRa | CD8 | IFNG | 0,12497 | 0,985405 |
| PAX4 | Primary | CRISPRa | CD8 | IFNG | -0,13112 | 0,985686 |
| ZBTB34 | Primary | CRISPRa | CD8 | IFNG | -0,041639 | 0,985686 |
| ASCL1 | Primary | CRISPRa | CD8 | IFNG | -0,0072419 | 0,985686 |
| ZNF688 | Primary | CRISPRa | CD8 | IFNG | 0,059834 | 0,985686 |
| ZNF112 | Primary | CRISPRa | CD8 | IFNG | 0,15869 | 0,985686 |
| IKZF5 | Primary | CRISPRa | CD8 | IFNG | -0,045218 | 0,986083 |
| ZNF582 | Primary | CRISPRa | CD8 | IFNG | -0,052691 | 0,987354 |
| RAD51 | Primary | CRISPRa | CD8 | IFNG | -0,0019735 | 0,987354 |
| SNAPC3 | Primary | CRISPRa | CD8 | IFNG | 0,055744 | 0,98754 |
| RIOK2 | Primary | CRISPRa | CD8 | IFNG | -0,10967 | 0,988075 |
| PBX4 | Primary | CRISPRa | CD8 | IFNG | -0,031033 | 0,988075 |
| PREB | Primary | CRISPRa | CD8 | IFNG | 0,055048 | 0,988075 |
| KRTAP5-9 | Primary | CRISPRa | CD8 | IFNG | -0,22309 | 0,990725 |
| ARID1B | Primary | CRISPRa | CD8 | IFNG | 0,0061064 | 0,992548 |
| SFRP4 | Primary | CRISPRa | CD8 | IFNG | -0,1555 | 0,992751 |
| ZNF488 | Primary | CRISPRa | CD8 | IFNG | -0,12222 | 0,992751 |
| THAP8 | Primary | CRISPRa | CD8 | IFNG | -0,10896 | 0,992751 |
| MEOX1 | Primary | CRISPRa | CD8 | IFNG | -0,1054 | 0,992751 |
| TP73 | Primary | CRISPRa | CD8 | IFNG | -0,29861 | 0,99325 |
| MNDA | Primary | CRISPRa | CD8 | IFNG | -0,066236 | 0,99325 |
| ZNF716 | Primary | CRISPRa | CD8 | IFNG | -0,11179 | 0,994657 |
| RRP1B | Primary | CRISPRa | CD8 | IFNG | -0,19797 | 0,994827 |
| SCAI | Primary | CRISPRa | CD8 | IFNG | -0,15912 | 0,994827 |
| RPS6KA5 | Primary | CRISPRa | CD8 | IFNG | -0,1332 | 0,994827 |
| ZNF571 | Primary | CRISPRa | CD8 | IFNG | -0,12487 | 0,994827 |
| ZNF454 | Primary | CRISPRa | CD8 | IFNG | -0,10011 | 0,994827 |
| ABT1 | Primary | CRISPRa | CD8 | IFNG | -0,089619 | 0,994827 |
| SOX30 | Primary | CRISPRa | CD8 | IFNG | -0,086409 | 0,994827 |
| NR1D2 | Primary | CRISPRa | CD8 | IFNG | -0,022968 | 0,994827 |
| NOD2 | Primary | CRISPRa | CD8 | IFNG | 0,037495 | 0,994827 |
| ZNF737 | Primary | CRISPRa | CD8 | IFNG | 0,064506 | 0,994827 |
| GLYR1 | Primary | CRISPRa | CD8 | IFNG | 0,076784 | 0,994827 |
| ZNF10 | Primary | CRISPRa | CD8 | IFNG | 0,090784 | 0,994827 |
| PLAG1 | Primary | CRISPRa | CD8 | IFNG | 0,10839 | 0,994827 |
| GFI1B | Primary | CRISPRa | CD8 | IFNG | 0,10914 | 0,994827 |
| MDM4 | Primary | CRISPRa | CD8 | IFNG | 0,11437 | 0,994827 |
| ZNF510 | Primary | CRISPRa | CD8 | IFNG | 0,24253 | 0,994827 |
| ZBTB4 | Primary | CRISPRa | CD8 | IFNG | 0,065098 | 0,99602 |
| ZFP36 | Primary | CRISPRa | CD8 | IFNG | -0,14376 | 0,999153 |
| TADA1 | Primary | CRISPRa | CD8 | IFNG | -0,085292 | 0,999153 |
| RITA1 | Primary | CRISPRa | CD8 | IFNG | 0,15575 | 0,999153 |
| RARA | Primary | CRISPRa | CD8 | IFNG | 0,011203 | 0,999313 |
| SP2 | Primary | CRISPRa | CD8 | IFNG | -0,1147 | 1 |
| RGS11 | Primary | CRISPRa | CD8 | IFNG | -0,071247 | 1 |
| PAWR | Primary | CRISPRa | CD8 | IFNG | -0,057548 | 1 |
| ZNF549 | Primary | CRISPRa | CD8 | IFNG | -0,0369 | 1 |
| STRAP | Primary | CRISPRa | CD8 | IFNG | 0,003259 | 1 |
| DLX2 | Primary | CRISPRa | CD8 | IFNG | 0,021521 | 1 |
| TDRD3 | Primary | CRISPRa | CD8 | IFNG | 0,10282 | 1 |
| PHF19 | Primary | CRISPRa | CD8 | IFNG | 0,10862 | 1 |
| ZBED5 | Primary | CRISPRa | CD8 | IFNG | 0,12953 | 1 |

**Table. S2.**

**Supplementary Table 2.** Expression levels (assessed by RNA-seq) of the top negative hits identified in the CRISPRa screen in CD8^+^ T cells cultured for 4 d under different conditions: No Cytokine (NC), IL-2, IL-2 + LDHi, IL-21, IL-21 + LDHi^23^

|  | **Symbol** | **NC.1** | **IL2.1** | **IL21.1** | **IL2.LDHi.1** | **IL21.LDHi.1** | **NC.2** | **IL2.2** | **IL21.2** | **IL2.LDHi.2** | **IL21.LDHi.2** |
| --- | --- | --- | --- | --- | --- | --- | --- | --- | --- | --- | --- |
| Jmjd1c | | 29,18 | 24,85 | 26,76 | 25,44 | 23,01 | 30,84 | 26,28 | 31,25 | 23,89 | 23,99 |
| Ikzf3 | | 141,54 | 38,89 | 98,92 | 72,10 | 47,18 | 122,59 | 41,31 | 117,32 | 70,59 | 53,98 |
| Foxf1 | | 0,02 | 0,04 | 0,00 | 0,00 | 0,01 | 0,00 | 0,05 | 0,06 | 0,00 | 0,01 |
| Foxl2 | | 0,00 | 0,00 | 0,00 | 0,00 | 0,01 | 0,00 | 0,00 | 0,00 | 0,00 | 0,02 |
| Gata3 | | 11,66 | 17,12 | 6,57 | 12,08 | 3,64 | 9,58 | 14,66 | 7,69 | 11,85 | 3,33 |
| Ebf2 | | 0,01 | 0,00 | 0,00 | 0,00 | 0,01 | 0,01 | 0,00 | 0,02 | 0,00 | 0,00 |
| Gata6 | | 0,02 | 0,00 | 0,00 | 0,03 | 0,00 | 0,00 | 0,00 | 0,00 | 0,01 | 0,00 |
| Cebpb | | 4,65 | 4,84 | 3,66 | 2,14 | 1,34 | 5,04 | 6,32 | 3,36 | 2,63 | 2,32 |
| Foxa3 | | 0,04 | 0,01 | 0,00 | 0,01 | 0,01 | 0,00 | 0,03 | 0,11 | 0,03 | 0,00 |
| Trim28 | | 70,52 | 97,25 | 62,17 | 85,44 | 67,63 | 62,03 | 108,48 | 46,59 | 92,58 | 72,43 |
| Ikzf1 | | 141,70 | 127,46 | 171,59 | 160,13 | 186,52 | 161,05 | 124,53 | 188,37 | 163,31 | 182,43 |
| Pou2af1 | | 6,54 | 2,11 | 14,40 | 4,89 | 10,65 | 7,20 | 2,15 | 18,03 | 5,63 | 8,15 |
| Foxf2 | | 0,00 | 0,01 | 0,00 | 0,01 | 0,01 | 0,00 | 0,00 | 0,03 | 0,00 | 0,00 |
| Lmo4 | | 14,31 | 13,51 | 10,59 | 14,13 | 48,98 | 20,04 | 13,51 | 22,25 | 15,38 | 42,92 |

Table. S3.

**Supplementary Table 3.** Changes in gene expression between pmel-1 Thy1.1^+^ and Lmo4-Thy1.1^+^ CD8^+^ T cells. Gene expression was evaluated by RNA-seq of pmel-1 CD62L^-^KLRG1^-^ T cells collected 5 d after transfer of 10^5^ pmel-1 Thy1.1^+^ and Lmo4-Thy1.1^+^ CD8^+^ T cells into wild-type mice

| **Gene** | **WT1** | **WT2** | **WT3** | **WT4** | **Lmo4OE2** | **Lmo4OE3** | **Lmo4OE4** | **Lmo4OE5** | **logFC** | **logCPM** | **PValue** | **FDR** |
| --- | --- | --- | --- | --- | --- | --- | --- | --- | --- | --- | --- | --- |
| Lmo4 | 10,14 | 11,53 | 11,16 | 10,01 | 483,55 | 504,63 | 503,71 | 476,99 | 5,53 | 8,75 | 0 | 0 |
| Lars2 | 19,04 | 143,42 | 45,47 | 82,27 | 123,54 | 157,63 | 124,97 | 90,24 | 0,77 | 8,58 | 9,18E-30 | 4,31E-26 |
| Slc40a1 | 12,51 | 6,67 | 5,60 | 7,54 | 5,45 | 7,23 | 1,99 | 1,07 | -1,05 | 4,34 | 2,83E-25 | 8,86E-22 |
| Vcam1 | 14,18 | 8,31 | 6,71 | 7,20 | 6,41 | 8,67 | 2,45 | 1,22 | -0,97 | 4,55 | 1,33E-23 | 3,14E-20 |
| Csf1r | 8,62 | 5,94 | 4,69 | 4,93 | 4,42 | 5,51 | 1,82 | 0,79 | -0,96 | 4,15 | 1,08E-19 | 2,04E-16 |
| Hsph1 | 42,93 | 41,67 | 48,37 | 37,28 | 67,57 | 64,08 | 53,43 | 71,65 | 0,60 | 7,54 | 1,66E-19 | 2,60E-16 |
| Aspm | 5,00 | 6,10 | 5,25 | 5,17 | 2,76 | 2,52 | 4,15 | 3,76 | -0,71 | 5,42 | 2,28E-19 | 3,06E-16 |
| Hmox1 | 23,07 | 20,90 | 17,52 | 16,24 | 13,35 | 15,70 | 8,01 | 5,90 | -0,86 | 4,57 | 3,09E-19 | 3,64E-16 |
| Mrc1 | 4,41 | 2,75 | 2,54 | 2,31 | 2,09 | 2,69 | 0,69 | 0,45 | -1,03 | 3,57 | 2,19E-17 | 2,08E-14 |
| Hist1h3b | 208,42 | 290,82 | 271,05 | 204,60 | 148,86 | 165,85 | 199,72 | 145,29 | -0,56 | 6,39 | 2,21E-17 | 2,08E-14 |
| Rpl38 | 230,70 | 411,71 | 429,79 | 190,68 | 444,70 | 482,91 | 428,30 | 487,09 | 0,56 | 7,16 | 2,66E-17 | 2,27E-14 |
| Fosb | 2,49 | 1,95 | 1,38 | 4,61 | 4,18 | 3,74 | 5,43 | 6,98 | 0,92 | 3,91 | 7,29E-17 | 5,71E-14 |
| Hpgd | 5,19 | 3,68 | 2,81 | 2,71 | 1,68 | 2,32 | 0,66 | 0,72 | -1,44 | 2,09 | 9,33E-17 | 6,75E-14 |
| Hist1h3c | 144,60 | 226,35 | 219,28 | 151,08 | 110,70 | 123,01 | 157,39 | 112,76 | -0,55 | 6,22 | 2,68E-16 | 1,80E-13 |
| Cd74 | 19,08 | 15,48 | 10,43 | 9,81 | 9,52 | 11,86 | 5,58 | 2,27 | -0,92 | 3,90 | 3,92E-16 | 2,46E-13 |
| Ncapg2 | 5,86 | 8,97 | 5,67 | 6,65 | 3,60 | 3,87 | 5,37 | 4,37 | -0,67 | 5,20 | 8,80E-16 | 5,17E-13 |
| Ppp1r15a | 28,24 | 31,63 | 25,25 | 27,05 | 42,31 | 39,30 | 37,54 | 43,18 | 0,53 | 6,33 | 1,88E-15 | 1,04E-12 |
| Ccna2 | 17,23 | 30,02 | 22,37 | 21,35 | 12,44 | 13,26 | 19,48 | 15,68 | -0,58 | 5,74 | 3,09E-15 | 1,62E-12 |
| Rrm2 | 22,93 | 39,12 | 25,72 | 29,78 | 18,14 | 18,00 | 23,33 | 19,52 | -0,58 | 5,74 | 3,91E-15 | 1,93E-12 |
| Hist1h1b | 106,07 | 163,87 | 134,26 | 109,60 | 79,39 | 81,87 | 106,55 | 88,79 | -0,53 | 6,19 | 8,11E-15 | 3,64E-12 |
| Trf | 7,77 | 5,09 | 4,99 | 4,77 | 4,04 | 4,93 | 1,69 | 0,70 | -1,00 | 3,30 | 8,13E-15 | 3,64E-12 |
| Emr4 | 2,56 | 1,81 | 1,14 | 1,31 | 1,17 | 1,01 | 0,34 | 0,21 | -1,33 | 2,08 | 1,08E-14 | 4,61E-12 |
| Marcks | 3,99 | 2,62 | 2,76 | 2,50 | 1,95 | 2,70 | 0,81 | 0,48 | -1,00 | 3,21 | 2,78E-14 | 1,14E-11 |
| Sirpa | 5,05 | 3,44 | 3,65 | 3,35 | 2,95 | 3,43 | 1,16 | 0,66 | -0,92 | 3,55 | 3,56E-14 | 1,40E-11 |
| Mpeg1 | 4,93 | 3,64 | 2,68 | 2,61 | 2,58 | 3,35 | 0,97 | 0,46 | -0,93 | 3,53 | 3,92E-14 | 1,48E-11 |
| Hist1h3e | 151,18 | 198,17 | 221,12 | 157,48 | 121,43 | 129,93 | 144,60 | 106,77 | -0,52 | 5,98 | 4,34E-14 | 1,57E-11 |
| Nlrp1a | 3,78 | 5,74 | 4,64 | 3,35 | 6,86 | 7,11 | 6,52 | 7,75 | 0,69 | 4,80 | 4,72E-14 | 1,64E-11 |
| Sdc3 | 8,04 | 5,47 | 4,87 | 4,93 | 4,85 | 5,42 | 2,57 | 1,34 | -0,72 | 4,54 | 6,26E-14 | 2,10E-11 |
| Casc5 | 6,88 | 8,29 | 6,97 | 7,21 | 4,02 | 4,07 | 5,94 | 5,32 | -0,60 | 5,31 | 6,66E-14 | 2,16E-11 |
| C1qb | 12,79 | 8,72 | 9,26 | 7,16 | 5,79 | 7,87 | 2,95 | 1,54 | -1,06 | 2,88 | 8,01E-14 | 2,51E-11 |
| Hist1h3d | 151,31 | 213,67 | 196,27 | 164,52 | 119,55 | 123,44 | 144,93 | 118,57 | -0,52 | 5,98 | 1,37E-13 | 4,17E-11 |
| Rpl36 | 178,41 | 335,58 | 367,82 | 157,82 | 334,89 | 399,32 | 307,55 | 400,04 | 0,48 | 6,98 | 1,75E-13 | 5,15E-11 |
| Fcna | 7,80 | 4,99 | 5,10 | 3,72 | 3,14 | 4,90 | 1,24 | 0,38 | -1,16 | 2,35 | 4,89E-13 | 1,39E-10 |
| Rpl11 | 298,69 | 463,50 | 383,85 | 277,61 | 487,40 | 474,28 | 450,81 | 565,11 | 0,48 | 7,98 | 1,18E-12 | 3,28E-10 |
| Tgm2 | 3,12 | 2,57 | 2,11 | 1,71 | 1,49 | 1,94 | 0,72 | 0,38 | -1,08 | 2,63 | 1,25E-12 | 3,36E-10 |
| Zfp488 | 69,87 | 36,25 | 37,27 | 45,78 | 34,04 | 34,65 | 35,54 | 33,35 | -0,46 | 7,46 | 1,64E-12 | 4,27E-10 |
| Kif11 | 16,37 | 23,06 | 20,33 | 20,04 | 12,95 | 13,08 | 17,15 | 14,59 | -0,47 | 6,37 | 1,95E-12 | 4,95E-10 |
| C1qc | 17,86 | 13,16 | 10,32 | 9,12 | 9,55 | 12,67 | 3,14 | 2,07 | -0,89 | 3,36 | 2,37E-12 | 5,85E-10 |
| Rps21 | 201,31 | 354,45 | 380,46 | 193,12 | 365,65 | 423,14 | 341,38 | 411,60 | 0,46 | 7,03 | 2,59E-12 | 6,23E-10 |
| Kif14 | 1,96 | 3,01 | 2,14 | 2,40 | 1,17 | 1,17 | 1,92 | 1,45 | -0,74 | 4,09 | 3,52E-12 | 8,28E-10 |
| Zfp36 | 47,90 | 64,92 | 60,71 | 45,95 | 76,96 | 67,91 | 75,65 | 78,88 | 0,45 | 6,84 | 4,11E-12 | 9,42E-10 |
| Tnfaip3 | 135,07 | 117,22 | 66,06 | 119,33 | 141,89 | 135,47 | 145,90 | 182,25 | 0,47 | 9,17 | 6,57E-12 | 1,47E-09 |
| Ccnb2 | 20,82 | 37,24 | 29,03 | 23,86 | 15,10 | 15,89 | 23,22 | 20,48 | -0,57 | 5,16 | 6,83E-12 | 1,49E-09 |
| Dnaja1 | 30,49 | 33,69 | 34,30 | 29,43 | 43,75 | 43,37 | 37,75 | 50,03 | 0,45 | 7,74 | 9,17E-12 | 1,96E-09 |
| Emr1 | 4,12 | 2,27 | 1,92 | 2,02 | 2,12 | 2,10 | 0,66 | 0,24 | -1,02 | 2,66 | 1,10E-11 | 2,30E-09 |
| Hist1h3h | 109,24 | 157,66 | 157,16 | 106,81 | 85,70 | 97,04 | 104,76 | 88,10 | -0,49 | 5,78 | 1,17E-11 | 2,39E-09 |
| Kif15 | 7,15 | 10,71 | 9,10 | 7,17 | 4,75 | 5,23 | 7,56 | 5,43 | -0,57 | 5,10 | 1,36E-11 | 2,73E-09 |
| Fxyd5 | 139,35 | 225,22 | 193,52 | 128,62 | 235,99 | 222,32 | 230,59 | 246,43 | 0,45 | 7,89 | 1,97E-11 | 3,84E-09 |
| Rpl29 | 337,40 | 492,00 | 500,56 | 302,69 | 573,50 | 534,95 | 566,37 | 558,77 | 0,45 | 8,42 | 2,00E-11 | 3,84E-09 |
| Fth1 | 101,38 | 169,65 | 153,00 | 84,38 | 171,46 | 180,93 | 155,15 | 177,36 | 0,44 | 7,14 | 2,67E-11 | 5,02E-09 |
| Rpl21 | 66,64 | 127,67 | 114,14 | 63,06 | 119,87 | 122,31 | 117,12 | 143,83 | 0,44 | 7,67 | 2,88E-11 | 5,30E-09 |
| Socs3 | 8,46 | 10,25 | 8,58 | 9,07 | 13,39 | 11,87 | 12,52 | 16,55 | 0,57 | 4,96 | 4,42E-11 | 7,99E-09 |
| Hist1h3g | 89,23 | 136,96 | 133,01 | 86,61 | 68,91 | 75,77 | 96,06 | 72,50 | -0,50 | 5,54 | 5,46E-11 | 9,69E-09 |
| Nr4a2 | 17,65 | 11,77 | 7,21 | 14,81 | 18,60 | 16,36 | 18,46 | 19,48 | 0,48 | 5,64 | 6,29E-11 | 1,09E-08 |
| Rpl19 | 195,83 | 326,36 | 331,00 | 195,48 | 332,75 | 355,07 | 349,69 | 376,11 | 0,44 | 7,86 | 7,76E-11 | 1,33E-08 |
| Mki67 | 39,89 | 54,39 | 47,09 | 41,56 | 30,46 | 28,38 | 41,49 | 34,43 | -0,44 | 8,64 | 8,93E-11 | 1,50E-08 |
| Rpl36a | 88,14 | 156,44 | 138,08 | 86,54 | 159,52 | 146,40 | 152,08 | 179,07 | 0,45 | 6,08 | 1,05E-10 | 1,73E-08 |
| Top2a | 39,86 | 62,65 | 49,76 | 49,45 | 34,28 | 34,00 | 43,35 | 38,63 | -0,43 | 7,84 | 1,56E-10 | 2,53E-08 |
| Nusap1 | 8,00 | 13,48 | 12,40 | 10,37 | 6,32 | 6,38 | 9,02 | 7,72 | -0,58 | 4,76 | 1,79E-10 | 2,85E-08 |
| Hmmr | 8,46 | 12,12 | 9,71 | 9,98 | 5,76 | 5,60 | 9,07 | 7,24 | -0,54 | 5,05 | 1,84E-10 | 2,88E-08 |
| Slc11a1 | 2,81 | 1,62 | 1,64 | 1,32 | 1,18 | 1,25 | 0,55 | 0,26 | -1,19 | 1,62 | 2,24E-10 | 3,46E-08 |
| Cit | 5,52 | 7,53 | 5,70 | 5,34 | 3,80 | 3,96 | 4,80 | 4,19 | -0,53 | 5,14 | 2,83E-10 | 4,29E-08 |
| Syk | 2,19 | 1,38 | 1,19 | 1,30 | 1,03 | 1,08 | 0,69 | 0,35 | -0,96 | 2,56 | 5,27E-10 | 7,87E-08 |
| C1qa | 8,60 | 8,33 | 5,33 | 4,58 | 4,69 | 6,43 | 1,76 | 0,87 | -0,98 | 2,47 | 5,66E-10 | 8,31E-08 |
| Tmsb10 | 498,20 | 1003,68 | 909,48 | 452,23 | 868,40 | 940,13 | 943,24 | 1076,22 | 0,42 | 9,06 | 6,87E-10 | 9,94E-08 |
| Brca1 | 2,42 | 3,87 | 2,67 | 3,15 | 1,80 | 1,95 | 2,29 | 1,68 | -0,66 | 4,05 | 1,03E-09 | 1,46E-07 |
| Kntc1 | 2,55 | 3,03 | 2,58 | 2,63 | 1,37 | 1,67 | 1,93 | 1,81 | -0,68 | 3,93 | 1,16E-09 | 1,62E-07 |
| Stip1 | 33,64 | 40,21 | 38,12 | 31,39 | 50,96 | 45,28 | 41,26 | 51,34 | 0,40 | 6,50 | 1,17E-09 | 1,62E-07 |
| Mafb | 2,59 | 1,43 | 1,45 | 1,45 | 1,08 | 1,68 | 0,47 | 0,22 | -1,02 | 2,14 | 1,38E-09 | 1,89E-07 |
| Asb2 | 2,44 | 2,10 | 2,64 | 1,49 | 1,36 | 1,44 | 0,59 | 0,85 | -1,01 | 2,09 | 1,58E-09 | 2,12E-07 |
| Hist1h2ak | 106,67 | 153,38 | 143,18 | 97,57 | 76,85 | 94,61 | 101,55 | 85,98 | -0,47 | 5,39 | 1,65E-09 | 2,18E-07 |
| Hist2h3c2 | 48,60 | 85,67 | 75,10 | 47,01 | 44,88 | 43,51 | 55,59 | 45,25 | -0,43 | 5,82 | 1,94E-09 | 2,53E-07 |
| Cenpf | 4,05 | 5,46 | 4,90 | 4,33 | 2,99 | 2,83 | 4,30 | 3,44 | -0,46 | 5,49 | 2,03E-09 | 2,62E-07 |
| Rpl23 | 284,58 | 462,06 | 415,59 | 274,16 | 475,78 | 465,04 | 440,76 | 521,61 | 0,41 | 8,79 | 2,26E-09 | 2,88E-07 |
| Ccr3 | 5,85 | 5,21 | 4,88 | 5,32 | 4,25 | 3,97 | 2,70 | 2,39 | -0,68 | 3,82 | 2,32E-09 | 2,91E-07 |
| Rpl23a | 692,27 | 1170,29 | 1107,10 | 637,43 | 1154,97 | 1181,63 | 1131,95 | 1305,68 | 0,41 | 9,19 | 2,58E-09 | 3,20E-07 |
| Sik1 | 22,21 | 21,55 | 14,37 | 21,24 | 24,84 | 23,08 | 28,00 | 28,20 | 0,38 | 6,70 | 2,69E-09 | 3,28E-07 |
| Rpl10a | 201,71 | 287,31 | 264,91 | 185,91 | 314,81 | 286,80 | 305,78 | 324,62 | 0,39 | 7,82 | 3,78E-09 | 4,56E-07 |
| Uhrf1 | 12,96 | 23,56 | 15,44 | 15,99 | 11,46 | 12,19 | 14,10 | 12,64 | -0,44 | 5,72 | 3,94E-09 | 4,69E-07 |
| Prr11 | 6,73 | 7,84 | 7,24 | 7,24 | 4,26 | 4,25 | 5,55 | 5,58 | -0,57 | 4,54 | 4,18E-09 | 4,92E-07 |
| Hist1h2bk | 72,42 | 108,17 | 118,40 | 76,06 | 59,62 | 65,98 | 70,78 | 64,02 | -0,51 | 4,91 | 4,42E-09 | 5,14E-07 |
| Dusp1 | 27,07 | 25,44 | 23,33 | 23,51 | 33,81 | 30,74 | 31,77 | 37,02 | 0,42 | 5,83 | 4,98E-09 | 5,72E-07 |
| Depdc1a | 4,91 | 6,30 | 4,70 | 5,31 | 3,07 | 2,73 | 4,26 | 3,42 | -0,66 | 3,85 | 5,11E-09 | 5,79E-07 |
| Hist1h3a | 85,21 | 123,16 | 114,05 | 84,20 | 65,02 | 77,36 | 86,72 | 68,47 | -0,45 | 5,55 | 5,20E-09 | 5,82E-07 |
| Axl | 11,36 | 9,39 | 7,74 | 7,58 | 7,10 | 8,35 | 5,42 | 5,25 | -0,47 | 5,31 | 5,53E-09 | 6,12E-07 |
| Plbd1 | 2,71 | 2,31 | 1,49 | 1,79 | 1,16 | 1,84 | 0,76 | 0,18 | -1,11 | 1,62 | 5,70E-09 | 6,23E-07 |
| Espl1 | 3,98 | 5,55 | 4,46 | 4,61 | 2,93 | 3,10 | 3,70 | 3,07 | -0,54 | 4,70 | 5,87E-09 | 6,35E-07 |
| Rps12 | 211,51 | 390,26 | 381,93 | 180,04 | 368,53 | 368,61 | 359,44 | 412,50 | 0,38 | 7,44 | 7,01E-09 | 7,49E-07 |
| Lyz2 | 5,26 | 2,46 | 3,31 | 3,53 | 2,36 | 2,67 | 1,25 | 0,42 | -1,11 | 1,50 | 7,43E-09 | 7,85E-07 |
| Rps28 | 219,77 | 362,23 | 327,96 | 193,28 | 353,93 | 342,64 | 338,22 | 393,52 | 0,38 | 6,69 | 7,97E-09 | 8,33E-07 |
| Kif20a | 6,38 | 10,24 | 8,14 | 9,12 | 5,42 | 5,04 | 7,03 | 5,93 | -0,54 | 4,68 | 9,60E-09 | 9,91E-07 |
| Hist1h3i | 124,88 | 173,81 | 173,28 | 123,04 | 103,95 | 113,59 | 125,02 | 107,55 | -0,40 | 5,97 | 1,21E-08 | 1,23E-06 |
| Hist2h2ac | 127,78 | 171,15 | 166,83 | 124,22 | 110,10 | 103,34 | 124,33 | 100,21 | -0,42 | 5,64 | 1,44E-08 | 1,45E-06 |
| Hist1h2ae | 51,72 | 78,10 | 68,91 | 45,76 | 37,46 | 46,37 | 49,98 | 42,87 | -0,46 | 5,21 | 1,50E-08 | 1,49E-06 |
| Clspn | 4,05 | 6,58 | 4,16 | 4,08 | 2,63 | 3,05 | 3,69 | 3,36 | -0,58 | 4,30 | 1,50E-08 | 1,49E-06 |
| Rrm1 | 18,49 | 25,00 | 17,63 | 20,77 | 14,38 | 15,59 | 17,49 | 15,46 | -0,39 | 6,18 | 1,52E-08 | 1,49E-06 |
| Pbk | 2,86 | 4,17 | 2,53 | 2,68 | 1,28 | 1,87 | 1,57 | 1,53 | -0,99 | 1,95 | 1,74E-08 | 1,69E-06 |
| Hist1h2ap | 92,66 | 161,98 | 137,88 | 86,71 | 78,06 | 88,07 | 104,67 | 79,31 | -0,45 | 5,34 | 1,91E-08 | 1,83E-06 |
| Hist1h2ao | 70,95 | 124,33 | 106,35 | 66,40 | 60,18 | 67,73 | 80,29 | 60,83 | -0,44 | 5,35 | 1,99E-08 | 1,89E-06 |
| Hist2h4 | 83,59 | 75,62 | 85,60 | 74,96 | 56,50 | 52,84 | 67,84 | 42,51 | -0,53 | 4,55 | 2,07E-08 | 1,95E-06 |
| Nhsl2 | 9,21 | 7,75 | 7,06 | 10,01 | 6,10 | 5,97 | 8,51 | 5,92 | -0,37 | 6,62 | 2,19E-08 | 2,03E-06 |
| Grk1 | 7,73 | 4,07 | 4,14 | 4,84 | 3,85 | 3,40 | 4,30 | 3,44 | -0,48 | 5,06 | 2,31E-08 | 2,12E-06 |
| Ckap2l | 6,10 | 8,87 | 7,87 | 6,80 | 4,13 | 4,11 | 6,41 | 5,30 | -0,57 | 4,28 | 2,33E-08 | 2,12E-06 |
| Atn1 | 2,99 | 3,44 | 4,22 | 2,19 | 4,52 | 5,25 | 3,83 | 5,57 | 0,60 | 4,14 | 2,46E-08 | 2,22E-06 |
| Tpx2 | 7,01 | 12,75 | 8,97 | 7,72 | 5,23 | 5,47 | 8,00 | 7,65 | -0,47 | 5,10 | 2,52E-08 | 2,25E-06 |
| Ccnb1 | 8,08 | 11,53 | 8,90 | 9,97 | 5,98 | 5,96 | 7,65 | 6,41 | -0,57 | 4,22 | 3,28E-08 | 2,91E-06 |
| Gltscr1 | 19,42 | 22,85 | 23,38 | 18,65 | 27,27 | 26,11 | 26,66 | 27,84 | 0,36 | 7,01 | 3,44E-08 | 3,02E-06 |
| Hist1h2ag | 69,19 | 115,83 | 106,77 | 72,98 | 57,40 | 67,00 | 80,63 | 60,66 | -0,45 | 5,21 | 3,54E-08 | 3,08E-06 |
| Rpl41 | 512,93 | 732,37 | 614,81 | 530,96 | 774,73 | 731,24 | 738,76 | 841,04 | 0,37 | 8,27 | 4,40E-08 | 3,79E-06 |
| Thy1 | 787,16 | 1049,08 | 934,85 | 789,00 | 1171,66 | 1129,52 | 1189,44 | 1121,32 | 0,37 | 10,79 | 4,46E-08 | 3,81E-06 |
| Hist1h2af | 72,66 | 114,72 | 119,11 | 78,98 | 60,54 | 67,04 | 79,14 | 70,24 | -0,47 | 5,01 | 4,65E-08 | 3,94E-06 |
| Sf3a2 | 24,02 | 39,35 | 32,48 | 25,69 | 39,81 | 38,61 | 40,17 | 40,58 | 0,39 | 5,88 | 4,85E-08 | 4,07E-06 |
| Ndufa13 | 8,90 | 17,26 | 17,57 | 9,70 | 19,19 | 18,92 | 19,12 | 20,24 | 0,56 | 4,36 | 5,65E-08 | 4,70E-06 |
| Per1 | 27,46 | 26,20 | 21,73 | 24,78 | 31,78 | 29,38 | 33,02 | 33,69 | 0,35 | 7,06 | 6,20E-08 | 5,11E-06 |
| Hist1h2bm | 43,79 | 70,87 | 65,28 | 43,37 | 34,33 | 35,44 | 41,35 | 39,21 | -0,56 | 4,14 | 7,28E-08 | 5,95E-06 |
| Hist1h1d | 60,85 | 79,81 | 90,38 | 56,15 | 53,29 | 59,45 | 60,30 | 51,55 | -0,35 | 7,57 | 8,10E-08 | 6,49E-06 |
| Rps9 | 363,35 | 518,44 | 475,81 | 370,71 | 534,60 | 546,51 | 526,80 | 613,45 | 0,36 | 8,44 | 8,14E-08 | 6,49E-06 |
| Zc3h12a | 17,96 | 17,38 | 14,73 | 17,45 | 21,67 | 21,82 | 20,99 | 24,30 | 0,39 | 5,79 | 8,14E-08 | 6,49E-06 |
| Lgmn | 4,37 | 3,16 | 2,83 | 1,78 | 1,86 | 2,98 | 1,04 | 0,66 | -0,90 | 2,13 | 8,49E-08 | 6,71E-06 |
| Ptgs1 | 2,50 | 1,52 | 1,52 | 1,56 | 1,38 | 1,13 | 0,84 | 0,40 | -0,92 | 1,97 | 8,95E-08 | 7,00E-06 |
| Hist2h3c1 | 26,55 | 48,95 | 44,36 | 26,28 | 26,00 | 26,31 | 32,81 | 26,59 | -0,38 | 5,87 | 9,02E-08 | 7,00E-06 |
| Hist1h2ai | 80,14 | 127,57 | 107,56 | 76,36 | 59,12 | 71,66 | 82,83 | 71,88 | -0,45 | 5,05 | 1,01E-07 | 7,77E-06 |
| Hist1h2be | 24,52 | 31,78 | 32,87 | 22,68 | 19,93 | 20,81 | 23,55 | 21,76 | -0,37 | 5,95 | 1,06E-07 | 8,11E-06 |
| Trp53 | 32,20 | 39,82 | 37,97 | 33,70 | 48,48 | 46,12 | 42,73 | 46,43 | 0,36 | 6,26 | 1,22E-07 | 9,25E-06 |
| Hist1h2bj | 54,03 | 94,54 | 76,70 | 53,02 | 40,65 | 51,83 | 53,50 | 48,69 | -0,51 | 4,49 | 1,28E-07 | 9,66E-06 |
| Spic | 3,64 | 2,52 | 2,92 | 2,21 | 2,01 | 2,47 | 0,36 | 0,66 | -1,03 | 1,41 | 1,51E-07 | 1,13E-05 |
| Rgs10 | 8,80 | 9,80 | 10,38 | 6,95 | 11,65 | 14,16 | 11,83 | 19,17 | 0,67 | 3,36 | 1,53E-07 | 1,13E-05 |
| Nuf2 | 4,22 | 6,56 | 5,86 | 5,34 | 2,76 | 3,35 | 4,25 | 3,64 | -0,65 | 3,43 | 1,59E-07 | 1,17E-05 |
| Fau | 485,17 | 679,77 | 661,48 | 491,46 | 801,33 | 701,48 | 730,00 | 730,40 | 0,36 | 8,92 | 1,78E-07 | 1,30E-05 |
| Adam4 | 4,61 | 1,43 | 1,30 | 1,49 | 1,38 | 1,12 | 1,17 | 1,17 | -0,89 | 2,03 | 2,30E-07 | 1,66E-05 |
| Ccl5 | 3563,69 | 4415,49 | 4136,15 | 3480,90 | 5509,59 | 4890,44 | 4799,06 | 4686,53 | 0,35 | 11,20 | 2,81E-07 | 2,02E-05 |
| Lyn | 3,50 | 2,35 | 2,30 | 2,00 | 1,82 | 2,42 | 1,16 | 0,73 | -0,73 | 2,81 | 3,07E-07 | 2,19E-05 |
| Hist1h2bn | 300,48 | 451,61 | 444,06 | 299,99 | 254,31 | 281,25 | 350,82 | 301,64 | -0,33 | 7,00 | 3,30E-07 | 2,33E-05 |
| Smc2 | 14,67 | 20,53 | 18,06 | 18,19 | 12,96 | 13,29 | 15,83 | 14,54 | -0,34 | 6,41 | 3,48E-07 | 2,44E-05 |
| Hist1h1a | 45,10 | 73,74 | 58,49 | 46,34 | 37,90 | 41,68 | 49,23 | 38,21 | -0,42 | 5,19 | 3,50E-07 | 2,44E-05 |
| Olfr613 | 20,07 | 9,38 | 9,32 | 12,35 | 9,25 | 9,62 | 11,07 | 9,94 | -0,37 | 5,87 | 3,64E-07 | 2,52E-05 |
| Iqgap3 | 1,22 | 1,63 | 1,58 | 1,17 | 0,71 | 0,69 | 1,10 | 0,79 | -0,75 | 2,65 | 3,81E-07 | 2,62E-05 |
| Cd5l | 4,02 | 2,69 | 2,92 | 2,33 | 2,01 | 3,36 | 0,94 | 0,44 | -0,83 | 2,25 | 3,90E-07 | 2,66E-05 |
| Tcf7 | 247,12 | 305,19 | 289,84 | 265,54 | 346,87 | 333,52 | 355,52 | 370,85 | 0,35 | 9,46 | 3,94E-07 | 2,66E-05 |
| Pdcd5 | 6,50 | 16,72 | 17,23 | 5,88 | 16,82 | 16,95 | 16,62 | 21,65 | 0,68 | 3,26 | 4,37E-07 | 2,94E-05 |
| Plk1 | 9,32 | 16,75 | 10,47 | 11,42 | 7,68 | 6,70 | 10,28 | 9,66 | -0,49 | 4,51 | 4,44E-07 | 2,94E-05 |
| Ier2 | 54,21 | 59,47 | 60,71 | 58,79 | 70,47 | 68,25 | 74,78 | 79,28 | 0,33 | 6,65 | 4,44E-07 | 2,94E-05 |
| Junb | 137,84 | 155,36 | 158,20 | 155,33 | 193,31 | 185,80 | 183,06 | 205,04 | 0,34 | 8,30 | 4,80E-07 | 3,16E-05 |
| Ctla4 | 80,69 | 78,24 | 55,87 | 76,20 | 84,56 | 84,78 | 87,70 | 108,21 | 0,32 | 7,31 | 5,50E-07 | 3,59E-05 |
| Nr4a1 | 17,04 | 16,51 | 11,61 | 17,23 | 21,36 | 18,60 | 21,84 | 20,22 | 0,38 | 5,50 | 5,70E-07 | 3,70E-05 |
| Gmfg | 91,95 | 142,59 | 140,63 | 83,10 | 145,07 | 146,23 | 138,06 | 142,76 | 0,33 | 7,02 | 5,95E-07 | 3,83E-05 |
| Bub1b | 6,82 | 11,39 | 8,43 | 8,74 | 5,22 | 5,86 | 8,04 | 6,81 | -0,45 | 4,81 | 5,98E-07 | 3,83E-05 |
| Mcm5 | 18,91 | 27,83 | 20,31 | 21,15 | 17,14 | 15,84 | 19,15 | 17,55 | -0,34 | 6,08 | 6,22E-07 | 3,95E-05 |
| Cep55 | 5,66 | 8,32 | 7,23 | 6,52 | 3,94 | 4,00 | 5,62 | 5,52 | -0,54 | 4,01 | 6,47E-07 | 4,09E-05 |
| Zfp36l1 | 76,95 | 90,43 | 87,19 | 80,17 | 109,22 | 99,53 | 100,71 | 112,13 | 0,33 | 8,14 | 6,62E-07 | 4,15E-05 |
| Tuba1b | 78,39 | 96,92 | 72,90 | 88,59 | 65,53 | 61,31 | 72,58 | 70,60 | -0,32 | 6,89 | 6,68E-07 | 4,16E-05 |
| Diap3 | 6,69 | 8,40 | 6,16 | 7,07 | 4,66 | 4,79 | 5,76 | 5,04 | -0,49 | 4,42 | 6,85E-07 | 4,24E-05 |
| E2f7 | 1,27 | 2,19 | 1,68 | 1,73 | 0,86 | 1,06 | 1,19 | 1,18 | -0,68 | 2,93 | 8,68E-07 | 5,33E-05 |
| Pole | 4,37 | 6,16 | 4,92 | 5,23 | 3,99 | 3,32 | 4,55 | 3,59 | -0,42 | 5,01 | 9,07E-07 | 5,54E-05 |
| Hist1h2ad | 81,64 | 132,06 | 109,25 | 80,97 | 66,01 | 73,84 | 83,41 | 80,06 | -0,41 | 5,11 | 9,36E-07 | 5,67E-05 |
| Colec12 | 24,26 | 15,12 | 16,80 | 17,18 | 14,43 | 13,77 | 15,99 | 13,08 | -0,36 | 5,76 | 9,80E-07 | 5,90E-05 |
| Rpl28 | 456,56 | 702,20 | 587,25 | 424,30 | 684,75 | 641,94 | 678,10 | 720,25 | 0,33 | 8,25 | 9,88E-07 | 5,92E-05 |
| Sgol2 | 2,28 | 3,66 | 2,79 | 2,88 | 1,73 | 1,51 | 2,64 | 1,96 | -0,57 | 3,69 | 9,97E-07 | 5,93E-05 |
| Lmnb1 | 52,46 | 74,35 | 64,60 | 55,55 | 45,16 | 48,29 | 54,39 | 50,34 | -0,32 | 7,30 | 1,02E-06 | 6,00E-05 |
| Hist1h4f | 75,07 | 112,31 | 115,18 | 79,05 | 53,09 | 72,02 | 80,62 | 71,20 | -0,45 | 4,67 | 1,02E-06 | 6,01E-05 |
| Vps13c | 19,51 | 12,84 | 13,43 | 18,07 | 13,50 | 11,73 | 13,71 | 12,37 | -0,32 | 7,38 | 1,12E-06 | 6,52E-05 |
| Hist1h2ah | 83,73 | 124,23 | 111,68 | 85,65 | 65,59 | 80,32 | 86,46 | 72,48 | -0,41 | 5,10 | 1,26E-06 | 7,29E-05 |
| Prkdc | 18,73 | 11,46 | 13,39 | 15,56 | 11,98 | 11,11 | 13,30 | 11,16 | -0,32 | 7,40 | 1,27E-06 | 7,33E-05 |
| Kif4 | 4,69 | 7,13 | 5,34 | 5,33 | 3,23 | 3,91 | 4,64 | 4,53 | -0,47 | 4,51 | 1,38E-06 | 7,93E-05 |
| Macf1 | 181,10 | 102,25 | 118,08 | 134,74 | 113,29 | 96,52 | 115,37 | 101,70 | -0,33 | 11,47 | 1,45E-06 | 8,27E-05 |
| H2-Ab1 | 3,63 | 3,70 | 2,85 | 3,00 | 2,24 | 2,66 | 1,52 | 0,66 | -0,90 | 1,62 | 1,48E-06 | 8,40E-05 |
| Hist2h3b | 159,90 | 240,74 | 217,90 | 149,61 | 150,22 | 138,15 | 175,86 | 146,49 | -0,32 | 6,14 | 1,51E-06 | 8,50E-05 |
| Edf1 | 34,05 | 70,25 | 66,72 | 37,97 | 63,35 | 68,30 | 66,58 | 73,58 | 0,39 | 5,33 | 1,53E-06 | 8,59E-05 |
| E2f8 | 4,42 | 8,15 | 5,84 | 4,86 | 3,72 | 4,00 | 4,80 | 3,98 | -0,50 | 4,20 | 1,59E-06 | 8,78E-05 |
| Prnp | 53,39 | 30,45 | 33,47 | 34,06 | 31,84 | 29,48 | 33,25 | 27,59 | -0,31 | 7,00 | 1,59E-06 | 8,78E-05 |
| Hist1h3f | 93,35 | 141,21 | 151,33 | 93,72 | 90,12 | 98,30 | 108,00 | 89,03 | -0,31 | 6,76 | 1,66E-06 | 9,11E-05 |
| Crtc2 | 26,93 | 30,53 | 32,91 | 27,57 | 38,28 | 35,80 | 34,80 | 37,58 | 0,32 | 6,44 | 1,70E-06 | 9,32E-05 |
| Rpl7a | 480,26 | 671,86 | 583,51 | 471,38 | 700,89 | 663,63 | 677,61 | 722,40 | 0,33 | 9,07 | 1,72E-06 | 9,35E-05 |
| Hist1h2ab | 78,09 | 154,86 | 123,73 | 79,27 | 70,91 | 87,72 | 98,34 | 81,64 | -0,36 | 5,61 | 1,74E-06 | 9,41E-05 |
| Lclat1 | 10,08 | 12,10 | 12,01 | 9,23 | 8,27 | 9,00 | 7,26 | 8,88 | -0,37 | 5,42 | 1,77E-06 | 9,50E-05 |
| Tyr | 2,25 | 1,05 | 1,14 | 1,35 | 0,94 | 0,72 | 0,86 | 0,76 | -0,83 | 1,93 | 1,88E-06 | 0,0001 |
| Fbrs | 41,12 | 50,62 | 53,92 | 42,87 | 60,62 | 58,67 | 54,84 | 59,05 | 0,31 | 7,13 | 1,92E-06 | 0,0001 |
| Fancd2 | 1,51 | 2,15 | 1,31 | 1,90 | 0,84 | 1,11 | 1,23 | 1,13 | -0,70 | 2,76 | 1,93E-06 | 0,0001 |
| Cenpe | 5,28 | 9,08 | 7,89 | 5,49 | 4,75 | 4,67 | 6,21 | 5,90 | -0,36 | 5,58 | 1,98E-06 | 0,0001 |
| Plxnb2 | 1,72 | 1,29 | 1,19 | 1,21 | 0,90 | 1,16 | 0,91 | 0,43 | -0,68 | 2,85 | 2,02E-06 | 0,0001 |
| Srgn | 159,16 | 223,46 | 203,15 | 147,36 | 213,88 | 222,47 | 243,27 | 229,33 | 0,31 | 7,59 | 2,22E-06 | 0,0001 |
| Arhgap19 | 5,35 | 7,95 | 7,03 | 6,62 | 4,53 | 4,33 | 5,72 | 5,61 | -0,41 | 4,92 | 2,36E-06 | 0,0001 |
| Wfikkn2 | 8,54 | 10,17 | 8,00 | 9,76 | 9,09 | 11,88 | 13,45 | 13,58 | 0,39 | 5,17 | 2,41E-06 | 0,0001 |
| Rpl18 | 661,67 | 804,97 | 725,08 | 645,52 | 892,24 | 886,06 | 834,95 | 929,21 | 0,32 | 9,08 | 2,44E-06 | 0,0001 |
| Uba52 | 384,26 | 599,69 | 590,39 | 377,28 | 602,87 | 623,53 | 570,28 | 628,33 | 0,32 | 8,08 | 2,54E-06 | 0,0001 |
| Rps23 | 587,52 | 729,98 | 638,48 | 506,68 | 810,26 | 761,60 | 684,71 | 814,84 | 0,32 | 8,59 | 2,61E-06 | 0,0001 |
| Kif18b | 4,98 | 8,67 | 6,83 | 6,01 | 4,95 | 3,93 | 5,68 | 4,40 | -0,48 | 4,24 | 2,69E-06 | 0,0001 |
| Hist1h2ac | 16,02 | 26,46 | 24,28 | 15,18 | 13,86 | 16,91 | 17,17 | 15,60 | -0,36 | 5,50 | 2,86E-06 | 0,0001 |
| Fos | 8,40 | 6,51 | 5,87 | 9,67 | 9,88 | 9,12 | 11,60 | 12,05 | 0,47 | 4,28 | 2,98E-06 | 0,0001 |
| Shfm1 | 23,85 | 46,21 | 46,80 | 24,97 | 45,65 | 52,02 | 42,81 | 54,21 | 0,48 | 4,37 | 3,19E-06 | 0,0002 |
| Hist1h2bc | 53,99 | 82,80 | 79,37 | 51,46 | 48,76 | 53,22 | 53,70 | 51,31 | -0,36 | 5,43 | 3,19E-06 | 0,0002 |
| Tyms | 23,46 | 15,21 | 14,84 | 17,80 | 14,92 | 13,72 | 14,70 | 13,60 | -0,33 | 5,93 | 3,37E-06 | 0,0002 |
| H2-Aa | 5,90 | 4,72 | 3,18 | 2,48 | 3,06 | 4,24 | 1,57 | 0,32 | -0,84 | 1,79 | 3,41E-06 | 0,0002 |
| Trip13 | 1,96 | 3,33 | 3,09 | 2,66 | 1,54 | 1,51 | 2,06 | 1,44 | -0,74 | 2,32 | 3,45E-06 | 0,0002 |
| Cdca8 | 11,35 | 17,18 | 12,21 | 12,27 | 8,44 | 8,86 | 10,61 | 10,22 | -0,48 | 4,21 | 3,49E-06 | 0,0002 |
| Blm | 3,36 | 3,89 | 3,34 | 3,41 | 2,08 | 2,60 | 2,61 | 2,50 | -0,52 | 3,86 | 3,62E-06 | 0,0002 |
| Rpl24 | 341,62 | 440,05 | 485,94 | 319,47 | 494,18 | 497,79 | 450,20 | 524,53 | 0,31 | 8,24 | 3,73E-06 | 0,0002 |
| Nucks1 | 43,08 | 50,05 | 44,57 | 49,97 | 35,00 | 35,89 | 40,90 | 39,82 | -0,31 | 8,01 | 3,79E-06 | 0,0002 |
| Eef1b2 | 63,43 | 84,02 | 73,88 | 61,64 | 84,83 | 86,79 | 83,59 | 93,06 | 0,30 | 7,25 | 3,80E-06 | 0,0002 |
| Rps24 | 496,65 | 633,28 | 635,62 | 380,67 | 700,20 | 668,70 | 636,61 | 661,19 | 0,31 | 9,04 | 3,85E-06 | 0,0002 |
| Hook3 | 14,96 | 10,31 | 11,30 | 13,94 | 10,31 | 9,73 | 11,07 | 9,97 | -0,30 | 7,20 | 3,90E-06 | 0,0002 |
| Rpl32 | 319,03 | 423,95 | 443,95 | 278,05 | 445,53 | 466,71 | 416,81 | 476,87 | 0,31 | 7,69 | 4,27E-06 | 0,0002 |
| Jun | 11,58 | 10,69 | 14,93 | 10,11 | 15,90 | 15,21 | 12,84 | 16,52 | 0,37 | 5,43 | 4,34E-06 | 0,0002 |
| Bard1 | 5,93 | 7,14 | 5,47 | 6,84 | 4,38 | 4,71 | 5,35 | 4,83 | -0,41 | 4,93 | 4,56E-06 | 0,0002 |
| Cox6c | 35,72 | 73,17 | 70,88 | 38,80 | 63,98 | 73,62 | 70,77 | 78,00 | 0,41 | 4,99 | 4,58E-06 | 0,0002 |
| Neil3 | 4,27 | 6,24 | 4,20 | 4,44 | 2,54 | 2,97 | 3,71 | 3,49 | -0,60 | 3,17 | 4,86E-06 | 0,0002 |
| Srp9 | 24,81 | 33,90 | 33,66 | 24,48 | 33,73 | 38,08 | 36,77 | 40,24 | 0,36 | 5,53 | 4,92E-06 | 0,0002 |
| Cdk1 | 4,66 | 8,21 | 6,58 | 6,43 | 4,02 | 4,25 | 5,32 | 4,76 | -0,50 | 3,98 | 4,93E-06 | 0,0002 |
| Cdca2 | 4,18 | 5,31 | 4,87 | 4,39 | 3,70 | 2,50 | 3,77 | 3,24 | -0,50 | 3,94 | 5,11E-06 | 0,0002 |
| Dlgap5 | 6,57 | 11,33 | 9,41 | 7,99 | 6,34 | 5,81 | 6,62 | 7,19 | -0,44 | 4,50 | 5,19E-06 | 0,0002 |
| Myo9a | 3,94 | 2,80 | 2,83 | 3,08 | 2,57 | 2,40 | 2,56 | 2,17 | -0,39 | 5,07 | 5,71E-06 | 0,0003 |
| Dnajb1 | 29,17 | 34,11 | 34,46 | 29,63 | 40,35 | 38,90 | 35,80 | 41,85 | 0,30 | 6,33 | 5,74E-06 | 0,0003 |
| Hist1h2an | 61,36 | 88,16 | 84,22 | 57,52 | 47,62 | 54,35 | 61,34 | 52,62 | -0,42 | 4,63 | 5,88E-06 | 0,0003 |
| Gps2 | 33,88 | 42,66 | 37,75 | 28,21 | 45,60 | 43,61 | 45,10 | 46,03 | 0,34 | 5,63 | 6,36E-06 | 0,0003 |
| Csrnp1 | 21,70 | 18,64 | 14,40 | 23,52 | 25,61 | 22,47 | 23,75 | 25,85 | 0,31 | 6,00 | 6,67E-06 | 0,0003 |
| Spag5 | 5,81 | 9,44 | 6,26 | 7,39 | 4,94 | 4,67 | 6,46 | 5,50 | -0,43 | 4,60 | 6,79E-06 | 0,0003 |
| Arpc3 | 108,84 | 153,49 | 145,42 | 99,92 | 154,19 | 147,69 | 156,12 | 161,78 | 0,29 | 6,90 | 7,29E-06 | 0,0003 |
| Gtse1 | 3,73 | 5,02 | 4,93 | 4,11 | 2,83 | 3,07 | 3,67 | 2,42 | -0,56 | 3,33 | 7,73E-06 | 0,0003 |
| Nckap5l | 9,56 | 11,07 | 13,03 | 10,09 | 14,99 | 13,78 | 11,59 | 13,97 | 0,32 | 5,90 | 8,48E-06 | 0,0004 |
| Mbd6 | 20,71 | 27,10 | 29,10 | 20,66 | 29,56 | 30,03 | 30,47 | 28,83 | 0,29 | 6,78 | 8,83E-06 | 0,0004 |
| Htt | 15,44 | 12,16 | 12,29 | 14,59 | 11,29 | 10,61 | 12,03 | 10,70 | -0,29 | 7,35 | 8,83E-06 | 0,0004 |
| Safb | 25,27 | 31,16 | 34,26 | 24,45 | 33,91 | 35,42 | 34,10 | 36,85 | 0,29 | 6,67 | 9,45E-06 | 0,0004 |
| Cd24a | 3,80 | 2,29 | 2,03 | 1,92 | 1,45 | 1,78 | 1,65 | 1,01 | -0,78 | 1,88 | 9,67E-06 | 0,0004 |
| Bub1 | 2,84 | 4,95 | 3,55 | 3,60 | 1,93 | 2,17 | 3,25 | 3,23 | -0,50 | 3,79 | 1,08E-05 | 0,0004 |
| Fcer1g | 8,37 | 7,67 | 7,33 | 3,80 | 6,02 | 5,74 | 2,83 | 1,05 | -0,77 | 1,82 | 1,08E-05 | 0,0004 |
| Ect2 | 5,83 | 6,76 | 6,65 | 6,88 | 4,00 | 4,58 | 5,59 | 5,32 | -0,42 | 4,54 | 1,08E-05 | 0,0004 |
| S100a11 | 105,35 | 175,80 | 162,12 | 102,54 | 150,67 | 155,64 | 192,87 | 169,03 | 0,30 | 6,26 | 1,09E-05 | 0,0005 |
| Dnah8 | 28,70 | 17,04 | 19,70 | 21,73 | 19,30 | 16,94 | 18,14 | 16,72 | -0,29 | 8,17 | 1,11E-05 | 0,0005 |
| Sbds | 18,76 | 26,64 | 23,15 | 18,93 | 27,78 | 27,35 | 26,25 | 30,84 | 0,36 | 5,21 | 1,21E-05 | 0,0005 |
| Lcor | 11,77 | 6,22 | 6,67 | 8,00 | 6,86 | 5,97 | 6,44 | 6,08 | -0,37 | 5,08 | 1,22E-05 | 0,0005 |
| Cox4i1 | 152,86 | 208,22 | 192,62 | 153,67 | 211,51 | 209,16 | 201,33 | 238,11 | 0,28 | 7,06 | 1,23E-05 | 0,0005 |
| Pnn | 13,13 | 18,76 | 19,57 | 13,77 | 17,49 | 20,59 | 19,21 | 23,12 | 0,31 | 5,98 | 1,25E-05 | 0,0005 |
| Cfp | 10,00 | 7,49 | 8,81 | 7,43 | 6,61 | 9,02 | 4,29 | 3,21 | -0,54 | 3,43 | 1,25E-05 | 0,0005 |
| Anln | 3,80 | 4,11 | 3,35 | 3,31 | 2,52 | 2,22 | 3,44 | 2,40 | -0,46 | 4,10 | 1,28E-05 | 0,0005 |
| Bzrap1 | 3,99 | 3,37 | 3,45 | 3,82 | 2,42 | 2,57 | 3,61 | 2,41 | -0,41 | 4,61 | 1,32E-05 | 0,0005 |
| Nek2 | 3,09 | 5,67 | 3,64 | 4,91 | 2,11 | 2,76 | 3,20 | 4,12 | -0,53 | 3,57 | 1,54E-05 | 0,0006 |
| Rps17 | 178,35 | 248,34 | 238,42 | 173,06 | 230,10 | 262,42 | 228,92 | 295,27 | 0,28 | 6,78 | 1,56E-05 | 0,0006 |
| Rpl31 | 118,04 | 160,16 | 155,93 | 108,79 | 160,94 | 159,14 | 150,37 | 189,25 | 0,28 | 7,56 | 1,69E-05 | 0,0007 |
| Myl12b | 149,99 | 229,43 | 184,51 | 151,38 | 215,12 | 209,62 | 219,47 | 225,18 | 0,28 | 7,54 | 1,75E-05 | 0,0007 |
| Fignl1 | 4,49 | 5,72 | 4,93 | 4,58 | 3,24 | 3,71 | 3,14 | 3,83 | -0,51 | 3,64 | 1,81E-05 | 0,0007 |
| H2-Eb1 | 2,50 | 2,73 | 2,32 | 2,06 | 1,42 | 2,60 | 1,31 | 0,27 | -0,79 | 1,68 | 1,88E-05 | 0,0007 |
| Ccr7 | 19,22 | 20,42 | 19,73 | 21,45 | 24,44 | 23,11 | 23,13 | 31,06 | 0,33 | 5,50 | 1,98E-05 | 0,0008 |
| Kif22 | 14,95 | 18,50 | 15,03 | 16,91 | 12,26 | 10,89 | 13,73 | 13,59 | -0,38 | 4,90 | 1,99E-05 | 0,0008 |
| Atm | 23,70 | 16,90 | 18,03 | 21,04 | 16,59 | 15,24 | 17,49 | 16,29 | -0,28 | 7,76 | 2,21E-05 | 0,0009 |
| Aurka | 4,60 | 7,64 | 4,52 | 4,76 | 3,34 | 3,09 | 4,29 | 3,92 | -0,57 | 3,12 | 2,28E-05 | 0,0009 |
| Pfdn5 | 70,43 | 94,04 | 95,13 | 63,21 | 100,52 | 105,66 | 89,93 | 100,04 | 0,30 | 5,93 | 2,29E-05 | 0,0009 |
| Cdc20 | 9,92 | 15,25 | 13,04 | 13,77 | 7,98 | 8,47 | 12,24 | 10,04 | -0,43 | 4,34 | 2,33E-05 | 0,0009 |
| Rps15 | 633,47 | 896,20 | 933,20 | 619,02 | 932,61 | 963,39 | 907,16 | 955,91 | 0,29 | 8,80 | 2,35E-05 | 0,0009 |
| Rsad2 | 3,25 | 3,03 | 2,77 | 2,60 | 1,93 | 2,21 | 1,82 | 2,01 | -0,55 | 3,22 | 2,40E-05 | 0,0009 |
| Sart1 | 13,10 | 17,63 | 20,23 | 12,58 | 18,26 | 18,07 | 19,99 | 20,35 | 0,28 | 6,48 | 2,43E-05 | 0,0009 |
| Stx4a | 21,03 | 26,27 | 30,78 | 19,86 | 29,40 | 31,83 | 28,12 | 34,50 | 0,35 | 5,24 | 2,44E-05 | 0,0009 |
| Zfand2a | 17,44 | 14,01 | 10,38 | 18,60 | 18,23 | 18,12 | 17,76 | 21,28 | 0,30 | 5,73 | 2,49E-05 | 0,0009 |
| Mastl | 1,57 | 2,01 | 1,22 | 2,00 | 0,97 | 1,15 | 1,46 | 0,91 | -0,62 | 2,74 | 2,56E-05 | 0,0010 |
| Ldb1 | 95,89 | 106,13 | 113,96 | 94,44 | 129,11 | 127,05 | 114,97 | 127,14 | 0,28 | 8,02 | 2,62E-05 | 0,0010 |
| Stil | 2,40 | 3,45 | 2,25 | 2,67 | 1,77 | 1,67 | 2,33 | 1,90 | -0,50 | 3,63 | 2,65E-05 | 0,0010 |
| Rpl26 | 265,88 | 310,83 | 298,17 | 258,17 | 332,31 | 348,76 | 320,49 | 367,27 | 0,27 | 7,41 | 2,76E-05 | 0,0010 |
| Parpbp | 2,28 | 3,61 | 2,74 | 2,61 | 1,70 | 1,53 | 2,49 | 1,92 | -0,56 | 3,10 | 2,77E-05 | 0,0010 |
| Cd81 | 4,16 | 2,78 | 3,05 | 2,29 | 2,38 | 3,52 | 1,05 | 0,42 | -0,73 | 1,90 | 2,85E-05 | 0,0010 |
| Pif1 | 2,44 | 3,66 | 2,98 | 2,98 | 1,74 | 1,81 | 2,53 | 2,23 | -0,54 | 3,23 | 2,90E-05 | 0,0011 |
| Hist1h2bg | 133,98 | 211,88 | 216,97 | 126,61 | 123,27 | 146,06 | 160,71 | 139,01 | -0,27 | 6,59 | 2,97E-05 | 0,0011 |
| Rps27 | 731,14 | 930,99 | 1123,63 | 688,31 | 1097,75 | 1110,68 | 971,41 | 1037,79 | 0,28 | 8,38 | 3,00E-05 | 0,0011 |
| Rps27rt | 733,26 | 933,68 | 1126,89 | 690,30 | 1100,93 | 1113,90 | 974,23 | 1040,80 | 0,28 | 8,38 | 3,00E-05 | 0,0011 |
| Ncapd2 | 30,55 | 42,50 | 33,60 | 34,92 | 28,80 | 26,98 | 31,27 | 30,57 | -0,27 | 7,19 | 3,41E-05 | 0,0012 |
| Lyst | 46,95 | 31,44 | 34,64 | 38,03 | 32,32 | 29,38 | 33,61 | 29,00 | -0,28 | 8,67 | 3,46E-05 | 0,0012 |
| Cln3 | 34,42 | 29,99 | 32,25 | 31,19 | 41,88 | 40,39 | 35,74 | 36,16 | 0,27 | 6,46 | 3,72E-05 | 0,0013 |
| Birc6 | 94,24 | 60,33 | 64,33 | 80,75 | 64,67 | 56,58 | 65,26 | 60,20 | -0,28 | 10,07 | 3,78E-05 | 0,0013 |
| Gzmm | 29,15 | 35,21 | 34,34 | 23,75 | 39,61 | 36,86 | 37,88 | 38,28 | 0,33 | 5,47 | 3,80E-05 | 0,0013 |
| Gen1 | 2,41 | 3,10 | 2,62 | 2,84 | 1,53 | 1,92 | 1,87 | 2,08 | -0,58 | 2,92 | 3,91E-05 | 0,0014 |
| Gramd1a | 111,33 | 109,53 | 138,38 | 115,84 | 150,26 | 148,21 | 131,26 | 145,82 | 0,28 | 8,46 | 4,00E-05 | 0,0014 |
| Hist1h4a | 61,70 | 110,11 | 101,24 | 66,32 | 55,30 | 65,40 | 71,74 | 64,70 | -0,39 | 4,53 | 4,13E-05 | 0,0014 |
| Vps13b | 33,50 | 22,75 | 22,24 | 27,94 | 22,22 | 20,86 | 24,09 | 20,84 | -0,28 | 8,34 | 4,40E-05 | 0,0015 |
| Mis18bp1 | 4,03 | 4,60 | 4,33 | 3,52 | 2,43 | 2,43 | 4,12 | 2,98 | -0,46 | 3,83 | 4,56E-05 | 0,0016 |
| Nfkbia | 52,69 | 59,78 | 46,12 | 53,72 | 63,76 | 64,77 | 54,06 | 73,32 | 0,26 | 6,53 | 4,58E-05 | 0,0016 |
| Vps13d | 37,26 | 23,02 | 24,98 | 31,43 | 25,57 | 22,54 | 25,86 | 22,46 | -0,28 | 8,72 | 4,98E-05 | 0,0017 |
| Hspbap1 | 2,33 | 2,27 | 2,48 | 2,37 | 1,49 | 1,67 | 1,91 | 1,48 | -0,52 | 3,20 | 5,09E-05 | 0,0017 |
| Hist1h2bl | 34,67 | 57,62 | 52,13 | 36,94 | 30,60 | 33,06 | 35,87 | 32,72 | -0,45 | 3,89 | 5,14E-05 | 0,0018 |
| Dusp7 | 30,27 | 29,61 | 38,10 | 31,46 | 39,37 | 38,19 | 37,13 | 40,23 | 0,27 | 6,84 | 5,17E-05 | 0,0018 |
| Uqcrh | 89,61 | 109,41 | 109,99 | 92,59 | 114,57 | 123,18 | 119,91 | 131,48 | 0,29 | 5,93 | 5,19E-05 | 0,0018 |
| F2rl1 | 5,03 | 5,53 | 4,56 | 6,46 | 7,20 | 7,24 | 7,03 | 7,65 | 0,42 | 4,15 | 5,29E-05 | 0,0018 |
| Vmn1r58 | 12,41 | 8,81 | 8,75 | 8,23 | 7,63 | 6,67 | 7,89 | 6,96 | -0,39 | 4,50 | 5,36E-05 | 0,0018 |
| Sh3bgrl3 | 194,22 | 354,00 | 319,16 | 194,05 | 308,23 | 309,33 | 332,00 | 325,53 | 0,27 | 7,69 | 5,54E-05 | 0,0019 |
| Cdca5 | 4,35 | 5,25 | 2,98 | 5,09 | 1,99 | 2,17 | 4,36 | 3,50 | -0,59 | 2,85 | 5,59E-05 | 0,0019 |
| Apoe | 9,15 | 7,91 | 9,22 | 5,29 | 6,26 | 8,46 | 3,70 | 3,12 | -0,54 | 3,04 | 5,72E-05 | 0,0019 |
| Hist1h2bb | 48,44 | 77,59 | 68,10 | 45,89 | 35,85 | 45,75 | 51,32 | 51,16 | -0,38 | 4,63 | 5,73E-05 | 0,0019 |
| Ska1 | 1,34 | 1,82 | 1,49 | 1,40 | 0,73 | 0,85 | 1,22 | 0,83 | -0,74 | 1,71 | 5,95E-05 | 0,0020 |
| Arrb2 | 108,72 | 117,27 | 100,99 | 102,23 | 132,75 | 121,72 | 129,28 | 132,47 | 0,27 | 7,88 | 6,33E-05 | 0,0021 |
| Rbm17 | 20,32 | 29,36 | 25,34 | 19,91 | 29,37 | 28,44 | 30,13 | 30,35 | 0,32 | 5,36 | 6,48E-05 | 0,0021 |
| Ckap5 | 28,46 | 31,22 | 29,79 | 30,72 | 23,71 | 23,40 | 28,21 | 24,99 | -0,26 | 7,50 | 6,60E-05 | 0,0022 |
| Ndufb7 | 18,96 | 40,09 | 30,16 | 16,28 | 34,87 | 34,41 | 31,86 | 40,56 | 0,43 | 4,06 | 6,67E-05 | 0,0022 |
| Cd3g | 137,99 | 193,58 | 173,33 | 142,50 | 185,98 | 190,93 | 190,79 | 207,76 | 0,26 | 7,51 | 6,73E-05 | 0,0022 |
| Slc25a40 | 17,23 | 13,47 | 14,08 | 15,84 | 11,55 | 11,65 | 13,60 | 11,66 | -0,33 | 5,25 | 6,76E-05 | 0,0022 |
| Ppargc1b | 3,22 | 3,10 | 2,77 | 3,03 | 4,46 | 3,75 | 3,68 | 4,82 | 0,46 | 3,75 | 7,14E-05 | 0,0023 |
| Herc2 | 37,80 | 25,51 | 28,26 | 34,67 | 26,88 | 23,70 | 28,22 | 25,96 | -0,27 | 8,79 | 7,24E-05 | 0,0023 |
| Neurl1b | 1,26 | 1,84 | 1,45 | 1,77 | 0,87 | 0,80 | 1,42 | 1,30 | -0,54 | 3,06 | 7,28E-05 | 0,0023 |
| Cwc15 | 38,02 | 54,66 | 48,19 | 35,84 | 55,24 | 52,92 | 50,45 | 56,65 | 0,29 | 5,82 | 7,54E-05 | 0,0024 |
| Ccnf | 6,71 | 10,56 | 7,63 | 7,99 | 6,25 | 5,25 | 7,52 | 6,26 | -0,38 | 4,50 | 7,74E-05 | 0,0025 |
| Rpl13a | 635,72 | 801,83 | 728,91 | 644,94 | 864,06 | 861,32 | 779,04 | 881,98 | 0,27 | 9,63 | 7,87E-05 | 0,0025 |
| Cbx7 | 24,42 | 25,88 | 28,70 | 26,12 | 32,57 | 31,22 | 30,63 | 31,33 | 0,26 | 6,39 | 8,28E-05 | 0,0026 |
| Ubr4 | 62,71 | 39,12 | 45,56 | 54,78 | 45,97 | 38,34 | 44,40 | 39,26 | -0,27 | 9,52 | 8,36E-05 | 0,0026 |
| Kif2c | 4,21 | 6,94 | 4,51 | 4,63 | 2,84 | 3,28 | 4,24 | 4,44 | -0,47 | 3,64 | 8,37E-05 | 0,0026 |
| Rps27a | 200,73 | 263,48 | 275,76 | 177,20 | 271,09 | 279,27 | 260,19 | 286,23 | 0,26 | 7,69 | 8,38E-05 | 0,0026 |
| Dagla | 2,33 | 2,32 | 2,60 | 1,89 | 1,66 | 1,54 | 1,81 | 1,49 | -0,48 | 3,45 | 8,49E-05 | 0,0026 |
| Pnpla2 | 21,75 | 26,78 | 26,95 | 21,86 | 29,08 | 27,84 | 30,53 | 29,55 | 0,27 | 6,14 | 8,72E-05 | 0,0027 |
| Slc14a1 | 8,01 | 7,55 | 6,26 | 7,67 | 6,31 | 5,16 | 5,83 | 5,64 | -0,37 | 4,67 | 8,78E-05 | 0,0027 |
| Snrpe | 52,39 | 72,41 | 65,55 | 45,05 | 69,43 | 71,37 | 76,13 | 79,44 | 0,34 | 5,04 | 9,20E-05 | 0,0028 |
| Snrpg | 64,63 | 76,81 | 76,04 | 52,78 | 87,80 | 82,56 | 84,68 | 87,11 | 0,35 | 4,92 | 9,69E-05 | 0,0030 |
| Rps29 | 444,37 | 623,39 | 579,79 | 419,17 | 586,79 | 608,43 | 586,61 | 689,07 | 0,26 | 7,79 | 9,70E-05 | 0,0030 |
| Tmem45b | 7,16 | 3,89 | 3,60 | 5,32 | 3,47 | 3,58 | 3,14 | 3,42 | -0,57 | 2,75 | 0,0001 | 0,0031 |
| Rpl37 | 172,63 | 210,61 | 228,08 | 173,74 | 233,39 | 245,10 | 204,33 | 252,44 | 0,26 | 7,54 | 0,0001 | 0,0033 |
| Cox5b | 84,39 | 118,51 | 103,71 | 78,42 | 117,20 | 110,68 | 116,26 | 121,03 | 0,28 | 5,92 | 0,0001 | 0,0033 |
| Atad5 | 2,62 | 3,52 | 2,74 | 2,66 | 2,12 | 2,04 | 2,37 | 2,23 | -0,40 | 4,21 | 0,0001 | 0,0033 |
| Mcm10 | 1,79 | 3,67 | 2,14 | 2,23 | 1,47 | 1,60 | 1,90 | 1,83 | -0,55 | 2,88 | 0,0001 | 0,0033 |
| Paf1 | 29,87 | 31,29 | 27,07 | 28,17 | 35,84 | 33,74 | 34,10 | 36,93 | 0,27 | 5,98 | 0,0001 | 0,0033 |
| Rbm3 | 28,64 | 42,06 | 37,70 | 29,08 | 38,43 | 40,48 | 37,64 | 46,93 | 0,25 | 6,77 | 0,0001 | 0,0033 |
| Hist1h2bq | 7,76 | 11,48 | 10,97 | 7,51 | 6,67 | 7,05 | 7,51 | 7,09 | -0,40 | 4,12 | 0,0001 | 0,0033 |
| Klrc2 | 98,59 | 46,20 | 56,85 | 60,80 | 54,73 | 57,62 | 56,23 | 44,30 | -0,31 | 5,42 | 0,0001 | 0,0033 |
| Ccdc117 | 15,22 | 17,57 | 14,43 | 16,62 | 19,80 | 17,97 | 17,15 | 22,40 | 0,27 | 5,91 | 0,0001 | 0,0034 |
| Alms1 | 2,42 | 1,86 | 1,47 | 1,69 | 1,57 | 1,38 | 1,27 | 1,38 | -0,42 | 4,03 | 0,0001 | 0,0034 |
| Foxp4 | 21,20 | 26,04 | 27,75 | 22,79 | 28,54 | 28,09 | 30,60 | 28,88 | 0,25 | 6,75 | 0,0001 | 0,0035 |
| Pik3c2a | 13,52 | 9,40 | 10,58 | 12,16 | 9,85 | 9,52 | 9,61 | 9,34 | -0,25 | 6,40 | 0,0001 | 0,0035 |
| H2-Ke6 | 23,35 | 28,67 | 33,45 | 24,92 | 34,63 | 35,28 | 31,18 | 38,03 | 0,35 | 4,91 | 0,0001 | 0,0036 |
| Ahsa2 | 8,74 | 7,19 | 8,58 | 8,03 | 11,16 | 10,30 | 8,76 | 10,57 | 0,33 | 5,05 | 0,0001 | 0,0036 |
| Birc5 | 5,26 | 9,34 | 5,98 | 5,35 | 4,22 | 4,26 | 5,82 | 5,55 | -0,39 | 4,29 | 0,0001 | 0,0036 |
| Ubald1 | 49,77 | 57,21 | 59,70 | 50,02 | 67,30 | 63,65 | 61,75 | 65,39 | 0,26 | 6,36 | 0,0001 | 0,0037 |
| Uqcrb | 41,25 | 61,87 | 58,47 | 37,92 | 66,20 | 60,70 | 65,26 | 60,29 | 0,35 | 4,84 | 0,0001 | 0,0038 |
| Mat2a | 49,83 | 47,92 | 47,66 | 50,05 | 60,52 | 56,38 | 52,12 | 63,15 | 0,25 | 7,23 | 0,0001 | 0,0038 |
| Pou5f2 | 5,75 | 1,76 | 1,56 | 1,69 | 1,30 | 1,52 | 2,05 | 1,81 | -0,72 | 1,65 | 0,0001 | 0,0038 |
| H2afx | 46,90 | 73,74 | 75,22 | 51,09 | 46,89 | 52,38 | 58,56 | 48,31 | -0,25 | 6,26 | 0,0001 | 0,0039 |
| Treml4 | 2,27 | 1,83 | 1,57 | 1,80 | 1,45 | 2,05 | 0,70 | 0,26 | -0,76 | 1,42 | 0,0001 | 0,0039 |
| Rad51ap1 | 2,39 | 3,42 | 2,22 | 2,94 | 1,34 | 1,81 | 1,95 | 2,00 | -0,65 | 2,08 | 0,0001 | 0,0039 |
| Cbx5 | 19,70 | 20,59 | 18,76 | 21,47 | 16,48 | 16,33 | 17,98 | 17,13 | -0,25 | 7,37 | 0,0001 | 0,0042 |
| Atp8b4 | 98,53 | 80,92 | 84,00 | 89,71 | 73,89 | 74,53 | 76,38 | 70,64 | -0,26 | 8,81 | 0,0001 | 0,0042 |
| Foxm1 | 8,20 | 12,35 | 9,90 | 9,09 | 7,47 | 7,39 | 9,45 | 7,67 | -0,31 | 5,29 | 0,0002 | 0,0043 |
| Phf1 | 35,42 | 34,76 | 42,32 | 33,25 | 45,10 | 46,92 | 39,36 | 41,14 | 0,25 | 6,60 | 0,0002 | 0,0045 |
| Tppp3 | 2,92 | 7,04 | 6,74 | 4,88 | 1,60 | 3,94 | 5,32 | 3,09 | -0,62 | 2,18 | 0,0002 | 0,0046 |
| Mafk | 35,34 | 31,45 | 27,15 | 37,07 | 37,33 | 37,99 | 36,69 | 43,62 | 0,24 | 6,67 | 0,0002 | 0,0046 |
| Hist2h2aa1 | 134,33 | 220,95 | 205,17 | 140,31 | 141,73 | 139,29 | 160,79 | 147,82 | -0,24 | 6,54 | 0,0002 | 0,0046 |
| Kctd12 | 2,09 | 1,38 | 1,30 | 1,53 | 1,16 | 1,54 | 1,03 | 0,72 | -0,51 | 3,04 | 0,0002 | 0,0047 |
| Ltb | 196,65 | 195,27 | 213,24 | 202,98 | 243,49 | 242,65 | 234,89 | 239,78 | 0,25 | 7,92 | 0,0002 | 0,0048 |
| Mospd3 | 22,09 | 26,19 | 22,34 | 19,96 | 29,35 | 27,33 | 28,22 | 27,28 | 0,31 | 5,23 | 0,0002 | 0,0048 |
| Nr1h3 | 2,13 | 1,48 | 1,89 | 1,88 | 1,17 | 1,65 | 1,09 | 0,61 | -0,70 | 1,56 | 0,0002 | 0,0048 |
| Bak1 | 42,68 | 59,41 | 48,80 | 43,40 | 58,03 | 55,09 | 58,65 | 58,32 | 0,24 | 6,73 | 0,0002 | 0,0048 |
| Prdm1 | 13,89 | 13,09 | 13,15 | 14,49 | 12,14 | 11,47 | 12,70 | 9,30 | -0,26 | 6,01 | 0,0002 | 0,0048 |
| Apobr | 5,68 | 6,66 | 7,28 | 5,71 | 7,58 | 8,45 | 8,31 | 7,81 | 0,35 | 4,70 | 0,0002 | 0,0049 |
| Hist1h2br | 11,98 | 17,98 | 20,38 | 12,55 | 12,46 | 12,90 | 12,59 | 11,69 | -0,32 | 4,89 | 0,0002 | 0,0050 |
| Abracl | 94,53 | 157,91 | 150,26 | 85,41 | 136,24 | 146,85 | 140,24 | 152,82 | 0,25 | 6,75 | 0,0002 | 0,0050 |
| Lpp | 7,69 | 5,45 | 6,13 | 6,99 | 5,78 | 5,71 | 5,54 | 5,17 | -0,24 | 6,57 | 0,0002 | 0,0051 |
| Kat8 | 9,18 | 10,11 | 10,76 | 9,53 | 11,16 | 13,81 | 13,02 | 13,99 | 0,40 | 4,12 | 0,0002 | 0,0052 |
| Upf3a | 5,84 | 10,14 | 10,13 | 6,23 | 9,24 | 10,46 | 10,63 | 13,03 | 0,44 | 3,74 | 0,0002 | 0,0054 |
| Nbas | 7,68 | 6,25 | 6,94 | 7,35 | 5,79 | 5,68 | 6,10 | 5,58 | -0,29 | 5,54 | 0,0002 | 0,0054 |
| Trp53inp2 | 9,91 | 9,31 | 6,78 | 9,56 | 11,42 | 10,17 | 11,07 | 11,31 | 0,29 | 5,30 | 0,0002 | 0,0055 |
| Ttyh3 | 26,86 | 29,92 | 33,68 | 28,79 | 36,52 | 36,56 | 31,81 | 35,88 | 0,24 | 7,24 | 0,0002 | 0,0055 |
| Timeless | 2,88 | 4,68 | 3,41 | 3,67 | 2,49 | 2,80 | 3,03 | 2,67 | -0,42 | 3,84 | 0,0002 | 0,0056 |
| H2-Q4 | 301,45 | 293,00 | 320,58 | 269,15 | 360,56 | 358,64 | 319,41 | 371,20 | 0,25 | 9,18 | 0,0002 | 0,0056 |
| Blvrb | 3,46 | 3,85 | 2,84 | 3,12 | 2,04 | 2,62 | 1,42 | 1,89 | -0,76 | 1,24 | 0,0002 | 0,0057 |
| Grina | 49,46 | 55,37 | 54,97 | 45,27 | 64,04 | 60,97 | 56,91 | 60,23 | 0,24 | 6,57 | 0,0002 | 0,0058 |
| Dynll1 | 40,19 | 38,53 | 42,96 | 34,86 | 47,56 | 47,71 | 43,36 | 46,34 | 0,25 | 6,43 | 0,0002 | 0,0059 |
| Snrpd2 | 36,49 | 48,30 | 40,89 | 33,26 | 46,73 | 55,44 | 49,50 | 52,41 | 0,36 | 4,48 | 0,0002 | 0,0059 |
| Cdc6 | 1,73 | 2,57 | 1,38 | 1,84 | 1,11 | 1,06 | 1,83 | 1,31 | -0,52 | 2,91 | 0,0002 | 0,0059 |
| Hist2h2aa2 | 122,60 | 199,37 | 180,53 | 118,45 | 122,81 | 130,04 | 145,78 | 125,90 | -0,24 | 6,56 | 0,0002 | 0,0059 |
| Depdc1b | 8,77 | 9,67 | 9,46 | 9,32 | 6,26 | 6,83 | 8,61 | 7,16 | -0,37 | 4,38 | 0,0002 | 0,0059 |
| Pom121 | 15,33 | 18,63 | 17,20 | 16,59 | 21,19 | 18,77 | 19,87 | 20,10 | 0,24 | 6,69 | 0,0002 | 0,0063 |
| Mga | 17,51 | 11,85 | 12,35 | 16,21 | 13,32 | 11,50 | 12,66 | 11,59 | -0,24 | 7,54 | 0,0002 | 0,0063 |
| Dtl | 4,53 | 4,84 | 3,38 | 3,90 | 2,94 | 3,50 | 3,18 | 3,05 | -0,41 | 3,95 | 0,0002 | 0,0064 |
| Rps19bp1 | 3,93 | 3,90 | 4,33 | 3,96 | 5,60 | 6,92 | 5,96 | 6,20 | 0,62 | 2,13 | 0,0003 | 0,0066 |
| Ncaph | 7,22 | 11,93 | 10,22 | 9,21 | 6,92 | 6,47 | 9,25 | 7,57 | -0,35 | 4,53 | 0,0003 | 0,0066 |
| Dennd6a | 10,63 | 9,41 | 10,40 | 10,69 | 8,30 | 8,74 | 8,93 | 8,52 | -0,25 | 5,99 | 0,0003 | 0,0071 |
| Egr1 | 10,88 | 12,19 | 10,90 | 11,25 | 13,88 | 12,78 | 14,60 | 14,25 | 0,29 | 5,28 | 0,0003 | 0,0071 |
| Myo5a | 3,76 | 3,61 | 3,40 | 3,39 | 2,97 | 2,80 | 3,04 | 2,71 | -0,30 | 5,23 | 0,0003 | 0,0072 |
| AU041133 | 4,26 | 2,40 | 3,02 | 3,37 | 2,16 | 2,31 | 2,59 | 2,12 | -0,51 | 2,88 | 0,0003 | 0,0073 |
| Amigo1 | 1,88 | 1,73 | 2,08 | 1,90 | 3,02 | 2,32 | 2,28 | 2,56 | 0,43 | 3,65 | 0,0003 | 0,0073 |
| Cdca3 | 8,52 | 15,25 | 11,25 | 10,45 | 7,23 | 7,85 | 9,12 | 10,24 | -0,40 | 3,90 | 0,0003 | 0,0074 |
| Melk | 1,91 | 2,91 | 2,43 | 2,09 | 1,45 | 1,80 | 1,73 | 1,36 | -0,56 | 2,52 | 0,0003 | 0,0074 |
| Rrbp1 | 9,18 | 12,52 | 11,54 | 8,59 | 12,86 | 11,71 | 12,08 | 13,29 | 0,26 | 5,90 | 0,0003 | 0,0075 |
| Spc25 | 4,70 | 7,47 | 5,86 | 5,11 | 3,43 | 3,92 | 4,74 | 4,35 | -0,50 | 2,97 | 0,0003 | 0,0075 |
| Arrdc3 | 12,88 | 11,49 | 12,37 | 14,13 | 11,54 | 10,64 | 10,50 | 9,35 | -0,28 | 5,57 | 0,0003 | 0,0075 |
| Tmem245 | 12,43 | 8,30 | 9,19 | 10,42 | 9,03 | 8,25 | 8,53 | 8,12 | -0,25 | 6,00 | 0,0003 | 0,0076 |
| Cdkn3 | 6,09 | 8,62 | 7,59 | 6,47 | 4,40 | 4,43 | 5,76 | 4,48 | -0,59 | 2,22 | 0,0003 | 0,0076 |
| Plk3 | 9,49 | 8,78 | 6,63 | 7,69 | 10,63 | 10,30 | 11,01 | 9,82 | 0,35 | 4,48 | 0,0003 | 0,0077 |
| Ubl5 | 24,49 | 28,13 | 23,80 | 19,85 | 30,40 | 27,46 | 30,42 | 27,63 | 0,27 | 5,69 | 0,0003 | 0,0080 |
| Brip1 | 3,16 | 3,27 | 2,41 | 2,98 | 2,06 | 2,19 | 2,57 | 2,35 | -0,38 | 4,20 | 0,0003 | 0,0080 |
| Zfp518a | 14,77 | 11,71 | 12,83 | 14,62 | 11,02 | 11,61 | 12,58 | 10,59 | -0,24 | 6,41 | 0,0003 | 0,0080 |
| Rps18 | 400,84 | 501,66 | 574,28 | 397,97 | 537,56 | 585,91 | 491,36 | 596,70 | 0,24 | 8,06 | 0,0003 | 0,0082 |
| Aurkb | 9,81 | 12,52 | 9,62 | 9,62 | 6,93 | 7,52 | 9,00 | 8,72 | -0,38 | 4,18 | 0,0003 | 0,0082 |
| Rps6 | 485,89 | 566,53 | 478,14 | 432,05 | 599,35 | 554,97 | 559,22 | 609,75 | 0,24 | 9,55 | 0,0003 | 0,0084 |
| Rnasek | 52,78 | 71,23 | 69,94 | 47,54 | 75,81 | 70,55 | 74,89 | 71,82 | 0,29 | 5,38 | 0,0004 | 0,0086 |
| Shcbp1 | 3,64 | 5,10 | 3,86 | 3,93 | 1,92 | 3,05 | 3,88 | 2,92 | -0,50 | 2,92 | 0,0004 | 0,0086 |
| Chaf1a | 4,53 | 6,89 | 5,15 | 4,88 | 4,04 | 4,16 | 4,53 | 3,66 | -0,39 | 3,97 | 0,0004 | 0,0089 |
| Tgfb1 | 51,68 | 68,99 | 68,47 | 52,85 | 72,12 | 68,64 | 67,95 | 74,89 | 0,23 | 7,11 | 0,0004 | 0,0089 |
| Pola1 | 11,59 | 12,66 | 11,99 | 10,21 | 9,62 | 9,50 | 10,49 | 9,23 | -0,26 | 5,83 | 0,0004 | 0,0090 |
| Ccl6 | 2,35 | 1,71 | 1,78 | 2,29 | 1,50 | 1,44 | 1,17 | 0,86 | -0,71 | 1,28 | 0,0004 | 0,0090 |
| Tanc2 | 2,50 | 1,94 | 1,86 | 2,30 | 1,72 | 1,83 | 2,02 | 1,22 | -0,35 | 4,52 | 0,0004 | 0,0090 |
| Ddx60 | 4,35 | 3,33 | 3,28 | 3,67 | 3,03 | 3,16 | 2,66 | 2,56 | -0,36 | 4,29 | 0,0004 | 0,0092 |
| Tuba1c | 47,44 | 60,68 | 44,06 | 56,30 | 43,83 | 41,28 | 46,76 | 46,19 | -0,23 | 6,58 | 0,0004 | 0,0096 |
| Kif18a | 3,79 | 4,58 | 3,59 | 4,13 | 2,78 | 3,23 | 2,86 | 3,19 | -0,43 | 3,58 | 0,0004 | 0,0096 |
| S100a13 | 25,65 | 39,62 | 30,29 | 22,01 | 36,35 | 37,13 | 37,02 | 38,06 | 0,34 | 4,58 | 0,0004 | 0,0097 |
| Nbeal1 | 4,78 | 3,20 | 3,30 | 3,96 | 3,12 | 3,01 | 3,41 | 3,04 | -0,28 | 5,40 | 0,0004 | 0,0097 |
| Tgif2 | 2,01 | 1,84 | 1,56 | 1,72 | 2,14 | 2,58 | 2,47 | 3,17 | 0,52 | 2,65 | 0,0004 | 0,0097 |
| Thada | 20,38 | 13,67 | 16,40 | 18,09 | 15,10 | 14,71 | 15,32 | 13,39 | -0,23 | 6,97 | 0,0004 | 0,0098 |
| Acp2 | 5,30 | 5,17 | 4,21 | 4,54 | 3,69 | 3,95 | 4,19 | 3,23 | -0,36 | 4,32 | 0,0004 | 0,0098 |
| Txk | 30,15 | 29,54 | 26,12 | 27,91 | 23,17 | 23,22 | 26,24 | 23,03 | -0,25 | 5,89 | 0,0004 | 0,0099 |
| Pim2 | 36,63 | 38,47 | 36,19 | 42,57 | 46,17 | 45,39 | 43,76 | 45,57 | 0,23 | 6,42 | 0,0004 | 0,0100 |
| Esco2 | 3,17 | 5,58 | 3,62 | 3,20 | 2,42 | 2,64 | 3,38 | 3,00 | -0,45 | 3,30 | 0,0004 | 0,0100 |
| Cnot3 | 41,05 | 45,55 | 44,96 | 36,54 | 51,07 | 48,30 | 48,71 | 48,59 | 0,23 | 7,05 | 0,0004 | 0,0102 |
| Rgs1 | 47,44 | 44,00 | 36,83 | 39,30 | 49,40 | 45,87 | 45,16 | 59,00 | 0,24 | 5,94 | 0,0004 | 0,0102 |
| Epg5 | 12,39 | 8,85 | 9,66 | 11,04 | 9,31 | 8,95 | 8,94 | 8,60 | -0,23 | 6,56 | 0,0004 | 0,0102 |
| Asf1b | 9,76 | 14,62 | 9,23 | 9,41 | 8,26 | 7,25 | 9,06 | 8,48 | -0,39 | 3,94 | 0,0004 | 0,0102 |
| Pygo2 | 19,58 | 21,11 | 22,71 | 20,10 | 23,87 | 24,13 | 21,93 | 28,72 | 0,24 | 6,05 | 0,0005 | 0,0104 |
| Ncapg | 7,66 | 10,14 | 9,51 | 8,98 | 6,42 | 7,42 | 8,36 | 7,08 | -0,31 | 4,92 | 0,0005 | 0,0104 |
| Ndufa6 | 46,88 | 63,45 | 68,23 | 44,48 | 65,69 | 72,42 | 59,27 | 75,09 | 0,30 | 5,12 | 0,0005 | 0,0104 |
| Fam64a | 2,81 | 4,01 | 3,29 | 3,75 | 1,53 | 1,96 | 2,97 | 2,80 | -0,59 | 2,10 | 0,0005 | 0,0104 |
| Rpl31-ps12 | 34,36 | 46,48 | 47,48 | 32,72 | 47,56 | 50,69 | 48,68 | 56,53 | 0,35 | 4,47 | 0,0005 | 0,0107 |
| Msl1 | 37,75 | 40,17 | 41,58 | 38,03 | 46,61 | 45,64 | 41,55 | 50,75 | 0,23 | 7,54 | 0,0005 | 0,0108 |
| Sepsecs | 7,47 | 5,40 | 7,07 | 7,31 | 5,57 | 4,76 | 5,24 | 4,73 | -0,42 | 3,52 | 0,0005 | 0,0109 |
| Utp3 | 12,19 | 19,44 | 17,96 | 14,09 | 17,95 | 18,41 | 19,34 | 23,30 | 0,32 | 4,84 | 0,0005 | 0,0110 |
| Lrba | 36,50 | 26,88 | 26,76 | 30,43 | 26,11 | 24,45 | 26,92 | 25,16 | -0,23 | 8,11 | 0,0005 | 0,0111 |
| Cd69 | 23,80 | 27,50 | 22,94 | 24,94 | 28,26 | 24,38 | 33,13 | 34,11 | 0,27 | 5,48 | 0,0005 | 0,0111 |
| Magt1 | 25,13 | 22,88 | 22,50 | 26,02 | 20,61 | 20,47 | 20,62 | 20,88 | -0,23 | 6,66 | 0,0005 | 0,0112 |
| Tecr | 48,96 | 63,87 | 61,22 | 44,27 | 65,75 | 62,70 | 66,21 | 62,33 | 0,24 | 6,13 | 0,0005 | 0,0112 |
| Nf1 | 1,77 | 1,80 | 1,70 | 1,71 | 1,38 | 1,21 | 1,42 | 1,44 | -0,36 | 4,20 | 0,0005 | 0,0113 |
| Dennd4a | 39,24 | 29,81 | 29,87 | 36,78 | 29,30 | 28,41 | 32,50 | 25,37 | -0,23 | 8,09 | 0,0005 | 0,0114 |
| Eif3g | 46,96 | 60,48 | 56,32 | 45,64 | 59,18 | 64,01 | 60,79 | 64,02 | 0,25 | 5,94 | 0,0005 | 0,0114 |
| Ralgapa2 | 27,08 | 20,03 | 19,83 | 23,73 | 19,88 | 18,72 | 20,57 | 18,26 | -0,23 | 7,66 | 0,0005 | 0,0114 |
| Cks1b | 14,20 | 21,69 | 15,74 | 14,77 | 10,97 | 10,12 | 15,05 | 13,40 | -0,43 | 3,46 | 0,0005 | 0,0114 |
| Ttk | 2,28 | 3,71 | 2,67 | 3,16 | 2,04 | 1,77 | 2,36 | 2,30 | -0,49 | 2,90 | 0,0005 | 0,0114 |
| Hist1h4b | 80,38 | 147,18 | 134,28 | 87,85 | 83,84 | 85,74 | 93,71 | 100,37 | -0,30 | 4,98 | 0,0005 | 0,0114 |
| Cd72 | 6,33 | 7,04 | 5,54 | 6,69 | 8,76 | 9,02 | 7,70 | 8,95 | 0,41 | 3,53 | 0,0005 | 0,0114 |
| Plxnc1 | 26,80 | 22,61 | 22,86 | 23,24 | 20,61 | 21,55 | 21,00 | 18,61 | -0,22 | 7,29 | 0,0005 | 0,0116 |
| Klrc1 | 261,27 | 165,38 | 208,22 | 230,11 | 186,94 | 194,30 | 190,83 | 163,11 | -0,23 | 8,63 | 0,0005 | 0,0120 |
| Zfp831 | 29,78 | 22,99 | 23,99 | 27,09 | 24,29 | 22,54 | 21,84 | 19,98 | -0,23 | 7,88 | 0,0006 | 0,0125 |
| Ctla2a | 121,90 | 131,35 | 140,51 | 116,32 | 145,81 | 145,56 | 143,48 | 161,36 | 0,23 | 7,57 | 0,0006 | 0,0125 |
| Use1 | 31,87 | 40,14 | 47,51 | 28,54 | 43,39 | 47,45 | 39,57 | 47,20 | 0,28 | 5,44 | 0,0006 | 0,0125 |
| Cenpi | 1,56 | 2,26 | 1,80 | 1,81 | 0,98 | 1,12 | 1,72 | 1,26 | -0,55 | 2,31 | 0,0006 | 0,0126 |
| Hsp90ab1 | 213,31 | 258,93 | 234,29 | 228,68 | 268,34 | 268,68 | 259,82 | 302,82 | 0,23 | 9,32 | 0,0006 | 0,0126 |
| Gtf2ird2 | 4,15 | 3,53 | 4,04 | 3,68 | 5,64 | 4,72 | 4,12 | 5,49 | 0,38 | 3,95 | 0,0006 | 0,0126 |
| Zfp524 | 11,90 | 13,30 | 13,69 | 11,80 | 15,46 | 15,59 | 16,80 | 17,68 | 0,38 | 4,02 | 0,0006 | 0,0127 |
| Ube3c | 18,66 | 17,34 | 18,49 | 19,33 | 15,59 | 15,09 | 16,71 | 15,73 | -0,23 | 6,43 | 0,0006 | 0,0129 |
| Herc1 | 38,48 | 25,90 | 26,53 | 33,17 | 28,38 | 23,24 | 27,46 | 26,54 | -0,23 | 8,77 | 0,0006 | 0,0130 |
| Fgl2 | 22,13 | 19,23 | 21,88 | 22,77 | 18,55 | 18,15 | 18,91 | 17,64 | -0,23 | 6,23 | 0,0006 | 0,0130 |
| Sapcd2 | 1,03 | 1,57 | 1,45 | 1,10 | 0,72 | 0,73 | 1,03 | 0,86 | -0,61 | 1,74 | 0,0006 | 0,0130 |
| Trmt112 | 28,45 | 31,91 | 32,90 | 24,47 | 34,73 | 36,95 | 38,63 | 33,32 | 0,29 | 5,08 | 0,0006 | 0,0131 |
| Ddx23 | 25,63 | 28,32 | 30,31 | 26,68 | 30,67 | 34,75 | 30,78 | 33,40 | 0,23 | 6,40 | 0,0006 | 0,0133 |
| Zdhhc21 | 14,14 | 12,34 | 12,76 | 13,97 | 11,26 | 11,36 | 11,57 | 11,46 | -0,22 | 6,76 | 0,0006 | 0,0133 |
| Rpl14 | 189,01 | 238,74 | 226,46 | 180,45 | 240,66 | 246,67 | 221,86 | 266,36 | 0,23 | 7,73 | 0,0006 | 0,0137 |
| Cd7 | 73,63 | 81,99 | 81,84 | 83,55 | 97,14 | 88,12 | 99,53 | 89,81 | 0,22 | 6,50 | 0,0006 | 0,0137 |
| Rhog | 85,33 | 113,83 | 117,39 | 89,30 | 117,23 | 116,98 | 120,22 | 117,89 | 0,22 | 7,12 | 0,0006 | 0,0137 |
| Slc35a3 | 17,17 | 16,02 | 16,23 | 20,15 | 14,03 | 15,38 | 14,79 | 14,96 | -0,24 | 6,02 | 0,0007 | 0,0141 |
| Ccl3 | 19,22 | 17,25 | 17,84 | 12,66 | 25,05 | 22,00 | 21,44 | 18,16 | 0,38 | 3,94 | 0,0007 | 0,0141 |
| Dcaf17 | 8,33 | 6,21 | 6,12 | 7,27 | 5,73 | 5,79 | 6,18 | 5,75 | -0,26 | 5,64 | 0,0007 | 0,0141 |
| Ndnl2 | 9,11 | 10,89 | 9,65 | 9,02 | 12,37 | 11,95 | 11,91 | 13,87 | 0,37 | 3,97 | 0,0007 | 0,0144 |
| Midn | 34,51 | 45,13 | 45,17 | 37,90 | 45,93 | 47,57 | 46,42 | 49,51 | 0,22 | 7,35 | 0,0007 | 0,0144 |
| Hsd17b11 | 18,69 | 19,87 | 20,21 | 20,06 | 26,26 | 22,77 | 22,07 | 24,46 | 0,28 | 5,22 | 0,0007 | 0,0146 |
| Vps37b | 35,87 | 37,30 | 36,29 | 38,67 | 41,23 | 43,90 | 40,72 | 46,83 | 0,22 | 6,66 | 0,0007 | 0,0146 |
| Sgol1 | 2,39 | 3,53 | 2,46 | 3,00 | 1,86 | 1,73 | 2,38 | 2,45 | -0,45 | 3,17 | 0,0007 | 0,0148 |
| Lxn | 15,23 | 13,15 | 17,93 | 13,29 | 18,95 | 18,82 | 18,13 | 19,71 | 0,36 | 4,21 | 0,0007 | 0,0148 |
| Eme1 | 2,57 | 3,83 | 2,97 | 3,08 | 2,10 | 1,94 | 2,18 | 2,51 | -0,52 | 2,57 | 0,0007 | 0,0148 |
| Cdc45 | 3,67 | 6,01 | 3,80 | 4,00 | 2,69 | 2,89 | 4,32 | 2,89 | -0,46 | 3,04 | 0,0007 | 0,0148 |
| Gpsm3 | 119,41 | 132,24 | 126,54 | 118,66 | 150,44 | 142,51 | 140,87 | 145,18 | 0,22 | 7,50 | 0,0007 | 0,0150 |
| Pnrc1 | 148,08 | 155,40 | 149,64 | 145,06 | 181,20 | 170,77 | 167,74 | 180,39 | 0,23 | 8,29 | 0,0007 | 0,0150 |
| Ier5 | 37,27 | 37,98 | 43,32 | 36,83 | 44,26 | 47,11 | 40,69 | 48,55 | 0,22 | 7,10 | 0,0007 | 0,0152 |
| Zwilch | 1,94 | 2,92 | 1,70 | 2,34 | 1,31 | 1,57 | 1,83 | 1,53 | -0,54 | 2,43 | 0,0008 | 0,0160 |
| Cd27 | 114,78 | 124,20 | 134,71 | 111,79 | 144,03 | 140,59 | 137,90 | 143,18 | 0,22 | 7,69 | 0,0008 | 0,0161 |
| Snrnp70 | 87,11 | 92,39 | 94,22 | 76,77 | 102,74 | 101,87 | 97,51 | 105,16 | 0,22 | 7,28 | 0,0008 | 0,0162 |
| Stmn1 | 40,41 | 61,72 | 40,42 | 38,95 | 34,10 | 35,36 | 41,68 | 40,79 | -0,26 | 5,47 | 0,0008 | 0,0167 |
| Emg1 | 58,84 | 57,12 | 48,33 | 56,07 | 66,74 | 64,41 | 62,00 | 66,52 | 0,23 | 5,98 | 0,0008 | 0,0170 |
| Cpt1a | 20,66 | 28,39 | 19,97 | 33,18 | 21,75 | 18,82 | 24,61 | 23,04 | -0,22 | 6,68 | 0,0009 | 0,0174 |
| Mdm1 | 4,63 | 5,32 | 6,21 | 4,57 | 3,67 | 4,05 | 3,95 | 4,24 | -0,37 | 3,84 | 0,0009 | 0,0174 |
| Rpl9 | 445,55 | 510,07 | 546,03 | 426,35 | 562,47 | 582,04 | 517,64 | 590,88 | 0,23 | 8,57 | 0,0009 | 0,0178 |
| Wdr92 | 13,78 | 15,64 | 16,40 | 14,22 | 16,85 | 17,33 | 17,16 | 19,51 | 0,24 | 5,81 | 0,0009 | 0,0179 |
| Ubxn1 | 50,62 | 70,45 | 56,49 | 55,20 | 69,81 | 64,96 | 66,28 | 71,98 | 0,23 | 6,03 | 0,0009 | 0,0186 |
| Slc8b1 | 11,01 | 10,13 | 9,67 | 11,24 | 13,17 | 13,16 | 11,30 | 13,56 | 0,28 | 5,08 | 0,0009 | 0,0187 |
| Gnrh1 | 2,56 | 3,00 | 1,27 | 2,16 | 4,62 | 4,34 | 3,48 | 3,13 | 0,73 | 0,83 | 0,0009 | 0,0187 |
| B3gntl1 | 9,27 | 7,00 | 6,63 | 7,83 | 6,10 | 6,27 | 6,26 | 5,80 | -0,34 | 4,30 | 0,0009 | 0,0187 |
| Selplg | 370,18 | 430,71 | 380,46 | 366,09 | 470,26 | 443,20 | 467,56 | 427,84 | 0,23 | 10,09 | 0,0009 | 0,0188 |
| Rps25 | 198,17 | 231,99 | 240,71 | 165,74 | 232,99 | 248,95 | 225,81 | 261,33 | 0,22 | 7,27 | 0,0010 | 0,0195 |
| Mex3a | 1,00 | 1,04 | 1,02 | 0,89 | 1,27 | 1,66 | 1,03 | 1,54 | 0,48 | 2,80 | 0,0010 | 0,0196 |
| Rpl15 | 195,62 | 246,66 | 251,66 | 178,50 | 255,58 | 248,72 | 249,97 | 263,79 | 0,22 | 8,77 | 0,0010 | 0,0199 |
| Mybl2 | 3,26 | 4,73 | 3,91 | 3,07 | 2,50 | 2,40 | 3,36 | 3,17 | -0,39 | 3,61 | 0,0010 | 0,0201 |
| Dusp5 | 98,15 | 92,92 | 80,32 | 93,99 | 106,87 | 96,62 | 109,24 | 111,15 | 0,21 | 6,84 | 0,0010 | 0,0202 |
| Atp5j2 | 25,11 | 41,40 | 40,25 | 22,92 | 37,52 | 40,77 | 36,26 | 49,47 | 0,35 | 4,14 | 0,0010 | 0,0202 |
| Tob2 | 26,76 | 29,82 | 27,18 | 27,36 | 33,58 | 31,19 | 30,33 | 33,64 | 0,21 | 6,91 | 0,0010 | 0,0204 |
| Dnajb9 | 32,63 | 26,86 | 22,67 | 30,45 | 33,40 | 31,82 | 33,91 | 33,85 | 0,23 | 5,85 | 0,0011 | 0,0207 |
| Rgs19 | 43,53 | 47,93 | 44,33 | 42,30 | 52,98 | 50,62 | 51,26 | 53,07 | 0,22 | 6,14 | 0,0011 | 0,0207 |
| Cenpn | 1,91 | 3,33 | 3,64 | 2,36 | 1,36 | 1,65 | 2,54 | 1,99 | -0,55 | 2,04 | 0,0011 | 0,0210 |
| Zrsr1 | 3,40 | 3,41 | 3,56 | 2,82 | 4,63 | 4,06 | 4,19 | 3,88 | 0,35 | 4,09 | 0,0011 | 0,0212 |
| Ndufc2 | 17,51 | 21,73 | 20,00 | 16,70 | 23,19 | 24,78 | 27,00 | 22,67 | 0,37 | 3,90 | 0,0011 | 0,0213 |
| Rbm38 | 67,61 | 80,20 | 80,92 | 69,57 | 84,69 | 88,75 | 80,54 | 91,17 | 0,21 | 7,16 | 0,0011 | 0,0213 |
| Rad51 | 2,28 | 4,47 | 3,32 | 3,05 | 1,51 | 2,78 | 2,48 | 2,57 | -0,50 | 2,59 | 0,0011 | 0,0214 |
| Il2ra | 15,43 | 12,75 | 12,77 | 13,39 | 11,98 | 11,14 | 12,22 | 10,82 | -0,24 | 5,80 | 0,0011 | 0,0217 |
| C2cd5 | 20,29 | 20,90 | 19,63 | 19,99 | 17,44 | 16,98 | 17,71 | 17,49 | -0,22 | 6,37 | 0,0011 | 0,0218 |
| Sfrs18 | 30,49 | 34,11 | 32,23 | 29,42 | 36,81 | 35,78 | 35,51 | 38,00 | 0,21 | 7,28 | 0,0011 | 0,0219 |
| Spc24 | 4,92 | 6,14 | 5,26 | 4,98 | 2,97 | 4,48 | 3,74 | 4,03 | -0,49 | 2,63 | 0,0012 | 0,0220 |
| Tnrc6b | 41,42 | 30,58 | 30,17 | 38,38 | 32,51 | 27,50 | 31,47 | 29,17 | -0,22 | 9,14 | 0,0012 | 0,0220 |
| D8Ertd738e | 45,79 | 52,42 | 48,07 | 50,37 | 62,03 | 57,54 | 56,19 | 61,45 | 0,27 | 5,17 | 0,0012 | 0,0220 |
| Pik3ap1 | 43,33 | 38,10 | 38,48 | 43,26 | 37,46 | 35,24 | 38,25 | 30,04 | -0,21 | 6,64 | 0,0012 | 0,0220 |
| Rps15a | 50,90 | 53,17 | 55,52 | 43,34 | 58,86 | 60,60 | 56,14 | 60,24 | 0,22 | 8,14 | 0,0012 | 0,0220 |
| Hist2h2ab | 21,77 | 31,67 | 30,08 | 20,84 | 18,94 | 20,00 | 19,18 | 19,13 | -0,42 | 3,15 | 0,0012 | 0,0222 |
| Pou2f2 | 20,80 | 24,46 | 24,74 | 19,75 | 27,17 | 25,31 | 24,64 | 26,96 | 0,22 | 6,33 | 0,0012 | 0,0223 |
| Nanos1 | 1,95 | 1,82 | 2,46 | 1,69 | 2,40 | 2,58 | 2,51 | 3,06 | 0,44 | 3,19 | 0,0012 | 0,0227 |
| Atxn2l | 105,72 | 116,08 | 118,61 | 103,13 | 132,06 | 130,01 | 128,88 | 125,52 | 0,22 | 8,80 | 0,0012 | 0,0227 |
| Zfp664 | 16,27 | 17,45 | 18,95 | 18,00 | 20,36 | 20,21 | 20,14 | 21,33 | 0,22 | 6,29 | 0,0012 | 0,0229 |
| Frat2 | 13,09 | 13,79 | 14,47 | 12,96 | 16,60 | 15,91 | 15,26 | 18,00 | 0,28 | 5,02 | 0,0012 | 0,0230 |
| Serpina3f | 1,33 | 1,33 | 1,38 | 1,76 | 0,80 | 1,25 | 0,89 | 0,83 | -0,63 | 1,46 | 0,0012 | 0,0230 |
| Tcirg1 | 41,92 | 35,72 | 44,71 | 37,64 | 47,56 | 49,88 | 41,39 | 45,87 | 0,21 | 7,03 | 0,0012 | 0,0232 |
| Ascc3 | 26,32 | 20,36 | 21,49 | 23,06 | 19,95 | 18,68 | 20,55 | 19,74 | -0,21 | 7,31 | 0,0012 | 0,0232 |
| Ccdc92 | 9,05 | 8,25 | 10,10 | 8,93 | 10,93 | 9,78 | 11,69 | 12,59 | 0,32 | 4,53 | 0,0013 | 0,0233 |
| Gzma | 566,69 | 486,55 | 504,30 | 572,54 | 483,66 | 458,58 | 476,82 | 411,22 | -0,22 | 8,76 | 0,0013 | 0,0234 |
| Leng1 | 3,00 | 4,03 | 3,68 | 3,79 | 4,86 | 4,60 | 4,92 | 4,99 | 0,42 | 3,26 | 0,0013 | 0,0235 |
| Lig1 | 7,66 | 13,12 | 7,83 | 8,52 | 7,27 | 6,98 | 8,93 | 7,18 | -0,30 | 4,75 | 0,0013 | 0,0236 |
| Gna12 | 9,87 | 8,21 | 9,71 | 9,31 | 6,27 | 8,04 | 7,99 | 6,71 | -0,35 | 3,96 | 0,0013 | 0,0237 |
| Sdf2 | 22,77 | 26,78 | 20,96 | 21,81 | 29,42 | 27,77 | 26,50 | 28,37 | 0,27 | 5,03 | 0,0013 | 0,0237 |
| Fkbp4 | 34,25 | 33,81 | 32,99 | 31,90 | 40,88 | 38,89 | 35,30 | 39,31 | 0,22 | 6,29 | 0,0013 | 0,0237 |
| Usp14 | 12,78 | 11,00 | 12,07 | 13,01 | 10,74 | 10,58 | 10,07 | 9,80 | -0,25 | 5,56 | 0,0013 | 0,0238 |
| Tceb2 | 71,79 | 107,66 | 110,77 | 65,54 | 98,67 | 100,96 | 112,52 | 107,03 | 0,25 | 5,62 | 0,0013 | 0,0240 |
| Mtmr10 | 2,78 | 2,89 | 2,78 | 2,76 | 2,08 | 2,00 | 2,53 | 2,04 | -0,37 | 3,70 | 0,0013 | 0,0244 |
| Atr | 9,79 | 7,16 | 7,34 | 8,71 | 6,72 | 7,32 | 7,55 | 6,69 | -0,23 | 5,95 | 0,0014 | 0,0246 |
| Sh3bp1 | 52,15 | 63,09 | 62,91 | 51,36 | 66,08 | 65,28 | 62,65 | 70,69 | 0,21 | 6,88 | 0,0014 | 0,0247 |
| Taf1d | 23,24 | 25,12 | 25,83 | 18,04 | 26,16 | 28,26 | 26,07 | 30,40 | 0,27 | 5,12 | 0,0014 | 0,0249 |
| Rpl17 | 556,87 | 717,52 | 665,53 | 524,56 | 750,96 | 707,53 | 683,89 | 721,54 | 0,22 | 9,01 | 0,0014 | 0,0254 |
| Rmi2 | 2,17 | 2,15 | 2,20 | 2,12 | 1,42 | 1,66 | 1,69 | 1,53 | -0,46 | 2,81 | 0,0014 | 0,0254 |
| H2-Oa | 5,20 | 7,80 | 7,84 | 6,17 | 8,98 | 8,55 | 8,44 | 10,28 | 0,44 | 3,08 | 0,0014 | 0,0256 |
| Gtf2a1 | 23,14 | 21,11 | 22,63 | 24,68 | 18,83 | 19,74 | 21,75 | 19,07 | -0,21 | 6,93 | 0,0014 | 0,0257 |
| Sh2d2a | 81,42 | 81,06 | 74,95 | 79,41 | 95,76 | 90,90 | 91,49 | 90,05 | 0,22 | 8,67 | 0,0014 | 0,0259 |
| Rpl7 | 543,43 | 654,00 | 657,13 | 556,39 | 684,52 | 695,65 | 663,60 | 756,94 | 0,22 | 9,25 | 0,0014 | 0,0259 |
| Zfp740 | 24,12 | 26,71 | 24,64 | 25,23 | 27,61 | 29,15 | 27,96 | 31,60 | 0,21 | 6,67 | 0,0014 | 0,0259 |
| Zfp407 | 16,73 | 12,57 | 12,22 | 13,08 | 11,74 | 11,38 | 12,78 | 11,45 | -0,21 | 6,66 | 0,0015 | 0,0260 |
| Flt3l | 47,30 | 51,74 | 49,35 | 43,70 | 57,63 | 57,55 | 56,09 | 54,03 | 0,23 | 5,81 | 0,0015 | 0,0260 |
| Mdn1 | 8,42 | 4,92 | 5,08 | 7,09 | 5,79 | 5,01 | 5,70 | 5,66 | -0,21 | 6,74 | 0,0015 | 0,0264 |
| Safb2 | 27,52 | 29,66 | 31,74 | 25,58 | 31,78 | 32,98 | 31,68 | 35,53 | 0,21 | 6,66 | 0,0015 | 0,0265 |
| Slc25a22 | 9,65 | 9,62 | 11,02 | 9,73 | 12,84 | 11,66 | 11,86 | 12,05 | 0,28 | 4,95 | 0,0015 | 0,0266 |
| Camta2 | 18,74 | 19,52 | 20,59 | 18,41 | 23,08 | 22,65 | 21,94 | 21,43 | 0,21 | 6,60 | 0,0015 | 0,0269 |
| Dgkh | 10,96 | 6,36 | 6,53 | 6,90 | 6,66 | 6,50 | 6,66 | 5,42 | -0,29 | 4,80 | 0,0015 | 0,0269 |
| Epas1 | 3,56 | 3,63 | 3,29 | 3,06 | 3,86 | 3,88 | 5,26 | 3,87 | 0,32 | 4,36 | 0,0015 | 0,0272 |
| Exoc6 | 25,34 | 22,67 | 21,59 | 22,57 | 19,35 | 19,10 | 21,45 | 18,95 | -0,23 | 5,87 | 0,0015 | 0,0272 |
| Gimap1 | 57,13 | 75,97 | 83,45 | 61,48 | 75,16 | 76,22 | 77,83 | 90,63 | 0,21 | 6,81 | 0,0015 | 0,0272 |
| Far1 | 31,51 | 29,47 | 32,08 | 33,10 | 26,87 | 28,19 | 28,11 | 26,30 | -0,20 | 7,00 | 0,0016 | 0,0273 |
| Hint1 | 60,53 | 93,16 | 77,17 | 59,84 | 77,68 | 87,03 | 85,29 | 94,14 | 0,25 | 5,52 | 0,0016 | 0,0275 |
| Kdm6b | 15,66 | 18,09 | 16,95 | 15,87 | 18,98 | 18,34 | 18,55 | 20,83 | 0,20 | 6,90 | 0,0016 | 0,0275 |
| Zfp458 | 2,12 | 1,92 | 1,82 | 1,97 | 1,23 | 1,52 | 1,84 | 1,02 | -0,49 | 2,56 | 0,0016 | 0,0276 |
| Tfe3 | 7,96 | 9,34 | 9,16 | 8,44 | 10,60 | 10,32 | 10,44 | 10,68 | 0,27 | 5,05 | 0,0016 | 0,0278 |
| Zbtb37 | 10,30 | 6,89 | 6,77 | 8,83 | 6,59 | 7,20 | 7,41 | 5,86 | -0,29 | 4,87 | 0,0016 | 0,0278 |
| Ppp1r18 | 91,62 | 112,25 | 104,99 | 90,00 | 116,46 | 114,55 | 110,62 | 120,58 | 0,21 | 8,42 | 0,0016 | 0,0278 |
| Rpl13 | 534,86 | 668,27 | 719,73 | 551,53 | 703,05 | 746,24 | 662,47 | 757,74 | 0,21 | 9,02 | 0,0016 | 0,0279 |
| Mybl1 | 1,07 | 1,25 | 1,46 | 1,10 | 0,69 | 0,67 | 1,01 | 0,93 | -0,55 | 1,87 | 0,0016 | 0,0280 |
| Raph1 | 5,45 | 4,67 | 4,51 | 4,96 | 4,37 | 3,70 | 4,27 | 4,13 | -0,25 | 5,36 | 0,0016 | 0,0280 |
| Ahsa1 | 41,24 | 48,45 | 47,00 | 41,94 | 53,75 | 52,21 | 48,91 | 52,96 | 0,22 | 6,00 | 0,0016 | 0,0282 |
| Chordc1 | 37,05 | 36,09 | 38,51 | 39,16 | 41,95 | 43,39 | 38,50 | 50,15 | 0,21 | 6,49 | 0,0016 | 0,0282 |
| Cd84 | 29,98 | 20,56 | 21,81 | 23,27 | 22,19 | 19,96 | 21,17 | 19,39 | -0,21 | 6,30 | 0,0017 | 0,0284 |
| Slc26a11 | 14,06 | 12,34 | 13,77 | 13,86 | 16,53 | 16,00 | 14,84 | 17,00 | 0,25 | 5,32 | 0,0017 | 0,0284 |
| Higd2a | 48,59 | 62,44 | 51,99 | 46,63 | 64,85 | 57,61 | 67,47 | 61,50 | 0,26 | 5,15 | 0,0017 | 0,0286 |
| Ccr2 | 238,72 | 217,15 | 254,33 | 248,87 | 217,28 | 216,43 | 208,11 | 185,28 | -0,21 | 9,65 | 0,0017 | 0,0289 |
| Lnpep | 182,08 | 138,71 | 141,92 | 182,44 | 141,55 | 131,11 | 145,78 | 138,11 | -0,21 | 9,60 | 0,0017 | 0,0289 |
| Iws1 | 14,20 | 11,06 | 11,38 | 12,57 | 11,21 | 10,44 | 10,80 | 10,31 | -0,20 | 6,79 | 0,0017 | 0,0289 |
| Braf | 11,36 | 8,23 | 8,77 | 9,93 | 8,65 | 7,87 | 8,64 | 8,06 | -0,21 | 6,44 | 0,0017 | 0,0290 |
| Zfp41 | 3,34 | 3,16 | 3,16 | 3,92 | 2,92 | 2,46 | 2,51 | 2,54 | -0,38 | 3,50 | 0,0017 | 0,0291 |
| Cdk2ap2 | 77,24 | 85,64 | 80,97 | 72,55 | 92,09 | 92,91 | 92,25 | 88,63 | 0,21 | 6,28 | 0,0017 | 0,0291 |
| Dnttip2 | 21,10 | 26,23 | 24,05 | 21,51 | 27,81 | 25,37 | 28,02 | 27,07 | 0,22 | 5,92 | 0,0017 | 0,0291 |
| Rps14 | 741,35 | 746,18 | 821,98 | 717,10 | 904,74 | 884,47 | 788,48 | 927,31 | 0,21 | 8,93 | 0,0018 | 0,0303 |
| Ralgapa1 | 19,05 | 14,40 | 15,62 | 16,66 | 14,90 | 13,87 | 14,82 | 13,58 | -0,20 | 7,01 | 0,0018 | 0,0306 |
| Mcm7 | 26,38 | 36,08 | 29,13 | 29,63 | 25,75 | 25,46 | 26,79 | 26,59 | -0,22 | 6,08 | 0,0018 | 0,0306 |
| Klrc3 | 24,17 | 12,14 | 17,32 | 18,48 | 14,92 | 14,52 | 13,63 | 12,03 | -0,39 | 3,41 | 0,0018 | 0,0306 |
| Mfng | 52,42 | 49,46 | 51,41 | 50,64 | 56,72 | 60,60 | 57,31 | 60,07 | 0,20 | 6,65 | 0,0018 | 0,0307 |
| Trdmt1 | 3,26 | 3,20 | 2,65 | 2,75 | 2,15 | 2,14 | 2,43 | 1,99 | -0,45 | 2,78 | 0,0019 | 0,0311 |
| Trim39 | 17,56 | 17,25 | 17,53 | 16,88 | 20,79 | 19,11 | 19,94 | 20,94 | 0,22 | 5,86 | 0,0019 | 0,0312 |
| Cacybp | 18,61 | 20,09 | 23,92 | 17,41 | 24,21 | 23,49 | 20,26 | 26,20 | 0,25 | 5,53 | 0,0019 | 0,0312 |
| Slfn5 | 12,40 | 10,06 | 10,80 | 11,28 | 10,29 | 10,00 | 9,24 | 8,76 | -0,22 | 5,96 | 0,0019 | 0,0313 |
| Rpl18a | 495,50 | 585,95 | 602,87 | 497,74 | 628,17 | 623,17 | 618,50 | 655,49 | 0,21 | 9,57 | 0,0019 | 0,0316 |
| Runx3 | 79,73 | 82,76 | 87,23 | 71,85 | 94,39 | 90,26 | 90,81 | 96,20 | 0,21 | 8,40 | 0,0019 | 0,0317 |
| Hist3h2bb-ps | 14,03 | 18,44 | 18,91 | 17,19 | 10,73 | 12,89 | 13,67 | 12,87 | -0,44 | 2,79 | 0,0019 | 0,0317 |
| Kif1c | 14,25 | 16,50 | 17,21 | 16,16 | 18,08 | 18,79 | 16,82 | 19,95 | 0,20 | 6,87 | 0,0019 | 0,0318 |
| Tnip2 | 3,78 | 4,41 | 4,52 | 3,59 | 5,25 | 5,18 | 4,97 | 6,09 | 0,41 | 3,24 | 0,0019 | 0,0318 |
| Prickle3 | 7,90 | 8,52 | 8,24 | 7,99 | 11,19 | 9,48 | 10,11 | 9,52 | 0,31 | 4,45 | 0,0020 | 0,0322 |
| Slc17a9 | 23,02 | 20,21 | 23,50 | 23,34 | 27,12 | 25,84 | 24,59 | 27,83 | 0,23 | 5,75 | 0,0020 | 0,0322 |
| Nup205 | 12,21 | 13,05 | 10,90 | 12,39 | 9,73 | 10,60 | 11,04 | 10,67 | -0,21 | 6,14 | 0,0020 | 0,0324 |
| Il18r1 | 59,71 | 56,60 | 51,58 | 50,53 | 41,78 | 49,37 | 55,09 | 43,41 | -0,20 | 7,73 | 0,0020 | 0,0327 |
| Rrs1 | 6,34 | 7,68 | 7,67 | 6,49 | 8,49 | 8,59 | 8,73 | 9,64 | 0,34 | 4,04 | 0,0020 | 0,0327 |
| Vim | 402,09 | 513,82 | 469,91 | 432,05 | 347,01 | 398,70 | 418,00 | 407,92 | -0,21 | 9,60 | 0,0020 | 0,0327 |
| Mfap1b | 22,24 | 28,28 | 28,07 | 25,08 | 29,64 | 31,64 | 29,86 | 30,12 | 0,23 | 5,73 | 0,0020 | 0,0329 |
| Slfn3 | 9,01 | 9,61 | 7,03 | 8,26 | 6,70 | 6,88 | 6,73 | 6,40 | -0,36 | 3,79 | 0,0020 | 0,0330 |
| Ost4 | 35,29 | 55,35 | 55,47 | 36,49 | 54,70 | 54,15 | 55,74 | 54,03 | 0,27 | 5,01 | 0,0020 | 0,0332 |
| Cyth2 | 21,54 | 24,22 | 23,46 | 22,16 | 25,57 | 25,42 | 27,35 | 27,80 | 0,22 | 5,97 | 0,0020 | 0,0332 |
| Spata5 | 22,82 | 18,26 | 17,06 | 18,69 | 15,67 | 17,87 | 17,29 | 14,86 | -0,23 | 5,70 | 0,0021 | 0,0333 |
| Gtf3c3 | 12,62 | 12,61 | 11,80 | 13,23 | 10,52 | 11,33 | 10,61 | 9,61 | -0,26 | 5,12 | 0,0021 | 0,0333 |
| Stk32c | 3,87 | 4,13 | 3,21 | 3,30 | 2,66 | 2,42 | 2,60 | 3,04 | -0,45 | 2,80 | 0,0021 | 0,0333 |
| Nsa2 | 58,55 | 73,60 | 69,36 | 61,23 | 72,32 | 73,11 | 75,02 | 81,71 | 0,20 | 7,57 | 0,0021 | 0,0333 |
| Arsb | 36,58 | 41,54 | 37,13 | 36,74 | 32,91 | 32,62 | 37,07 | 29,80 | -0,20 | 7,15 | 0,0021 | 0,0333 |
| Llph | 12,56 | 14,04 | 12,45 | 12,24 | 15,22 | 17,04 | 15,43 | 16,82 | 0,33 | 4,09 | 0,0021 | 0,0333 |
| Ahnak | 584,75 | 394,51 | 408,13 | 497,61 | 436,99 | 344,26 | 463,25 | 384,98 | -0,21 | 12,96 | 0,0021 | 0,0333 |
| Rnf40 | 26,92 | 33,76 | 34,88 | 29,83 | 37,43 | 35,97 | 34,05 | 36,36 | 0,20 | 7,13 | 0,0021 | 0,0335 |
| Prr14 | 41,55 | 50,43 | 51,39 | 42,68 | 53,98 | 53,37 | 53,69 | 52,30 | 0,20 | 6,83 | 0,0021 | 0,0337 |
| Dhx38 | 19,18 | 22,95 | 21,74 | 19,87 | 23,90 | 23,31 | 23,97 | 25,00 | 0,20 | 6,56 | 0,0021 | 0,0340 |
| Pqbp1 | 19,69 | 23,65 | 22,24 | 17,94 | 24,36 | 24,23 | 24,04 | 28,41 | 0,28 | 4,84 | 0,0022 | 0,0343 |
| Nfkb2 | 26,83 | 26,59 | 25,55 | 25,01 | 29,79 | 31,58 | 26,63 | 31,55 | 0,20 | 6,49 | 0,0022 | 0,0345 |
| Zfp277 | 30,64 | 20,57 | 21,85 | 22,36 | 19,95 | 20,41 | 21,52 | 19,92 | -0,22 | 5,77 | 0,0022 | 0,0352 |
| Cdk11b | 23,83 | 24,53 | 22,79 | 21,00 | 26,59 | 25,92 | 27,32 | 26,96 | 0,21 | 6,01 | 0,0022 | 0,0354 |
| Orai1 | 14,32 | 14,98 | 16,07 | 12,40 | 18,86 | 16,24 | 16,36 | 18,06 | 0,28 | 4,90 | 0,0022 | 0,0355 |
| Traf4 | 7,34 | 7,25 | 7,65 | 7,04 | 9,14 | 9,57 | 8,04 | 8,99 | 0,29 | 4,62 | 0,0023 | 0,0356 |
| Clip1 | 27,47 | 28,86 | 30,33 | 26,60 | 32,96 | 32,24 | 29,07 | 35,54 | 0,20 | 7,11 | 0,0023 | 0,0356 |
| Glce | 2,09 | 2,45 | 1,68 | 2,41 | 1,49 | 1,62 | 2,17 | 1,29 | -0,41 | 3,16 | 0,0023 | 0,0357 |
| Irs2 | 2,09 | 1,51 | 1,77 | 1,54 | 2,49 | 2,61 | 1,83 | 2,28 | 0,42 | 3,04 | 0,0023 | 0,0357 |
| Hist1h4j | 58,78 | 93,46 | 90,63 | 60,82 | 56,81 | 59,58 | 67,23 | 61,68 | -0,30 | 4,41 | 0,0023 | 0,0357 |
| Xcl1 | 1,38 | 1,56 | 1,51 | 0,78 | 2,74 | 2,91 | 1,45 | 2,46 | 0,89 | 0,07 | 0,0023 | 0,0357 |
| St8sia4 | 39,69 | 34,85 | 39,89 | 43,43 | 34,23 | 34,05 | 34,88 | 34,18 | -0,20 | 7,64 | 0,0023 | 0,0359 |
| Cep250 | 18,69 | 21,73 | 24,69 | 18,02 | 23,94 | 24,19 | 22,24 | 24,91 | 0,20 | 7,48 | 0,0023 | 0,0366 |
| Hspd1 | 45,35 | 54,58 | 50,20 | 46,57 | 53,85 | 53,25 | 55,23 | 63,07 | 0,20 | 6,95 | 0,0024 | 0,0367 |
| Xpo1 | 41,67 | 40,65 | 41,18 | 43,02 | 35,15 | 36,50 | 37,88 | 35,44 | -0,20 | 7,65 | 0,0024 | 0,0369 |
| Atp1b1 | 0,95 | 1,19 | 1,13 | 0,87 | 1,43 | 1,48 | 1,14 | 2,04 | 0,56 | 1,78 | 0,0024 | 0,0369 |
| Ate1 | 12,14 | 10,83 | 11,89 | 12,57 | 10,15 | 10,02 | 11,06 | 9,45 | -0,22 | 5,78 | 0,0024 | 0,0373 |
| Gfpt1 | 31,13 | 28,42 | 28,15 | 31,70 | 26,09 | 26,40 | 27,70 | 23,95 | -0,20 | 7,45 | 0,0024 | 0,0376 |
| Vamp2 | 59,13 | 54,92 | 50,30 | 55,40 | 65,67 | 58,18 | 64,83 | 63,19 | 0,19 | 7,00 | 0,0025 | 0,0380 |
| Gpsm2 | 4,74 | 6,27 | 5,23 | 4,18 | 3,39 | 3,96 | 4,65 | 4,26 | -0,33 | 4,00 | 0,0025 | 0,0380 |
| Tesc | 5,00 | 5,98 | 4,82 | 4,53 | 8,22 | 6,85 | 5,69 | 7,35 | 0,46 | 2,56 | 0,0025 | 0,0381 |
| Ceacam16 | 3,24 | 3,69 | 3,82 | 3,54 | 5,03 | 4,28 | 4,36 | 5,63 | 0,44 | 2,81 | 0,0025 | 0,0384 |
| Setx | 65,56 | 54,40 | 54,25 | 65,39 | 51,77 | 49,03 | 54,76 | 52,31 | -0,21 | 9,26 | 0,0025 | 0,0386 |
| Ndufa2 | 27,00 | 34,84 | 35,97 | 27,69 | 34,45 | 39,17 | 38,99 | 41,50 | 0,31 | 4,40 | 0,0025 | 0,0386 |
| Zgpat | 44,52 | 40,70 | 53,17 | 40,94 | 54,15 | 52,18 | 46,77 | 51,83 | 0,20 | 6,78 | 0,0025 | 0,0386 |
| Cd300a | 1,91 | 1,19 | 1,43 | 1,08 | 1,31 | 0,97 | 0,87 | 0,90 | -0,46 | 2,49 | 0,0025 | 0,0386 |
| Ltn1 | 22,30 | 19,99 | 20,73 | 23,78 | 18,76 | 18,77 | 19,95 | 18,34 | -0,20 | 7,30 | 0,0025 | 0,0388 |
| Gltscr2 | 190,71 | 229,39 | 256,68 | 200,79 | 250,71 | 257,98 | 248,91 | 252,70 | 0,20 | 8,52 | 0,0026 | 0,0392 |
| Xrn1 | 53,08 | 44,10 | 43,61 | 49,02 | 42,33 | 39,59 | 41,29 | 42,06 | -0,20 | 7,93 | 0,0026 | 0,0395 |
| Mea1 | 16,61 | 19,29 | 17,00 | 14,61 | 22,20 | 19,72 | 20,38 | 21,68 | 0,32 | 4,17 | 0,0026 | 0,0395 |
| Syf2 | 61,98 | 81,31 | 81,01 | 62,29 | 79,04 | 80,98 | 79,20 | 88,63 | 0,20 | 6,62 | 0,0026 | 0,0395 |
| Ticrr | 2,85 | 3,71 | 2,47 | 2,91 | 2,22 | 2,14 | 2,49 | 2,86 | -0,31 | 4,29 | 0,0026 | 0,0395 |
| Zyx | 192,22 | 262,22 | 281,27 | 198,73 | 270,29 | 268,16 | 271,89 | 265,69 | 0,20 | 9,26 | 0,0027 | 0,0400 |
| Klf6 | 87,29 | 87,68 | 78,69 | 102,74 | 95,93 | 89,89 | 106,15 | 118,74 | 0,20 | 8,66 | 0,0027 | 0,0402 |
| Rps11 | 726,49 | 898,48 | 875,48 | 683,70 | 961,05 | 902,07 | 844,46 | 959,14 | 0,20 | 9,01 | 0,0027 | 0,0402 |
| Mycbp2 | 50,02 | 32,73 | 34,64 | 42,67 | 36,68 | 32,23 | 34,93 | 35,16 | -0,20 | 9,15 | 0,0027 | 0,0402 |
| Rrp1 | 43,94 | 51,54 | 51,52 | 44,96 | 52,80 | 54,95 | 53,70 | 58,17 | 0,20 | 6,69 | 0,0027 | 0,0402 |
| Cab39l | 15,72 | 16,39 | 16,19 | 17,79 | 13,50 | 13,62 | 15,89 | 13,36 | -0,23 | 5,54 | 0,0027 | 0,0402 |
| Hic1 | 3,16 | 3,28 | 4,62 | 3,54 | 4,10 | 5,03 | 4,08 | 4,89 | 0,33 | 4,12 | 0,0027 | 0,0404 |
| Hypk | 3,72 | 6,57 | 7,52 | 4,30 | 8,19 | 8,67 | 6,31 | 8,03 | 0,54 | 2,04 | 0,0027 | 0,0404 |
| Ube3a | 33,65 | 26,39 | 28,52 | 30,47 | 24,53 | 26,39 | 26,52 | 26,65 | -0,19 | 7,15 | 0,0027 | 0,0406 |
| Arl6ip4 | 15,92 | 21,97 | 18,00 | 14,73 | 19,36 | 23,19 | 20,09 | 23,65 | 0,29 | 4,55 | 0,0028 | 0,0411 |
| Whsc1 | 13,23 | 12,61 | 11,61 | 12,12 | 10,44 | 10,93 | 11,35 | 10,59 | -0,20 | 6,50 | 0,0028 | 0,0414 |
| Syvn1 | 15,16 | 17,27 | 16,92 | 15,87 | 19,53 | 17,87 | 18,89 | 19,16 | 0,21 | 5,94 | 0,0028 | 0,0416 |
| Rps3 | 291,33 | 318,75 | 295,04 | 292,63 | 341,45 | 348,31 | 322,26 | 366,70 | 0,20 | 9,24 | 0,0028 | 0,0416 |
| Rad18 | 5,60 | 6,01 | 5,04 | 5,63 | 3,35 | 4,35 | 4,94 | 5,00 | -0,35 | 3,76 | 0,0028 | 0,0417 |
| Tmie | 1,32 | 1,53 | 1,79 | 1,35 | 2,54 | 1,84 | 1,70 | 2,29 | 0,51 | 2,17 | 0,0028 | 0,0418 |
| Smdt1 | 38,76 | 62,33 | 52,38 | 37,63 | 52,44 | 59,34 | 55,88 | 61,14 | 0,26 | 4,95 | 0,0028 | 0,0418 |
| Polr2g | 20,82 | 26,54 | 21,14 | 22,37 | 29,89 | 27,63 | 26,47 | 27,47 | 0,29 | 4,47 | 0,0029 | 0,0420 |
| Zkscan8 | 3,50 | 2,79 | 2,43 | 3,36 | 2,64 | 2,60 | 2,66 | 2,07 | -0,29 | 4,61 | 0,0029 | 0,0420 |
| Dync2h1 | 2,90 | 1,86 | 1,85 | 1,89 | 1,63 | 1,71 | 1,83 | 1,89 | -0,28 | 4,77 | 0,0029 | 0,0422 |
| Cwc27 | 5,34 | 5,05 | 5,87 | 3,49 | 3,54 | 3,90 | 3,64 | 3,73 | -0,40 | 3,01 | 0,0029 | 0,0422 |
| Dnajc7 | 43,27 | 46,46 | 49,45 | 43,79 | 51,72 | 51,49 | 50,43 | 55,31 | 0,19 | 6,83 | 0,0029 | 0,0429 |
| Rtcb | 41,85 | 46,53 | 40,76 | 44,42 | 46,96 | 49,90 | 49,26 | 52,67 | 0,19 | 6,53 | 0,0030 | 0,0434 |
| Picalm | 131,21 | 128,46 | 127,17 | 132,57 | 110,28 | 113,29 | 117,44 | 110,69 | -0,20 | 8,99 | 0,0030 | 0,0435 |
| H2-Q8 | 448,61 | 417,45 | 436,00 | 410,84 | 515,70 | 512,72 | 448,63 | 492,58 | 0,20 | 8,86 | 0,0030 | 0,0436 |
| Plk4 | 7,58 | 9,42 | 7,55 | 8,17 | 6,04 | 6,62 | 7,71 | 6,76 | -0,28 | 4,73 | 0,0030 | 0,0438 |
| H2-Q6 | 462,28 | 430,02 | 449,23 | 423,21 | 531,16 | 528,09 | 462,16 | 507,49 | 0,20 | 8,86 | 0,0030 | 0,0439 |
| Rfxank | 9,99 | 9,72 | 10,17 | 9,44 | 11,36 | 12,41 | 11,59 | 11,02 | 0,24 | 5,31 | 0,0030 | 0,0440 |
| Ybx1 | 53,81 | 81,15 | 71,05 | 52,21 | 68,63 | 72,57 | 75,51 | 77,97 | 0,19 | 7,24 | 0,0031 | 0,0453 |
| Hist1h4m | 92,16 | 124,88 | 141,50 | 97,58 | 87,22 | 100,39 | 107,84 | 90,88 | -0,23 | 5,41 | 0,0032 | 0,0455 |
| Jkamp | 10,15 | 10,17 | 10,05 | 11,76 | 9,12 | 8,21 | 9,65 | 7,19 | -0,30 | 4,24 | 0,0032 | 0,0457 |
| Vmn2r96 | 8,90 | 5,46 | 5,49 | 6,13 | 5,49 | 5,20 | 4,67 | 5,28 | -0,34 | 3,78 | 0,0032 | 0,0459 |
| Wdr89 | 9,54 | 11,16 | 15,39 | 9,69 | 12,95 | 14,48 | 11,83 | 14,52 | 0,25 | 5,21 | 0,0032 | 0,0462 |
| Kmt2c | 29,13 | 17,83 | 19,97 | 24,08 | 22,22 | 17,88 | 19,70 | 19,50 | -0,20 | 8,48 | 0,0032 | 0,0463 |
| Wbp2 | 42,06 | 50,04 | 49,37 | 43,57 | 51,22 | 53,10 | 54,89 | 52,12 | 0,19 | 6,48 | 0,0032 | 0,0465 |
| Bloc1s1 | 21,97 | 23,97 | 24,07 | 17,38 | 28,01 | 27,39 | 24,27 | 30,49 | 0,34 | 3,78 | 0,0033 | 0,0466 |
| Fbl | 21,36 | 31,46 | 28,89 | 21,87 | 30,48 | 27,30 | 28,56 | 36,75 | 0,25 | 5,04 | 0,0033 | 0,0467 |
| Rara | 41,54 | 49,48 | 51,95 | 40,15 | 54,01 | 50,74 | 53,81 | 50,29 | 0,19 | 7,37 | 0,0033 | 0,0467 |
| Dynlrb1 | 56,82 | 90,44 | 83,68 | 60,61 | 84,42 | 78,36 | 93,12 | 81,78 | 0,22 | 5,76 | 0,0033 | 0,0472 |
| Hist1h4n | 119,11 | 161,47 | 182,17 | 126,51 | 112,39 | 130,31 | 139,70 | 117,18 | -0,23 | 5,40 | 0,0034 | 0,0477 |
| Mbd2 | 112,03 | 126,97 | 125,60 | 110,50 | 134,31 | 130,20 | 138,27 | 140,82 | 0,20 | 7,88 | 0,0034 | 0,0480 |
| Prc1 | 15,13 | 21,68 | 18,23 | 18,25 | 15,90 | 14,51 | 17,22 | 15,41 | -0,22 | 5,69 | 0,0034 | 0,0480 |
| Tcf19 | 11,04 | 16,50 | 12,85 | 12,17 | 10,94 | 10,68 | 11,14 | 10,27 | -0,29 | 4,41 | 0,0034 | 0,0480 |
| Chst2 | 7,84 | 8,00 | 9,08 | 7,87 | 6,62 | 7,66 | 6,74 | 7,00 | -0,22 | 5,57 | 0,0034 | 0,0481 |
| Atf4 | 98,01 | 100,14 | 100,75 | 98,69 | 113,42 | 105,98 | 113,94 | 120,66 | 0,19 | 7,53 | 0,0034 | 0,0486 |
| Thoc2 | 36,68 | 29,63 | 31,12 | 33,66 | 27,96 | 27,79 | 30,92 | 27,95 | -0,19 | 7,87 | 0,0035 | 0,0490 |
| Rps10 | 517,18 | 650,00 | 668,10 | 496,66 | 670,97 | 673,93 | 635,78 | 691,93 | 0,20 | 8,49 | 0,0035 | 0,0492 |
| Pbx2 | 16,74 | 18,00 | 19,41 | 16,68 | 21,29 | 20,05 | 18,66 | 22,08 | 0,22 | 5,75 | 0,0035 | 0,0493 |
| Rpl8 | 494,40 | 645,39 | 653,32 | 493,97 | 635,19 | 652,83 | 613,61 | 721,21 | 0,20 | 9,05 | 0,0035 | 0,0497 |
| Txndc17 | 8,14 | 12,17 | 12,16 | 8,78 | 11,31 | 13,17 | 12,40 | 14,16 | 0,32 | 4,07 | 0,0036 | 0,0497 |
| Huwe1 | 66,36 | 45,27 | 47,09 | 60,18 | 51,00 | 43,19 | 50,93 | 45,75 | -0,20 | 9,55 | 0,0036 | 0,0498 |
| Prr5l | 4,33 | 4,46 | 4,16 | 4,19 | 3,58 | 3,64 | 3,06 | 3,55 | -0,31 | 4,06 | 0,0036 | 0,0498 |
| Socs1 | 8,87 | 8,75 | 10,52 | 6,92 | 9,77 | 12,91 | 8,48 | 12,97 | 0,35 | 3,73 | 0,0036 | 0,0500 |
| Furin | 28,99 | 30,15 | 31,31 | 30,11 | 33,79 | 34,91 | 33,86 | 34,80 | 0,19 | 7,11 | 0,0036 | 0,0500 |
| Eif3k | 73,30 | 89,71 | 94,27 | 71,58 | 95,00 | 91,63 | 94,01 | 94,55 | 0,20 | 6,36 | 0,0036 | 0,0500 |
| Psmc3 | 43,24 | 52,78 | 46,21 | 43,46 | 51,60 | 52,14 | 51,15 | 57,49 | 0,19 | 6,35 | 0,0036 | 0,0504 |
| Nedd1 | 12,08 | 11,26 | 12,39 | 12,75 | 9,89 | 10,50 | 10,51 | 10,32 | -0,23 | 5,31 | 0,0037 | 0,0506 |
| Klf2 | 249,63 | 340,73 | 349,85 | 261,53 | 347,32 | 345,45 | 334,31 | 350,26 | 0,20 | 9,19 | 0,0037 | 0,0509 |
| Entpd1 | 13,87 | 13,52 | 13,05 | 12,54 | 12,96 | 11,07 | 11,57 | 10,01 | -0,22 | 5,69 | 0,0037 | 0,0509 |
| Hsp90aa1 | 83,60 | 93,90 | 91,78 | 84,85 | 101,49 | 101,25 | 96,08 | 106,24 | 0,19 | 8,08 | 0,0037 | 0,0509 |
| Nudc | 16,16 | 25,10 | 20,43 | 17,91 | 24,82 | 22,25 | 24,00 | 24,09 | 0,26 | 4,89 | 0,0037 | 0,0512 |
| Sod1 | 119,65 | 152,39 | 121,72 | 117,89 | 148,79 | 137,84 | 143,56 | 153,92 | 0,19 | 6,48 | 0,0037 | 0,0513 |
| Cdk6 | 25,74 | 20,30 | 18,38 | 24,20 | 21,93 | 18,01 | 18,75 | 17,79 | -0,22 | 5,68 | 0,0038 | 0,0515 |
| Wscd2 | 1,64 | 1,41 | 1,34 | 1,89 | 1,29 | 1,31 | 1,19 | 0,82 | -0,46 | 2,40 | 0,0038 | 0,0515 |
| Rad54l2 | 17,93 | 17,13 | 15,85 | 19,86 | 16,09 | 14,51 | 16,37 | 15,22 | -0,19 | 7,28 | 0,0038 | 0,0520 |
| Cdc25c | 1,65 | 1,97 | 1,80 | 1,87 | 0,84 | 1,12 | 1,75 | 1,33 | -0,54 | 1,61 | 0,0038 | 0,0520 |
| Hist1h4h | 108,40 | 149,58 | 160,85 | 113,86 | 101,62 | 125,11 | 129,46 | 104,02 | -0,20 | 5,89 | 0,0039 | 0,0526 |
| Tnfrsf25 | 0,77 | 0,89 | 0,83 | 1,47 | 1,45 | 1,82 | 1,42 | 1,45 | 0,60 | 1,12 | 0,0039 | 0,0528 |
| Tpcn1 | 18,29 | 18,00 | 18,62 | 17,42 | 22,17 | 19,19 | 19,51 | 21,54 | 0,19 | 6,49 | 0,0039 | 0,0528 |
| Calcoco1 | 23,38 | 27,30 | 27,63 | 24,64 | 30,04 | 29,35 | 28,38 | 29,82 | 0,19 | 6,28 | 0,0039 | 0,0533 |
| Setd8 | 22,46 | 27,26 | 26,70 | 23,19 | 27,41 | 28,08 | 27,11 | 31,35 | 0,20 | 6,19 | 0,0040 | 0,0539 |
| Tmem167 | 10,76 | 8,41 | 8,94 | 9,82 | 8,02 | 8,50 | 8,49 | 8,33 | -0,19 | 6,59 | 0,0040 | 0,0539 |
| Ireb2 | 26,04 | 22,34 | 23,51 | 26,72 | 20,76 | 21,10 | 22,69 | 22,17 | -0,19 | 7,07 | 0,0040 | 0,0542 |
| Lair1 | 16,25 | 14,41 | 13,41 | 15,54 | 13,37 | 12,64 | 12,24 | 13,10 | -0,22 | 5,61 | 0,0040 | 0,0542 |
| Rps8 | 443,24 | 569,21 | 556,30 | 463,23 | 536,99 | 618,24 | 506,04 | 664,81 | 0,20 | 8,66 | 0,0040 | 0,0545 |
| Tmem63a | 19,98 | 20,93 | 20,91 | 23,45 | 24,85 | 25,09 | 23,70 | 23,93 | 0,19 | 6,21 | 0,0041 | 0,0549 |
| Bhlhe40 | 82,07 | 73,80 | 54,71 | 68,03 | 81,28 | 74,96 | 84,34 | 77,62 | 0,19 | 7,86 | 0,0042 | 0,0562 |
| Zc3h15 | 30,14 | 41,81 | 39,19 | 31,26 | 40,77 | 39,67 | 38,99 | 43,17 | 0,20 | 6,23 | 0,0042 | 0,0564 |
| Ptp4a1 | 40,96 | 35,63 | 34,81 | 40,89 | 41,59 | 42,28 | 41,94 | 47,59 | 0,19 | 7,40 | 0,0042 | 0,0566 |
| Ubxn4 | 27,45 | 27,08 | 29,04 | 26,46 | 30,62 | 31,54 | 30,04 | 32,82 | 0,19 | 6,91 | 0,0042 | 0,0568 |
| Smg1 | 74,03 | 47,46 | 52,54 | 65,48 | 53,48 | 47,80 | 54,92 | 53,14 | -0,19 | 9,77 | 0,0042 | 0,0568 |
| Rnf6 | 21,47 | 27,56 | 26,01 | 21,83 | 25,79 | 26,75 | 26,21 | 31,51 | 0,19 | 6,44 | 0,0043 | 0,0569 |
| Ebna1bp2 | 7,93 | 8,59 | 7,99 | 6,59 | 9,48 | 9,60 | 8,79 | 9,56 | 0,27 | 4,67 | 0,0043 | 0,0569 |
| Hist1h4k | 76,44 | 120,95 | 111,24 | 71,19 | 71,71 | 89,78 | 78,58 | 75,64 | -0,26 | 4,76 | 0,0043 | 0,0569 |
| Il18 | 4,94 | 4,81 | 3,93 | 4,25 | 3,43 | 2,68 | 3,46 | 3,03 | -0,52 | 1,76 | 0,0043 | 0,0569 |
| Crip2 | 5,05 | 5,55 | 4,67 | 3,85 | 3,17 | 3,71 | 3,66 | 3,64 | -0,43 | 2,59 | 0,0043 | 0,0569 |
| Eri2 | 5,44 | 4,49 | 4,85 | 5,56 | 4,00 | 3,95 | 4,57 | 3,94 | -0,31 | 4,04 | 0,0043 | 0,0569 |
| Aif1 | 2,14 | 1,68 | 1,08 | 1,06 | 0,98 | 1,90 | 0,47 | 0,17 | -0,80 | 0,17 | 0,0043 | 0,0572 |
| Ndufb3 | 16,99 | 24,69 | 24,71 | 12,72 | 21,92 | 24,53 | 24,41 | 28,83 | 0,35 | 3,58 | 0,0043 | 0,0572 |
| Bms1 | 17,38 | 20,14 | 20,40 | 17,96 | 20,69 | 21,51 | 21,19 | 22,94 | 0,19 | 6,44 | 0,0044 | 0,0580 |
| Cnpy4 | 5,07 | 6,22 | 4,78 | 5,56 | 6,84 | 6,76 | 6,73 | 7,36 | 0,34 | 3,49 | 0,0044 | 0,0583 |
| Myl12a | 106,76 | 156,44 | 145,50 | 106,69 | 146,27 | 145,19 | 145,90 | 150,00 | 0,19 | 7,94 | 0,0044 | 0,0583 |
| Lamtor2 | 26,41 | 34,33 | 33,95 | 23,19 | 36,09 | 33,70 | 37,77 | 36,39 | 0,30 | 4,23 | 0,0044 | 0,0584 |
| Ptbp2 | 13,97 | 8,14 | 9,59 | 10,65 | 8,29 | 9,39 | 9,08 | 9,09 | -0,24 | 5,04 | 0,0044 | 0,0584 |
| Fnip2 | 1,68 | 1,24 | 1,20 | 1,40 | 1,10 | 1,21 | 1,00 | 0,97 | -0,38 | 3,13 | 0,0045 | 0,0586 |
| Copg2 | 9,60 | 8,90 | 8,31 | 10,35 | 7,78 | 8,07 | 8,42 | 7,31 | -0,24 | 5,11 | 0,0045 | 0,0593 |
| Hspe1 | 30,08 | 38,67 | 35,83 | 27,07 | 38,43 | 37,72 | 39,49 | 41,49 | 0,26 | 4,80 | 0,0046 | 0,0602 |
| Itga1 | 13,06 | 10,16 | 10,51 | 14,56 | 10,72 | 9,44 | 11,93 | 10,21 | -0,20 | 6,09 | 0,0047 | 0,0619 |
| Eef1d | 24,69 | 32,12 | 27,69 | 24,49 | 29,54 | 31,04 | 29,93 | 33,57 | 0,19 | 6,38 | 0,0048 | 0,0620 |
| Hcst | 92,67 | 96,35 | 115,54 | 81,32 | 110,58 | 125,72 | 103,44 | 107,34 | 0,22 | 5,52 | 0,0048 | 0,0620 |
| Pltp | 1,54 | 2,04 | 1,94 | 2,04 | 2,50 | 2,59 | 3,12 | 2,30 | 0,48 | 2,08 | 0,0048 | 0,0621 |
| Ndufb10 | 31,24 | 36,58 | 37,38 | 30,11 | 39,84 | 38,48 | 42,68 | 41,76 | 0,27 | 4,57 | 0,0048 | 0,0625 |
| Il7r | 69,90 | 60,84 | 66,99 | 78,99 | 72,89 | 76,79 | 85,70 | 79,77 | 0,19 | 7,89 | 0,0048 | 0,0625 |
| Slc36a1 | 14,58 | 14,20 | 14,22 | 15,30 | 16,94 | 16,71 | 15,73 | 17,02 | 0,19 | 6,36 | 0,0048 | 0,0625 |
| Pou6f1 | 24,87 | 26,36 | 25,67 | 27,35 | 31,64 | 27,02 | 28,17 | 31,48 | 0,18 | 6,99 | 0,0048 | 0,0625 |
| Ssh3 | 6,20 | 5,77 | 5,79 | 5,39 | 7,15 | 7,19 | 6,86 | 7,24 | 0,30 | 4,15 | 0,0048 | 0,0626 |
| Os9 | 54,74 | 65,75 | 56,85 | 56,99 | 68,34 | 67,65 | 65,26 | 65,68 | 0,19 | 7,99 | 0,0049 | 0,0628 |
| Dpy19l4 | 6,77 | 5,62 | 5,17 | 7,18 | 5,02 | 5,01 | 5,19 | 4,96 | -0,30 | 4,08 | 0,0049 | 0,0630 |
| Ccl27a | 0,49 | 1,67 | 0,75 | 0,61 | 1,37 | 1,77 | 1,44 | 1,46 | 0,73 | 0,25 | 0,0049 | 0,0636 |
| Rexo4 | 21,61 | 25,52 | 26,00 | 21,60 | 27,44 | 27,32 | 24,44 | 29,58 | 0,20 | 5,86 | 0,0050 | 0,0636 |
| Tbxa2r | 20,49 | 21,98 | 28,34 | 20,40 | 26,93 | 26,75 | 24,25 | 27,39 | 0,22 | 5,58 | 0,0050 | 0,0640 |
| Mms22l | 10,49 | 8,81 | 8,43 | 8,43 | 7,96 | 7,65 | 7,36 | 7,91 | -0,23 | 5,23 | 0,0050 | 0,0646 |
| Rin3 | 47,77 | 54,38 | 54,52 | 50,78 | 60,41 | 56,82 | 60,36 | 58,23 | 0,19 | 7,74 | 0,0051 | 0,0653 |
| Rps26 | 523,62 | 678,55 | 685,42 | 479,50 | 640,18 | 681,93 | 685,99 | 685,67 | 0,19 | 8,15 | 0,0051 | 0,0653 |
| Phf14 | 19,34 | 16,01 | 15,71 | 14,57 | 13,90 | 14,55 | 14,76 | 14,70 | -0,18 | 6,84 | 0,0051 | 0,0654 |
| Zfp703 | 5,10 | 7,18 | 7,53 | 5,28 | 7,94 | 7,61 | 6,68 | 7,90 | 0,28 | 4,52 | 0,0052 | 0,0659 |
| Zfp622 | 20,55 | 25,56 | 25,68 | 22,36 | 25,45 | 26,74 | 26,39 | 28,73 | 0,19 | 6,14 | 0,0052 | 0,0661 |
| Parp3 | 18,87 | 20,12 | 22,27 | 17,84 | 21,72 | 25,51 | 21,62 | 22,00 | 0,21 | 5,81 | 0,0052 | 0,0665 |
| Zranb3 | 1,23 | 1,62 | 1,19 | 1,27 | 0,94 | 0,81 | 1,26 | 0,90 | -0,45 | 2,31 | 0,0052 | 0,0665 |
| H2-Ke2 | 11,75 | 22,83 | 21,83 | 11,73 | 17,95 | 21,09 | 21,03 | 24,43 | 0,33 | 3,78 | 0,0053 | 0,0667 |
| Spdl1 | 1,62 | 3,32 | 2,34 | 1,60 | 0,75 | 1,63 | 2,04 | 2,07 | -0,46 | 2,28 | 0,0053 | 0,0667 |
| Clk2 | 38,63 | 42,03 | 46,18 | 39,08 | 47,76 | 47,74 | 44,46 | 47,95 | 0,18 | 6,68 | 0,0053 | 0,0667 |
| Mrps15 | 13,40 | 17,14 | 16,64 | 10,14 | 17,29 | 17,13 | 17,33 | 18,99 | 0,32 | 3,92 | 0,0053 | 0,0670 |
| Fanci | 1,60 | 2,15 | 1,58 | 1,93 | 1,25 | 1,29 | 1,57 | 1,47 | -0,39 | 2,91 | 0,0053 | 0,0670 |
| Ddrgk1 | 19,67 | 23,99 | 23,96 | 19,33 | 25,95 | 26,95 | 24,54 | 26,38 | 0,26 | 4,72 | 0,0053 | 0,0670 |
| Rnf126 | 17,00 | 20,03 | 23,66 | 19,21 | 24,12 | 23,48 | 22,58 | 23,30 | 0,24 | 5,15 | 0,0054 | 0,0673 |
| Anxa2 | 129,87 | 171,13 | 127,19 | 143,50 | 111,29 | 119,90 | 138,55 | 134,56 | -0,18 | 7,53 | 0,0054 | 0,0673 |
| Gabpb1 | 7,08 | 5,74 | 5,78 | 6,04 | 4,71 | 5,37 | 5,12 | 5,00 | -0,29 | 4,19 | 0,0054 | 0,0675 |
| Strn3 | 18,03 | 14,82 | 16,22 | 16,53 | 14,20 | 14,10 | 14,81 | 14,26 | -0,19 | 6,00 | 0,0054 | 0,0675 |
| Map7d1 | 39,21 | 47,79 | 48,26 | 38,64 | 50,33 | 47,87 | 46,82 | 51,85 | 0,18 | 7,29 | 0,0055 | 0,0683 |
| Cdk16 | 18,26 | 16,79 | 18,06 | 16,63 | 21,33 | 20,42 | 18,89 | 19,43 | 0,20 | 5,84 | 0,0055 | 0,0683 |
| Nars | 26,82 | 30,99 | 28,19 | 26,31 | 32,05 | 32,00 | 30,39 | 33,29 | 0,19 | 6,32 | 0,0055 | 0,0683 |
| Ckap2 | 4,56 | 6,39 | 5,64 | 5,34 | 3,49 | 3,58 | 5,06 | 5,38 | -0,32 | 3,68 | 0,0055 | 0,0687 |
| Uqcr11 | 19,87 | 38,22 | 40,78 | 20,81 | 37,79 | 37,55 | 38,63 | 33,13 | 0,33 | 3,87 | 0,0055 | 0,0689 |
| Rb1cc1 | 30,61 | 26,19 | 28,00 | 30,37 | 25,05 | 25,43 | 26,79 | 24,28 | -0,18 | 7,58 | 0,0056 | 0,0692 |
| Dedd | 12,03 | 11,35 | 11,00 | 11,16 | 14,07 | 13,03 | 13,16 | 13,34 | 0,23 | 5,09 | 0,0056 | 0,0698 |
| Atp8a1 | 9,23 | 6,52 | 6,79 | 7,49 | 6,78 | 6,91 | 6,15 | 6,35 | -0,20 | 5,85 | 0,0057 | 0,0700 |
| Fbxl2 | 5,48 | 3,76 | 4,52 | 4,72 | 4,12 | 3,96 | 3,74 | 2,97 | -0,32 | 3,74 | 0,0057 | 0,0705 |
| Zfp292 | 33,39 | 24,95 | 26,76 | 28,59 | 25,25 | 24,03 | 25,67 | 25,10 | -0,18 | 8,06 | 0,0057 | 0,0705 |
| Ly6e | 197,50 | 237,60 | 214,56 | 188,16 | 240,34 | 232,99 | 249,44 | 231,38 | 0,19 | 9,00 | 0,0057 | 0,0705 |
| Mbnl3 | 14,21 | 12,69 | 12,43 | 15,05 | 12,22 | 11,04 | 11,90 | 12,94 | -0,18 | 6,80 | 0,0057 | 0,0706 |
| Rpl12 | 603,78 | 678,34 | 601,81 | 526,92 | 703,43 | 678,31 | 635,69 | 728,29 | 0,19 | 8,68 | 0,0057 | 0,0706 |
| Srsf5 | 206,87 | 227,57 | 209,96 | 227,16 | 239,04 | 242,48 | 250,49 | 260,30 | 0,19 | 8,59 | 0,0058 | 0,0717 |
| Orc3 | 11,61 | 8,95 | 10,04 | 9,92 | 9,02 | 8,80 | 8,93 | 8,62 | -0,20 | 5,90 | 0,0058 | 0,0717 |
| Dda1 | 30,53 | 38,43 | 40,03 | 33,82 | 41,80 | 39,06 | 41,59 | 39,19 | 0,18 | 6,44 | 0,0059 | 0,0721 |
| Zfp935 | 14,82 | 12,91 | 13,85 | 14,02 | 12,51 | 11,54 | 12,20 | 10,87 | -0,24 | 4,99 | 0,0059 | 0,0721 |
| Zfp236 | 20,27 | 16,79 | 17,55 | 19,49 | 17,20 | 16,08 | 16,42 | 15,80 | -0,18 | 7,37 | 0,0059 | 0,0726 |
| Gpd2 | 10,77 | 9,43 | 9,20 | 10,57 | 9,53 | 8,08 | 9,59 | 7,60 | -0,20 | 5,75 | 0,0060 | 0,0730 |
| Tsc22d4 | 62,35 | 70,29 | 75,80 | 61,56 | 76,80 | 79,25 | 71,20 | 78,28 | 0,18 | 7,49 | 0,0060 | 0,0735 |
| Hist1h2bf | 34,67 | 56,98 | 55,10 | 33,07 | 32,52 | 34,64 | 41,47 | 36,36 | -0,30 | 3,94 | 0,0060 | 0,0737 |
| Ctdnep1 | 38,70 | 47,97 | 52,09 | 40,19 | 49,85 | 49,13 | 50,83 | 53,00 | 0,19 | 6,27 | 0,0061 | 0,0745 |
| Klrg1 | 11,25 | 12,61 | 11,99 | 14,34 | 9,90 | 10,01 | 13,81 | 7,07 | -0,30 | 3,96 | 0,0061 | 0,0747 |
| Lactb2 | 19,33 | 19,56 | 22,20 | 20,40 | 16,58 | 17,83 | 19,30 | 15,73 | -0,23 | 5,15 | 0,0062 | 0,0749 |
| Itpr1 | 19,88 | 17,23 | 17,18 | 18,30 | 16,26 | 15,25 | 16,77 | 15,91 | -0,18 | 7,40 | 0,0062 | 0,0749 |
| Ik | 29,33 | 40,46 | 38,19 | 32,57 | 37,16 | 39,55 | 38,69 | 44,19 | 0,19 | 6,23 | 0,0062 | 0,0753 |
| Unc119 | 17,03 | 19,36 | 19,79 | 17,42 | 23,04 | 23,40 | 20,02 | 21,20 | 0,26 | 4,70 | 0,0063 | 0,0759 |
| Usp34 | 44,54 | 32,32 | 35,87 | 39,87 | 35,18 | 30,84 | 34,19 | 34,00 | -0,19 | 8,77 | 0,0063 | 0,0760 |
| Pign | 9,12 | 6,83 | 6,75 | 8,44 | 6,55 | 6,72 | 6,89 | 6,89 | -0,21 | 5,63 | 0,0063 | 0,0760 |
| Polq | 1,40 | 1,64 | 1,27 | 1,47 | 1,05 | 0,98 | 1,31 | 1,27 | -0,34 | 3,48 | 0,0063 | 0,0760 |
| Hist1h2bp | 27,10 | 39,27 | 37,25 | 27,82 | 25,86 | 25,87 | 29,04 | 26,65 | -0,28 | 4,15 | 0,0064 | 0,0767 |
| Fbxl4 | 5,08 | 3,89 | 4,35 | 5,16 | 3,97 | 3,74 | 4,10 | 2,82 | -0,34 | 3,41 | 0,0064 | 0,0769 |
| Il4ra | 21,67 | 22,05 | 21,52 | 23,52 | 25,12 | 23,91 | 25,16 | 26,12 | 0,18 | 6,91 | 0,0064 | 0,0770 |
| Zmym4 | 7,98 | 6,38 | 6,71 | 7,09 | 6,64 | 6,03 | 5,62 | 6,08 | -0,21 | 5,53 | 0,0064 | 0,0772 |
| Nolc1 | 8,30 | 10,42 | 11,17 | 8,38 | 10,80 | 11,48 | 10,06 | 12,18 | 0,23 | 5,25 | 0,0065 | 0,0772 |
| Ngly1 | 43,65 | 40,85 | 40,36 | 46,90 | 38,19 | 36,00 | 38,63 | 39,33 | -0,18 | 6,88 | 0,0065 | 0,0775 |
| Zdhhc2 | 17,12 | 16,70 | 16,47 | 17,47 | 14,04 | 15,91 | 14,18 | 14,12 | -0,22 | 5,30 | 0,0065 | 0,0778 |
| Prr13 | 132,00 | 160,10 | 146,43 | 131,34 | 159,25 | 157,86 | 169,08 | 158,52 | 0,18 | 7,55 | 0,0065 | 0,0779 |
| Exoc4 | 27,87 | 23,80 | 23,25 | 24,23 | 22,14 | 21,14 | 23,17 | 21,22 | -0,18 | 6,44 | 0,0066 | 0,0781 |
| Dock5 | 49,64 | 40,55 | 42,60 | 47,98 | 41,40 | 37,88 | 43,20 | 36,62 | -0,18 | 8,78 | 0,0066 | 0,0784 |
| Cdan1 | 10,71 | 10,28 | 10,87 | 9,96 | 12,19 | 11,77 | 11,55 | 12,00 | 0,19 | 6,16 | 0,0066 | 0,0784 |
| Sytl1 | 12,86 | 13,67 | 15,18 | 12,46 | 15,78 | 16,57 | 15,68 | 15,99 | 0,25 | 4,83 | 0,0066 | 0,0788 |
| Chek1 | 1,28 | 1,70 | 1,20 | 1,37 | 1,01 | 1,13 | 1,08 | 0,84 | -0,46 | 2,06 | 0,0067 | 0,0788 |
| Ptpn6 | 84,00 | 85,70 | 91,59 | 77,00 | 99,03 | 95,02 | 89,68 | 98,95 | 0,18 | 7,65 | 0,0067 | 0,0792 |
| Rps20 | 293,56 | 330,45 | 340,41 | 297,81 | 326,37 | 378,25 | 332,01 | 389,18 | 0,18 | 7,40 | 0,0067 | 0,0796 |
| Trim65 | 13,53 | 12,37 | 15,44 | 12,97 | 16,45 | 15,25 | 14,17 | 16,82 | 0,21 | 5,48 | 0,0068 | 0,0802 |
| Gpcpd1 | 33,82 | 26,79 | 27,35 | 30,10 | 25,19 | 26,71 | 28,35 | 24,36 | -0,18 | 6,69 | 0,0069 | 0,0809 |
| Ankrd32 | 6,50 | 5,25 | 5,31 | 6,07 | 4,81 | 5,36 | 4,66 | 4,35 | -0,28 | 4,31 | 0,0069 | 0,0814 |
| Chtf18 | 1,84 | 3,11 | 2,53 | 2,21 | 1,57 | 1,93 | 1,93 | 1,94 | -0,40 | 2,73 | 0,0069 | 0,0815 |
| Ubn2 | 17,95 | 11,97 | 12,39 | 15,39 | 13,14 | 12,24 | 13,27 | 12,40 | -0,18 | 7,64 | 0,0070 | 0,0822 |
| Exoc2 | 36,98 | 35,09 | 31,62 | 34,00 | 29,92 | 29,47 | 32,57 | 30,13 | -0,18 | 7,11 | 0,0070 | 0,0822 |
| Tbck | 19,02 | 13,49 | 14,51 | 18,00 | 14,90 | 13,72 | 14,93 | 13,13 | -0,20 | 5,69 | 0,0070 | 0,0822 |
| Rpe | 27,61 | 24,88 | 25,82 | 27,35 | 22,35 | 24,08 | 21,96 | 24,51 | -0,19 | 6,03 | 0,0070 | 0,0822 |
| Fam129b | 5,66 | 6,75 | 5,94 | 4,94 | 4,15 | 5,69 | 4,81 | 4,64 | -0,27 | 4,30 | 0,0071 | 0,0828 |
| Lrpap1 | 10,65 | 10,94 | 9,63 | 10,36 | 12,62 | 11,89 | 11,62 | 12,71 | 0,23 | 5,05 | 0,0071 | 0,0829 |
| Tmem41b | 6,26 | 6,99 | 6,59 | 6,79 | 5,14 | 5,62 | 5,70 | 5,76 | -0,26 | 4,47 | 0,0072 | 0,0836 |
| Trib1 | 3,04 | 4,39 | 3,24 | 3,19 | 4,09 | 3,82 | 4,75 | 4,39 | 0,29 | 3,98 | 0,0072 | 0,0836 |
| Hist1h4c | 79,18 | 119,06 | 138,82 | 79,84 | 86,19 | 92,04 | 91,09 | 81,50 | -0,23 | 4,89 | 0,0072 | 0,0841 |
| Fnip1 | 8,82 | 7,37 | 7,65 | 8,34 | 6,74 | 7,15 | 7,34 | 6,70 | -0,21 | 5,56 | 0,0072 | 0,0841 |
| Bcor | 19,30 | 21,22 | 21,34 | 20,07 | 23,44 | 22,23 | 22,31 | 24,44 | 0,17 | 7,24 | 0,0073 | 0,0843 |
| Hnrnpc | 104,74 | 96,17 | 98,79 | 111,57 | 87,32 | 87,67 | 98,40 | 89,87 | -0,18 | 8,11 | 0,0073 | 0,0844 |
| Otud1 | 7,48 | 6,36 | 4,90 | 7,19 | 7,89 | 7,83 | 7,41 | 8,51 | 0,27 | 4,21 | 0,0073 | 0,0850 |
| Wdhd1 | 8,49 | 8,74 | 7,79 | 7,53 | 6,77 | 6,52 | 7,39 | 7,05 | -0,23 | 4,98 | 0,0074 | 0,0850 |
| Map1lc3a | 3,79 | 3,10 | 3,53 | 3,57 | 4,45 | 4,00 | 3,87 | 6,77 | 0,45 | 2,16 | 0,0074 | 0,0850 |
| Hspa8 | 914,31 | 935,60 | 961,12 | 923,82 | 1079,83 | 1024,40 | 997,71 | 1137,13 | 0,18 | 11,03 | 0,0074 | 0,0850 |
| Ddit3 | 6,26 | 7,65 | 8,58 | 7,43 | 9,93 | 9,43 | 8,81 | 10,51 | 0,38 | 2,92 | 0,0074 | 0,0854 |
| Anp32a | 52,55 | 64,19 | 54,56 | 53,72 | 63,90 | 61,17 | 63,74 | 64,94 | 0,17 | 6,97 | 0,0075 | 0,0866 |
| Asah1 | 13,61 | 14,39 | 12,81 | 14,07 | 11,53 | 11,77 | 11,46 | 11,80 | -0,24 | 4,84 | 0,0076 | 0,0869 |
| Med31 | 2,16 | 2,03 | 1,95 | 1,69 | 2,48 | 2,52 | 3,25 | 2,94 | 0,51 | 1,54 | 0,0076 | 0,0869 |
| Tsen54 | 7,00 | 7,38 | 8,92 | 7,01 | 9,36 | 8,61 | 9,12 | 9,71 | 0,29 | 4,06 | 0,0077 | 0,0878 |
| Ccdc82 | 50,37 | 40,37 | 41,74 | 41,49 | 38,59 | 38,46 | 39,43 | 37,70 | -0,17 | 7,57 | 0,0078 | 0,0889 |
| Ubfd1 | 14,51 | 12,08 | 12,72 | 14,81 | 15,55 | 14,50 | 14,76 | 16,64 | 0,18 | 6,13 | 0,0078 | 0,0889 |
| Tmsb15b2 | 2,91 | 4,32 | 4,17 | 2,95 | 4,36 | 5,70 | 4,27 | 5,96 | 0,51 | 1,61 | 0,0078 | 0,0889 |
| Glcci1 | 20,06 | 20,51 | 19,78 | 21,99 | 17,96 | 18,04 | 19,01 | 18,11 | -0,17 | 6,91 | 0,0078 | 0,0889 |
| Nedd8 | 48,94 | 62,93 | 52,94 | 48,54 | 63,46 | 55,48 | 64,78 | 65,33 | 0,22 | 5,13 | 0,0078 | 0,0890 |
| Narf | 18,16 | 17,86 | 17,38 | 18,35 | 20,78 | 19,22 | 20,44 | 20,63 | 0,18 | 6,40 | 0,0078 | 0,0890 |
| Chm | 16,28 | 14,75 | 15,89 | 16,07 | 13,07 | 14,83 | 14,30 | 13,38 | -0,18 | 6,17 | 0,0078 | 0,0890 |
| Ncapd3 | 18,70 | 17,55 | 16,51 | 17,58 | 16,64 | 14,68 | 16,13 | 14,96 | -0,17 | 6,51 | 0,0079 | 0,0893 |
| Hnrnpm | 44,07 | 50,26 | 48,93 | 41,04 | 48,99 | 51,26 | 51,86 | 55,39 | 0,17 | 6,96 | 0,0079 | 0,0894 |
| Lactb | 7,45 | 9,36 | 8,66 | 8,23 | 10,38 | 9,48 | 9,59 | 11,28 | 0,27 | 4,25 | 0,0079 | 0,0895 |
| Intu | 3,68 | 2,05 | 1,90 | 2,77 | 2,49 | 1,96 | 2,20 | 1,87 | -0,30 | 3,93 | 0,0080 | 0,0898 |
| Fam126b | 4,33 | 3,36 | 2,86 | 4,06 | 3,36 | 2,75 | 3,19 | 3,16 | -0,24 | 4,89 | 0,0080 | 0,0898 |
| Hist1h2bh | 35,51 | 60,45 | 63,29 | 37,97 | 37,04 | 39,27 | 41,83 | 42,85 | -0,27 | 4,08 | 0,0080 | 0,0902 |
| Calcrl | 6,04 | 5,59 | 5,37 | 6,73 | 4,48 | 5,14 | 5,20 | 5,24 | -0,25 | 4,70 | 0,0080 | 0,0903 |
| Armc7 | 95,41 | 99,21 | 92,05 | 96,85 | 110,13 | 107,59 | 107,38 | 108,21 | 0,18 | 7,84 | 0,0081 | 0,0910 |
| Ash1l | 32,96 | 21,61 | 24,01 | 26,28 | 25,18 | 22,23 | 23,15 | 22,21 | -0,18 | 8,12 | 0,0081 | 0,0910 |
| Pbrm1 | 43,20 | 40,74 | 41,03 | 46,75 | 38,07 | 37,36 | 39,31 | 37,05 | -0,18 | 8,36 | 0,0081 | 0,0911 |
| Ccdc59 | 13,89 | 17,18 | 14,48 | 15,10 | 16,68 | 19,07 | 17,69 | 20,15 | 0,27 | 4,19 | 0,0081 | 0,0913 |
| Bag3 | 5,05 | 6,82 | 6,46 | 6,30 | 8,23 | 7,05 | 6,64 | 7,92 | 0,28 | 4,16 | 0,0082 | 0,0914 |
| Rgs2 | 6,43 | 4,92 | 3,93 | 6,26 | 6,76 | 6,18 | 5,82 | 7,48 | 0,26 | 4,19 | 0,0082 | 0,0915 |
| Gnptg | 48,92 | 47,10 | 46,94 | 45,89 | 54,40 | 55,90 | 53,61 | 50,88 | 0,19 | 5,96 | 0,0082 | 0,0917 |
| Rbm8a | 10,06 | 14,71 | 12,18 | 11,19 | 13,27 | 14,91 | 13,16 | 14,88 | 0,22 | 5,10 | 0,0082 | 0,0918 |
| Asxl2 | 35,72 | 26,91 | 27,91 | 32,46 | 29,49 | 26,03 | 27,96 | 25,42 | -0,18 | 8,03 | 0,0083 | 0,0918 |
| Cfdp1 | 15,84 | 23,11 | 21,64 | 15,38 | 22,59 | 20,32 | 22,50 | 24,55 | 0,25 | 4,66 | 0,0083 | 0,0918 |
| Arhgap11a | 8,35 | 12,73 | 9,05 | 9,80 | 7,77 | 8,18 | 8,85 | 9,99 | -0,21 | 5,55 | 0,0083 | 0,0923 |
| Hsd17b10 | 16,79 | 19,47 | 18,89 | 18,08 | 25,08 | 19,30 | 23,07 | 20,96 | 0,28 | 4,22 | 0,0083 | 0,0925 |
| Nfkbiz | 48,31 | 39,26 | 35,10 | 36,44 | 45,97 | 45,17 | 42,20 | 45,98 | 0,17 | 7,35 | 0,0083 | 0,0925 |
| Rere | 15,57 | 17,24 | 16,09 | 16,73 | 18,71 | 18,02 | 18,85 | 18,29 | 0,17 | 7,03 | 0,0084 | 0,0925 |
| Ncl | 42,99 | 48,78 | 43,81 | 42,71 | 48,51 | 48,10 | 50,06 | 55,16 | 0,18 | 8,62 | 0,0084 | 0,0926 |
| BC037034 | 35,54 | 34,92 | 42,72 | 36,50 | 42,31 | 44,90 | 40,52 | 40,53 | 0,17 | 6,66 | 0,0084 | 0,0929 |
| Dhfr | 1,74 | 2,08 | 1,68 | 1,75 | 1,55 | 1,35 | 1,55 | 1,23 | -0,35 | 3,11 | 0,0084 | 0,0929 |
| Phf11a | 23,62 | 27,87 | 21,58 | 25,10 | 21,70 | 21,25 | 22,70 | 18,27 | -0,23 | 4,95 | 0,0085 | 0,0933 |
| Chid1 | 5,98 | 4,32 | 5,20 | 5,58 | 4,44 | 4,41 | 4,74 | 3,95 | -0,26 | 4,35 | 0,0085 | 0,0933 |
| Anapc1 | 27,48 | 25,98 | 24,97 | 28,74 | 24,10 | 22,76 | 25,50 | 22,64 | -0,17 | 7,82 | 0,0085 | 0,0939 |
| Hmga1 | 45,23 | 53,09 | 52,90 | 45,71 | 57,73 | 52,37 | 53,08 | 58,31 | 0,17 | 6,71 | 0,0086 | 0,0939 |
| Rpl6 | 467,60 | 563,30 | 554,10 | 460,05 | 575,33 | 577,07 | 547,69 | 614,10 | 0,18 | 9,41 | 0,0086 | 0,0940 |
| Prr14l | 31,14 | 24,68 | 26,58 | 30,35 | 26,31 | 23,49 | 25,51 | 24,53 | -0,18 | 8,13 | 0,0086 | 0,0940 |
| Tnfrsf22 | 8,53 | 8,96 | 8,73 | 11,06 | 10,55 | 10,30 | 12,33 | 11,31 | 0,25 | 4,55 | 0,0086 | 0,0940 |
| Ranbp2 | 53,23 | 41,22 | 40,56 | 50,98 | 41,43 | 38,12 | 44,02 | 40,84 | -0,18 | 8,70 | 0,0086 | 0,0940 |
| Rpl34 | 350,55 | 379,02 | 410,05 | 305,60 | 413,26 | 420,85 | 374,71 | 421,31 | 0,18 | 7,92 | 0,0086 | 0,0943 |
| Fbxo5 | 10,15 | 13,91 | 9,21 | 9,88 | 8,19 | 8,36 | 10,08 | 9,20 | -0,28 | 4,17 | 0,0087 | 0,0943 |
| Usf1 | 28,84 | 33,64 | 31,11 | 30,05 | 36,22 | 35,41 | 33,18 | 35,86 | 0,19 | 5,92 | 0,0087 | 0,0943 |
| Map3k14 | 16,84 | 16,05 | 15,64 | 16,47 | 19,57 | 18,88 | 17,32 | 17,81 | 0,18 | 6,18 | 0,0087 | 0,0948 |
| Myeov2 | 29,54 | 43,41 | 39,08 | 28,32 | 41,94 | 47,16 | 39,39 | 41,45 | 0,28 | 4,10 | 0,0088 | 0,0956 |
| Ifng | 62,73 | 55,46 | 50,22 | 48,83 | 63,70 | 59,85 | 60,49 | 62,10 | 0,18 | 6,13 | 0,0089 | 0,0961 |
| Hmga1-rs1 | 56,15 | 65,80 | 65,59 | 56,58 | 71,41 | 64,68 | 65,93 | 72,40 | 0,17 | 6,70 | 0,0089 | 0,0962 |
| Rbfa | 22,72 | 25,73 | 28,39 | 26,55 | 28,12 | 29,99 | 30,45 | 31,22 | 0,22 | 5,23 | 0,0089 | 0,0966 |
| Ska3 | 3,03 | 3,49 | 2,47 | 3,34 | 2,28 | 2,18 | 2,65 | 2,34 | -0,40 | 2,65 | 0,0090 | 0,0968 |
| Gpr52 | 3,35 | 1,90 | 2,44 | 1,81 | 1,51 | 1,67 | 1,80 | 1,50 | -0,54 | 1,15 | 0,0090 | 0,0968 |
| Mavs | 12,36 | 14,17 | 12,97 | 12,52 | 15,29 | 15,58 | 14,29 | 14,82 | 0,20 | 5,44 | 0,0090 | 0,0968 |
| Atp7a | 6,69 | 5,72 | 5,74 | 7,07 | 5,54 | 5,46 | 5,82 | 5,21 | -0,20 | 5,60 | 0,0090 | 0,0968 |
| Ubb | 523,24 | 659,93 | 580,90 | 530,40 | 658,33 | 659,82 | 665,44 | 611,26 | 0,18 | 9,48 | 0,0090 | 0,0968 |
| Utp14a | 13,55 | 17,74 | 13,50 | 13,31 | 15,57 | 16,66 | 17,09 | 18,13 | 0,21 | 5,26 | 0,0090 | 0,0970 |
| Tmsb15l | 2,67 | 4,06 | 3,69 | 2,63 | 3,88 | 5,28 | 3,82 | 5,24 | 0,49 | 1,73 | 0,0091 | 0,0973 |
| Atp6v0b | 33,29 | 29,33 | 35,13 | 32,00 | 39,44 | 38,24 | 36,70 | 36,31 | 0,22 | 5,13 | 0,0091 | 0,0973 |
| Ppcdc | 10,75 | 11,21 | 9,58 | 9,52 | 12,06 | 12,20 | 11,60 | 12,32 | 0,23 | 4,95 | 0,0091 | 0,0976 |
| Cherp | 28,04 | 30,60 | 31,58 | 26,04 | 33,15 | 32,53 | 32,98 | 31,90 | 0,17 | 6,87 | 0,0091 | 0,0976 |
| Ppm1m | 28,42 | 28,71 | 29,69 | 26,69 | 31,78 | 33,50 | 31,55 | 32,60 | 0,19 | 5,78 | 0,0091 | 0,0977 |
| Imp3 | 28,19 | 33,09 | 32,51 | 29,67 | 33,12 | 37,10 | 36,21 | 38,25 | 0,23 | 4,92 | 0,0093 | 0,0989 |
| Atp5j | 39,57 | 67,82 | 73,98 | 35,77 | 55,63 | 63,61 | 60,08 | 69,05 | 0,21 | 5,46 | 0,0093 | 0,0991 |
| Wbp4 | 8,12 | 10,43 | 10,63 | 8,74 | 10,89 | 11,16 | 10,30 | 11,62 | 0,22 | 5,15 | 0,0093 | 0,0993 |
| Rpl34-ps1 | 531,74 | 575,74 | 622,83 | 463,98 | 627,45 | 637,74 | 567,58 | 638,96 | 0,17 | 7,91 | 0,0094 | 0,1000 |
| Tcta | 5,88 | 4,45 | 6,04 | 5,75 | 3,78 | 4,45 | 4,96 | 4,07 | -0,35 | 3,00 | 0,0094 | 0,1000 |
| Dvl2 | 13,23 | 15,31 | 14,63 | 14,52 | 18,06 | 16,44 | 15,38 | 16,40 | 0,20 | 5,51 | 0,0094 | 0,1000 |
| Exosc3 | 14,22 | 24,69 | 22,96 | 15,93 | 20,97 | 23,34 | 22,88 | 25,31 | 0,26 | 4,47 | 0,0094 | 0,1000 |
| Exoc1 | 18,45 | 18,24 | 17,03 | 17,21 | 14,33 | 17,40 | 16,34 | 14,25 | -0,19 | 5,81 | 0,0094 | 0,1000 |
| Farp1 | 2,26 | 2,03 | 2,30 | 1,96 | 1,51 | 1,94 | 1,79 | 1,50 | -0,34 | 3,21 | 0,0094 | 0,1000 |
| Hs6st1 | 6,71 | 6,97 | 6,48 | 6,66 | 8,31 | 7,87 | 7,37 | 8,10 | 0,24 | 4,78 | 0,0095 | 0,1003 |
| Dmxl1 | 14,64 | 9,87 | 10,53 | 12,97 | 10,97 | 10,69 | 10,89 | 10,23 | -0,17 | 7,12 | 0,0096 | 0,1009 |
| Acin1 | 69,35 | 73,57 | 75,84 | 66,13 | 82,79 | 79,03 | 76,12 | 83,65 | 0,18 | 8,49 | 0,0096 | 0,1009 |
| Rpl27a | 232,50 | 313,70 | 304,46 | 233,22 | 303,03 | 311,07 | 290,86 | 317,48 | 0,17 | 8,32 | 0,0096 | 0,1015 |
| Setd1b | 26,31 | 28,67 | 30,78 | 27,58 | 34,07 | 29,49 | 31,83 | 32,31 | 0,17 | 8,06 | 0,0097 | 0,1015 |
| Lrrc8b | 4,14 | 5,73 | 5,33 | 4,69 | 3,73 | 4,08 | 4,47 | 3,83 | -0,30 | 3,68 | 0,0097 | 0,1015 |
| Lrrn4cl | 4,22 | 2,64 | 3,15 | 3,55 | 3,35 | 2,46 | 2,60 | 2,18 | -0,35 | 2,98 | 0,0097 | 0,1016 |
| Mad2l1 | 11,38 | 15,40 | 9,98 | 13,67 | 10,10 | 9,78 | 11,45 | 11,01 | -0,27 | 4,33 | 0,0098 | 0,1028 |
| Cox6a1 | 67,92 | 88,36 | 84,81 | 63,75 | 84,19 | 82,52 | 95,15 | 88,09 | 0,21 | 5,45 | 0,0098 | 0,1032 |
| Rb1 | 25,37 | 22,53 | 22,10 | 24,28 | 21,04 | 21,02 | 21,46 | 20,47 | -0,17 | 6,69 | 0,0099 | 0,1041 |
| Ppp2r5b | 10,41 | 9,72 | 10,76 | 9,81 | 12,50 | 12,58 | 11,32 | 11,20 | 0,23 | 4,91 | 0,0100 | 0,1045 |
| Ngdn | 23,22 | 30,34 | 28,53 | 24,80 | 29,69 | 29,60 | 28,11 | 36,69 | 0,22 | 5,12 | 0,0100 | 0,1045 |
| Casc4 | 10,14 | 9,23 | 8,51 | 8,94 | 8,20 | 8,07 | 8,04 | 7,49 | -0,21 | 5,17 | 0,0100 | 0,1045 |
| Pik3ip1 | 32,42 | 34,74 | 33,86 | 37,51 | 40,70 | 40,27 | 33,64 | 41,40 | 0,17 | 6,37 | 0,0100 | 0,1045 |
| Clasp2 | 15,88 | 14,82 | 15,62 | 16,52 | 14,14 | 13,69 | 14,18 | 13,92 | -0,17 | 6,56 | 0,0101 | 0,1049 |
| Sltm | 27,08 | 29,02 | 29,72 | 24,30 | 30,82 | 30,27 | 28,93 | 33,50 | 0,17 | 6,75 | 0,0101 | 0,1050 |
| Yrdc | 14,45 | 14,13 | 11,20 | 14,20 | 15,23 | 18,03 | 14,11 | 17,03 | 0,24 | 4,54 | 0,0101 | 0,1051 |
| Als2cl | 47,56 | 40,39 | 46,66 | 41,59 | 50,70 | 49,86 | 45,82 | 51,89 | 0,17 | 7,92 | 0,0103 | 0,1064 |
| Nrm | 19,03 | 22,66 | 19,70 | 21,40 | 17,57 | 17,07 | 20,25 | 15,68 | -0,23 | 4,82 | 0,0103 | 0,1067 |
| Etv6 | 17,70 | 17,25 | 14,88 | 17,29 | 20,21 | 17,92 | 18,11 | 19,20 | 0,16 | 6,63 | 0,0104 | 0,1074 |
| Ccnl2 | 73,05 | 69,36 | 80,40 | 66,24 | 81,96 | 82,65 | 77,81 | 82,07 | 0,17 | 7,55 | 0,0104 | 0,1074 |
| Plagl2 | 15,45 | 17,94 | 16,56 | 17,58 | 18,88 | 18,20 | 18,92 | 19,86 | 0,17 | 6,60 | 0,0104 | 0,1075 |
| Zkscan14 | 5,40 | 8,29 | 7,21 | 6,09 | 8,64 | 8,27 | 7,72 | 8,22 | 0,29 | 3,90 | 0,0105 | 0,1078 |
| Gabarap | 101,70 | 107,67 | 101,40 | 96,58 | 123,09 | 113,11 | 109,66 | 110,98 | 0,17 | 6,92 | 0,0105 | 0,1078 |
| Sqstm1 | 97,11 | 103,94 | 98,97 | 102,60 | 112,75 | 110,33 | 111,35 | 118,36 | 0,17 | 7,74 | 0,0105 | 0,1085 |
| Lman1 | 9,17 | 10,77 | 10,37 | 10,38 | 8,11 | 8,86 | 9,38 | 8,66 | -0,22 | 5,08 | 0,0106 | 0,1085 |
| Tmem106a | 2,04 | 2,11 | 1,89 | 1,73 | 1,89 | 1,58 | 1,10 | 1,14 | -0,44 | 1,98 | 0,0106 | 0,1085 |
| Zzef1 | 31,24 | 23,74 | 25,58 | 29,60 | 26,19 | 23,18 | 24,51 | 23,97 | -0,17 | 8,18 | 0,0106 | 0,1088 |
| Vps13a | 31,95 | 23,69 | 23,84 | 26,93 | 23,11 | 22,35 | 24,32 | 24,78 | -0,17 | 8,13 | 0,0107 | 0,1091 |
| Gas7 | 2,81 | 3,43 | 2,61 | 3,27 | 2,42 | 2,34 | 3,03 | 2,38 | -0,26 | 4,32 | 0,0107 | 0,1091 |
| Slc41a1 | 10,86 | 11,72 | 12,60 | 12,11 | 14,04 | 13,46 | 12,43 | 13,69 | 0,18 | 5,88 | 0,0107 | 0,1091 |
| Slc3a2 | 47,26 | 45,57 | 39,13 | 46,14 | 50,84 | 48,16 | 50,63 | 50,30 | 0,16 | 7,06 | 0,0107 | 0,1091 |
| Taok2 | 37,29 | 37,26 | 40,38 | 35,01 | 44,02 | 42,50 | 38,77 | 43,15 | 0,17 | 7,71 | 0,0107 | 0,1097 |
| Cuta | 42,33 | 46,66 | 45,69 | 35,22 | 51,27 | 46,48 | 49,11 | 50,52 | 0,22 | 5,03 | 0,0108 | 0,1098 |
| Aamp | 43,95 | 47,15 | 47,23 | 44,83 | 49,97 | 52,79 | 50,47 | 52,35 | 0,17 | 6,47 | 0,0108 | 0,1102 |
| Nab2 | 14,12 | 14,65 | 18,91 | 14,64 | 16,26 | 19,66 | 16,22 | 19,21 | 0,21 | 5,43 | 0,0108 | 0,1104 |
| Atg7 | 11,99 | 10,90 | 10,03 | 11,49 | 9,91 | 9,77 | 9,68 | 9,28 | -0,21 | 5,35 | 0,0109 | 0,1108 |
| Tmem214 | 15,41 | 14,25 | 17,09 | 14,40 | 18,02 | 17,69 | 17,95 | 16,25 | 0,20 | 5,50 | 0,0110 | 0,1115 |
| Ccr5 | 163,98 | 152,35 | 154,17 | 168,82 | 148,12 | 142,65 | 143,13 | 133,41 | -0,17 | 8,79 | 0,0111 | 0,1121 |
| Nsd1 | 68,50 | 51,06 | 55,69 | 67,95 | 56,93 | 50,56 | 55,37 | 52,94 | -0,17 | 9,52 | 0,0111 | 0,1121 |
| Banf1 | 44,45 | 47,77 | 41,31 | 49,08 | 38,17 | 38,01 | 43,72 | 40,05 | -0,20 | 5,57 | 0,0111 | 0,1123 |
| Gzmk | 151,71 | 174,99 | 171,47 | 135,15 | 155,75 | 136,45 | 147,44 | 124,99 | -0,16 | 6,89 | 0,0112 | 0,1128 |
| Atad2b | 26,30 | 21,01 | 22,05 | 24,66 | 20,68 | 20,28 | 21,97 | 20,91 | -0,17 | 7,49 | 0,0112 | 0,1130 |
| Hist1h4i | 40,73 | 64,61 | 61,81 | 41,00 | 42,30 | 45,78 | 46,42 | 41,46 | -0,23 | 4,68 | 0,0112 | 0,1132 |
| Lta | 2,63 | 2,59 | 2,05 | 1,81 | 3,14 | 3,06 | 3,28 | 2,86 | 0,43 | 1,95 | 0,0112 | 0,1132 |
| Wiz | 14,06 | 16,56 | 15,43 | 14,17 | 17,18 | 16,62 | 16,81 | 17,31 | 0,17 | 6,09 | 0,0113 | 0,1138 |
| Jmjd6 | 15,64 | 20,80 | 20,41 | 18,55 | 21,72 | 20,48 | 20,85 | 24,32 | 0,22 | 5,09 | 0,0113 | 0,1139 |
| Dnajc13 | 28,48 | 24,58 | 25,40 | 28,41 | 23,82 | 22,61 | 24,79 | 24,05 | -0,17 | 7,60 | 0,0114 | 0,1141 |
| Gpi1 | 135,95 | 152,77 | 134,24 | 133,67 | 161,27 | 148,69 | 159,22 | 157,95 | 0,17 | 8,72 | 0,0114 | 0,1141 |
| Hexim1 | 19,16 | 22,54 | 23,36 | 21,55 | 23,18 | 25,31 | 22,79 | 26,02 | 0,17 | 6,29 | 0,0115 | 0,1154 |
| Zdhhc17 | 11,17 | 8,14 | 9,52 | 9,70 | 7,90 | 8,63 | 8,55 | 8,44 | -0,20 | 5,36 | 0,0115 | 0,1155 |
| AA467197 | 3,43 | 2,53 | 1,77 | 1,69 | 3,36 | 4,41 | 3,26 | 2,86 | 0,53 | 1,01 | 0,0115 | 0,1155 |
| Prr12 | 22,95 | 23,58 | 24,64 | 24,04 | 28,74 | 26,67 | 24,59 | 26,68 | 0,17 | 7,44 | 0,0116 | 0,1158 |
| Cdc27 | 21,17 | 20,83 | 19,74 | 21,46 | 19,33 | 17,84 | 19,19 | 17,98 | -0,16 | 6,84 | 0,0116 | 0,1158 |
| Atrnl1 | 1,81 | 1,69 | 1,98 | 1,41 | 1,24 | 1,58 | 1,48 | 1,20 | -0,31 | 3,34 | 0,0116 | 0,1159 |
| D14Abb1e | 35,05 | 28,13 | 28,11 | 32,11 | 27,59 | 27,84 | 27,91 | 26,62 | -0,17 | 7,79 | 0,0117 | 0,1165 |
| Alyref | 20,93 | 28,96 | 26,98 | 21,87 | 29,49 | 25,41 | 27,99 | 32,08 | 0,22 | 4,92 | 0,0117 | 0,1165 |
| Bud31 | 44,37 | 42,82 | 43,60 | 41,31 | 50,47 | 48,66 | 47,98 | 52,70 | 0,22 | 5,04 | 0,0117 | 0,1166 |
| Cx3cr1 | 91,33 | 84,02 | 90,26 | 91,51 | 85,89 | 77,48 | 88,30 | 65,83 | -0,17 | 8,31 | 0,0117 | 0,1166 |
| Nr1h2 | 49,11 | 53,64 | 58,37 | 48,86 | 57,56 | 58,55 | 61,37 | 57,35 | 0,17 | 6,81 | 0,0118 | 0,1167 |
| E2f2 | 31,26 | 38,06 | 33,63 | 31,93 | 29,15 | 30,93 | 30,45 | 29,95 | -0,16 | 7,24 | 0,0118 | 0,1172 |
| Chmp7 | 15,73 | 16,35 | 16,08 | 15,75 | 17,98 | 18,49 | 17,11 | 19,51 | 0,19 | 5,54 | 0,0119 | 0,1173 |
| Nup160 | 10,56 | 10,62 | 10,53 | 10,42 | 9,58 | 9,04 | 9,47 | 9,04 | -0,18 | 5,82 | 0,0119 | 0,1178 |
| Matk | 2,40 | 2,20 | 2,72 | 2,70 | 3,41 | 4,15 | 2,87 | 2,67 | 0,39 | 2,55 | 0,0119 | 0,1179 |
| Tmem203 | 5,97 | 7,94 | 7,76 | 7,03 | 9,84 | 9,15 | 9,19 | 8,39 | 0,36 | 2,93 | 0,0120 | 0,1181 |
| Mfsd10 | 11,65 | 13,49 | 12,47 | 10,57 | 14,69 | 14,01 | 15,28 | 12,84 | 0,24 | 4,59 | 0,0121 | 0,1189 |
| Atp5e | 44,82 | 49,48 | 53,45 | 38,85 | 53,00 | 63,04 | 49,57 | 55,45 | 0,25 | 4,40 | 0,0123 | 0,1206 |
| Mipol1 | 1,65 | 1,25 | 1,30 | 1,21 | 0,98 | 0,94 | 0,94 | 0,82 | -0,56 | 0,79 | 0,0123 | 0,1208 |
| Hist2h2bb | 51,74 | 114,91 | 89,77 | 52,51 | 69,90 | 59,58 | 66,40 | 70,65 | -0,20 | 5,15 | 0,0123 | 0,1209 |
| Tmem115 | 17,61 | 19,16 | 18,46 | 18,15 | 22,79 | 22,12 | 18,75 | 20,45 | 0,20 | 5,43 | 0,0123 | 0,1209 |
| Pde4dip | 3,18 | 3,45 | 3,22 | 3,23 | 3,95 | 3,76 | 3,68 | 3,88 | 0,22 | 4,90 | 0,0123 | 0,1209 |
| Hdgfrp2 | 22,78 | 26,87 | 27,94 | 21,61 | 28,19 | 28,42 | 26,91 | 28,48 | 0,18 | 5,89 | 0,0123 | 0,1209 |
| Chd9 | 9,80 | 8,01 | 7,99 | 9,34 | 8,13 | 7,45 | 8,05 | 7,79 | -0,16 | 6,58 | 0,0124 | 0,1214 |
| Ager | 1,98 | 2,46 | 2,60 | 2,02 | 3,05 | 2,15 | 3,40 | 3,54 | 0,44 | 1,99 | 0,0124 | 0,1214 |
| Bsdc1 | 24,27 | 23,31 | 24,26 | 22,85 | 26,71 | 26,41 | 27,15 | 26,36 | 0,17 | 6,07 | 0,0125 | 0,1216 |
| Ppp1r37 | 16,09 | 14,25 | 16,17 | 14,69 | 17,75 | 17,43 | 17,45 | 17,07 | 0,19 | 5,61 | 0,0125 | 0,1218 |
| Surf2 | 10,73 | 14,67 | 14,82 | 12,00 | 15,60 | 16,45 | 14,51 | 16,20 | 0,27 | 4,04 | 0,0125 | 0,1219 |
| Nsg2 | 13,04 | 13,70 | 13,20 | 12,18 | 11,91 | 14,76 | 12,89 | 21,04 | 0,22 | 4,99 | 0,0125 | 0,1219 |
| Serpinb6a | 10,60 | 8,89 | 9,95 | 9,04 | 7,26 | 9,75 | 6,92 | 7,75 | -0,28 | 3,86 | 0,0126 | 0,1219 |
| Ccdc69 | 19,97 | 21,41 | 22,53 | 17,38 | 23,44 | 25,24 | 21,60 | 24,06 | 0,22 | 4,97 | 0,0126 | 0,1219 |
| Rplp1 | 821,96 | 996,78 | 1117,46 | 851,80 | 1042,80 | 1090,92 | 1034,73 | 1089,89 | 0,17 | 8,88 | 0,0126 | 0,1219 |
| Dync1li2 | 17,20 | 17,55 | 18,80 | 18,01 | 19,87 | 19,90 | 19,26 | 21,06 | 0,16 | 6,46 | 0,0128 | 0,1239 |
| Traf3ip3 | 106,46 | 113,59 | 111,56 | 103,34 | 124,14 | 117,72 | 118,72 | 127,17 | 0,17 | 7,94 | 0,0129 | 0,1249 |
| Slc39a11 | 2,44 | 2,91 | 2,00 | 2,11 | 2,84 | 3,38 | 2,60 | 3,30 | 0,34 | 2,92 | 0,0129 | 0,1251 |
| Pfdn2 | 8,51 | 11,90 | 6,39 | 8,83 | 10,51 | 12,55 | 9,20 | 14,24 | 0,35 | 2,72 | 0,0129 | 0,1251 |
| Zfp457 | 19,56 | 9,89 | 10,28 | 13,44 | 12,79 | 10,19 | 13,02 | 9,47 | -0,23 | 4,72 | 0,0130 | 0,1253 |
| Sharpin | 44,86 | 43,52 | 49,39 | 40,57 | 49,95 | 50,64 | 48,49 | 50,53 | 0,17 | 6,36 | 0,0130 | 0,1255 |
| Ogfr | 41,79 | 49,54 | 44,47 | 45,63 | 50,11 | 50,58 | 47,05 | 55,20 | 0,16 | 6,78 | 0,0130 | 0,1255 |
| Zfp933 | 6,09 | 4,11 | 4,12 | 5,14 | 3,92 | 3,90 | 4,20 | 4,35 | -0,26 | 4,26 | 0,0131 | 0,1256 |
| Ythdf3 | 46,83 | 38,99 | 40,14 | 44,34 | 36,71 | 38,86 | 38,35 | 38,18 | -0,16 | 7,70 | 0,0131 | 0,1260 |
| H2afj | 15,22 | 20,12 | 21,22 | 16,72 | 18,98 | 21,95 | 21,74 | 21,61 | 0,21 | 5,17 | 0,0131 | 0,1260 |
| Atp6v1g1 | 19,32 | 23,81 | 24,57 | 23,01 | 26,49 | 27,47 | 24,67 | 27,48 | 0,23 | 4,76 | 0,0131 | 0,1260 |
| Ssb | 52,17 | 65,53 | 62,28 | 54,34 | 60,11 | 65,85 | 64,49 | 71,32 | 0,16 | 7,02 | 0,0132 | 0,1260 |
| Dync1h1 | 68,48 | 54,39 | 55,94 | 67,94 | 59,40 | 49,55 | 57,94 | 52,65 | -0,17 | 9,71 | 0,0132 | 0,1260 |
| Ahdc1 | 18,64 | 18,34 | 20,18 | 17,28 | 22,06 | 20,54 | 18,78 | 21,73 | 0,16 | 7,02 | 0,0132 | 0,1260 |
| Ndufa4 | 54,12 | 68,93 | 64,58 | 47,54 | 63,05 | 73,11 | 69,95 | 66,57 | 0,22 | 4,97 | 0,0132 | 0,1262 |
| Nfil3 | 2,87 | 2,90 | 1,47 | 2,88 | 3,26 | 2,78 | 3,33 | 3,97 | 0,35 | 2,61 | 0,0132 | 0,1262 |
| Snrpf | 19,47 | 24,77 | 18,03 | 18,94 | 22,54 | 22,00 | 25,80 | 27,08 | 0,25 | 4,20 | 0,0133 | 0,1262 |
| Ensa | 35,38 | 35,01 | 33,37 | 35,85 | 39,30 | 39,84 | 37,10 | 40,23 | 0,16 | 6,40 | 0,0133 | 0,1262 |
| Hdac4 | 11,85 | 11,47 | 11,22 | 12,37 | 10,52 | 10,15 | 10,66 | 9,69 | -0,19 | 5,44 | 0,0133 | 0,1262 |
| Rpl4 | 779,70 | 973,10 | 990,39 | 815,46 | 971,99 | 999,45 | 968,95 | 1059,81 | 0,17 | 10,39 | 0,0133 | 0,1262 |
| Ndufa3 | 56,38 | 73,30 | 58,53 | 51,84 | 71,26 | 69,29 | 70,77 | 72,30 | 0,24 | 4,53 | 0,0134 | 0,1268 |
| Zmynd11 | 30,14 | 31,09 | 33,15 | 27,58 | 33,70 | 32,90 | 34,27 | 35,27 | 0,16 | 7,05 | 0,0135 | 0,1276 |
| Arfgef2 | 14,68 | 13,58 | 14,48 | 15,14 | 12,69 | 12,61 | 13,63 | 12,90 | -0,16 | 6,91 | 0,0136 | 0,1293 |
| Scnm1 | 14,23 | 15,01 | 16,16 | 13,55 | 17,76 | 17,79 | 17,08 | 18,72 | 0,28 | 3,84 | 0,0137 | 0,1293 |
| Dzip3 | 8,24 | 7,04 | 7,98 | 7,87 | 7,02 | 6,70 | 7,11 | 6,34 | -0,19 | 5,39 | 0,0137 | 0,1295 |
| Zfp830 | 7,54 | 7,16 | 6,92 | 7,55 | 8,83 | 7,98 | 8,34 | 9,09 | 0,23 | 4,71 | 0,0137 | 0,1297 |
| Efemp2 | 1,07 | 0,74 | 0,95 | 0,57 | 1,29 | 1,07 | 1,14 | 1,35 | 0,57 | 0,97 | 0,0137 | 0,1297 |
| Marc2 | 23,29 | 26,62 | 22,85 | 22,13 | 27,53 | 25,12 | 26,38 | 29,14 | 0,19 | 5,59 | 0,0137 | 0,1297 |
| Ppm1d | 9,85 | 12,02 | 11,40 | 9,89 | 12,77 | 11,75 | 12,50 | 12,82 | 0,21 | 5,08 | 0,0138 | 0,1297 |
| Mrfap1 | 65,31 | 78,29 | 72,52 | 67,68 | 76,81 | 80,67 | 75,18 | 84,26 | 0,16 | 6,94 | 0,0138 | 0,1297 |
| Pggt1b | 14,99 | 12,95 | 13,90 | 13,64 | 11,48 | 11,43 | 13,04 | 12,15 | -0,20 | 5,15 | 0,0138 | 0,1298 |
| Tep1 | 11,72 | 9,10 | 9,62 | 11,36 | 9,90 | 9,76 | 9,33 | 8,37 | -0,16 | 6,34 | 0,0138 | 0,1298 |
| Ppp1r7 | 13,28 | 11,67 | 12,43 | 12,85 | 10,41 | 10,99 | 12,11 | 10,41 | -0,19 | 5,43 | 0,0138 | 0,1298 |
| Mak16 | 21,97 | 27,89 | 25,67 | 22,31 | 28,22 | 26,40 | 26,97 | 29,71 | 0,19 | 5,61 | 0,0139 | 0,1298 |
| Ap1s3 | 43,26 | 30,43 | 28,38 | 31,80 | 31,31 | 27,19 | 33,20 | 28,23 | -0,16 | 6,51 | 0,0139 | 0,1298 |
| Lsmd1 | 12,18 | 24,03 | 19,72 | 13,06 | 19,52 | 22,95 | 21,39 | 21,58 | 0,32 | 3,34 | 0,0139 | 0,1298 |
| Pou2f1 | 4,80 | 3,73 | 4,25 | 4,65 | 4,14 | 3,47 | 3,98 | 3,79 | -0,18 | 5,74 | 0,0139 | 0,1300 |
| Hnrnph1 | 82,03 | 86,24 | 92,11 | 77,51 | 91,94 | 93,88 | 89,62 | 102,35 | 0,16 | 7,64 | 0,0140 | 0,1306 |
| Plgrkt | 49,98 | 52,03 | 54,97 | 47,46 | 58,23 | 57,55 | 57,03 | 59,98 | 0,19 | 5,53 | 0,0140 | 0,1306 |
| Mnt | 17,66 | 21,59 | 19,65 | 19,07 | 22,38 | 21,86 | 21,80 | 21,09 | 0,16 | 6,57 | 0,0141 | 0,1315 |
| Mphosph10 | 9,06 | 9,39 | 9,88 | 9,17 | 11,27 | 10,98 | 10,91 | 11,03 | 0,24 | 4,50 | 0,0141 | 0,1315 |
| Vav3 | 7,52 | 5,80 | 6,98 | 6,73 | 5,83 | 5,55 | 5,90 | 6,01 | -0,21 | 4,97 | 0,0142 | 0,1324 |
| Hnrnpa1 | 102,74 | 128,24 | 109,87 | 104,82 | 116,61 | 124,43 | 117,28 | 140,67 | 0,16 | 7,86 | 0,0143 | 0,1326 |
| Tubb4b | 57,72 | 74,86 | 64,72 | 64,23 | 58,24 | 52,49 | 61,52 | 61,97 | -0,16 | 6,63 | 0,0143 | 0,1328 |
| Atg16l2 | 26,24 | 25,07 | 32,91 | 25,92 | 32,03 | 32,64 | 29,29 | 29,63 | 0,18 | 5,98 | 0,0144 | 0,1334 |
| Alg6 | 2,42 | 2,42 | 2,03 | 2,02 | 1,67 | 1,91 | 1,57 | 1,76 | -0,37 | 2,64 | 0,0144 | 0,1334 |
| Relt | 11,35 | 13,12 | 12,66 | 12,57 | 14,56 | 14,81 | 12,99 | 14,73 | 0,20 | 5,24 | 0,0144 | 0,1334 |
| Rhof | 69,24 | 69,38 | 73,83 | 66,03 | 83,86 | 77,09 | 76,40 | 73,34 | 0,16 | 7,30 | 0,0144 | 0,1334 |
| Dnm1l | 26,80 | 27,53 | 22,83 | 26,49 | 22,43 | 23,34 | 24,56 | 22,65 | -0,16 | 6,65 | 0,0145 | 0,1338 |
| Fam57a | 2,70 | 3,00 | 2,34 | 2,27 | 2,54 | 1,30 | 1,17 | 2,18 | -0,52 | 1,09 | 0,0146 | 0,1344 |
| Mdh1 | 32,61 | 46,18 | 40,72 | 34,16 | 38,30 | 45,54 | 41,67 | 46,47 | 0,16 | 6,32 | 0,0146 | 0,1344 |
| Dcun1d5 | 31,61 | 33,66 | 39,21 | 32,91 | 38,87 | 38,30 | 38,03 | 41,19 | 0,19 | 5,47 | 0,0146 | 0,1346 |
| Slc7a14 | 4,55 | 2,81 | 2,21 | 3,22 | 2,62 | 2,72 | 2,93 | 2,74 | -0,23 | 4,73 | 0,0147 | 0,1349 |
| Scai | 7,17 | 4,44 | 5,21 | 5,72 | 4,92 | 5,08 | 5,16 | 4,81 | -0,18 | 5,83 | 0,0147 | 0,1349 |
| Eif1 | 316,37 | 325,69 | 280,27 | 333,56 | 340,68 | 330,98 | 356,65 | 380,91 | 0,17 | 8,66 | 0,0147 | 0,1349 |
| Apaf1 | 29,06 | 26,80 | 28,43 | 31,14 | 25,87 | 25,04 | 26,48 | 25,98 | -0,16 | 7,49 | 0,0147 | 0,1350 |
| Hmgn2 | 88,10 | 106,45 | 83,18 | 89,74 | 74,69 | 83,81 | 85,84 | 85,45 | -0,16 | 6,77 | 0,0148 | 0,1353 |
| Rqcd1 | 17,55 | 19,91 | 18,36 | 20,78 | 17,52 | 16,86 | 16,68 | 16,91 | -0,17 | 5,89 | 0,0148 | 0,1356 |
| Hells | 9,19 | 11,82 | 10,34 | 10,72 | 8,85 | 9,03 | 9,55 | 8,82 | -0,22 | 4,89 | 0,0150 | 0,1369 |
| Zc3h6 | 2,09 | 2,52 | 2,58 | 2,16 | 1,94 | 1,99 | 1,81 | 1,80 | -0,30 | 3,38 | 0,0151 | 0,1375 |
| Smco1 | 8,95 | 5,84 | 6,12 | 7,28 | 5,23 | 5,85 | 6,31 | 5,12 | -0,33 | 3,02 | 0,0151 | 0,1377 |
| Zfp934 | 2,08 | 1,38 | 1,82 | 1,82 | 1,41 | 1,40 | 1,38 | 1,06 | -0,43 | 1,81 | 0,0153 | 0,1392 |
| Nop56 | 32,28 | 35,95 | 34,88 | 30,53 | 38,62 | 36,82 | 37,04 | 37,44 | 0,17 | 6,07 | 0,0154 | 0,1400 |
| Gpr183 | 48,69 | 37,93 | 37,29 | 44,93 | 47,62 | 44,69 | 46,73 | 49,36 | 0,16 | 7,04 | 0,0155 | 0,1408 |
| Mctp2 | 27,37 | 20,43 | 21,60 | 23,08 | 21,09 | 20,63 | 21,50 | 19,79 | -0,16 | 6,99 | 0,0155 | 0,1408 |
| Prpsap1 | 25,16 | 27,97 | 27,64 | 24,65 | 30,52 | 29,42 | 28,41 | 30,91 | 0,18 | 5,72 | 0,0156 | 0,1413 |
| Map4k2 | 125,07 | 118,12 | 135,14 | 112,68 | 146,42 | 140,54 | 130,59 | 131,85 | 0,16 | 8,33 | 0,0156 | 0,1416 |
| Phc3 | 32,88 | 22,94 | 24,57 | 29,49 | 25,24 | 22,95 | 26,01 | 24,05 | -0,16 | 8,17 | 0,0156 | 0,1416 |
| Zeb2 | 31,40 | 27,59 | 30,34 | 29,64 | 28,21 | 26,68 | 27,90 | 23,58 | -0,16 | 8,00 | 0,0157 | 0,1419 |
| Cd52 | 763,81 | 976,43 | 937,80 | 744,86 | 982,64 | 961,52 | 964,72 | 925,14 | 0,16 | 8,83 | 0,0157 | 0,1423 |
| Hnrnpu | 90,13 | 127,95 | 124,02 | 99,04 | 117,34 | 120,61 | 122,11 | 134,05 | 0,16 | 8,75 | 0,0157 | 0,1423 |
| Myc | 9,29 | 10,36 | 10,01 | 7,54 | 10,64 | 10,27 | 11,90 | 10,72 | 0,23 | 4,61 | 0,0158 | 0,1427 |
| Bin2 | 272,14 | 316,59 | 323,68 | 265,89 | 327,12 | 331,52 | 321,14 | 340,03 | 0,16 | 9,27 | 0,0158 | 0,1427 |
| H2-K1 | 1505,76 | 1404,31 | 1468,65 | 1486,31 | 1702,01 | 1720,35 | 1531,93 | 1619,39 | 0,16 | 11,32 | 0,0158 | 0,1427 |
| Ikbke | 57,08 | 58,39 | 58,52 | 54,30 | 64,70 | 64,03 | 58,20 | 67,82 | 0,16 | 7,60 | 0,0158 | 0,1427 |
| Grcc10 | 64,32 | 80,64 | 86,73 | 63,39 | 79,14 | 85,92 | 87,20 | 82,72 | 0,19 | 5,48 | 0,0159 | 0,1430 |
| Arl4a | 7,20 | 8,19 | 8,02 | 7,08 | 8,11 | 8,92 | 9,61 | 8,63 | 0,21 | 4,93 | 0,0159 | 0,1433 |
| Rab19 | 23,96 | 26,38 | 24,70 | 25,20 | 28,27 | 27,08 | 29,11 | 30,78 | 0,20 | 5,16 | 0,0160 | 0,1436 |
| Thra | 2,31 | 1,70 | 2,19 | 2,23 | 3,18 | 2,80 | 2,34 | 2,58 | 0,38 | 2,48 | 0,0160 | 0,1436 |
| Irf2 | 56,42 | 67,30 | 62,06 | 55,86 | 66,32 | 66,14 | 68,74 | 68,04 | 0,16 | 7,31 | 0,0160 | 0,1436 |
| Prpf38b | 46,40 | 50,26 | 46,10 | 44,33 | 52,70 | 51,37 | 50,56 | 53,89 | 0,16 | 7,22 | 0,0160 | 0,1436 |
| Cetn3 | 5,96 | 12,67 | 15,03 | 7,45 | 11,43 | 13,78 | 11,03 | 13,53 | 0,31 | 3,52 | 0,0161 | 0,1440 |
| Exosc5 | 15,61 | 15,59 | 16,24 | 14,84 | 16,86 | 21,35 | 18,89 | 17,08 | 0,25 | 4,15 | 0,0161 | 0,1440 |
| Cd46 | 3,00 | 1,58 | 2,88 | 2,47 | 2,20 | 1,04 | 2,43 | 1,35 | -0,46 | 1,39 | 0,0161 | 0,1440 |
| Prcc | 28,52 | 30,39 | 30,08 | 28,28 | 32,78 | 32,22 | 32,04 | 34,63 | 0,17 | 6,00 | 0,0161 | 0,1440 |
| Naca | 45,46 | 50,27 | 52,46 | 42,85 | 51,46 | 54,59 | 50,44 | 57,27 | 0,16 | 8,48 | 0,0162 | 0,1440 |
| H2-Q10 | 57,76 | 44,81 | 50,33 | 53,03 | 62,68 | 60,13 | 53,63 | 53,67 | 0,16 | 6,33 | 0,0163 | 0,1448 |
| Ing2 | 4,98 | 5,14 | 5,58 | 5,11 | 6,68 | 5,48 | 5,92 | 6,79 | 0,26 | 4,02 | 0,0163 | 0,1448 |
| Homez | 1,96 | 2,02 | 2,07 | 2,20 | 1,72 | 1,73 | 1,73 | 1,52 | -0,30 | 3,43 | 0,0164 | 0,1460 |
| Apol7b | 10,59 | 11,51 | 11,85 | 11,30 | 13,00 | 13,19 | 14,21 | 12,22 | 0,22 | 4,78 | 0,0165 | 0,1463 |
| Ppip5k2 | 20,32 | 20,51 | 19,79 | 22,42 | 18,10 | 18,48 | 19,86 | 18,17 | -0,16 | 6,78 | 0,0166 | 0,1468 |
| Rpl22 | 25,40 | 32,38 | 30,29 | 26,23 | 29,03 | 33,86 | 29,63 | 35,56 | 0,17 | 6,08 | 0,0166 | 0,1468 |
| Dcdc2c | 15,47 | 7,96 | 10,42 | 10,67 | 9,30 | 10,29 | 8,99 | 8,42 | -0,27 | 3,87 | 0,0167 | 0,1477 |
| Nop16 | 1,79 | 2,57 | 3,16 | 2,01 | 2,95 | 3,28 | 2,85 | 3,25 | 0,41 | 2,27 | 0,0168 | 0,1483 |
| Appl2 | 5,16 | 5,61 | 6,84 | 5,58 | 7,17 | 6,86 | 6,69 | 6,67 | 0,25 | 4,23 | 0,0168 | 0,1483 |
| Ggt1 | 8,25 | 11,39 | 8,37 | 7,42 | 5,53 | 7,75 | 9,59 | 6,85 | -0,26 | 4,05 | 0,0169 | 0,1488 |
| Pdcd4 | 123,60 | 141,26 | 139,08 | 123,31 | 142,61 | 140,37 | 152,43 | 153,98 | 0,16 | 8,40 | 0,0169 | 0,1494 |
| Elovl1 | 30,49 | 36,17 | 36,90 | 33,30 | 36,54 | 36,93 | 39,80 | 40,00 | 0,17 | 6,04 | 0,0170 | 0,1494 |
| Dnajb11 | 15,45 | 24,14 | 19,29 | 14,38 | 20,54 | 19,44 | 21,23 | 21,71 | 0,18 | 5,63 | 0,0171 | 0,1501 |
| Frmd8 | 70,73 | 70,55 | 75,63 | 75,11 | 83,78 | 82,16 | 75,00 | 84,98 | 0,16 | 7,93 | 0,0172 | 0,1510 |
| Car5b | 14,15 | 16,54 | 14,56 | 14,91 | 13,34 | 12,27 | 13,22 | 14,32 | -0,18 | 5,61 | 0,0172 | 0,1510 |
| Btg1 | 259,91 | 244,34 | 252,00 | 257,44 | 286,41 | 281,54 | 261,24 | 305,28 | 0,16 | 10,39 | 0,0173 | 0,1515 |
| Stag1 | 29,91 | 26,72 | 27,41 | 27,03 | 24,17 | 24,85 | 26,06 | 24,72 | -0,15 | 7,31 | 0,0173 | 0,1515 |
| Ubtf | 47,75 | 52,48 | 57,54 | 49,36 | 59,53 | 60,11 | 53,14 | 58,33 | 0,16 | 8,01 | 0,0173 | 0,1515 |
| Pglyrp1 | 40,67 | 44,04 | 39,99 | 37,37 | 47,64 | 49,34 | 48,00 | 42,64 | 0,21 | 4,90 | 0,0173 | 0,1518 |
| Josd1 | 10,21 | 10,12 | 9,36 | 10,56 | 11,55 | 11,33 | 10,97 | 12,35 | 0,19 | 5,21 | 0,0174 | 0,1518 |
| Lrif1 | 12,47 | 11,59 | 11,88 | 10,08 | 10,14 | 10,00 | 10,07 | 10,03 | -0,19 | 5,29 | 0,0174 | 0,1518 |
| Serinc3 | 137,52 | 133,26 | 125,14 | 140,45 | 116,72 | 114,32 | 127,71 | 120,91 | -0,16 | 8,87 | 0,0174 | 0,1518 |
| Pcf11 | 35,52 | 35,84 | 35,50 | 36,37 | 38,73 | 37,45 | 39,94 | 43,66 | 0,16 | 7,78 | 0,0174 | 0,1518 |
| Slc4a7 | 45,16 | 42,93 | 41,28 | 46,82 | 39,25 | 38,22 | 41,89 | 38,43 | -0,16 | 8,27 | 0,0176 | 0,1530 |
| Ranbp3 | 31,84 | 34,32 | 36,02 | 32,29 | 38,44 | 37,54 | 36,36 | 37,23 | 0,16 | 6,59 | 0,0176 | 0,1530 |
| Cirbp | 21,29 | 24,27 | 21,84 | 21,20 | 18,70 | 18,69 | 18,18 | 20,48 | -0,22 | 4,69 | 0,0176 | 0,1532 |
| Rpl3 | 467,97 | 546,64 | 510,58 | 496,10 | 545,57 | 543,11 | 539,45 | 632,03 | 0,16 | 9,51 | 0,0177 | 0,1538 |
| Nabp2 | 27,60 | 34,94 | 27,33 | 27,64 | 35,25 | 32,42 | 34,40 | 33,30 | 0,20 | 5,07 | 0,0177 | 0,1538 |
| Ap4e1 | 9,94 | 9,41 | 8,48 | 9,86 | 8,26 | 8,61 | 8,50 | 8,20 | -0,17 | 5,86 | 0,0177 | 0,1538 |
| Chmp2a | 37,96 | 50,12 | 45,30 | 35,81 | 47,25 | 47,17 | 50,79 | 47,23 | 0,19 | 5,39 | 0,0178 | 0,1545 |
| Txn2 | 42,34 | 45,38 | 38,95 | 42,38 | 51,55 | 44,60 | 47,28 | 47,00 | 0,17 | 5,85 | 0,0179 | 0,1547 |
| BC005624 | 17,03 | 22,33 | 19,73 | 18,65 | 23,23 | 23,71 | 22,26 | 21,01 | 0,21 | 4,81 | 0,0179 | 0,1549 |
| S100a4 | 257,18 | 337,15 | 293,00 | 241,04 | 287,38 | 311,16 | 339,28 | 316,67 | 0,15 | 7,22 | 0,0181 | 0,1566 |
| Ccdc107 | 5,72 | 8,01 | 9,18 | 6,14 | 8,83 | 8,54 | 8,31 | 10,94 | 0,36 | 2,76 | 0,0182 | 0,1569 |
| Ehd4 | 8,32 | 8,97 | 8,15 | 8,47 | 7,46 | 7,53 | 7,32 | 6,89 | -0,22 | 4,74 | 0,0182 | 0,1570 |
| Polr2l | 6,56 | 10,12 | 10,75 | 7,04 | 10,20 | 10,79 | 10,24 | 9,74 | 0,27 | 3,98 | 0,0182 | 0,1572 |
| Fcgrt | 41,63 | 42,78 | 44,89 | 39,22 | 36,69 | 40,61 | 40,46 | 32,74 | -0,16 | 6,11 | 0,0183 | 0,1575 |
| Zfp36l2 | 353,49 | 406,98 | 391,77 | 339,44 | 402,38 | 396,95 | 410,75 | 457,45 | 0,16 | 10,45 | 0,0183 | 0,1578 |
| Nop14 | 10,11 | 11,54 | 11,17 | 11,98 | 12,47 | 12,64 | 13,39 | 13,05 | 0,20 | 5,03 | 0,0184 | 0,1579 |
| Cmklr1 | 15,21 | 15,83 | 14,73 | 14,84 | 13,20 | 12,18 | 15,21 | 12,61 | -0,19 | 5,34 | 0,0185 | 0,1592 |
| Tbx6 | 5,80 | 6,40 | 6,51 | 5,63 | 8,13 | 7,08 | 6,88 | 7,47 | 0,29 | 3,55 | 0,0186 | 0,1599 |
| Ybey | 2,19 | 2,19 | 2,28 | 2,84 | 1,76 | 1,86 | 2,34 | 1,76 | -0,30 | 3,29 | 0,0187 | 0,1600 |
| Eif3a | 50,60 | 66,02 | 63,09 | 56,45 | 63,48 | 64,62 | 64,83 | 70,56 | 0,16 | 8,34 | 0,0187 | 0,1602 |
| Dbi | 13,35 | 26,53 | 23,44 | 13,73 | 20,46 | 24,72 | 23,20 | 24,53 | 0,28 | 3,66 | 0,0187 | 0,1602 |
| Ubox5 | 4,34 | 3,34 | 4,16 | 4,71 | 3,41 | 3,62 | 4,02 | 2,74 | -0,26 | 3,88 | 0,0188 | 0,1608 |
| Mapk8 | 5,18 | 3,94 | 4,04 | 4,72 | 3,82 | 3,76 | 4,22 | 3,54 | -0,23 | 4,58 | 0,0188 | 0,1608 |
| Cstad | 1,51 | 1,32 | 2,55 | 1,68 | 1,21 | 1,05 | 1,61 | 0,61 | -0,57 | 0,29 | 0,0189 | 0,1615 |
| Spopl | 16,13 | 12,36 | 13,47 | 12,22 | 11,22 | 11,60 | 12,47 | 12,07 | -0,19 | 5,18 | 0,0191 | 0,1627 |
| Timm13 | 12,05 | 13,64 | 15,13 | 10,09 | 14,22 | 14,93 | 14,97 | 16,11 | 0,26 | 4,09 | 0,0191 | 0,1627 |
| Oxct1 | 17,98 | 19,72 | 19,94 | 21,21 | 18,65 | 17,87 | 16,94 | 16,99 | -0,16 | 6,02 | 0,0191 | 0,1628 |
| Spns1 | 16,76 | 17,59 | 18,93 | 17,80 | 20,45 | 21,84 | 17,94 | 19,91 | 0,18 | 5,68 | 0,0192 | 0,1630 |
| Sell | 2,49 | 2,43 | 2,97 | 1,96 | 1,74 | 2,04 | 1,86 | 1,90 | -0,37 | 2,33 | 0,0192 | 0,1630 |
| Pfas | 6,18 | 6,02 | 5,81 | 6,11 | 5,33 | 4,71 | 6,07 | 4,96 | -0,19 | 5,17 | 0,0192 | 0,1633 |
| Ccm2 | 54,58 | 51,31 | 57,70 | 50,99 | 61,84 | 61,53 | 55,72 | 59,10 | 0,15 | 6,73 | 0,0193 | 0,1635 |
| Foxo3 | 26,04 | 24,68 | 26,48 | 28,45 | 31,96 | 26,80 | 31,52 | 27,37 | 0,16 | 6,34 | 0,0193 | 0,1635 |
| Lgals1 | 344,29 | 519,72 | 418,00 | 337,41 | 415,35 | 430,18 | 485,29 | 475,75 | 0,16 | 8,42 | 0,0193 | 0,1635 |
| Grasp | 1,96 | 2,77 | 2,30 | 1,87 | 2,54 | 2,90 | 2,52 | 3,59 | 0,37 | 2,40 | 0,0193 | 0,1635 |
| Sae1 | 23,95 | 26,81 | 25,16 | 23,96 | 26,53 | 27,94 | 28,20 | 29,93 | 0,17 | 5,72 | 0,0194 | 0,1635 |
| Rcbtb2 | 12,70 | 11,18 | 12,43 | 12,37 | 10,90 | 10,30 | 11,37 | 10,07 | -0,19 | 5,28 | 0,0194 | 0,1641 |
| Sf3b4 | 26,81 | 29,60 | 29,54 | 27,02 | 30,68 | 29,66 | 32,48 | 34,58 | 0,18 | 5,68 | 0,0196 | 0,1655 |
| Tada2b | 9,72 | 10,43 | 10,55 | 10,31 | 11,22 | 11,38 | 11,58 | 12,44 | 0,19 | 5,38 | 0,0196 | 0,1655 |
| Zfp459 | 5,70 | 4,71 | 5,23 | 5,01 | 4,78 | 4,01 | 4,72 | 3,77 | -0,25 | 3,98 | 0,0197 | 0,1657 |
| Mxd3 | 4,12 | 7,51 | 5,97 | 5,85 | 3,97 | 4,50 | 5,52 | 4,57 | -0,34 | 2,78 | 0,0197 | 0,1657 |
| Pabpn1 | 75,09 | 88,53 | 81,65 | 79,47 | 91,11 | 88,82 | 86,40 | 94,31 | 0,15 | 7,26 | 0,0197 | 0,1657 |
| Cep89 | 2,75 | 2,96 | 2,84 | 2,46 | 2,10 | 2,28 | 2,28 | 1,99 | -0,35 | 2,67 | 0,0198 | 0,1661 |
| Cd86 | 12,13 | 10,27 | 10,62 | 10,53 | 9,18 | 10,58 | 9,35 | 8,36 | -0,22 | 4,69 | 0,0198 | 0,1663 |
| Psmc5 | 25,08 | 36,38 | 26,14 | 25,80 | 29,35 | 32,03 | 31,22 | 36,96 | 0,19 | 5,29 | 0,0199 | 0,1668 |
| Ifih1 | 12,81 | 11,67 | 11,16 | 11,35 | 10,24 | 10,91 | 10,46 | 10,37 | -0,17 | 5,94 | 0,0199 | 0,1668 |
| H2-Ob | 2,50 | 2,77 | 3,40 | 3,43 | 3,46 | 4,21 | 2,92 | 4,34 | 0,31 | 3,19 | 0,0200 | 0,1668 |
| Clasp1 | 15,00 | 12,76 | 12,13 | 13,55 | 12,56 | 11,54 | 11,92 | 12,15 | -0,15 | 6,64 | 0,0200 | 0,1668 |
| Traf1 | 86,50 | 77,48 | 88,35 | 84,80 | 95,62 | 95,23 | 89,51 | 94,52 | 0,15 | 7,69 | 0,0200 | 0,1668 |
| Id3 | 4,32 | 5,14 | 4,27 | 4,63 | 6,05 | 5,50 | 3,91 | 8,46 | 0,37 | 2,38 | 0,0200 | 0,1668 |
| Srrt | 65,11 | 69,77 | 67,46 | 58,63 | 72,48 | 71,07 | 69,20 | 77,47 | 0,15 | 7,70 | 0,0201 | 0,1675 |
| Pvr | 15,02 | 14,64 | 13,62 | 14,36 | 17,68 | 16,54 | 15,23 | 15,95 | 0,18 | 5,48 | 0,0202 | 0,1681 |
| Chpt1 | 6,17 | 6,61 | 6,28 | 6,92 | 5,51 | 5,60 | 5,83 | 5,13 | -0,24 | 4,29 | 0,0203 | 0,1686 |
| Git1 | 20,80 | 25,00 | 25,34 | 22,49 | 26,70 | 26,25 | 25,03 | 25,94 | 0,15 | 6,50 | 0,0203 | 0,1688 |
| Tnfaip8l2 | 42,46 | 51,10 | 53,44 | 42,83 | 54,68 | 54,96 | 50,66 | 52,52 | 0,17 | 5,82 | 0,0203 | 0,1689 |
| Pld3 | 76,33 | 78,38 | 77,18 | 73,66 | 89,51 | 82,86 | 86,15 | 80,77 | 0,15 | 7,45 | 0,0204 | 0,1692 |
| Rpgrip1l | 1,32 | 1,01 | 1,40 | 1,42 | 0,98 | 0,93 | 1,28 | 0,94 | -0,31 | 3,02 | 0,0205 | 0,1696 |
| Banp | 9,25 | 7,04 | 8,60 | 8,20 | 9,78 | 9,37 | 8,47 | 9,72 | 0,18 | 5,60 | 0,0205 | 0,1696 |
| Lnp | 4,59 | 4,21 | 3,96 | 4,67 | 3,73 | 4,13 | 3,77 | 3,66 | -0,19 | 5,17 | 0,0205 | 0,1696 |
| Ino80d | 27,70 | 19,54 | 20,26 | 26,08 | 22,61 | 19,48 | 21,70 | 20,27 | -0,16 | 8,23 | 0,0205 | 0,1696 |
| Sf1 | 78,87 | 78,39 | 76,40 | 78,95 | 89,84 | 83,66 | 85,60 | 89,38 | 0,16 | 8,49 | 0,0205 | 0,1696 |
| Mrpl2 | 8,14 | 11,38 | 8,69 | 8,43 | 10,80 | 11,00 | 11,62 | 11,49 | 0,29 | 3,39 | 0,0206 | 0,1699 |
| Oxa1l | 21,71 | 27,77 | 26,87 | 24,98 | 28,39 | 29,31 | 28,33 | 27,04 | 0,16 | 6,09 | 0,0206 | 0,1701 |
| Zfp781 | 2,56 | 2,04 | 2,53 | 2,39 | 1,85 | 2,29 | 1,74 | 1,80 | -0,30 | 3,13 | 0,0206 | 0,1701 |
| Tnks1bp1 | 6,07 | 6,28 | 5,46 | 5,65 | 6,99 | 7,09 | 6,03 | 6,71 | 0,19 | 5,18 | 0,0207 | 0,1704 |
| Zfhx2 | 9,83 | 9,76 | 10,67 | 9,05 | 11,80 | 10,25 | 11,17 | 10,37 | 0,15 | 6,50 | 0,0208 | 0,1705 |
| Vegfb | 1,46 | 1,99 | 1,55 | 1,72 | 3,15 | 1,81 | 2,34 | 1,89 | 0,45 | 1,44 | 0,0208 | 0,1705 |
| Acaca | 9,03 | 10,11 | 8,78 | 10,16 | 8,92 | 8,33 | 8,84 | 8,19 | -0,15 | 6,35 | 0,0208 | 0,1705 |
| Ift80 | 9,20 | 8,81 | 8,25 | 10,12 | 7,62 | 7,94 | 7,84 | 8,49 | -0,20 | 5,13 | 0,0208 | 0,1705 |
| Sept11 | 68,59 | 70,39 | 71,34 | 68,98 | 57,98 | 59,26 | 69,18 | 64,28 | -0,16 | 8,37 | 0,0208 | 0,1705 |
| Azin1 | 29,22 | 30,22 | 28,16 | 33,16 | 24,64 | 28,67 | 28,13 | 27,51 | -0,15 | 7,17 | 0,0208 | 0,1705 |
| Tfip11 | 25,45 | 25,79 | 25,79 | 25,66 | 28,59 | 27,85 | 27,82 | 29,73 | 0,15 | 6,59 | 0,0209 | 0,1712 |
| Samm50 | 23,81 | 32,95 | 27,92 | 25,53 | 30,60 | 31,56 | 28,85 | 33,42 | 0,17 | 5,62 | 0,0210 | 0,1719 |
| Gcc2 | 15,24 | 14,31 | 14,55 | 14,08 | 13,20 | 13,05 | 13,39 | 12,76 | -0,15 | 6,49 | 0,0211 | 0,1721 |
| Srsf10 | 17,38 | 17,72 | 17,53 | 18,96 | 19,96 | 18,79 | 20,74 | 20,52 | 0,16 | 6,02 | 0,0211 | 0,1721 |
| Cdc14a | 10,38 | 9,90 | 9,15 | 10,70 | 8,23 | 8,41 | 9,62 | 9,19 | -0,18 | 5,41 | 0,0211 | 0,1723 |
| Slc38a9 | 6,49 | 5,22 | 5,61 | 5,92 | 5,34 | 5,22 | 5,02 | 5,02 | -0,17 | 5,61 | 0,0212 | 0,1726 |
| Smpd1 | 27,93 | 25,56 | 29,37 | 27,55 | 32,38 | 30,83 | 30,46 | 29,31 | 0,16 | 6,14 | 0,0212 | 0,1726 |
| Xpo4 | 14,23 | 10,27 | 9,77 | 12,16 | 9,79 | 10,07 | 11,05 | 9,96 | -0,19 | 5,24 | 0,0212 | 0,1726 |
| Filip1l | 2,18 | 2,25 | 3,04 | 1,83 | 2,93 | 3,24 | 2,38 | 2,72 | 0,31 | 3,35 | 0,0213 | 0,1734 |
| Nat6 | 3,88 | 4,19 | 3,79 | 3,53 | 4,31 | 5,22 | 4,75 | 4,78 | 0,30 | 3,13 | 0,0214 | 0,1736 |
| Klk8 | 26,82 | 30,06 | 27,04 | 25,85 | 34,95 | 31,97 | 27,75 | 30,28 | 0,19 | 5,28 | 0,0214 | 0,1736 |
| Nxf1 | 61,82 | 52,47 | 51,77 | 57,33 | 65,84 | 62,25 | 59,46 | 60,89 | 0,15 | 7,90 | 0,0215 | 0,1742 |
| Tob1 | 32,48 | 37,62 | 38,53 | 36,97 | 39,75 | 42,02 | 39,62 | 40,16 | 0,15 | 6,46 | 0,0215 | 0,1742 |
| Usp9x | 29,40 | 23,00 | 23,15 | 28,65 | 24,19 | 20,77 | 24,35 | 24,39 | -0,15 | 8,20 | 0,0217 | 0,1754 |
| Akap11 | 9,09 | 7,34 | 7,87 | 9,50 | 7,48 | 7,52 | 8,08 | 7,33 | -0,16 | 6,24 | 0,0217 | 0,1754 |
| Sav1 | 13,82 | 11,93 | 14,24 | 13,74 | 15,01 | 15,20 | 14,94 | 16,08 | 0,19 | 5,18 | 0,0217 | 0,1754 |
| Evi2b | 51,76 | 59,37 | 62,17 | 51,92 | 63,40 | 60,87 | 65,43 | 60,31 | 0,15 | 7,71 | 0,0218 | 0,1757 |
| Eef1g | 186,93 | 219,48 | 187,81 | 191,81 | 216,88 | 218,26 | 213,66 | 226,12 | 0,15 | 8,34 | 0,0219 | 0,1766 |
| Rnf44 | 58,80 | 66,97 | 67,86 | 62,28 | 71,34 | 71,23 | 69,98 | 71,93 | 0,15 | 8,08 | 0,0220 | 0,1766 |
| Lrrc58 | 38,93 | 35,39 | 35,92 | 41,71 | 32,66 | 33,60 | 36,31 | 34,04 | -0,15 | 8,28 | 0,0220 | 0,1766 |
| Abhd15 | 1,17 | 1,24 | 1,72 | 1,30 | 1,72 | 1,86 | 1,77 | 1,58 | 0,39 | 2,35 | 0,0220 | 0,1766 |
| Fryl | 110,84 | 89,33 | 89,87 | 107,48 | 94,54 | 83,46 | 89,72 | 89,11 | -0,16 | 10,08 | 0,0220 | 0,1766 |
| Cry1 | 10,90 | 10,84 | 12,42 | 11,36 | 12,14 | 12,36 | 12,66 | 14,65 | 0,19 | 5,20 | 0,0220 | 0,1766 |
| Cpne3 | 52,76 | 50,04 | 52,21 | 59,92 | 48,40 | 48,14 | 49,50 | 47,25 | -0,15 | 8,17 | 0,0221 | 0,1771 |
| Cenpm | 2,71 | 3,66 | 2,43 | 2,12 | 1,57 | 2,13 | 2,15 | 2,30 | -0,44 | 1,56 | 0,0221 | 0,1772 |
| Slc35c1 | 31,01 | 37,11 | 35,59 | 34,00 | 38,51 | 38,29 | 37,20 | 38,56 | 0,15 | 6,83 | 0,0222 | 0,1775 |
| Rbm41 | 10,80 | 9,05 | 8,77 | 9,26 | 8,79 | 8,41 | 8,25 | 8,21 | -0,17 | 5,65 | 0,0222 | 0,1779 |
| Fam210a | 5,40 | 4,19 | 5,02 | 5,63 | 4,57 | 4,06 | 4,60 | 4,67 | -0,18 | 5,53 | 0,0223 | 0,1781 |
| Loxl2 | 2,47 | 1,66 | 1,81 | 2,20 | 1,80 | 1,53 | 1,63 | 1,69 | -0,29 | 3,27 | 0,0223 | 0,1781 |
| Crtc3 | 16,28 | 18,36 | 19,28 | 18,47 | 20,06 | 19,78 | 19,92 | 20,46 | 0,15 | 6,61 | 0,0223 | 0,1781 |
| BC055324 | 1,14 | 1,75 | 1,23 | 1,60 | 1,06 | 0,95 | 1,53 | 0,83 | -0,40 | 1,92 | 0,0224 | 0,1781 |
| Wrn | 24,93 | 21,90 | 21,60 | 23,39 | 20,15 | 20,02 | 21,40 | 21,36 | -0,15 | 7,12 | 0,0224 | 0,1781 |
| Chd3 | 126,97 | 120,88 | 133,90 | 125,85 | 150,30 | 139,40 | 132,01 | 143,58 | 0,16 | 9,93 | 0,0224 | 0,1781 |
| Vps37a | 14,90 | 12,05 | 13,00 | 14,27 | 12,43 | 12,37 | 11,95 | 12,08 | -0,15 | 6,36 | 0,0224 | 0,1781 |
| Ddx3x | 65,84 | 71,14 | 63,12 | 67,89 | 72,40 | 70,76 | 74,16 | 80,86 | 0,15 | 8,34 | 0,0224 | 0,1783 |
| Zdhhc9 | 6,53 | 7,31 | 7,86 | 6,04 | 7,64 | 8,19 | 8,02 | 8,42 | 0,23 | 4,50 | 0,0225 | 0,1786 |
| Kpna2 | 34,03 | 40,98 | 34,76 | 36,38 | 32,55 | 28,87 | 36,27 | 33,62 | -0,16 | 6,15 | 0,0225 | 0,1786 |
| Gimap6 | 344,85 | 363,66 | 350,94 | 346,12 | 389,84 | 375,57 | 386,59 | 412,73 | 0,15 | 9,23 | 0,0227 | 0,1798 |
| Tmc4 | 1,66 | 1,84 | 1,29 | 1,99 | 2,11 | 2,13 | 2,09 | 2,55 | 0,36 | 2,26 | 0,0227 | 0,1798 |
| Emp1 | 25,40 | 28,26 | 24,18 | 27,04 | 20,37 | 20,72 | 28,94 | 24,26 | -0,16 | 6,14 | 0,0227 | 0,1799 |
| Nudt4 | 24,86 | 25,89 | 27,98 | 27,45 | 22,78 | 23,61 | 25,42 | 23,73 | -0,15 | 6,35 | 0,0228 | 0,1799 |
| Trappc9 | 8,59 | 6,48 | 6,33 | 7,61 | 6,26 | 6,47 | 6,58 | 6,08 | -0,20 | 5,00 | 0,0228 | 0,1799 |
| Med13 | 53,64 | 39,56 | 39,70 | 51,23 | 43,81 | 38,46 | 43,21 | 39,98 | -0,15 | 9,01 | 0,0228 | 0,1799 |
| Tbc1d7 | 4,07 | 4,47 | 4,35 | 4,47 | 3,48 | 2,64 | 4,07 | 3,26 | -0,36 | 2,30 | 0,0228 | 0,1799 |
| Rab3ip | 8,29 | 9,66 | 7,91 | 8,91 | 9,68 | 10,54 | 9,16 | 11,03 | 0,21 | 4,67 | 0,0228 | 0,1799 |
| Uchl5 | 5,98 | 8,02 | 8,74 | 6,41 | 8,62 | 7,97 | 9,20 | 8,79 | 0,26 | 3,89 | 0,0228 | 0,1799 |
| Sart3 | 15,64 | 15,58 | 14,95 | 15,33 | 17,92 | 17,34 | 16,19 | 17,42 | 0,16 | 5,87 | 0,0230 | 0,1810 |
| Gigyf2 | 19,69 | 20,29 | 20,22 | 19,18 | 22,76 | 21,45 | 20,70 | 22,97 | 0,15 | 6,91 | 0,0231 | 0,1814 |
| Tbl1xr1 | 69,35 | 66,89 | 64,21 | 79,10 | 63,43 | 60,58 | 64,98 | 62,25 | -0,15 | 9,08 | 0,0231 | 0,1814 |
| Phldb3 | 6,19 | 6,96 | 7,25 | 6,77 | 8,37 | 8,12 | 7,74 | 7,85 | 0,24 | 4,09 | 0,0231 | 0,1814 |
| Slc7a6os | 16,66 | 19,18 | 17,12 | 14,57 | 19,81 | 19,69 | 18,93 | 19,46 | 0,21 | 4,80 | 0,0232 | 0,1816 |
| Phf6 | 19,14 | 17,78 | 18,41 | 20,79 | 16,55 | 16,11 | 17,70 | 18,20 | -0,15 | 6,30 | 0,0232 | 0,1816 |
| Cep192 | 24,39 | 22,52 | 21,82 | 23,77 | 22,64 | 19,52 | 21,03 | 20,30 | -0,15 | 7,48 | 0,0232 | 0,1818 |
| Zfp276 | 9,67 | 9,14 | 10,59 | 11,01 | 12,22 | 11,53 | 10,46 | 11,16 | 0,17 | 5,71 | 0,0233 | 0,1818 |
| Ptpn4 | 51,44 | 43,70 | 46,44 | 52,98 | 46,59 | 43,67 | 43,37 | 41,73 | -0,15 | 7,77 | 0,0233 | 0,1818 |
| Lpcat4 | 46,08 | 46,04 | 50,86 | 42,64 | 52,12 | 51,36 | 50,51 | 51,45 | 0,15 | 6,54 | 0,0233 | 0,1820 |
| Ccdc15 | 2,76 | 2,53 | 3,70 | 2,04 | 2,09 | 2,02 | 2,38 | 2,36 | -0,29 | 3,11 | 0,0233 | 0,1820 |
| Dram2 | 10,35 | 8,14 | 8,03 | 9,84 | 7,92 | 7,57 | 8,12 | 7,71 | -0,22 | 4,53 | 0,0233 | 0,1820 |
| Snrnp25 | 4,43 | 6,71 | 5,23 | 6,19 | 7,50 | 6,84 | 6,55 | 8,09 | 0,35 | 2,47 | 0,0234 | 0,1820 |
| Acd | 18,76 | 18,74 | 16,79 | 15,97 | 20,17 | 20,86 | 19,98 | 20,24 | 0,21 | 4,76 | 0,0234 | 0,1820 |
| Vipr1 | 5,52 | 4,77 | 4,86 | 4,41 | 6,14 | 5,47 | 5,24 | 5,78 | 0,21 | 4,70 | 0,0234 | 0,1822 |
| Pin4 | 7,43 | 9,39 | 8,36 | 7,33 | 10,05 | 9,44 | 11,18 | 11,46 | 0,38 | 2,19 | 0,0236 | 0,1834 |
| Btaf1 | 15,81 | 13,43 | 13,73 | 14,38 | 13,34 | 12,61 | 12,60 | 13,28 | -0,15 | 6,86 | 0,0236 | 0,1835 |
| Fan1 | 4,19 | 3,36 | 3,16 | 3,67 | 3,16 | 3,28 | 2,94 | 2,55 | -0,28 | 3,53 | 0,0237 | 0,1835 |
| Rmdn1 | 3,56 | 2,14 | 3,38 | 2,75 | 2,39 | 2,34 | 2,52 | 1,87 | -0,36 | 2,30 | 0,0237 | 0,1835 |
| Pibf1 | 6,65 | 4,24 | 5,13 | 4,50 | 4,12 | 4,84 | 4,57 | 3,74 | -0,25 | 3,96 | 0,0237 | 0,1835 |
| Mpi | 4,11 | 3,85 | 3,71 | 3,74 | 5,29 | 5,73 | 3,51 | 4,65 | 0,31 | 2,95 | 0,0237 | 0,1835 |
| Ccne2 | 1,94 | 3,07 | 1,64 | 2,13 | 1,92 | 1,86 | 1,81 | 1,43 | -0,35 | 2,65 | 0,0241 | 0,1860 |
| Taf7 | 12,29 | 11,92 | 11,28 | 11,72 | 12,17 | 13,20 | 14,23 | 13,90 | 0,18 | 5,38 | 0,0241 | 0,1861 |
| Osbpl3 | 21,43 | 19,44 | 18,28 | 20,82 | 18,02 | 19,23 | 19,36 | 15,73 | -0,15 | 7,01 | 0,0241 | 0,1863 |
| Park7 | 27,71 | 46,84 | 39,17 | 33,99 | 39,40 | 41,51 | 44,25 | 42,95 | 0,19 | 5,16 | 0,0243 | 0,1871 |
| Nfat5 | 27,66 | 17,44 | 20,30 | 21,69 | 20,45 | 19,04 | 19,77 | 19,21 | -0,15 | 8,12 | 0,0243 | 0,1874 |
| Sun1 | 23,79 | 27,02 | 23,29 | 26,01 | 21,77 | 22,64 | 24,22 | 21,91 | -0,15 | 6,62 | 0,0244 | 0,1875 |
| Atl3 | 34,86 | 33,86 | 32,61 | 39,00 | 31,60 | 30,96 | 31,55 | 32,51 | -0,15 | 7,76 | 0,0244 | 0,1875 |
| Rnf26 | 13,70 | 15,02 | 14,37 | 14,98 | 12,62 | 12,25 | 13,01 | 12,81 | -0,20 | 4,95 | 0,0244 | 0,1875 |
| Slc25a4 | 59,23 | 72,00 | 68,41 | 62,60 | 71,81 | 70,88 | 70,03 | 78,37 | 0,15 | 6,26 | 0,0244 | 0,1876 |
| Pspc1 | 18,37 | 14,65 | 13,94 | 12,57 | 12,01 | 13,30 | 13,23 | 13,64 | -0,19 | 5,05 | 0,0245 | 0,1880 |
| Erc1 | 2,98 | 2,60 | 2,49 | 2,49 | 2,05 | 2,25 | 2,62 | 2,14 | -0,22 | 4,43 | 0,0246 | 0,1883 |
| Qprt | 15,59 | 10,89 | 13,19 | 13,80 | 11,32 | 11,74 | 11,57 | 10,29 | -0,25 | 3,88 | 0,0246 | 0,1883 |
| Polh | 12,12 | 14,47 | 13,34 | 13,95 | 12,09 | 11,83 | 11,63 | 11,62 | -0,19 | 5,03 | 0,0247 | 0,1889 |
| Ndufb11 | 41,19 | 47,46 | 46,68 | 35,98 | 46,05 | 50,20 | 45,93 | 51,85 | 0,18 | 5,29 | 0,0247 | 0,1889 |
| Tspan32 | 19,86 | 16,59 | 20,80 | 21,77 | 15,70 | 17,88 | 21,01 | 14,32 | -0,19 | 4,97 | 0,0249 | 0,1897 |
| Ggh | 12,98 | 12,45 | 11,55 | 12,94 | 9,38 | 11,36 | 10,99 | 10,45 | -0,25 | 3,94 | 0,0249 | 0,1897 |
| Prkce | 4,98 | 3,31 | 3,66 | 4,10 | 3,63 | 3,45 | 3,71 | 3,05 | -0,22 | 4,55 | 0,0249 | 0,1897 |
| Rpl36al | 126,08 | 160,86 | 156,40 | 128,98 | 149,96 | 156,11 | 162,29 | 166,03 | 0,15 | 6,29 | 0,0249 | 0,1897 |
| Tmem168 | 17,62 | 14,82 | 15,87 | 16,87 | 13,89 | 15,36 | 15,39 | 14,00 | -0,15 | 6,14 | 0,0249 | 0,1897 |
| Btla | 10,79 | 10,15 | 9,88 | 9,11 | 8,38 | 8,24 | 9,25 | 8,96 | -0,20 | 4,92 | 0,0250 | 0,1906 |
| Arhgef39 | 0,96 | 1,39 | 1,40 | 1,84 | 2,15 | 1,52 | 1,74 | 2,20 | 0,44 | 1,50 | 0,0251 | 0,1906 |
| Ddx5 | 400,26 | 422,64 | 449,26 | 426,46 | 465,31 | 468,88 | 468,14 | 485,85 | 0,15 | 10,62 | 0,0251 | 0,1910 |
| Pskh1 | 13,48 | 14,52 | 14,22 | 15,57 | 13,21 | 12,84 | 14,04 | 11,13 | -0,17 | 5,47 | 0,0251 | 0,1910 |
| Odc1 | 40,96 | 42,35 | 34,01 | 39,27 | 42,69 | 44,31 | 44,55 | 41,94 | 0,14 | 6,72 | 0,0252 | 0,1910 |
| Tagap | 39,57 | 47,78 | 41,51 | 43,23 | 46,26 | 44,68 | 48,41 | 51,00 | 0,14 | 7,12 | 0,0252 | 0,1911 |
| Polr2j | 23,63 | 33,45 | 35,67 | 20,50 | 32,75 | 29,49 | 35,95 | 34,11 | 0,24 | 4,17 | 0,0253 | 0,1920 |
| Hid1 | 61,83 | 56,86 | 61,29 | 60,29 | 74,65 | 66,50 | 63,03 | 61,88 | 0,15 | 7,68 | 0,0254 | 0,1925 |
| Slc38a2 | 53,01 | 57,32 | 50,04 | 56,96 | 58,32 | 56,77 | 56,25 | 69,77 | 0,15 | 8,05 | 0,0254 | 0,1925 |
| Gnpda1 | 32,76 | 30,51 | 32,56 | 34,00 | 29,22 | 30,71 | 30,58 | 26,33 | -0,15 | 6,15 | 0,0255 | 0,1930 |
| Afg3l2 | 17,94 | 17,40 | 16,58 | 17,08 | 19,71 | 19,52 | 19,03 | 18,98 | 0,16 | 5,81 | 0,0256 | 0,1931 |
| Phax | 20,34 | 25,76 | 22,78 | 19,27 | 24,16 | 23,90 | 24,94 | 26,40 | 0,17 | 5,48 | 0,0256 | 0,1931 |
| Tmem248 | 19,37 | 18,75 | 18,04 | 19,66 | 21,17 | 20,32 | 21,09 | 21,68 | 0,15 | 6,20 | 0,0256 | 0,1931 |
| Ndufa1 | 31,02 | 43,94 | 39,52 | 27,55 | 38,66 | 42,41 | 39,14 | 45,96 | 0,23 | 4,25 | 0,0256 | 0,1931 |
| Arhgap27 | 25,60 | 25,62 | 26,88 | 25,39 | 29,07 | 30,83 | 26,86 | 27,59 | 0,14 | 6,96 | 0,0256 | 0,1931 |
| Slc26a2 | 7,68 | 7,48 | 7,81 | 8,14 | 6,72 | 6,64 | 7,58 | 6,11 | -0,20 | 4,81 | 0,0257 | 0,1935 |
| Dnajc21 | 9,27 | 9,69 | 10,00 | 9,34 | 9,79 | 11,75 | 10,86 | 12,22 | 0,22 | 4,42 | 0,0258 | 0,1937 |
| Hace1 | 12,55 | 9,81 | 10,94 | 13,17 | 10,03 | 10,37 | 10,72 | 10,04 | -0,18 | 5,38 | 0,0258 | 0,1937 |
| Zbtb18 | 2,56 | 2,52 | 2,77 | 3,24 | 3,68 | 3,57 | 2,83 | 3,10 | 0,25 | 3,92 | 0,0258 | 0,1937 |
| Chrac1 | 9,61 | 11,29 | 12,11 | 9,16 | 14,00 | 13,02 | 12,16 | 11,86 | 0,29 | 3,33 | 0,0258 | 0,1937 |
| Cenpa | 38,03 | 41,93 | 37,56 | 43,09 | 34,21 | 34,33 | 38,41 | 36,37 | -0,17 | 5,67 | 0,0260 | 0,1943 |
| Mrs2 | 22,76 | 21,00 | 22,01 | 23,46 | 19,71 | 19,91 | 21,32 | 18,27 | -0,17 | 5,51 | 0,0260 | 0,1943 |
| Ipo11 | 8,33 | 7,40 | 7,86 | 8,16 | 7,12 | 7,55 | 7,03 | 6,09 | -0,19 | 5,00 | 0,0260 | 0,1943 |
| Jtb | 36,80 | 41,28 | 38,58 | 37,48 | 44,47 | 43,53 | 39,19 | 46,56 | 0,17 | 5,50 | 0,0260 | 0,1943 |
| Kif23 | 9,30 | 14,09 | 11,60 | 10,09 | 9,30 | 8,84 | 11,25 | 10,28 | -0,18 | 5,18 | 0,0260 | 0,1943 |
| Wnk1 | 101,10 | 83,56 | 81,92 | 98,47 | 86,39 | 76,80 | 84,76 | 80,77 | -0,15 | 9,94 | 0,0261 | 0,1944 |
| Mlxip | 24,52 | 23,51 | 23,02 | 23,59 | 26,43 | 24,72 | 25,96 | 27,59 | 0,15 | 7,51 | 0,0261 | 0,1945 |
| Fam177a | 9,76 | 12,30 | 10,44 | 10,11 | 11,59 | 10,99 | 11,48 | 14,05 | 0,17 | 5,45 | 0,0261 | 0,1946 |
| Dxo | 15,55 | 14,82 | 14,33 | 14,12 | 17,71 | 16,05 | 17,58 | 16,87 | 0,21 | 4,56 | 0,0262 | 0,1948 |
| Sirt2 | 34,76 | 37,62 | 37,77 | 32,06 | 39,56 | 40,08 | 38,21 | 39,99 | 0,15 | 6,13 | 0,0262 | 0,1949 |
| Fam129a | 54,61 | 56,66 | 54,04 | 56,83 | 51,36 | 49,64 | 51,05 | 48,64 | -0,15 | 7,70 | 0,0262 | 0,1951 |
| Gpr174 | 36,23 | 30,57 | 32,34 | 36,33 | 30,85 | 31,64 | 30,09 | 30,01 | -0,14 | 7,39 | 0,0263 | 0,1951 |
| Zfp182 | 11,75 | 8,35 | 9,48 | 9,82 | 8,34 | 9,54 | 9,24 | 8,13 | -0,16 | 5,79 | 0,0263 | 0,1952 |
| Stard10 | 23,75 | 23,32 | 27,23 | 21,04 | 27,87 | 27,46 | 27,63 | 25,81 | 0,20 | 4,94 | 0,0264 | 0,1959 |
| Scrn3 | 3,00 | 2,79 | 3,03 | 3,01 | 2,33 | 2,53 | 2,46 | 2,32 | -0,29 | 3,13 | 0,0264 | 0,1960 |
| Creb1 | 35,20 | 32,35 | 33,40 | 36,76 | 30,16 | 30,36 | 32,45 | 31,30 | -0,15 | 8,11 | 0,0265 | 0,1961 |
| Polr2i | 10,07 | 13,57 | 11,38 | 8,17 | 13,91 | 14,97 | 11,78 | 12,48 | 0,30 | 3,08 | 0,0265 | 0,1962 |
| Tmem251 | 12,82 | 11,92 | 15,54 | 11,72 | 14,46 | 16,01 | 12,89 | 17,61 | 0,24 | 4,09 | 0,0266 | 0,1965 |
| Tbc1d8b | 10,85 | 9,88 | 9,61 | 10,56 | 8,94 | 9,10 | 8,83 | 9,79 | -0,16 | 5,84 | 0,0267 | 0,1969 |
| Hmgn1 | 14,13 | 19,40 | 17,81 | 13,77 | 20,33 | 19,06 | 15,89 | 20,41 | 0,22 | 4,41 | 0,0267 | 0,1971 |
| Ube2v2 | 7,61 | 6,56 | 8,03 | 7,47 | 6,44 | 6,84 | 6,63 | 6,29 | -0,17 | 5,36 | 0,0267 | 0,1971 |
| Mbd3 | 32,94 | 40,83 | 43,80 | 34,56 | 38,61 | 45,37 | 41,74 | 43,25 | 0,16 | 6,00 | 0,0268 | 0,1971 |
| Pea15a | 39,01 | 38,76 | 41,04 | 39,25 | 45,30 | 41,39 | 42,36 | 45,52 | 0,14 | 6,69 | 0,0268 | 0,1972 |
| Herc4 | 47,72 | 41,73 | 41,26 | 45,69 | 40,92 | 39,75 | 39,44 | 39,60 | -0,14 | 7,41 | 0,0269 | 0,1977 |
| Atp10d | 34,47 | 27,99 | 26,84 | 29,75 | 27,08 | 27,34 | 28,19 | 25,20 | -0,14 | 7,42 | 0,0270 | 0,1985 |
| Hinfp | 8,66 | 11,29 | 11,08 | 10,81 | 12,03 | 12,07 | 11,78 | 12,53 | 0,21 | 4,57 | 0,0271 | 0,1985 |
| Atp2b1 | 85,11 | 80,06 | 78,53 | 86,40 | 71,87 | 71,74 | 81,04 | 72,86 | -0,15 | 9,13 | 0,0271 | 0,1985 |
| Troap | 1,75 | 3,07 | 2,63 | 1,88 | 1,29 | 1,87 | 2,04 | 2,03 | -0,36 | 2,24 | 0,0271 | 0,1985 |
| Bloc1s2 | 18,36 | 19,94 | 20,77 | 20,33 | 22,16 | 23,48 | 22,68 | 24,49 | 0,23 | 4,28 | 0,0272 | 0,1995 |
| Gnb4 | 2,31 | 1,80 | 2,13 | 2,60 | 1,58 | 1,89 | 1,90 | 1,60 | -0,35 | 2,46 | 0,0272 | 0,1995 |
| Pja1 | 11,94 | 11,56 | 12,12 | 11,51 | 12,59 | 13,27 | 14,12 | 13,63 | 0,19 | 5,09 | 0,0275 | 0,2009 |
| Hivep2 | 43,82 | 37,00 | 38,51 | 45,58 | 38,97 | 34,24 | 39,34 | 36,16 | -0,15 | 8,58 | 0,0275 | 0,2009 |
| Phf12 | 30,44 | 31,16 | 31,33 | 29,40 | 34,84 | 33,99 | 32,73 | 33,46 | 0,14 | 7,14 | 0,0275 | 0,2011 |
| Tomm7 | 19,72 | 30,89 | 26,69 | 17,73 | 26,65 | 29,89 | 24,32 | 28,02 | 0,20 | 4,83 | 0,0276 | 0,2012 |
| Rrp9 | 7,10 | 8,51 | 8,49 | 7,15 | 8,20 | 10,46 | 9,16 | 9,43 | 0,26 | 3,73 | 0,0276 | 0,2013 |
| Dtwd1 | 2,63 | 3,28 | 2,96 | 2,37 | 1,95 | 1,99 | 2,27 | 2,28 | -0,40 | 1,75 | 0,0276 | 0,2013 |
| Rad54l | 1,81 | 3,00 | 1,81 | 2,47 | 1,73 | 1,41 | 2,27 | 1,90 | -0,33 | 2,69 | 0,0277 | 0,2021 |
| Mvb12a | 25,83 | 32,95 | 34,46 | 30,06 | 32,13 | 36,43 | 35,18 | 36,10 | 0,19 | 5,13 | 0,0278 | 0,2021 |
| Rps3a1 | 598,17 | 699,45 | 686,76 | 597,03 | 705,73 | 706,76 | 689,93 | 760,35 | 0,15 | 9,33 | 0,0278 | 0,2021 |
| Ehd3 | 65,46 | 67,27 | 68,56 | 67,64 | 74,50 | 72,06 | 73,09 | 78,04 | 0,15 | 8,02 | 0,0279 | 0,2021 |
| Magi3 | 2,70 | 2,23 | 2,48 | 2,13 | 1,92 | 2,06 | 2,02 | 2,05 | -0,24 | 3,87 | 0,0279 | 0,2021 |
| Ctc1 | 3,44 | 3,98 | 3,09 | 3,58 | 3,09 | 2,73 | 3,20 | 2,84 | -0,26 | 3,77 | 0,0279 | 0,2021 |
| Cct8 | 54,34 | 65,20 | 58,38 | 59,78 | 62,30 | 64,79 | 65,32 | 70,06 | 0,14 | 7,23 | 0,0279 | 0,2021 |
| Mrpl48 | 8,02 | 10,35 | 8,19 | 8,44 | 10,63 | 11,38 | 10,31 | 10,50 | 0,28 | 3,23 | 0,0281 | 0,2034 |
| Gimap5 | 66,41 | 70,92 | 69,75 | 69,90 | 76,99 | 76,26 | 75,10 | 77,30 | 0,14 | 7,15 | 0,0282 | 0,2041 |
| Tmem259 | 38,97 | 40,25 | 41,52 | 36,00 | 44,51 | 43,92 | 41,48 | 43,06 | 0,14 | 6,52 | 0,0283 | 0,2047 |
| Ube2m | 39,89 | 50,75 | 55,50 | 39,30 | 52,19 | 52,48 | 49,12 | 51,77 | 0,16 | 5,98 | 0,0283 | 0,2047 |
| Atp11c | 13,10 | 11,84 | 11,46 | 13,08 | 11,31 | 10,85 | 11,32 | 11,19 | -0,15 | 6,16 | 0,0284 | 0,2048 |
| Chkb | 22,27 | 20,60 | 22,87 | 21,54 | 25,40 | 25,49 | 22,20 | 25,37 | 0,18 | 5,35 | 0,0284 | 0,2049 |
| N4bp2l2 | 20,79 | 14,48 | 14,06 | 17,80 | 15,67 | 14,83 | 15,89 | 14,51 | -0,14 | 7,17 | 0,0284 | 0,2050 |
| Chpf2 | 22,30 | 20,50 | 27,68 | 22,64 | 25,35 | 26,99 | 23,95 | 26,29 | 0,15 | 6,51 | 0,0286 | 0,2059 |
| Klhdc3 | 31,96 | 34,22 | 32,53 | 33,73 | 37,21 | 36,08 | 35,53 | 37,97 | 0,15 | 6,22 | 0,0286 | 0,2062 |
| Cd209c | 2,41 | 1,45 | 1,10 | 1,52 | 1,53 | 1,07 | 1,44 | 0,77 | -0,45 | 1,21 | 0,0287 | 0,2062 |
| Lamc1 | 15,46 | 16,01 | 14,79 | 16,60 | 14,73 | 13,61 | 14,45 | 14,24 | -0,14 | 6,84 | 0,0287 | 0,2064 |
| Slc16a6 | 14,94 | 10,75 | 12,09 | 12,93 | 11,12 | 10,55 | 12,33 | 11,29 | -0,16 | 5,66 | 0,0287 | 0,2064 |
| Ifi27l2a | 31,84 | 38,98 | 43,94 | 36,35 | 32,67 | 40,00 | 31,88 | 25,59 | -0,21 | 4,54 | 0,0288 | 0,2064 |
| Cnot4 | 13,75 | 10,71 | 10,05 | 11,39 | 10,39 | 9,96 | 11,23 | 9,75 | -0,16 | 5,94 | 0,0290 | 0,2074 |
| Rps5 | 555,46 | 605,76 | 600,26 | 537,05 | 622,52 | 626,41 | 637,95 | 660,20 | 0,15 | 8,81 | 0,0290 | 0,2074 |
| Zbtb7b | 18,68 | 18,73 | 19,68 | 17,00 | 21,16 | 21,44 | 20,29 | 19,13 | 0,15 | 6,17 | 0,0290 | 0,2074 |
| Heatr2 | 12,84 | 10,57 | 13,95 | 12,47 | 10,83 | 10,46 | 12,49 | 10,22 | -0,17 | 5,33 | 0,0290 | 0,2074 |
| Nol12 | 4,78 | 6,49 | 5,84 | 5,26 | 7,25 | 6,02 | 7,13 | 6,33 | 0,26 | 3,63 | 0,0290 | 0,2074 |
| Map3k7 | 20,71 | 20,68 | 19,42 | 21,57 | 18,97 | 17,99 | 20,27 | 17,51 | -0,14 | 6,82 | 0,0290 | 0,2074 |
| Samd4b | 15,65 | 17,42 | 17,47 | 16,62 | 19,40 | 17,76 | 19,05 | 18,06 | 0,15 | 6,28 | 0,0291 | 0,2077 |
| Tet2 | 19,51 | 15,35 | 15,18 | 17,10 | 15,73 | 15,29 | 15,66 | 14,24 | -0,14 | 7,20 | 0,0292 | 0,2080 |
| Arhgap33 | 1,19 | 1,73 | 1,72 | 1,25 | 1,35 | 1,09 | 1,08 | 1,11 | -0,33 | 2,54 | 0,0292 | 0,2081 |
| Narfl | 6,17 | 6,29 | 6,59 | 6,22 | 7,54 | 7,12 | 7,93 | 7,01 | 0,23 | 4,14 | 0,0293 | 0,2083 |
| Tnpo2 | 24,68 | 25,13 | 25,89 | 26,40 | 28,20 | 27,21 | 27,14 | 30,04 | 0,14 | 7,04 | 0,0293 | 0,2083 |
| Ip6k2 | 13,82 | 16,50 | 15,56 | 12,38 | 17,56 | 16,38 | 15,90 | 16,76 | 0,20 | 4,85 | 0,0293 | 0,2087 |
| Evl | 147,48 | 161,68 | 152,84 | 152,92 | 177,76 | 166,42 | 165,34 | 171,44 | 0,15 | 8,47 | 0,0294 | 0,2089 |
| Itpr2 | 33,81 | 24,06 | 26,08 | 27,50 | 26,63 | 24,05 | 25,89 | 24,14 | -0,15 | 8,29 | 0,0294 | 0,2089 |
| Snx10 | 25,79 | 26,17 | 26,45 | 24,35 | 21,49 | 24,40 | 24,37 | 22,16 | -0,15 | 5,97 | 0,0295 | 0,2092 |
| Scfd2 | 9,69 | 6,79 | 7,73 | 7,40 | 6,93 | 6,94 | 6,78 | 6,93 | -0,20 | 4,82 | 0,0295 | 0,2092 |
| Tgif1 | 11,53 | 11,48 | 10,83 | 11,40 | 13,11 | 11,80 | 13,01 | 14,70 | 0,21 | 4,41 | 0,0295 | 0,2092 |
| Zfp937 | 4,38 | 3,54 | 3,18 | 3,16 | 3,15 | 3,25 | 3,00 | 2,97 | -0,21 | 4,53 | 0,0298 | 0,2111 |
| Gnl3 | 10,86 | 10,94 | 8,05 | 8,81 | 10,10 | 10,75 | 12,40 | 11,92 | 0,21 | 4,35 | 0,0299 | 0,2112 |
| Evi2a-evi2b | 47,54 | 54,40 | 57,16 | 47,41 | 57,93 | 55,58 | 59,37 | 55,11 | 0,14 | 7,75 | 0,0299 | 0,2114 |
| Nsun3 | 7,32 | 5,14 | 5,63 | 6,31 | 5,38 | 4,99 | 5,74 | 3,68 | -0,30 | 2,91 | 0,0300 | 0,2120 |
| Ftsj3 | 12,45 | 14,78 | 13,93 | 12,13 | 14,57 | 15,39 | 13,80 | 16,32 | 0,17 | 5,35 | 0,0300 | 0,2121 |
| Akap8l | 23,77 | 23,11 | 25,06 | 22,72 | 28,80 | 26,79 | 23,71 | 26,41 | 0,16 | 5,70 | 0,0301 | 0,2125 |
| Gadd45a | 3,28 | 3,44 | 3,71 | 2,44 | 4,29 | 3,75 | 3,89 | 4,49 | 0,37 | 2,20 | 0,0302 | 0,2129 |
| Ercc6 | 12,03 | 9,59 | 10,69 | 10,50 | 9,80 | 9,50 | 10,03 | 9,43 | -0,14 | 6,42 | 0,0302 | 0,2129 |
| Tbc1d20 | 42,09 | 39,74 | 40,79 | 43,99 | 48,34 | 46,24 | 43,88 | 45,56 | 0,14 | 6,41 | 0,0303 | 0,2129 |
| Wdfy2 | 4,97 | 4,26 | 3,86 | 4,24 | 3,12 | 3,66 | 3,82 | 3,59 | -0,30 | 3,00 | 0,0303 | 0,2129 |
| Spag7 | 11,38 | 15,32 | 15,80 | 12,12 | 15,16 | 15,40 | 16,31 | 16,96 | 0,24 | 4,11 | 0,0304 | 0,2134 |
| Jup | 1,67 | 1,51 | 1,55 | 1,29 | 1,53 | 1,44 | 1,15 | 0,70 | -0,31 | 2,77 | 0,0305 | 0,2139 |
| Oraov1 | 12,53 | 11,03 | 10,67 | 12,31 | 13,00 | 13,53 | 13,35 | 13,86 | 0,20 | 4,63 | 0,0305 | 0,2142 |
| Dazap1 | 54,40 | 62,14 | 64,32 | 56,31 | 54,04 | 53,99 | 54,97 | 52,06 | -0,14 | 6,88 | 0,0305 | 0,2142 |
| Gripap1 | 28,88 | 27,56 | 31,46 | 25,93 | 33,00 | 32,59 | 29,75 | 30,04 | 0,14 | 6,45 | 0,0307 | 0,2154 |
| Ypel3 | 117,15 | 116,77 | 139,56 | 123,07 | 145,54 | 141,33 | 136,25 | 123,28 | 0,14 | 7,10 | 0,0308 | 0,2154 |
| March7 | 50,40 | 43,02 | 44,45 | 45,03 | 43,14 | 40,91 | 40,74 | 41,29 | -0,14 | 6,89 | 0,0308 | 0,2158 |
| Aup1 | 85,82 | 91,17 | 88,81 | 80,64 | 99,49 | 89,92 | 92,25 | 99,90 | 0,14 | 7,10 | 0,0310 | 0,2163 |
| Trim30d | 18,04 | 15,97 | 16,23 | 17,14 | 14,89 | 16,64 | 14,99 | 14,13 | -0,15 | 5,91 | 0,0310 | 0,2163 |
| Ercc6l2 | 9,21 | 8,05 | 7,92 | 8,74 | 7,41 | 7,17 | 8,07 | 7,58 | -0,17 | 5,48 | 0,0310 | 0,2166 |
| Kif1b | 13,18 | 10,38 | 10,14 | 13,02 | 11,32 | 10,15 | 10,42 | 10,59 | -0,14 | 6,82 | 0,0311 | 0,2169 |
| Alg9 | 6,12 | 6,05 | 3,91 | 5,81 | 4,94 | 4,44 | 4,74 | 4,51 | -0,25 | 3,84 | 0,0311 | 0,2170 |
| Med4 | 16,16 | 20,78 | 20,67 | 18,16 | 21,94 | 20,94 | 22,21 | 21,74 | 0,20 | 4,73 | 0,0312 | 0,2170 |
| Spg11 | 19,30 | 15,57 | 17,10 | 18,28 | 16,46 | 15,54 | 16,35 | 15,45 | -0,14 | 7,01 | 0,0312 | 0,2171 |
| B4galnt1 | 263,94 | 275,68 | 283,02 | 267,66 | 310,67 | 306,51 | 304,95 | 284,48 | 0,15 | 9,47 | 0,0313 | 0,2174 |
| Zfp64 | 9,69 | 8,72 | 9,45 | 9,08 | 10,75 | 9,81 | 11,15 | 10,60 | 0,20 | 4,78 | 0,0314 | 0,2180 |
| Dnttip1 | 15,47 | 20,21 | 22,20 | 17,46 | 22,07 | 21,55 | 21,46 | 21,28 | 0,21 | 4,66 | 0,0314 | 0,2180 |
| Mcm3 | 20,88 | 30,14 | 22,94 | 23,58 | 20,78 | 21,87 | 22,70 | 22,80 | -0,15 | 6,07 | 0,0314 | 0,2182 |
| Lyrm1 | 2,09 | 1,80 | 1,70 | 1,87 | 0,84 | 1,77 | 1,77 | 1,08 | -0,47 | 1,10 | 0,0315 | 0,2184 |
| Vps8 | 7,22 | 6,28 | 5,61 | 6,84 | 5,95 | 5,61 | 5,79 | 5,48 | -0,19 | 4,94 | 0,0316 | 0,2187 |
| Khnyn | 46,28 | 48,73 | 50,73 | 50,44 | 53,79 | 54,59 | 51,94 | 56,05 | 0,14 | 7,64 | 0,0316 | 0,2187 |
| Zbtb14 | 11,38 | 12,80 | 11,41 | 12,61 | 13,99 | 12,70 | 13,49 | 13,96 | 0,16 | 5,52 | 0,0316 | 0,2187 |
| Tango6 | 4,44 | 3,03 | 3,54 | 3,02 | 2,67 | 3,10 | 2,97 | 3,12 | -0,24 | 3,84 | 0,0316 | 0,2187 |
| Dip2a | 5,19 | 4,36 | 4,81 | 5,47 | 4,59 | 4,36 | 4,50 | 3,93 | -0,19 | 4,89 | 0,0317 | 0,2187 |
| Sin3b | 28,68 | 28,86 | 30,69 | 28,09 | 33,02 | 31,64 | 31,23 | 32,12 | 0,14 | 6,98 | 0,0317 | 0,2187 |
| D3Ertd751e | 2,27 | 1,78 | 1,85 | 2,33 | 1,78 | 1,92 | 1,70 | 1,26 | -0,31 | 2,75 | 0,0318 | 0,2193 |
| Cep350 | 32,01 | 24,37 | 27,37 | 31,50 | 27,50 | 23,70 | 26,65 | 26,39 | -0,15 | 8,51 | 0,0318 | 0,2193 |
| Mrpl9 | 46,99 | 48,70 | 44,07 | 49,00 | 51,14 | 52,11 | 51,30 | 54,14 | 0,14 | 6,32 | 0,0319 | 0,2197 |
| Bag1 | 35,91 | 45,41 | 46,83 | 35,40 | 43,98 | 43,88 | 44,93 | 48,74 | 0,16 | 5,86 | 0,0320 | 0,2198 |
| C2cd3 | 25,65 | 23,02 | 22,75 | 26,54 | 23,88 | 21,09 | 23,56 | 20,39 | -0,14 | 7,51 | 0,0320 | 0,2198 |
| Zfp654 | 27,02 | 23,18 | 23,30 | 25,52 | 22,80 | 22,75 | 21,81 | 22,64 | -0,14 | 6,81 | 0,0320 | 0,2198 |
| Tmem63b | 4,29 | 3,21 | 4,09 | 3,73 | 4,56 | 5,20 | 4,00 | 4,39 | 0,25 | 3,77 | 0,0320 | 0,2198 |
| Tnf | 5,05 | 5,54 | 3,95 | 4,66 | 6,48 | 5,76 | 5,80 | 5,45 | 0,28 | 3,18 | 0,0320 | 0,2198 |
| L1cam | 7,25 | 6,39 | 6,62 | 6,80 | 6,18 | 6,06 | 7,03 | 4,58 | -0,18 | 5,08 | 0,0321 | 0,2200 |
| Ubqln4 | 12,92 | 13,84 | 13,13 | 13,99 | 15,32 | 15,49 | 13,75 | 15,80 | 0,16 | 5,59 | 0,0321 | 0,2200 |
| Tmem64 | 3,70 | 4,19 | 4,12 | 4,16 | 4,94 | 4,54 | 4,18 | 5,10 | 0,22 | 4,36 | 0,0322 | 0,2208 |
| Znrf2 | 152,34 | 146,12 | 160,77 | 177,57 | 145,38 | 147,20 | 143,67 | 139,53 | -0,15 | 8,63 | 0,0323 | 0,2208 |
| Phf10 | 10,32 | 13,67 | 12,79 | 11,49 | 12,51 | 13,17 | 13,53 | 16,59 | 0,21 | 4,44 | 0,0326 | 0,2231 |
| E2f1 | 2,91 | 3,43 | 2,96 | 2,63 | 2,54 | 2,46 | 2,44 | 2,19 | -0,31 | 2,74 | 0,0327 | 0,2232 |
| Mrto4 | 8,74 | 10,12 | 8,24 | 11,39 | 11,98 | 11,38 | 12,06 | 11,28 | 0,26 | 3,34 | 0,0328 | 0,2237 |
| Snx15 | 7,18 | 9,00 | 8,04 | 6,55 | 9,33 | 9,22 | 8,90 | 9,09 | 0,25 | 3,71 | 0,0328 | 0,2237 |
| Rfx3 | 10,10 | 7,57 | 7,27 | 8,76 | 7,33 | 7,56 | 8,04 | 7,63 | -0,15 | 6,21 | 0,0328 | 0,2237 |
| Phactr4 | 6,88 | 7,73 | 6,91 | 6,93 | 8,98 | 7,43 | 7,70 | 8,12 | 0,18 | 5,12 | 0,0329 | 0,2238 |
| Pag1 | 32,76 | 27,20 | 30,38 | 33,68 | 29,24 | 27,50 | 28,33 | 27,33 | -0,14 | 7,93 | 0,0330 | 0,2246 |
| Mcph1 | 8,12 | 6,22 | 5,92 | 6,67 | 5,94 | 6,21 | 5,89 | 5,61 | -0,19 | 4,87 | 0,0330 | 0,2246 |
| Dynll2 | 12,31 | 14,47 | 14,28 | 13,66 | 14,39 | 14,91 | 15,13 | 17,40 | 0,18 | 5,18 | 0,0331 | 0,2247 |
| Mrpl28 | 10,19 | 11,37 | 11,28 | 9,16 | 11,76 | 12,30 | 12,75 | 13,15 | 0,26 | 3,63 | 0,0331 | 0,2247 |
| Fam83d | 1,54 | 2,04 | 1,46 | 2,02 | 1,33 | 1,11 | 1,93 | 1,09 | -0,38 | 1,87 | 0,0332 | 0,2256 |
| Ccdc55 | 2,90 | 3,57 | 4,28 | 2,75 | 4,03 | 4,04 | 3,95 | 4,00 | 0,27 | 3,53 | 0,0333 | 0,2256 |
| Thumpd1 | 17,19 | 18,54 | 17,34 | 17,18 | 19,21 | 18,99 | 19,36 | 20,98 | 0,16 | 5,65 | 0,0333 | 0,2256 |
| Npm1 | 163,16 | 201,71 | 188,54 | 173,55 | 198,82 | 192,98 | 195,34 | 214,98 | 0,14 | 8,09 | 0,0335 | 0,2269 |
| Rabggtb | 14,13 | 15,44 | 15,31 | 13,29 | 14,43 | 16,04 | 18,03 | 18,50 | 0,21 | 4,54 | 0,0335 | 0,2270 |
| Wdr78 | 3,24 | 2,00 | 2,44 | 2,91 | 2,08 | 2,18 | 2,39 | 2,11 | -0,28 | 3,21 | 0,0336 | 0,2272 |
| Rps19 | 349,23 | 379,98 | 413,16 | 321,17 | 394,13 | 417,30 | 377,72 | 423,94 | 0,14 | 7,92 | 0,0337 | 0,2277 |
| Dlg4 | 6,66 | 6,63 | 6,40 | 6,24 | 8,08 | 6,97 | 7,51 | 7,30 | 0,20 | 4,55 | 0,0337 | 0,2278 |
| Arap2 | 40,80 | 33,46 | 32,54 | 35,71 | 32,41 | 31,70 | 32,89 | 32,26 | -0,14 | 7,97 | 0,0338 | 0,2278 |
| Exosc6 | 2,70 | 5,88 | 6,49 | 4,73 | 5,81 | 6,42 | 5,68 | 6,23 | 0,31 | 2,88 | 0,0338 | 0,2278 |
| Nup155 | 9,92 | 9,11 | 9,01 | 10,64 | 8,79 | 8,34 | 9,35 | 8,37 | -0,15 | 5,88 | 0,0338 | 0,2278 |
| Smpd2 | 9,95 | 9,36 | 11,87 | 8,66 | 11,87 | 11,38 | 11,48 | 11,58 | 0,23 | 4,11 | 0,0339 | 0,2278 |
| Gtpbp2 | 54,90 | 52,51 | 55,58 | 54,52 | 61,43 | 60,16 | 56,79 | 60,93 | 0,14 | 7,41 | 0,0339 | 0,2278 |
| Abcg1 | 27,39 | 22,21 | 24,33 | 25,30 | 29,62 | 28,96 | 26,13 | 24,45 | 0,14 | 7,25 | 0,0339 | 0,2278 |
| N4bp2l1 | 32,82 | 31,70 | 35,38 | 32,34 | 37,02 | 35,97 | 35,64 | 37,56 | 0,15 | 6,05 | 0,0339 | 0,2281 |
| Gpatch11 | 4,08 | 3,81 | 4,41 | 3,35 | 4,40 | 4,74 | 4,66 | 4,39 | 0,23 | 4,16 | 0,0340 | 0,2281 |
| Fxr1 | 30,47 | 35,39 | 34,49 | 28,20 | 35,27 | 35,75 | 34,15 | 36,51 | 0,14 | 6,30 | 0,0340 | 0,2282 |
| Tom1 | 11,54 | 12,82 | 11,47 | 12,00 | 14,34 | 13,87 | 12,78 | 13,51 | 0,19 | 4,92 | 0,0341 | 0,2286 |
| Zfyve16 | 7,51 | 6,36 | 6,49 | 7,45 | 6,47 | 6,53 | 5,94 | 5,70 | -0,18 | 5,16 | 0,0341 | 0,2286 |
| Sclt1 | 2,78 | 2,19 | 1,82 | 2,05 | 1,74 | 1,90 | 1,83 | 1,64 | -0,33 | 2,61 | 0,0341 | 0,2286 |
| Adamts10 | 62,80 | 52,20 | 63,65 | 54,36 | 67,65 | 65,87 | 60,25 | 63,02 | 0,14 | 7,96 | 0,0343 | 0,2297 |
| Sh2b1 | 54,35 | 52,56 | 58,73 | 54,38 | 62,41 | 61,10 | 58,27 | 60,40 | 0,14 | 7,63 | 0,0344 | 0,2302 |
| Nt5dc1 | 20,91 | 21,26 | 19,19 | 22,13 | 18,71 | 19,66 | 19,39 | 17,32 | -0,16 | 5,75 | 0,0345 | 0,2302 |
| Camk2n1 | 1,01 | 1,15 | 1,20 | 1,15 | 1,44 | 1,55 | 1,58 | 1,16 | 0,35 | 2,25 | 0,0345 | 0,2302 |
| Zmat1 | 1,47 | 1,09 | 1,08 | 1,08 | 1,03 | 0,83 | 1,10 | 0,68 | -0,37 | 1,91 | 0,0346 | 0,2308 |
| Ccdc102a | 2,62 | 3,13 | 3,53 | 2,49 | 3,49 | 3,96 | 2,98 | 3,93 | 0,30 | 2,93 | 0,0346 | 0,2308 |
| Adamts14 | 3,94 | 3,15 | 4,05 | 3,24 | 5,35 | 3,81 | 3,84 | 3,59 | 0,22 | 4,34 | 0,0347 | 0,2309 |
| Ccnt2 | 39,26 | 31,71 | 32,56 | 34,42 | 32,89 | 31,70 | 33,49 | 27,47 | -0,14 | 6,86 | 0,0347 | 0,2310 |
| E4f1 | 18,88 | 20,45 | 22,71 | 18,40 | 23,32 | 22,20 | 20,02 | 23,79 | 0,16 | 5,77 | 0,0347 | 0,2310 |
| Akna | 214,44 | 229,33 | 233,22 | 214,84 | 255,25 | 245,86 | 241,93 | 242,31 | 0,14 | 10,33 | 0,0347 | 0,2310 |
| Maf | 4,73 | 4,55 | 4,49 | 4,09 | 3,77 | 4,21 | 3,83 | 3,38 | -0,23 | 3,91 | 0,0350 | 0,2323 |
| Emc9 | 3,65 | 4,87 | 5,87 | 3,05 | 4,49 | 5,95 | 5,70 | 6,02 | 0,38 | 2,02 | 0,0350 | 0,2327 |
| Phf19 | 3,46 | 4,08 | 3,61 | 3,55 | 3,40 | 2,72 | 3,01 | 3,26 | -0,25 | 3,67 | 0,0351 | 0,2329 |
| Pank4 | 15,97 | 15,00 | 18,62 | 15,33 | 18,53 | 19,03 | 16,60 | 18,38 | 0,17 | 5,46 | 0,0352 | 0,2331 |
| Sec22a | 2,09 | 1,78 | 1,42 | 1,76 | 1,30 | 1,51 | 1,43 | 1,46 | -0,32 | 2,69 | 0,0352 | 0,2331 |
| Tmem184b | 12,76 | 13,90 | 14,24 | 12,88 | 15,44 | 14,52 | 15,82 | 14,25 | 0,16 | 5,58 | 0,0353 | 0,2331 |
| Ncln | 55,79 | 46,96 | 55,71 | 53,53 | 64,63 | 57,89 | 55,32 | 55,08 | 0,14 | 7,32 | 0,0353 | 0,2331 |
| Rfc4 | 3,34 | 4,55 | 3,27 | 3,79 | 2,16 | 3,31 | 2,99 | 3,30 | -0,37 | 2,05 | 0,0353 | 0,2331 |
| Scaf1 | 36,46 | 37,12 | 40,19 | 35,58 | 42,50 | 40,45 | 40,55 | 40,58 | 0,14 | 7,36 | 0,0353 | 0,2331 |
| Rpgrip1 | 1,37 | 1,50 | 1,45 | 1,25 | 1,68 | 1,65 | 1,25 | 2,21 | 0,29 | 3,03 | 0,0354 | 0,2331 |
| Agtpbp1 | 8,88 | 8,04 | 8,05 | 7,36 | 6,69 | 6,69 | 7,80 | 7,40 | -0,18 | 5,10 | 0,0354 | 0,2331 |
| Zfp644 | 17,45 | 15,24 | 15,06 | 16,74 | 14,88 | 14,51 | 14,49 | 14,78 | -0,14 | 6,45 | 0,0354 | 0,2331 |
| Sesn2 | 4,67 | 5,59 | 5,49 | 4,45 | 6,32 | 5,58 | 5,03 | 6,82 | 0,24 | 3,88 | 0,0354 | 0,2331 |
| Eif5 | 65,46 | 63,16 | 61,50 | 67,84 | 72,43 | 70,35 | 66,73 | 74,87 | 0,14 | 8,07 | 0,0355 | 0,2339 |
| Rpl30 | 669,93 | 697,17 | 627,29 | 620,29 | 733,07 | 699,88 | 708,50 | 745,37 | 0,14 | 8,63 | 0,0356 | 0,2341 |
| Qsox1 | 16,90 | 17,67 | 16,66 | 16,70 | 18,80 | 18,69 | 18,91 | 18,94 | 0,15 | 5,92 | 0,0356 | 0,2342 |
| Fancb | 1,41 | 1,34 | 1,50 | 1,19 | 0,89 | 1,21 | 1,24 | 0,84 | -0,37 | 1,85 | 0,0358 | 0,2351 |
| Pstpip1 | 113,59 | 112,92 | 115,67 | 106,25 | 125,49 | 122,26 | 121,59 | 124,36 | 0,14 | 7,77 | 0,0358 | 0,2351 |
| Trrap | 45,74 | 34,69 | 38,16 | 45,40 | 40,66 | 34,23 | 38,45 | 35,25 | -0,14 | 8,93 | 0,0359 | 0,2355 |
| Rnf167 | 86,31 | 87,27 | 85,14 | 84,01 | 93,70 | 96,07 | 94,23 | 92,58 | 0,14 | 7,10 | 0,0360 | 0,2359 |
| Glipr1 | 45,11 | 45,65 | 46,02 | 42,77 | 50,83 | 48,00 | 51,24 | 49,93 | 0,16 | 5,68 | 0,0360 | 0,2360 |
| Rp2h | 7,60 | 6,93 | 7,77 | 8,30 | 6,75 | 5,92 | 7,06 | 7,03 | -0,19 | 4,76 | 0,0360 | 0,2360 |
| Bcl2l12 | 9,60 | 11,08 | 12,49 | 10,40 | 13,57 | 12,79 | 13,01 | 12,10 | 0,25 | 3,69 | 0,0361 | 0,2361 |
| Erf | 9,10 | 10,16 | 11,34 | 8,85 | 11,16 | 10,83 | 10,32 | 12,02 | 0,18 | 5,20 | 0,0362 | 0,2363 |
| Il1rl2 | 1,62 | 1,73 | 0,87 | 1,74 | 2,38 | 1,89 | 1,77 | 1,80 | 0,35 | 1,84 | 0,0362 | 0,2363 |
| Zfp799 | 5,48 | 3,98 | 4,50 | 4,41 | 3,88 | 4,14 | 4,20 | 3,57 | -0,22 | 4,18 | 0,0362 | 0,2363 |
| Psmb10 | 61,18 | 74,47 | 74,17 | 60,62 | 72,33 | 72,63 | 71,07 | 81,03 | 0,14 | 6,49 | 0,0362 | 0,2363 |
| Rilpl2 | 41,55 | 52,70 | 47,96 | 42,85 | 50,84 | 50,82 | 50,48 | 52,77 | 0,15 | 5,94 | 0,0363 | 0,2367 |
| Trim8 | 43,89 | 51,62 | 55,79 | 47,07 | 52,12 | 55,49 | 53,20 | 57,01 | 0,14 | 7,40 | 0,0363 | 0,2367 |
| Rev3l | 19,34 | 14,67 | 15,57 | 17,33 | 15,77 | 14,59 | 15,55 | 15,01 | -0,14 | 7,41 | 0,0364 | 0,2367 |
| Idh3a | 30,72 | 32,94 | 30,13 | 34,20 | 27,97 | 27,43 | 31,03 | 29,72 | -0,14 | 6,20 | 0,0364 | 0,2367 |
| Brd4 | 53,16 | 58,86 | 57,66 | 53,11 | 62,62 | 58,88 | 63,03 | 61,14 | 0,14 | 8,46 | 0,0364 | 0,2367 |
| Tacc3 | 9,78 | 13,96 | 10,27 | 10,31 | 8,41 | 9,46 | 10,81 | 10,23 | -0,19 | 4,78 | 0,0364 | 0,2367 |
| Usmg5 | 38,89 | 41,25 | 34,11 | 33,36 | 41,13 | 46,69 | 44,74 | 42,07 | 0,24 | 3,80 | 0,0365 | 0,2367 |
| Morf4l1 | 77,98 | 88,00 | 81,36 | 80,29 | 91,82 | 86,68 | 87,57 | 94,03 | 0,14 | 7,41 | 0,0365 | 0,2367 |
| Ssbp4 | 46,33 | 57,21 | 65,01 | 46,87 | 58,13 | 61,45 | 57,16 | 59,70 | 0,14 | 6,38 | 0,0365 | 0,2367 |
| Traip | 1,98 | 2,38 | 1,58 | 2,20 | 1,34 | 1,40 | 2,05 | 1,72 | -0,34 | 2,34 | 0,0365 | 0,2367 |
| Pet117 | 16,44 | 19,63 | 25,85 | 14,47 | 15,50 | 16,12 | 11,84 | 16,59 | -0,31 | 2,44 | 0,0366 | 0,2368 |
| Hist1h2ba | 14,06 | 16,59 | 15,76 | 11,62 | 11,09 | 12,86 | 11,35 | 10,56 | -0,33 | 2,33 | 0,0366 | 0,2369 |
| Il31ra | 1,17 | 1,16 | 1,37 | 1,32 | 1,63 | 1,24 | 1,14 | 2,31 | 0,34 | 2,41 | 0,0366 | 0,2369 |
| Rbl1 | 24,17 | 24,14 | 22,66 | 26,43 | 22,42 | 21,42 | 23,04 | 21,87 | -0,14 | 6,83 | 0,0367 | 0,2369 |
| Fam50a | 14,54 | 16,48 | 17,51 | 12,69 | 16,83 | 16,90 | 18,12 | 18,35 | 0,21 | 4,51 | 0,0368 | 0,2376 |
| Psat1 | 13,23 | 15,16 | 13,93 | 14,61 | 12,37 | 11,63 | 12,50 | 13,69 | -0,18 | 4,94 | 0,0369 | 0,2378 |
| Hyi | 4,85 | 4,28 | 5,00 | 4,81 | 5,43 | 5,82 | 6,95 | 5,66 | 0,34 | 2,35 | 0,0369 | 0,2378 |
| Tubb5 | 255,23 | 325,63 | 272,73 | 292,64 | 254,07 | 244,98 | 268,75 | 271,27 | -0,14 | 9,50 | 0,0369 | 0,2380 |
| Slc37a3 | 22,42 | 15,64 | 17,38 | 17,23 | 16,54 | 16,54 | 16,98 | 15,81 | -0,14 | 6,14 | 0,0370 | 0,2385 |
| Ccdc50 | 30,85 | 31,34 | 31,59 | 35,81 | 28,10 | 28,68 | 30,36 | 30,66 | -0,14 | 7,75 | 0,0371 | 0,2386 |
| Nabp1 | 56,17 | 56,57 | 61,31 | 51,94 | 51,56 | 53,93 | 54,47 | 45,66 | -0,13 | 7,26 | 0,0371 | 0,2386 |
| Cxcr4 | 13,47 | 16,43 | 9,28 | 10,33 | 12,19 | 13,28 | 15,62 | 16,04 | 0,19 | 4,61 | 0,0372 | 0,2386 |
| Cog5 | 31,42 | 24,15 | 27,48 | 28,45 | 25,25 | 26,01 | 25,83 | 24,18 | -0,14 | 6,32 | 0,0372 | 0,2386 |
| Rims3 | 1,20 | 2,01 | 1,16 | 1,71 | 1,32 | 1,24 | 0,65 | 1,06 | -0,55 | 0,45 | 0,0372 | 0,2386 |
| Ppp1r13b | 15,77 | 14,52 | 15,08 | 16,25 | 18,49 | 15,87 | 16,01 | 17,65 | 0,14 | 6,14 | 0,0372 | 0,2386 |
| Ttc1 | 16,36 | 18,21 | 18,10 | 19,24 | 17,91 | 19,53 | 22,67 | 21,94 | 0,19 | 4,79 | 0,0372 | 0,2387 |
| Pja2 | 7,84 | 6,56 | 7,96 | 7,98 | 6,89 | 6,46 | 6,94 | 6,52 | -0,17 | 5,07 | 0,0373 | 0,2390 |
| Tbc1d23 | 8,06 | 6,86 | 6,81 | 7,72 | 7,12 | 6,21 | 6,51 | 5,92 | -0,20 | 4,66 | 0,0374 | 0,2396 |
| Zc3h18 | 27,59 | 29,55 | 28,86 | 28,50 | 31,68 | 31,22 | 31,45 | 31,36 | 0,13 | 6,84 | 0,0376 | 0,2405 |
| Rbmxl1 | 21,30 | 26,53 | 23,68 | 21,86 | 26,44 | 26,05 | 25,03 | 27,07 | 0,16 | 5,42 | 0,0377 | 0,2410 |
| Spcs3 | 34,76 | 34,44 | 36,67 | 38,44 | 30,86 | 33,39 | 33,99 | 33,24 | -0,13 | 6,90 | 0,0378 | 0,2413 |
| Eif4a2 | 96,80 | 94,83 | 97,58 | 94,75 | 107,13 | 104,27 | 105,75 | 105,82 | 0,14 | 8,32 | 0,0378 | 0,2414 |
| Ttll5 | 8,15 | 5,42 | 6,37 | 6,43 | 5,87 | 5,92 | 6,31 | 5,12 | -0,18 | 4,91 | 0,0378 | 0,2414 |
| Ppp1r12b | 6,44 | 4,19 | 4,82 | 4,27 | 3,54 | 4,42 | 4,65 | 4,16 | -0,23 | 3,84 | 0,0379 | 0,2414 |
| Mrpl1 | 6,19 | 5,87 | 5,90 | 6,94 | 5,04 | 5,74 | 5,70 | 5,08 | -0,21 | 4,33 | 0,0379 | 0,2414 |
| Ifi30 | 12,10 | 16,09 | 11,66 | 8,95 | 9,30 | 11,97 | 9,40 | 10,34 | -0,26 | 3,47 | 0,0379 | 0,2415 |
| Ccnk | 34,66 | 38,32 | 35,97 | 33,76 | 39,90 | 37,80 | 38,78 | 40,23 | 0,14 | 6,62 | 0,0380 | 0,2418 |
| Acp6 | 6,36 | 5,86 | 6,41 | 5,98 | 8,38 | 6,71 | 6,88 | 7,21 | 0,25 | 3,58 | 0,0381 | 0,2419 |
| LOC102308570 | 7,25 | 11,59 | 10,85 | 6,14 | 10,66 | 11,04 | 10,78 | 9,98 | 0,26 | 3,50 | 0,0382 | 0,2423 |
| Cct3 | 38,83 | 38,09 | 37,89 | 38,31 | 41,46 | 42,09 | 39,72 | 45,33 | 0,14 | 6,30 | 0,0382 | 0,2423 |
| Rgcc | 4,50 | 5,84 | 6,10 | 4,35 | 5,08 | 6,50 | 7,20 | 7,14 | 0,33 | 2,44 | 0,0382 | 0,2424 |
| Slc5a3 | 3,89 | 2,82 | 3,23 | 3,15 | 2,72 | 2,75 | 3,06 | 3,05 | -0,18 | 5,07 | 0,0382 | 0,2424 |
| Pdcl3 | 10,78 | 17,29 | 17,79 | 12,42 | 14,50 | 16,42 | 16,29 | 19,08 | 0,20 | 4,73 | 0,0383 | 0,2429 |
| Sytl2 | 13,22 | 9,49 | 10,41 | 11,07 | 9,55 | 10,18 | 10,83 | 9,15 | -0,16 | 5,66 | 0,0384 | 0,2432 |
| Ccdc138 | 1,52 | 1,43 | 1,65 | 1,75 | 1,50 | 1,25 | 1,18 | 0,98 | -0,36 | 1,82 | 0,0385 | 0,2432 |
| Trip6 | 4,25 | 3,04 | 3,76 | 4,18 | 4,50 | 5,30 | 4,13 | 4,69 | 0,29 | 2,92 | 0,0385 | 0,2432 |
| St7l | 7,99 | 5,19 | 5,53 | 6,18 | 5,11 | 5,66 | 6,08 | 5,24 | -0,18 | 5,08 | 0,0385 | 0,2433 |
| Dedd2 | 31,59 | 30,91 | 34,87 | 32,70 | 35,45 | 38,12 | 34,47 | 35,64 | 0,15 | 5,97 | 0,0385 | 0,2433 |
| Ubc | 414,14 | 411,90 | 365,75 | 426,48 | 451,90 | 448,27 | 444,16 | 439,92 | 0,14 | 10,00 | 0,0387 | 0,2437 |
| Ldlrap1 | 20,08 | 22,59 | 22,16 | 20,58 | 24,37 | 22,12 | 21,69 | 26,32 | 0,15 | 5,91 | 0,0387 | 0,2437 |
| Fxr2 | 24,92 | 28,17 | 27,51 | 23,67 | 28,51 | 28,80 | 27,12 | 30,18 | 0,14 | 6,35 | 0,0387 | 0,2437 |
| Aldh2 | 2,45 | 2,46 | 1,71 | 2,09 | 1,85 | 2,49 | 1,21 | 1,37 | -0,36 | 2,14 | 0,0388 | 0,2437 |
| Plod3 | 2,76 | 2,65 | 2,80 | 2,63 | 3,48 | 3,37 | 3,27 | 2,88 | 0,27 | 3,31 | 0,0388 | 0,2437 |
| Erp29 | 33,08 | 37,10 | 36,14 | 33,02 | 37,42 | 39,88 | 37,07 | 41,19 | 0,16 | 5,52 | 0,0388 | 0,2437 |
| Cysltr2 | 8,58 | 7,86 | 7,19 | 8,52 | 6,83 | 6,43 | 8,16 | 5,90 | -0,24 | 3,76 | 0,0388 | 0,2437 |
| Mtfr1l | 22,42 | 21,54 | 23,13 | 22,06 | 23,86 | 24,02 | 24,90 | 26,62 | 0,16 | 5,58 | 0,0388 | 0,2437 |
| Zfp335 | 29,79 | 28,49 | 31,66 | 27,21 | 34,19 | 31,88 | 30,46 | 31,89 | 0,14 | 7,13 | 0,0388 | 0,2437 |
| Bax | 48,73 | 52,11 | 51,47 | 48,86 | 57,04 | 55,25 | 54,02 | 58,24 | 0,16 | 5,54 | 0,0389 | 0,2437 |
| Mrpl24 | 58,60 | 60,52 | 63,48 | 54,56 | 68,69 | 67,21 | 59,66 | 66,78 | 0,15 | 5,89 | 0,0389 | 0,2437 |
| Utp20 | 4,83 | 4,78 | 4,24 | 5,53 | 4,51 | 4,10 | 4,46 | 4,26 | -0,17 | 5,35 | 0,0389 | 0,2437 |
| Mrps18c | 20,88 | 25,77 | 23,36 | 20,12 | 24,08 | 25,99 | 29,76 | 26,81 | 0,24 | 3,64 | 0,0390 | 0,2437 |
| Reep4 | 18,73 | 21,60 | 22,14 | 19,70 | 23,47 | 20,85 | 21,89 | 26,16 | 0,17 | 5,19 | 0,0390 | 0,2437 |
| Surf1 | 30,69 | 37,28 | 34,36 | 29,89 | 37,55 | 33,49 | 39,31 | 37,67 | 0,17 | 5,37 | 0,0391 | 0,2447 |
| Ctso | 12,71 | 12,99 | 11,80 | 12,85 | 11,45 | 11,13 | 11,78 | 10,63 | -0,17 | 5,37 | 0,0393 | 0,2452 |
| Stx5a | 38,79 | 35,43 | 37,88 | 35,89 | 42,45 | 40,29 | 40,39 | 39,49 | 0,14 | 6,36 | 0,0393 | 0,2453 |
| Eif3i | 63,84 | 67,92 | 63,10 | 67,43 | 69,60 | 72,05 | 73,49 | 73,79 | 0,14 | 6,26 | 0,0394 | 0,2457 |
| Ccdc84 | 7,20 | 8,00 | 10,21 | 7,66 | 9,70 | 10,31 | 9,84 | 9,44 | 0,27 | 3,40 | 0,0395 | 0,2462 |
| Tef | 8,81 | 7,86 | 9,72 | 9,38 | 11,18 | 10,65 | 8,53 | 9,68 | 0,17 | 5,33 | 0,0396 | 0,2465 |
| Cbx4 | 31,53 | 30,94 | 28,37 | 31,40 | 32,99 | 34,38 | 32,99 | 33,83 | 0,13 | 7,38 | 0,0396 | 0,2465 |
| Ankle1 | 1,33 | 1,86 | 1,95 | 1,38 | 1,25 | 1,27 | 1,38 | 1,04 | -0,37 | 1,63 | 0,0396 | 0,2465 |
| Hnrnph3 | 11,94 | 13,25 | 14,13 | 11,42 | 13,53 | 14,41 | 14,03 | 15,52 | 0,19 | 4,88 | 0,0397 | 0,2470 |
| Strn4 | 27,15 | 25,65 | 27,39 | 26,20 | 29,82 | 29,90 | 26,68 | 30,31 | 0,13 | 6,63 | 0,0398 | 0,2474 |
| Swap70 | 5,69 | 6,76 | 6,56 | 6,88 | 6,80 | 6,69 | 8,63 | 7,33 | 0,19 | 4,81 | 0,0399 | 0,2476 |
| Pex26 | 4,08 | 4,25 | 4,40 | 4,49 | 4,88 | 5,22 | 4,66 | 5,18 | 0,21 | 4,26 | 0,0399 | 0,2478 |
| Zfp566 | 3,26 | 2,38 | 3,07 | 2,67 | 3,43 | 3,75 | 3,46 | 3,51 | 0,33 | 2,46 | 0,0400 | 0,2478 |
| Timm44 | 18,51 | 21,15 | 20,11 | 17,28 | 22,60 | 21,01 | 20,64 | 22,35 | 0,17 | 5,19 | 0,0402 | 0,2490 |
| Mnda | 5,89 | 6,38 | 6,42 | 6,33 | 7,82 | 7,35 | 7,26 | 7,31 | 0,25 | 3,52 | 0,0402 | 0,2490 |
| Swi5 | 23,05 | 35,54 | 29,55 | 23,75 | 33,33 | 30,77 | 31,15 | 32,98 | 0,20 | 4,54 | 0,0402 | 0,2490 |
| Arid3b | 28,65 | 25,98 | 28,34 | 26,79 | 30,57 | 32,00 | 28,98 | 28,75 | 0,13 | 6,77 | 0,0403 | 0,2493 |
| Rela | 39,46 | 41,61 | 38,16 | 39,44 | 44,99 | 42,94 | 42,62 | 43,48 | 0,13 | 6,82 | 0,0403 | 0,2493 |
| Zfp687 | 15,02 | 13,73 | 13,29 | 14,74 | 16,54 | 15,34 | 14,79 | 15,97 | 0,14 | 6,12 | 0,0404 | 0,2493 |
| Atg2a | 35,79 | 33,25 | 32,01 | 34,63 | 38,55 | 37,48 | 35,83 | 37,28 | 0,14 | 7,82 | 0,0405 | 0,2499 |
| Tsr2 | 3,50 | 2,86 | 2,34 | 2,94 | 3,29 | 3,35 | 3,69 | 3,52 | 0,24 | 3,63 | 0,0406 | 0,2502 |
| Adrm1 | 46,40 | 49,80 | 44,01 | 44,02 | 50,52 | 48,09 | 49,94 | 54,63 | 0,14 | 6,12 | 0,0406 | 0,2502 |
| Eif3f | 228,40 | 246,79 | 258,80 | 232,22 | 260,46 | 261,89 | 255,99 | 285,18 | 0,14 | 8,58 | 0,0407 | 0,2505 |
| Fam35a | 7,56 | 7,85 | 7,48 | 7,88 | 7,03 | 6,55 | 7,77 | 5,58 | -0,19 | 4,64 | 0,0407 | 0,2505 |
| Dclre1c | 6,79 | 5,51 | 5,15 | 5,74 | 5,16 | 4,74 | 5,57 | 4,76 | -0,20 | 4,47 | 0,0407 | 0,2505 |
| Ndufab1 | 7,95 | 10,17 | 9,81 | 8,27 | 10,81 | 12,09 | 9,76 | 11,19 | 0,28 | 3,02 | 0,0409 | 0,2513 |
| Serp2 | 4,33 | 4,04 | 3,71 | 2,88 | 4,39 | 4,81 | 4,93 | 5,24 | 0,37 | 1,77 | 0,0409 | 0,2515 |
| Nob1 | 16,14 | 18,81 | 14,99 | 15,54 | 17,50 | 18,63 | 17,95 | 20,43 | 0,18 | 4,86 | 0,0411 | 0,2521 |
| Mcl1 | 133,70 | 153,53 | 142,74 | 142,78 | 158,64 | 158,45 | 148,18 | 165,30 | 0,14 | 9,04 | 0,0411 | 0,2521 |
| Card6 | 28,72 | 29,03 | 29,95 | 29,87 | 32,92 | 32,80 | 31,61 | 31,50 | 0,13 | 7,13 | 0,0412 | 0,2524 |
| Angptl4 | 1,46 | 2,21 | 1,29 | 1,57 | 2,37 | 2,01 | 1,81 | 2,29 | 0,35 | 1,91 | 0,0412 | 0,2524 |
| Dpf2 | 35,01 | 34,18 | 31,32 | 33,78 | 36,50 | 35,79 | 37,59 | 37,52 | 0,13 | 6,57 | 0,0414 | 0,2535 |
| Atxn7l2 | 7,78 | 8,02 | 7,70 | 7,96 | 9,26 | 9,14 | 8,50 | 9,04 | 0,19 | 4,67 | 0,0415 | 0,2543 |
| Pgm2l1 | 14,88 | 13,39 | 14,48 | 15,96 | 13,30 | 12,49 | 15,03 | 12,78 | -0,13 | 6,92 | 0,0416 | 0,2546 |
| Arhgef2 | 95,69 | 96,89 | 102,87 | 95,98 | 113,03 | 105,93 | 101,97 | 109,82 | 0,14 | 8,83 | 0,0416 | 0,2546 |
| Hip1r | 40,62 | 46,80 | 49,83 | 43,85 | 48,29 | 51,08 | 48,52 | 50,79 | 0,14 | 7,70 | 0,0417 | 0,2549 |
| Tmtc4 | 4,95 | 3,38 | 4,71 | 4,48 | 4,02 | 3,64 | 3,74 | 3,47 | -0,23 | 3,78 | 0,0418 | 0,2550 |
| Slc39a7 | 35,35 | 38,32 | 40,78 | 37,09 | 41,82 | 42,44 | 39,14 | 42,61 | 0,13 | 6,57 | 0,0418 | 0,2550 |
| Wdr7 | 12,57 | 11,04 | 10,23 | 11,66 | 10,44 | 10,31 | 10,09 | 10,62 | -0,14 | 6,27 | 0,0419 | 0,2551 |
| Pdss2 | 4,19 | 2,87 | 3,15 | 3,68 | 2,43 | 2,87 | 2,71 | 3,21 | -0,32 | 2,49 | 0,0419 | 0,2551 |
| Hnrnpa3 | 107,53 | 134,85 | 117,25 | 108,08 | 119,95 | 121,88 | 130,15 | 142,77 | 0,14 | 9,33 | 0,0419 | 0,2551 |
| Krtcap3 | 6,21 | 5,53 | 6,38 | 5,70 | 7,57 | 7,80 | 7,23 | 6,79 | 0,31 | 2,62 | 0,0419 | 0,2551 |
| Exosc1 | 8,77 | 8,70 | 8,80 | 8,80 | 10,03 | 10,25 | 10,13 | 10,55 | 0,22 | 3,96 | 0,0420 | 0,2553 |
| Camk4 | 28,37 | 24,69 | 24,00 | 27,17 | 23,86 | 21,84 | 24,67 | 24,48 | -0,14 | 8,26 | 0,0420 | 0,2553 |
| Cyb5r1 | 5,49 | 4,67 | 3,95 | 4,25 | 6,40 | 5,61 | 4,64 | 5,66 | 0,27 | 3,08 | 0,0420 | 0,2553 |
| Scaper | 8,62 | 7,90 | 6,74 | 7,24 | 6,71 | 6,95 | 6,49 | 7,12 | -0,17 | 5,25 | 0,0421 | 0,2557 |
| Bcl2 | 5,39 | 5,37 | 4,58 | 5,96 | 5,77 | 6,27 | 5,85 | 6,02 | 0,16 | 5,36 | 0,0424 | 0,2569 |
| Phf11d | 5,45 | 4,56 | 4,47 | 4,47 | 4,44 | 3,73 | 4,18 | 3,68 | -0,24 | 3,56 | 0,0426 | 0,2580 |
| Znrf1 | 11,33 | 10,80 | 13,40 | 11,45 | 13,99 | 13,15 | 11,18 | 13,29 | 0,14 | 6,10 | 0,0426 | 0,2580 |
| Phf11c | 37,97 | 34,52 | 37,29 | 38,02 | 32,42 | 35,39 | 33,98 | 31,68 | -0,15 | 5,81 | 0,0428 | 0,2589 |
| Rab31 | 2,23 | 2,11 | 2,07 | 2,54 | 2,09 | 1,91 | 1,83 | 1,51 | -0,29 | 2,84 | 0,0428 | 0,2590 |
| Scamp3 | 58,42 | 68,02 | 69,75 | 58,93 | 72,11 | 69,72 | 69,11 | 68,23 | 0,13 | 6,63 | 0,0431 | 0,2603 |
| Ing4 | 26,98 | 28,34 | 29,48 | 28,75 | 33,31 | 30,94 | 30,66 | 31,30 | 0,15 | 5,60 | 0,0431 | 0,2603 |
| Acap1 | 138,53 | 126,11 | 139,38 | 125,66 | 145,23 | 153,64 | 139,11 | 144,15 | 0,14 | 8,43 | 0,0431 | 0,2603 |
| Mgat4a | 8,01 | 5,94 | 6,69 | 7,46 | 6,41 | 6,17 | 6,73 | 5,95 | -0,16 | 5,56 | 0,0432 | 0,2605 |
| Zbtb2 | 20,43 | 18,41 | 20,95 | 19,95 | 22,72 | 21,40 | 22,34 | 21,25 | 0,14 | 6,08 | 0,0433 | 0,2611 |
| Rtfdc1 | 37,10 | 41,59 | 39,78 | 39,37 | 43,27 | 43,18 | 41,40 | 45,18 | 0,13 | 6,47 | 0,0433 | 0,2611 |
| Zcchc17 | 11,33 | 13,37 | 12,33 | 11,57 | 14,45 | 14,32 | 13,19 | 14,24 | 0,21 | 4,22 | 0,0434 | 0,2611 |
| Dnajb2 | 1,44 | 1,30 | 1,73 | 1,69 | 2,25 | 1,88 | 1,73 | 1,78 | 0,33 | 2,43 | 0,0434 | 0,2611 |
| Galt | 13,31 | 12,15 | 10,36 | 10,44 | 13,75 | 13,60 | 13,85 | 11,70 | 0,19 | 4,64 | 0,0435 | 0,2614 |
| Mppe1 | 32,58 | 32,99 | 36,19 | 32,59 | 34,41 | 37,78 | 37,60 | 37,86 | 0,14 | 6,15 | 0,0435 | 0,2614 |
| Sap25 | 42,43 | 41,18 | 48,40 | 38,40 | 50,86 | 48,35 | 45,49 | 44,36 | 0,16 | 5,58 | 0,0435 | 0,2615 |
| Prkab1 | 26,02 | 28,77 | 28,77 | 25,84 | 30,98 | 30,11 | 30,59 | 29,09 | 0,15 | 5,89 | 0,0436 | 0,2617 |
| Zfp26 | 13,66 | 11,85 | 12,63 | 15,02 | 12,72 | 11,66 | 12,48 | 11,72 | -0,13 | 7,18 | 0,0437 | 0,2620 |
| Trmu | 3,80 | 3,80 | 4,67 | 3,42 | 5,29 | 4,18 | 4,45 | 5,18 | 0,30 | 2,75 | 0,0437 | 0,2621 |
| Fbxl12 | 16,50 | 15,17 | 14,90 | 16,21 | 17,34 | 17,32 | 17,40 | 18,55 | 0,17 | 5,16 | 0,0438 | 0,2623 |
| Tardbp | 39,05 | 38,82 | 40,04 | 43,58 | 43,18 | 43,09 | 43,64 | 47,47 | 0,14 | 8,30 | 0,0443 | 0,2653 |
| Klrb1f | 4,29 | 3,96 | 2,77 | 3,56 | 2,54 | 2,98 | 3,06 | 3,27 | -0,32 | 2,53 | 0,0444 | 0,2655 |
| Gprin3 | 44,95 | 36,82 | 37,98 | 38,33 | 37,25 | 35,31 | 37,82 | 34,10 | -0,13 | 6,87 | 0,0444 | 0,2656 |
| Slc30a4 | 2,63 | 2,92 | 3,21 | 3,28 | 3,56 | 3,30 | 3,42 | 3,62 | 0,21 | 4,16 | 0,0445 | 0,2657 |
| Rpap1 | 14,25 | 12,53 | 13,57 | 13,23 | 11,87 | 13,02 | 12,80 | 10,88 | -0,14 | 5,94 | 0,0445 | 0,2657 |
| Klf16 | 12,32 | 14,40 | 14,58 | 14,42 | 16,81 | 15,54 | 12,89 | 17,09 | 0,16 | 5,30 | 0,0445 | 0,2657 |
| Dennd1b | 31,13 | 28,28 | 28,92 | 34,52 | 27,88 | 26,54 | 29,54 | 28,06 | -0,13 | 7,91 | 0,0445 | 0,2657 |
| Mepce | 26,31 | 31,56 | 30,86 | 29,41 | 33,72 | 34,05 | 30,83 | 30,71 | 0,13 | 6,62 | 0,0446 | 0,2659 |
| Hars | 21,00 | 26,81 | 25,15 | 19,01 | 25,40 | 25,38 | 24,81 | 26,49 | 0,16 | 5,59 | 0,0446 | 0,2659 |
| Ttc17 | 21,82 | 20,71 | 21,32 | 21,97 | 20,01 | 20,04 | 20,23 | 18,08 | -0,13 | 6,56 | 0,0447 | 0,2662 |
| Map3k11 | 24,97 | 27,53 | 29,74 | 26,32 | 30,21 | 30,47 | 28,31 | 29,70 | 0,13 | 6,76 | 0,0448 | 0,2667 |
| Cd79b | 6,20 | 6,42 | 4,89 | 6,26 | 6,86 | 7,02 | 6,33 | 8,75 | 0,26 | 3,02 | 0,0449 | 0,2671 |
| Srrm1 | 52,98 | 58,37 | 55,88 | 49,02 | 59,69 | 57,98 | 59,83 | 59,51 | 0,13 | 7,73 | 0,0449 | 0,2671 |
| Rxrb | 34,71 | 31,18 | 36,96 | 34,19 | 39,77 | 38,33 | 34,26 | 37,52 | 0,13 | 6,56 | 0,0450 | 0,2671 |
| Tnks | 12,58 | 11,29 | 10,57 | 13,03 | 10,54 | 10,68 | 11,12 | 10,94 | -0,14 | 6,19 | 0,0450 | 0,2674 |
| Proser1 | 14,14 | 15,02 | 15,80 | 14,22 | 16,22 | 16,56 | 15,83 | 16,44 | 0,14 | 6,07 | 0,0452 | 0,2682 |
| Trappc12 | 20,64 | 18,82 | 21,53 | 21,73 | 19,82 | 18,88 | 19,14 | 17,27 | -0,14 | 6,06 | 0,0453 | 0,2684 |
| Trim34a | 43,13 | 36,60 | 34,27 | 44,02 | 37,00 | 35,60 | 37,59 | 34,40 | -0,13 | 6,61 | 0,0454 | 0,2684 |
| Tpd52 | 2,59 | 2,68 | 2,14 | 2,46 | 1,79 | 2,24 | 1,87 | 2,12 | -0,32 | 2,47 | 0,0454 | 0,2684 |
| Gnl2 | 12,40 | 13,89 | 10,91 | 12,04 | 13,13 | 14,06 | 14,06 | 14,48 | 0,17 | 4,96 | 0,0454 | 0,2684 |
| Pdhb | 43,79 | 46,23 | 42,41 | 41,70 | 45,84 | 46,55 | 48,83 | 50,44 | 0,14 | 6,09 | 0,0454 | 0,2684 |
| Htra2 | 6,02 | 5,60 | 4,83 | 5,15 | 7,39 | 6,02 | 5,66 | 6,72 | 0,25 | 3,38 | 0,0454 | 0,2684 |
| Tsnax | 22,83 | 25,50 | 24,88 | 22,93 | 26,07 | 25,87 | 24,53 | 29,55 | 0,14 | 5,92 | 0,0455 | 0,2684 |
| Zfp953 | 8,39 | 6,82 | 7,48 | 8,06 | 7,25 | 6,87 | 6,70 | 6,52 | -0,17 | 5,08 | 0,0455 | 0,2686 |
| Hmbs | 9,25 | 8,44 | 7,82 | 7,34 | 6,58 | 6,98 | 8,11 | 6,23 | -0,24 | 3,61 | 0,0455 | 0,2686 |
| Snw1 | 38,83 | 48,46 | 44,67 | 42,08 | 45,16 | 47,59 | 47,49 | 50,20 | 0,13 | 6,69 | 0,0456 | 0,2690 |
| Rnf181 | 43,03 | 42,63 | 47,66 | 42,72 | 52,67 | 47,94 | 44,78 | 48,19 | 0,14 | 6,00 | 0,0459 | 0,2703 |
| Rfng | 7,62 | 8,86 | 7,81 | 8,36 | 10,18 | 8,06 | 9,77 | 9,69 | 0,20 | 4,24 | 0,0460 | 0,2708 |
| Faah | 25,29 | 26,15 | 25,77 | 26,82 | 28,01 | 27,61 | 28,16 | 30,07 | 0,13 | 6,70 | 0,0460 | 0,2708 |
| Dhrs11 | 5,16 | 4,10 | 4,33 | 3,97 | 5,87 | 5,92 | 4,22 | 5,40 | 0,29 | 2,82 | 0,0461 | 0,2710 |
| Smchd1 | 63,17 | 58,03 | 56,10 | 61,16 | 54,08 | 53,96 | 54,99 | 54,11 | -0,14 | 8,65 | 0,0462 | 0,2716 |
| Adamts6 | 12,43 | 8,21 | 8,77 | 10,83 | 9,05 | 8,32 | 10,14 | 8,74 | -0,16 | 5,53 | 0,0463 | 0,2716 |
| Rabac1 | 39,78 | 35,90 | 41,29 | 41,52 | 44,45 | 45,49 | 42,70 | 44,84 | 0,17 | 5,20 | 0,0463 | 0,2716 |
| Psmb8 | 158,01 | 203,60 | 207,70 | 156,56 | 194,79 | 203,17 | 189,88 | 207,20 | 0,13 | 7,86 | 0,0463 | 0,2718 |
| Gpr132 | 60,29 | 54,75 | 60,29 | 63,37 | 65,92 | 65,40 | 62,02 | 67,69 | 0,13 | 7,25 | 0,0465 | 0,2726 |
| Zfp358 | 1,79 | 2,33 | 2,51 | 2,08 | 2,86 | 2,52 | 2,70 | 2,75 | 0,33 | 2,32 | 0,0466 | 0,2728 |
| Zfp433 | 5,16 | 3,64 | 4,80 | 4,30 | 3,99 | 3,99 | 3,56 | 3,40 | -0,25 | 3,29 | 0,0467 | 0,2729 |
| Tatdn2 | 49,53 | 42,95 | 42,71 | 46,68 | 46,68 | 49,07 | 48,47 | 54,75 | 0,13 | 6,98 | 0,0467 | 0,2729 |
| H2-Q1 | 20,34 | 17,75 | 17,90 | 21,17 | 21,02 | 23,52 | 19,58 | 22,12 | 0,16 | 5,38 | 0,0467 | 0,2729 |
| Ahi1 | 4,43 | 3,51 | 3,18 | 3,41 | 2,78 | 3,16 | 3,18 | 3,44 | -0,22 | 4,05 | 0,0468 | 0,2732 |
| Ms4a6d | 5,89 | 6,08 | 5,79 | 6,37 | 7,69 | 8,30 | 6,52 | 6,46 | 0,26 | 3,21 | 0,0468 | 0,2732 |
| Creld2 | 8,70 | 11,43 | 10,12 | 9,85 | 11,86 | 10,42 | 12,98 | 11,50 | 0,22 | 3,90 | 0,0468 | 0,2733 |
| Nudt5 | 12,75 | 11,16 | 12,44 | 13,55 | 10,10 | 11,67 | 11,09 | 10,43 | -0,21 | 4,21 | 0,0469 | 0,2735 |
| Kdm4c | 29,17 | 24,41 | 26,10 | 26,85 | 24,82 | 23,35 | 24,84 | 24,42 | -0,13 | 6,76 | 0,0469 | 0,2735 |
| Gsap | 22,33 | 17,39 | 19,55 | 19,22 | 17,78 | 17,76 | 18,43 | 17,46 | -0,14 | 6,13 | 0,0470 | 0,2737 |
| Maf1 | 61,79 | 55,98 | 68,35 | 65,10 | 70,43 | 71,87 | 63,65 | 68,40 | 0,13 | 6,85 | 0,0472 | 0,2748 |
| Nsl1 | 5,41 | 4,10 | 3,66 | 4,27 | 3,63 | 3,19 | 4,55 | 3,49 | -0,24 | 3,60 | 0,0472 | 0,2748 |
| Uba6 | 13,73 | 12,11 | 12,70 | 13,59 | 12,21 | 11,68 | 12,18 | 11,29 | -0,14 | 5,98 | 0,0473 | 0,2749 |
| Mthfd1 | 4,92 | 6,56 | 5,23 | 5,71 | 4,48 | 4,83 | 5,00 | 5,13 | -0,21 | 4,13 | 0,0473 | 0,2749 |
| Ube2v1 | 77,82 | 86,08 | 79,10 | 74,51 | 87,81 | 86,11 | 85,15 | 88,09 | 0,13 | 7,34 | 0,0473 | 0,2749 |
| Ciz1 | 21,34 | 23,51 | 22,62 | 19,68 | 25,86 | 23,18 | 23,98 | 22,65 | 0,14 | 6,11 | 0,0475 | 0,2752 |
| Celsr1 | 21,94 | 17,57 | 19,20 | 22,34 | 20,01 | 17,45 | 18,23 | 18,32 | -0,13 | 7,74 | 0,0475 | 0,2752 |
| Zfyve26 | 12,80 | 11,70 | 11,87 | 14,03 | 12,01 | 10,99 | 11,97 | 11,16 | -0,13 | 6,82 | 0,0475 | 0,2752 |
| Gnl1 | 11,98 | 12,73 | 13,27 | 12,26 | 13,37 | 15,55 | 12,57 | 14,75 | 0,16 | 5,20 | 0,0475 | 0,2752 |
| Orc4 | 16,78 | 13,71 | 15,84 | 13,87 | 13,66 | 14,47 | 12,76 | 13,61 | -0,14 | 5,85 | 0,0477 | 0,2761 |
| Ppp1r2 | 13,47 | 12,34 | 12,49 | 13,66 | 11,33 | 12,25 | 11,60 | 11,73 | -0,15 | 5,66 | 0,0478 | 0,2768 |
| Grpel1 | 9,66 | 9,38 | 8,24 | 9,86 | 10,12 | 10,32 | 9,97 | 11,46 | 0,17 | 5,05 | 0,0479 | 0,2771 |
| Gstcd | 6,19 | 4,79 | 5,42 | 5,67 | 4,91 | 4,17 | 4,77 | 4,99 | -0,23 | 3,75 | 0,0480 | 0,2771 |
| Fhod1 | 19,40 | 17,47 | 18,92 | 17,97 | 21,95 | 20,81 | 17,10 | 20,98 | 0,13 | 6,26 | 0,0480 | 0,2771 |
| Ctxn1 | 1,13 | 1,52 | 1,39 | 2,04 | 1,58 | 2,63 | 2,00 | 1,94 | 0,39 | 1,21 | 0,0481 | 0,2775 |
| Rrad | 7,81 | 9,46 | 10,09 | 8,13 | 9,72 | 9,95 | 10,35 | 11,15 | 0,22 | 3,92 | 0,0482 | 0,2777 |
| Fig4 | 14,77 | 13,26 | 13,53 | 14,59 | 12,53 | 12,41 | 13,37 | 12,16 | -0,16 | 5,45 | 0,0482 | 0,2781 |
| Ndufaf2 | 1,26 | 1,87 | 2,78 | 1,37 | 2,91 | 2,44 | 1,80 | 2,69 | 0,51 | 0,63 | 0,0484 | 0,2787 |
| Gadd45b | 11,91 | 14,90 | 17,55 | 13,05 | 15,63 | 18,46 | 14,85 | 16,76 | 0,21 | 4,29 | 0,0484 | 0,2787 |
| Atp6v1c1 | 31,99 | 28,54 | 27,45 | 30,31 | 27,19 | 27,28 | 26,81 | 26,15 | -0,14 | 5,89 | 0,0486 | 0,2792 |
| Hmces | 10,41 | 13,13 | 12,30 | 10,42 | 12,51 | 13,32 | 12,97 | 14,59 | 0,21 | 4,14 | 0,0486 | 0,2792 |
| Phactr2 | 2,03 | 1,93 | 1,88 | 2,01 | 1,87 | 1,74 | 1,67 | 1,49 | -0,22 | 3,97 | 0,0486 | 0,2792 |
| Zdhhc6 | 19,16 | 18,93 | 20,37 | 20,56 | 16,35 | 19,61 | 18,57 | 16,40 | -0,15 | 5,42 | 0,0486 | 0,2793 |
| Cst7 | 109,76 | 110,54 | 102,82 | 108,71 | 124,80 | 116,45 | 122,62 | 108,13 | 0,13 | 6,80 | 0,0488 | 0,2799 |
| Dnajc15 | 193,67 | 188,99 | 200,38 | 183,95 | 181,53 | 168,79 | 178,38 | 173,08 | -0,13 | 6,89 | 0,0488 | 0,2800 |
| Bcorl1 | 6,39 | 6,37 | 6,70 | 5,97 | 7,52 | 6,47 | 7,03 | 7,30 | 0,16 | 5,37 | 0,0489 | 0,2801 |
| Brf1 | 25,76 | 27,70 | 28,25 | 23,28 | 27,64 | 30,28 | 25,30 | 31,77 | 0,13 | 6,24 | 0,0489 | 0,2802 |
| Kcnc1 | 2,90 | 2,24 | 3,06 | 3,02 | 2,37 | 1,98 | 3,26 | 2,13 | -0,19 | 4,35 | 0,0489 | 0,2802 |
| Doc2g | 1,65 | 2,28 | 1,96 | 1,97 | 2,09 | 3,03 | 2,53 | 2,51 | 0,36 | 1,71 | 0,0490 | 0,2805 |
| Usp24 | 44,65 | 33,39 | 36,17 | 43,09 | 36,86 | 34,16 | 36,40 | 36,00 | -0,13 | 8,64 | 0,0491 | 0,2805 |
| Folr4 | 2,57 | 3,18 | 1,88 | 1,91 | 1,68 | 1,81 | 1,80 | 2,15 | -0,39 | 1,53 | 0,0491 | 0,2805 |
| Nol10 | 15,31 | 12,64 | 12,48 | 14,47 | 11,60 | 12,84 | 13,49 | 11,32 | -0,16 | 5,28 | 0,0492 | 0,2810 |
| Zfp563 | 2,19 | 1,73 | 2,02 | 2,17 | 1,74 | 1,76 | 1,74 | 1,64 | -0,23 | 3,58 | 0,0493 | 0,2810 |
| BC068281 | 3,92 | 2,94 | 3,38 | 3,44 | 3,72 | 4,27 | 3,91 | 4,28 | 0,24 | 3,47 | 0,0493 | 0,2810 |
| Prelid1 | 179,42 | 205,25 | 210,94 | 174,97 | 206,19 | 204,48 | 217,09 | 215,20 | 0,13 | 7,78 | 0,0493 | 0,2813 |
| Ep400 | 51,06 | 42,19 | 42,61 | 49,41 | 44,58 | 39,48 | 43,64 | 41,23 | -0,13 | 8,90 | 0,0494 | 0,2813 |
| Gpr114 | 12,93 | 11,31 | 14,89 | 13,16 | 13,72 | 15,54 | 13,80 | 15,19 | 0,16 | 5,29 | 0,0494 | 0,2813 |
| Slc25a24 | 15,76 | 16,46 | 16,94 | 16,77 | 14,26 | 15,32 | 15,85 | 14,17 | -0,14 | 5,73 | 0,0495 | 0,2816 |
| Drap1 | 47,87 | 55,00 | 48,42 | 50,75 | 58,81 | 53,24 | 53,53 | 58,60 | 0,15 | 5,61 | 0,0495 | 0,2816 |
| Psmd4 | 44,12 | 58,89 | 57,00 | 50,53 | 57,53 | 61,36 | 55,60 | 56,10 | 0,13 | 6,21 | 0,0497 | 0,2816 |
| Fmr1 | 26,57 | 28,38 | 25,34 | 27,63 | 23,48 | 25,00 | 25,98 | 24,44 | -0,13 | 6,83 | 0,0497 | 0,2816 |
| Pfkl | 12,13 | 13,04 | 12,80 | 10,69 | 13,53 | 13,38 | 13,29 | 13,72 | 0,15 | 5,58 | 0,0497 | 0,2816 |
| Rnf113a2 | 7,06 | 10,09 | 9,67 | 8,82 | 10,33 | 11,33 | 9,02 | 10,98 | 0,23 | 3,75 | 0,0497 | 0,2816 |
| Kdelc2 | 3,54 | 2,86 | 2,72 | 3,53 | 2,67 | 2,41 | 2,80 | 2,85 | -0,25 | 3,43 | 0,0498 | 0,2816 |
| Spr | 8,50 | 10,43 | 11,26 | 8,44 | 10,91 | 10,73 | 10,29 | 13,16 | 0,24 | 3,69 | 0,0499 | 0,2816 |
| Parp9 | 40,96 | 41,80 | 40,25 | 46,03 | 39,07 | 39,21 | 38,32 | 38,28 | -0,13 | 7,17 | 0,0499 | 0,2816 |
| Ptbp3 | 139,08 | 127,06 | 136,00 | 147,58 | 127,99 | 120,89 | 127,00 | 125,32 | -0,13 | 9,82 | 0,0499 | 0,2816 |
| Elp4 | 3,40 | 3,13 | 2,59 | 3,20 | 2,45 | 2,80 | 3,03 | 2,22 | -0,24 | 3,49 | 0,0499 | 0,2816 |
| Ddx46 | 42,63 | 47,45 | 46,27 | 41,27 | 47,83 | 47,51 | 47,09 | 52,01 | 0,13 | 8,05 | 0,0500 | 0,2816 |
| Mrpl39 | 7,43 | 9,70 | 9,15 | 8,06 | 9,81 | 8,61 | 11,36 | 9,81 | 0,21 | 4,11 | 0,0500 | 0,2816 |
| Xpr1 | 15,73 | 13,42 | 14,92 | 16,07 | 13,75 | 13,25 | 14,35 | 13,72 | -0,13 | 6,78 | 0,0500 | 0,2816 |

Table. S4.
[truncated: 14,822 more chars]
